# DFT Insights into NHC-Catalyzed Switchable [3+4] and [3+2] Annulations of Isatin-Derived Enals and N-Sulfonyl Ketimines: Mechanism, Regio- and Stereoselectivity

**DOI:** 10.3390/molecules30214218

**Published:** 2025-10-29

**Authors:** Saisai Yu, Wenxin Zhou, Yueming Jiang, Hangyu Wang, Xiaoyu Zhou, Shengwen Yang

**Affiliations:** 1 Key Laboratory of Xinjiang Endemic Phytomedicine Resources, Ministry of Education, School of Pharmacy, Shihezi University, Shihezi 832002, China; 20232115021@stu.shzu.edu.cn (S.Y.); 20222015040@stu.shzu.edu.cn (W.Z.); jiangym@shzu.edu.cn (Y.J.); 2 School of Pharmaceutical and Chemical Engineering, Taizhou University, Taizhou 317700, China; 3 Yunnan Key Laboratory of Chiral Functional Substance Research and Application, Yunnan Minzu University, Kunming 650504, China

**Keywords:** N-heterocyclic carbene, spirocyclopentane oxindole, [3+4] annulation, [3+2] annulation, isatin-derived enal

## Abstract

Density functional theory (DFT) calculations at the M06-2X-D3/6-311++G(2df,2pd) level elucidate the mechanism and selectivity origins in the NHC-catalyzed divergent synthesis of spirocyclopentane oxindoles from isatin-derived enals and N-sulfonyl ketimines. The Michael addition constitutes the regio- and stereoselectivity-determining step, where Parr function analysis demonstrates that nucleophile/electrophile electrophilicity governs regioselectivity, while distortion/interaction and non-covalent interaction analyses reveal stereoselectivity is controlled by distortion and weak interactions. K_3_PO_4_ facilitates Breslow intermediate formation and proton transfer toward the *β*-lactam-fused spirocyclopentane oxindole, whereas N,N-diisopropylethylamine (DIPEA) promotes these processes for the spirocyclopentane oxindole bearing an enaminone moiety. Catalyst roles are also further delineated.

## 1. Introduction

**Figure 1:**
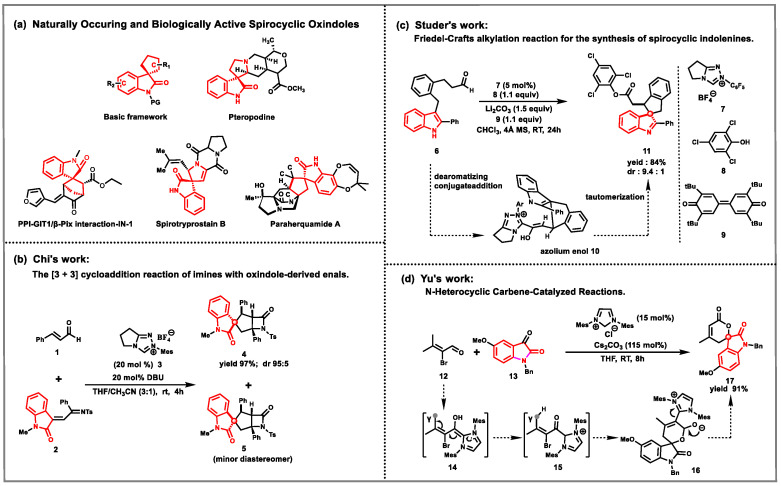
Representative examples of natural products containing the spirocyclopentane oxindole scaffold and related research.

Spirocyclopentane oxindoles have attracted considerable interest in the fields of medicinal chemistry and organic synthesis, owing to their intricate structural features and potential applications in the domain of drug discovery [1,2,3]. Molecules endowed with spirocyclopentane oxindole not only augment the molecular rigidity and stereoconfiguration but also affords avenues for the nuanced modulation of the resultant molecules’ pharmacokinetic and pharmacodynamic attributes [4,5,6,7]. These compounds have been identified to possess diverse biological activities, including anti-inflammatory [8,9], anticancer [10], and antimicrobial properties [11,12] (Figure 1a). Stereochemical control for the synthesis of spirocyclopentane oxindole is crucial for ensuring the biological activity of the compounds.

Since the successful isolation and characterization of N-heterocyclic carbenes (NHCs) in 1991, NHCs have been widely recognized as powerful tools for enantioselective C-C bond formation, particularly in the synthesis of spirocyclic cyclopentane-fused oxindole molecules, Chi [13,14,15,16], Studer [17], Yu [18,19,20] and others have done a lot of excellent work [21,22,23,24] (Figure 1b–d). By modulating the electronic and steric properties of NHCs and substrates, they enable asymmetric syntheses of reactive intermediates with highly enantioenriched structures, such as acyl anion [25,26], acyl azoles [27,28,29,30,31,32], enolates [33,34,35,36,37,38] and homoenolates [39,40,41,42,43], to achieve selective transformations. Despite significant advances in switchable syntheses via catalytic regiodivergent processes [44,45,46], the development of switchable synthetic platforms that exploit a single substrate mode through distinct NHC catalysts remains a formidable challenge.

**Figure 2:**
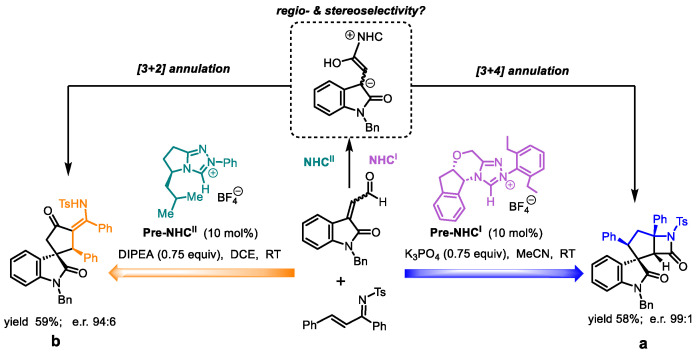
NHC-Catalyzed Switchable [3+4] and [3+2] Annulations of Isatin-Derived Enals and N-Sulfonyl Ketimines (**a**: *β*-Lactam fused spirocyclopentane oxindole; **b**: spirocyclopentane oxindoles bearing an enaminone moiety).

A variety of theoretical studied on NHC-catalyzed reactions have an important progress [47,48,49,50]. Notably, NHCs are commonly used in the synthesis of spirocyclization reactions. In this context, the switchable spirocyclization reaction developed by Enders and co-workers was the first to employ an NHC catalyst, which can catalyze indigo-derived enals and N-sulfonyl ketimines to yield different spirocyclic pentane oxindoles (Figure 2) [51]. Under **Pre-NHC^I^** catalysis, the reaction employing K_3_PO_4_ as the base in acetonitrile (MeCN) solvent affords spirocyclopentane oxindole scaffolds bearing a *β*-lactam moiety. Conversely, when mediated by **Pre-NHC^II^** with N,N-diisopropylethylamine (DIPEA) as the base in dichloroethane (DCE), the protocol selectively generates analogous spirocyclic architectures functionalized with an enaminone group, demonstrating precise catalyst-controlled divergent synthesis. Nevertheless, the detailed mechanism of NHC-controlled asymmetric reaction has remained unclear.

Herein, we employed the mechanistically unexplored NHC-catalyzed switchable formation of spirocyclopentane oxindoles from isatin-derived enals and N-sulfonyl ketimines as a computational model (Figure 2). Density functional theory (DFT) studies were conducted to elucidate the reaction mechanism, regio- and stereoselectivity, as well as the role of the NHC catalyst.

## 2. Results and Discussion

### 2.1. Reaction Mechanism

**Figure 3:**
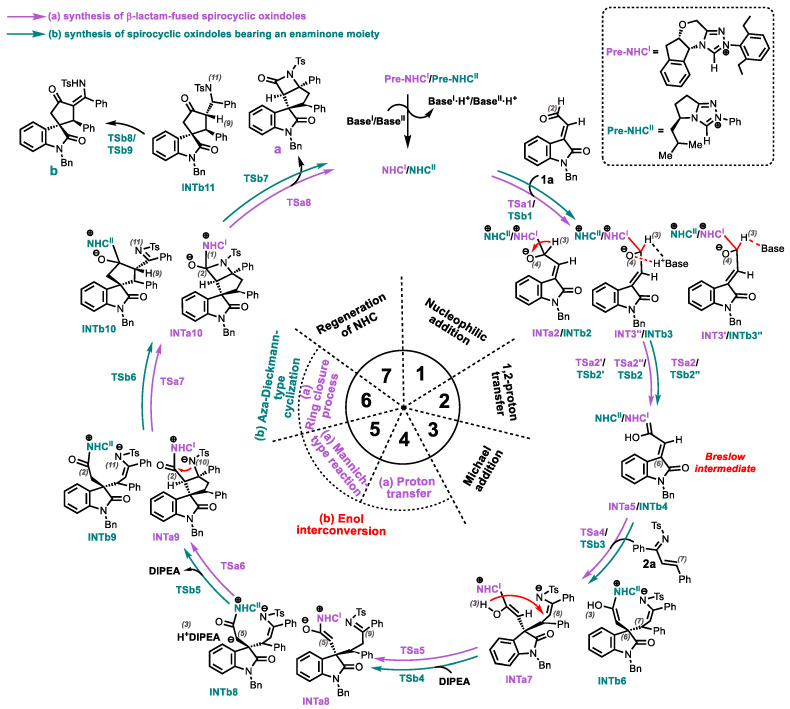
A plausible catalytic cycle of the reaction.

Based on the mechanistic hypothesis of Enders’ group [51] and the computational reports by Tang [52], Chen [53], and Zhang [54] et al., a plausible catalytic mechanism has been postulated for the NHC-catalyzed [3+4] and [3+2] annulation reactions of isatin-derived enals with N-sulfonylketimines, respectively, as depicted in Figure 3. In the catalytic pathway affording product **a** (purple cycle), the precatalyst **Pre-NHC^I^** undergoes base-mediated (K_3_PO_4_) deprotonation to generate the active catalyst **NHC^I^**. The ensuing seven-step catalytic manifold comprises: (1) Nucleophilic addition, (2) Breslow intermediate generation through 1,2-proton transfer, (3) Michael addition, (4) 1,3-proton transfer, (5) Mannich-type reaction, (6) Cyclization, and (7) Catalyst elimination. Conversely, the product **b** formation pathway (green cycle) initiates with DIPEA-promoted deprotonation of **Pre-NHC^II^** to access catalytically active catalyst **NHC^II^** species. This divergent mechanism features: (1) Nucleophilic addition, (2) Breslow intermediate generation through 1,2-proton transfer, (3) Michael addition, (4) Enol-tautomerism, (5) Aza-Dieckmann-type cyclization, (6) Catalyst elimination, and (7) Tautomerization. The detailed mechanisms of the reactions will be discussed step by step below.

Product Generation Process for Compound **a**:

Step 1: **NHC^I^** Generation & Nucleophilic Addition

Following the above proposal, calculations were performed to reveal the mechanism of the **NHC^I^**-catalyzed [3+4] annulation reactions of isatin-derived enals **1a** with N-sulfonylketimines **2a**. The transformation begins with the barrierless K_3_PO_4_-mediated deprotonation of **Pre-NHC^I^** to provide the **NHC^I^**. This process is highly exergonic by 28.0 kcal/mol (Figure S1, Please see Supplementary Information for details).

**Figure 4:**
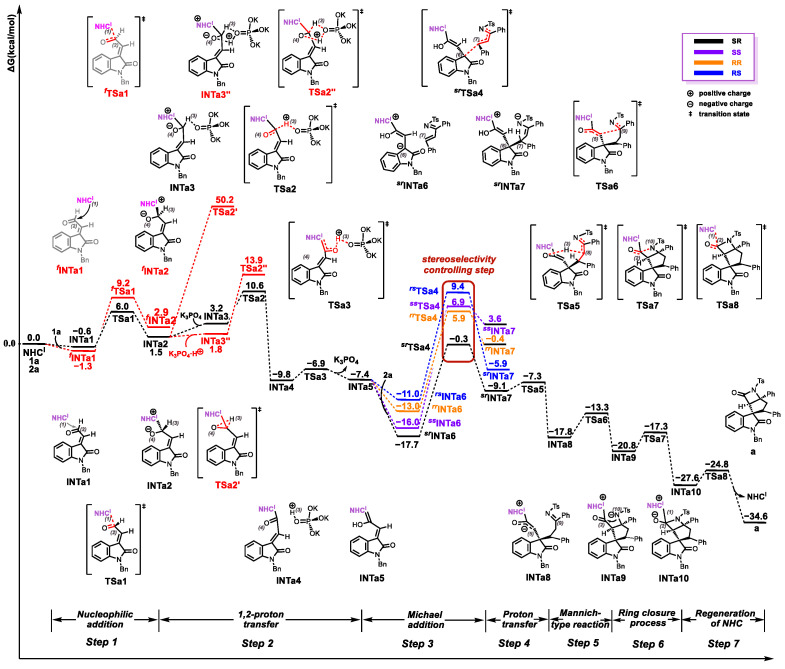
The Gibbs free energy profile of the process of generating product **a**.

Next, coordination of **NHC^I^** to **1a** through an *n*→π* interaction between the carbene carbon and the carbonyl group delivers the complex **INTa1** with an energy release of 0.6 kcal/mol (see Figure 4). Nucleophilic addition of **NHC^I^** to **1a** via the reverse-side transition state **TSa1** (6.0 kcal/mol) affords intermediate **INTa2** (1.5 kcal/mol). The stereochemical alternative involving front-side nucleophilic attack of **NHC^I^** on **1a** was also evaluated. Calculations revealed that the energy barrier for the front-side transition state ***^f^*TSa1** (∆*G*^‡^ = 10.5 kcal/mol) is 3.9 kcal/mol higher than that of the reverse-side transition state **TSa1** (∆*G*^‡^ = 6.6 kcal/mol), indicating that the reverse-side pathway is energetically favored.

Step 2: Formation of the Breslow Intermediate

We initially explored the direct proton transfer pathway from **INTa2**. However, the calculated energy barrier for the corresponding transition state **TSa2′** was prohibitively high (∆*G*^‡^ = 48.7 kcal/mol relative to **INTa2**). We then evaluated an alternative pathway involving proton (H3) migration assisted by K_3_PO_4_·H^+^ via transition state **TSa2″**. This pathway exhibited a significantly reduced barrier (∆*G*^‡^ = 12.4 kcal/mol relative to **INTa2**). Additionally, we investigated the K_3_PO_4_-assisted mechanism. In this pathway, **INTa2** forms a hydrogen-bonded complex **INTa3** with K_3_PO_4_, which is endergonic by 3.2 kcal/mol. Subsequently, proton migration within **INTa3** via transition state **TSa2** affords complex **INTa4**. This step requires an energy barrier of 9.1 kcal/mol relative to **INTa2** and is exergonic (∆*G* = −11.3 kcal/mol relative to **INTa2**). Crucially, the barrier for this step (∆*G*^‡^ = 9.1 kcal/mol) is lower than both the direct proton transfer barrier (∆*G*^‡^ = 48.7 kcal/mol) and the K_3_PO_4_·H^+^-assisted barrier (∆*G*^‡^ = 12.4 kcal/mol). Finally, the proton from the K_3_PO_4_·H^+^ moiety in **INTa4** migrates to O4 via transition state **TSa3** (∆*G*^‡^ = 2.9 kcal/mol relative to **INTa4**), generating the Breslow intermediate **INTa5** and releasing K_3_PO_4_. Therefore, the K_3_PO_4_-assisted pathway is energetically favored for the formation of the Breslow intermediate.

Step 3: Michael Addition

**Figure 5:**
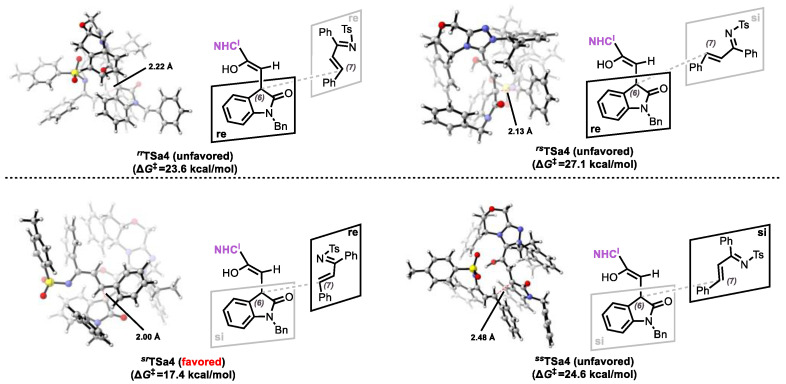
Four stereochemical configurations are possible for the Michael addition.

Following the generation of intermediate **INTa5**, a stereoselective Michael addition with **2a** proceeds to form intermediate **INTa7**. Considering the prochiral of both reacting partners (**INTa5** and **2a**), we systematically evaluated four distinct facial addition patterns: *re*-*re*, *re*-*si*, *si*-*re*, and *si*-*si*, corresponding to the formation of 6*R*,7*R*-, 6*R*,7*S*-, 6*S*,7*R*-, and 6*S*,7*S*-configured adducts, respectively. Transition state analysis revealed activation barriers of 17.4 kcal/mol (***^sr^*TSa4**), 24.6 kcal/mol (***^ss^*TSa4**), 23.6 kcal/mol (***^rr^*TSa4**), and 27.1 kcal/mol (***^rs^*TSa4**) (see Figure 5). The observed preference for 6*R*,7*R*-configuration formation, consistent with experimental results, can be rationalized by the lowest energy transition state (***^sr^*TSa4**, ∆*G*^‡^ = 17.4 kcal/mol).

Step 4: Proton Transfer

Proton transfer from the hydroxyl group (H3) of intermediate ***^sr^*INTa7** to the adjacent C8 atom occurs via **TSa5** (∆*G*^‡^ = 1.8 kcal/mol), exergonically forming **INTa8** (∆*G* = −8.7 kcal/mol). This thermodynamically driven process is facilitated by optimal spatial proximity between H3 and C8.

Step 5: Mannich-type Reaction

The enol-structured intermediate **INTa8**, bearing strong nucleophilicity at the C5 position, undergoes a Mannich reaction with the electrophilic C9 position via a five-membered ring transition state **TSa6**. This process surmounts an activation barrier of 4.5 kcal/mol, yielding the more stable five-membered ring intermediate **INTa9** with an exergonicity of 3.0 kcal/mol relative to **INTa8**.

Step 6: Formation of the Four-membered Ring

Intermediate **INTa9**, featuring an electrophilic carbonyl carbon atom at the C2 position, undergoes nucleophilic addition with the N-tosylamide anion. This reaction proceeds via four-membered ring transition state **TSa7**, yielding a *β*-lactam intermediate **INTa10**. This process has an energy barrier of 3.5 kcal/mol and liberates 6.8 kcal/mol of free energy relative to intermediate **INTa9**.

Step 7: Catalyst Regeneration

Finally, intermediate **INTa10** undergoes catalyst **NHC^I^** regeneration via transition state **TSa8**, affording product **a**. This step requires overcoming an energy barrier of only 2.8 kcal/mol and liberates 7.0 kcal/mol of free energy relative to intermediate **INTa10**.

Product Generation Process for Compound **b**:

Step 1: Nucleophilic Addition

The NHC^II^-catalyzed [3+4] annulation commences with DIPEA-mediated deprotonation of **Pre-NHC^II^**, generating the active catalyst **NHC^II^**. This step proceeds via transition state **TS-NHC^II^** with a calculated barrier of ∆*G*^‡^ = 9.7 kcal/mol and is endergonic (∆*G* = +6.3 kcal/mol), both relative to the ZPE-corrected ground state of **Pre-NHC^II^** and DIPEA (Figure S2). This contrasts sharply with the barrierless formation of the alternative active catalyst **NHC^I^**.

**Figure 6:**
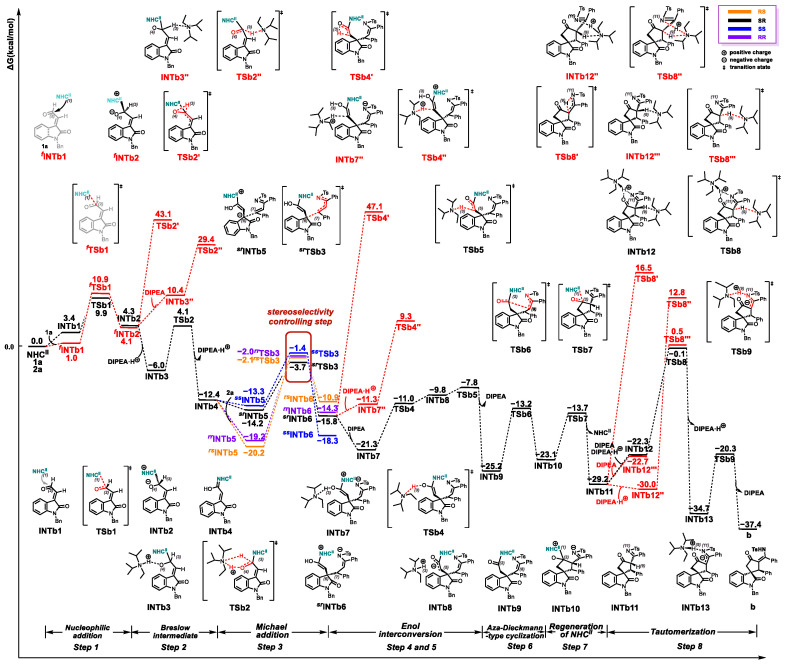
The Gibbs free energy profile of the process of generating product **b**.

As depicted in Figure 6, coordination of **NHC^II^** to **1a** via an *n*→π* interaction between the carbene carbon and the carbonyl group forms complex **INTb1**, which is endergonic by 3.4 kcal/mol. Nucleophilic addition of **NHC^II^** to **1a** then proceeds via the reverse-side transition state **TSb1** (∆*G*^‡^ = 9.9 kcal/mol), affording intermediate **INTb2** (∆*G* = +4.3 kcal/mol). For comparison, the stereochemical alternative involving frontside attack was evaluated. Calculations reveal that the barrier for the front-side transition state ***^f^*TSb1** (∆*G*^‡^ = 10.9 kcal/mol) is 1.0 kcal/mol higher than that for **TSb1** (∆*G*^‡^ = 9.9 kcal/mol), indicating that the reverse-side pathway is energetically favored.

Step 2: Formation of the Breslow Intermediate

Three distinct pathways for Breslow intermediate formation were evaluated. First, the direct proton transfer from **INTb2** via transition state **TSb2′** was excluded due to its prohibitively high barrier (∆*G*^‡^ = 38.8 kcal/mol relative to **INTb2**). Second, the DIPEA-assisted proton (H3) migration via transition state **TSb2″** exhibited a similarly high barrier (∆*G*^‡^ = 25.3 kcal/mol relative to **INTb2**). Third, the DIPEA·H^+^-assisted mechanism was investigated. In this pathway: (i) **INTb2** forms a hydrogen-bonded complex **INTb3** with DIPEA·H^+^, which is exergonic (∆*G* = −10.3 kcal/mol relative to **INTb2**). (ii) Proton migration within **INTb3** through a hydrogen-bonded transition state **TSb2** affords the Breslow intermediate **INTb4**. This step requires a barrier of ∆*G*^‡^ = 10.1 kcal/mol and is exergonic (∆*G* = −6.4 kcal/mol), both relative to **INTb3**. Consequently, the DIPEA·H^+^-assisted pathway is energetically favored for Breslow intermediate formation.

Step 3: Michael Addition

**Figure 7:**
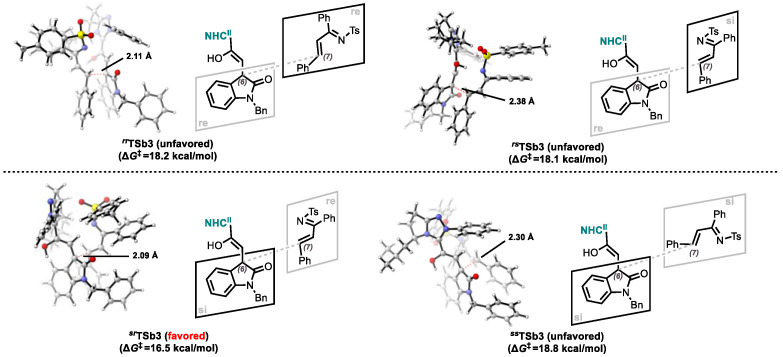
Four stereochemical configurations are possible for the Michael addition.

Given the prochiral of both reaction partners (**INTb4** and **2a**), four stereochemically distinct pathways exist for the Michael addition of **INTb4** to **2a** to form **INTb6** via transition state **TSb3**. These correspond to the *re*-facial/*re*-facial (*re*/*re*), *re*-facial/*si*-facial (*re*/*si*), *si*-facial/*re*-facial (*si*/*re*), and *si*-facial/*si*-facial (*si*/*si*) addition modes, yielding the (*6R*,*7R*)-, (*6R*,*7S*)-, (*6S*,*7R*)-, and (*6S*,*7S*)-configured adducts, respectively. Transition state analysis revealed the following activation barriers: ∆*G*^‡^ = 18.2 kcal/mol for ***^rr^*TSb3** (*re*/*re*), ∆*G*^‡^ = 18.1 kcal/mol for ***^rs^*TSb3** (*re*/*si*), ∆*G*^‡^ = 16.5 kcal/mol for ***^sr^*TSb3** (*si*/*re*), ∆*G*^‡^ = 18.8 kcal/mol for ***^ss^*TSb3** (*si*/*si*) (Figure 6 and Figure 7). The observed diastereoselectivity toward the (*6R*,*7S*)-adduct aligns with experimental results and is attributed to the lowest-energy transition state (***^sr^*TSb3**, ∆*G*^‡^ = 16.5 kcal/mol).

Step 4 and Step 5: Enol Tautomerism

Three distinct mechanistic pathways for enol tautomerization were evaluated computationally. First, direct proton transfer from ***^sr^*INTb6** via transition state **TSb4′** was excluded due to its prohibitively high barrier (∆*G*^‡^ = 62.9 kcal/mol relative to ***^sr^*INTb6**). Second, DIPEA·H^+^-assisted proton migration via **TSb4″** also exhibited a high barrier (∆*G*^‡^ = 25.1 kcal/mol relative to ***^sr^*INTb6**). Third, the DIPEA-assisted mechanism proceeds as follows: (i) ***^rs^*INTb6** forms a hydrogen-bonded complex **INTb7** with DIPEA, which is exergonic (∆*G* = −5.5 kcal/mol relative to ***^sr^*INTb6**). (ii) Proton transfer through the hydrogen-bonded transition state **TSb4** affords **INTb8**. This step has a barrier of ∆*G*^‡^ = 10.3 kcal/mol and is endergonic (∆*G*^‡^ = +11.5 kcal/mol), both relative to **INTb7**. (iii) The DIPEA·H^+^ proton in **INTb8** migrates to C5 via **TSb5** (∆*G*^‡^ = 2.0 kcal/mol) to form **INTb9**, with strong exergonicity (∆*G* = −25.2 kcal/mol), both relative to **INTb8**. Consequently, the DIPEA-assisted pathway is energetically favored for enol tautomerization.

Step 6: Aza-Dieckmann-type Cyclization

The intermediate **INTb9** undergoes an aza-Dieckmann-type cyclization via the five-membered-ring transition state **TSb6** to afford **INTb10**. This step has a low activation barrier (∆*G*^‡^ = 12.0 kcal/mol) and is slightly endergonic (∆*G* = +2.1 kcal/mol), both relative to **INTb9**.

Step 7: Regeneration of **NHC^II^**

**INTb10** releases the catalyst **NHC^II^** and forms **INTb11** via transition state **TSb7**. This dissociation step exhibits a low activation barrier (∆*G*^‡^ = 9.4 kcal/mol) and is exergonic (∆*G* = −6.1 kcal/mol), both relative to **INTb10**. These results demonstrate that the regeneration of catalyst **NHC^II^** is both kinetically and thermodynamically favorable.

Step 8: Isomerization via Proton Transfer

Four distinct mechanistic pathways were evaluated for the isomerization of the imine moiety. First, the direct proton migration pathway from **INTb11** via transition state **TSb8′** was excluded due to its prohibitively high activation barrier (∆*G*^‡^ = 45.7 kcal/mol relative to **INTb11**). Second, DIPEA·H^+^-assisted proton migration via **TSb8″** also exhibited a similarly high barrier (∆*G*^‡^ = 42.8 kcal/mol relative to **INTb12″**). Consequently, both the direct and DIPEA·H^+^-assisted proton migration mechanisms are energetically unfavorable. Third, the DIPEA-assisted pathway via transition state **TSb8‴** (∆*G*^‡^ = 29.7 kcal/mol relative to **INTb11**) showed a lower barrier compared to the first two pathways. In contrast, considering that DIPEA·H^+^ is already generated in the second step, the positively charged DIPEA·H^+^ can form an intermolecular hydrogen bond with the ketone carbonyl oxygen in **INTb11**, which facilitates the proton migration at H9. Calculation results revealed that **TSb8** exhibits the lowest barrier among the four pathways (∆*G*^‡^ = 29.1 kcal/mol relative to **INTb11**). Therefore, the pathway co-assisted by DIPEA·H^+^ and DIPEA was considered viable: (i) **INTb11** forms a hydrogen-bonded complex **INTb12** with DIPEA·H^+^ and DIPEA, which is endergonic (∆*G* = +6.9 kcal/mol relative to **INTb11**). (ii) Proton transfer through the hydrogen-bonded transition state **TSb8** affords complex **INTb13**. This step exhibits an activation barrier of ∆*G*^‡^ = 29.1 kcal/mol relative to **INTb11**. (iii) The DIPEA·H^+^ proton in **INTb13** migrates to nitrogen via **TSb9** (∆*G* = 14.4 kcal/mol relative to **INTb13**) to form product **b**, a mildly exergonic process (∆*G* = −2.7 kcal/mol relative to **INTb13**). Therefore, the pathway co-assisted by DIPEA·H^+^ and DIPEA is energetically favored for this isomerization process.

### 2.2. Origins of Regioselectivity

**Figure 8:**
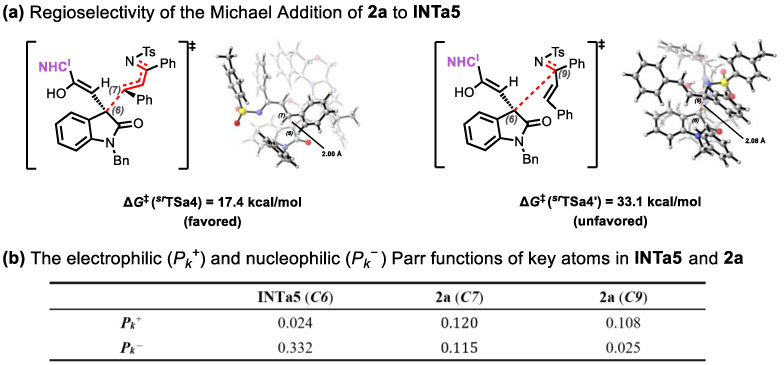
(**a**) Regioselectivity of the Michael Addition of **2a** to **INTa5**; (**b**) The electrophilic (*P_k_*^+^) and nucleophilic (*P_k_*^−^) Parr functions of key atoms in **INTa5** and **2a**.

For the Michael addition of **INTa5** with **2a**, besides the pathway generating the intermediate ***^sr^*INTa7** via ***^sr^*TSa4** described in the previous section, we also identified an alternative pathway that involves C6-C9 bond formation through transition state ***^sr^*TSa4′** (Figure 8a). However, the corresponding activation barrier is 15.7 kcal/mol higher than that of ***^sr^*TSa4** indicating that formation of ***^sr^*INTa7** is energetically favored. This regioselectivity is consistent with Parr function analysis [55], as summarized in (Figure 8b). Specifically, the calculated electrophilic Parr function (*P_k_*^+^) value for the C7 atom (0.120) is higher than that for the C9 atom (0.108). Consequently, nucleophilic attack occurs preferentially at the C7 atom during the addition of **2a** to **INTa5**, in agreement with our DFT calculations.

**Figure 9:**
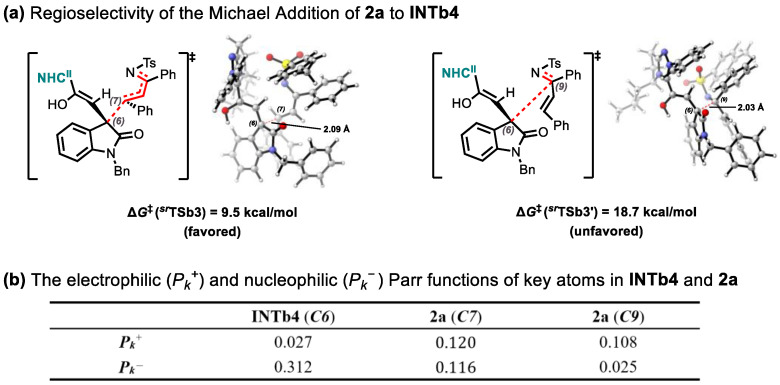
(**a**) Regioselectivity of the Michael Addition of **2a** to **INTb4**; (**b**) The electrophilic (*P_k_*^+^) and nucleophilic (*P_k_*^−^) Parr functions of key atoms in **INTb4** and **2a**.

Regarding the Michael addition between **INTb4** and **2b**, in addition to the previously described pathway forming intermediate ***^sr^*INTb6** via ***^sr^*TSb3**, computational exploration revealed an alternative route. This pathway proceeds through transition state ***^sr^*TSb3′** with concomitant C6-C9 bond formation (Figure 9a). Notably, the activation barrier for this alternative route surpasses that of ***^sr^*TSb3** by 9.2 kcal/mol, establishing ***^sr^*INTb6** formation as the energetically preferred process. The observed regioselectivity is rationalized through Parr function analysis [55] (Figure 9b). For electrophile **2a**, computed electrophilic Parr function (*P_k_*^+^) values at C7 (0.120) and C9 (0.108) reveal enhanced electrophilicity at C7. This electronic preference facilitates nucleophilic attack by **INTb4**’s C6 atom at the C7 position of **2a**, in full accord with DFT computational outcomes.

### 2.3. Origins of Stereoselectivity

The observed stereoselectivity in the formation of product **a** originates from the Michael addition step, which establishes the stereogenic centers at C6 and C7. As detailed in Step **3**, the computed energy barriers for the diastereomeric addition pathways are: 17.4 kcal/mol (***^sr^*TSa4**(6*S*,7*R*)), 24.6 kcal/mol (***^ss^*TSa4**(6*S*,7*S*)), 23.6 kcal/mol (***^rr^*TSa4**(6*R*,7*R*)), and 27.1 kcal/mol (***^rs^*TSa4**(6*R*,7*S*)), with the *si*/re pathway being energetically most favorable. The energy difference between ***^sr^*TSa4** and ***^rs^*TSa4** (Δ∆*G*^‡^ = 9.7 kcal/mol) corresponds to a predicted enantiomeric excess of 99.9%, in excellent agreement with the experimental value (98% ee).

**Figure 10:**
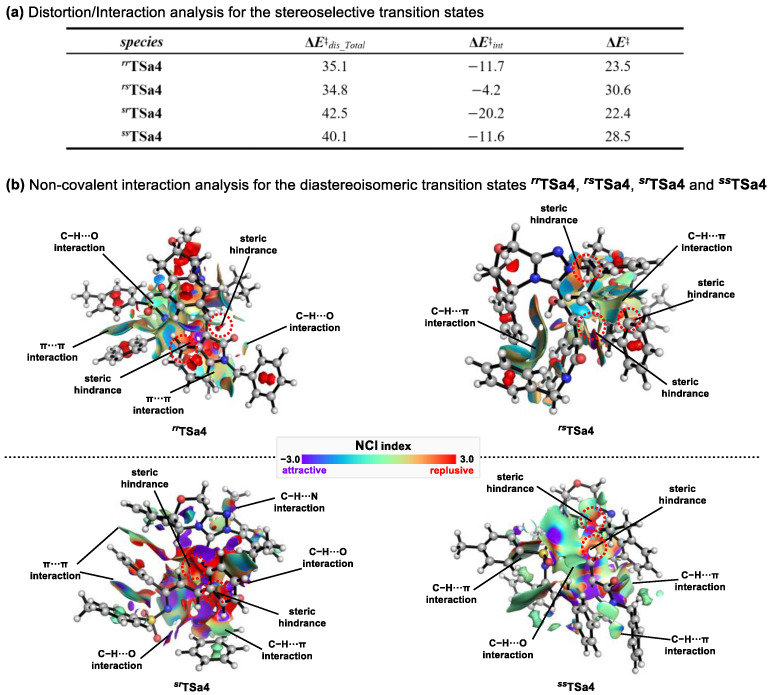
(**a**) Distortion/interaction analysis for the stereoselective transition states; (**b**) Interaction analysis for the diastereoisomeric transition states ***^rr^*TSa4**, ***^rs^*TSa4**, ***^sr^*TSa4** and ***^ss^*TSa4**.

To further elucidate the origin of the stereoselectivity, a distortion-interaction analysis [56] was carried out (Figure 10a). The computational results reveal that the activation energies (**Δ*E*^‡^**) are consistent with the Gibbs free energy barriers (**Δ*G*^‡^**) derived from the reaction mechanism. Although the total distortion energies (**Δ*E*^‡^***_dis_Total_*) of ***^sr^*TSa4** (42.5 kcal/mol) and ***^ss^*TSa4** (40.1 kcal/mol) are higher than those of ***^rr^*TSa4** (35.1 kcal/mol) and ***^rs^*TSa4** (34.8 kcal/mol), ***^sr^*TSa4**, which exhibits the lowest activation energy, features the most favorable interaction energy (−20.2 kcal/mol). This plays a decisive role in lowering the reaction barrier. Overall, the interaction energy governs the energy variations in this reaction.

Further non-covalent interaction (NCI) analysis (Figure 10b) revealed that ***^sr^*TSa4** exhibits the most extensive attractive regions (shown in purple), including two *π*⋯*π* interactions (3.021 and 3.14 Å), two C-H⋯O interactions (2.863 and 2.857 Å), one C-H⋯*π* interaction (2.681 Å), and one C-H⋯N interaction (2.636 Å). This was followed by ***^ss^*TSa4**, which contains three C-H⋯*π* interactions (3.023, 2.852, and 2.764 Å) and one C-H⋯O interaction (2.320 Å), and ***^rr^*TSa4**, which exhibits two *π*⋯*π* interactions (2.476 and 2.566 Å) together with two C-H⋯O interactions (3.031 and 2.944 Å). In contrast, ***^rs^*TSa4** only shows two C-H⋯*π* interactions (2.835 and 2.981 Å). Additionally, repulsive non-covalent interactions in all four transition states are mainly localized in the region between the Breslow intermediate **INTa5** and substrate **2a** (red areas). These results clearly demonstrate that, among the four transition states, ***^sr^*TSa4** (6*S*,7*R*) features the strongest attractive interactions, which likely accounts for the stereoselectivity observed experimentally.

Stereocontrol in product **b** formation is dictated by the Michael addition step, which generates the C6/C7 stereogenic centers. Computational analysis (Step 3) identifies the si/re pathway as kinetically preferred, exhibiting the lowest energy barrier among diastereomeric transition states: 18.2 kcal/mol (***^rr^*TSb3**(6*R*,7*R*)), 18.1 kcal/mol (***^rs^*TSb3**(6*R*,7*S*)), 16.5 kcal/mol (***^sr^*TSb3**(*6S*,7*R*)), 18.8 kcal/mol (***^ss^*TSb3**(6*S*,7*S*)). The Δ∆*G*^‡^ = 1.6 kcal/mol between the dominant (***^sr^*TSb3**) and major competing (***^rs^*TSb3**) transition states corresponds to 87.4% predicted ee, closely matching the experimental 88% ee.

**Figure 11:**
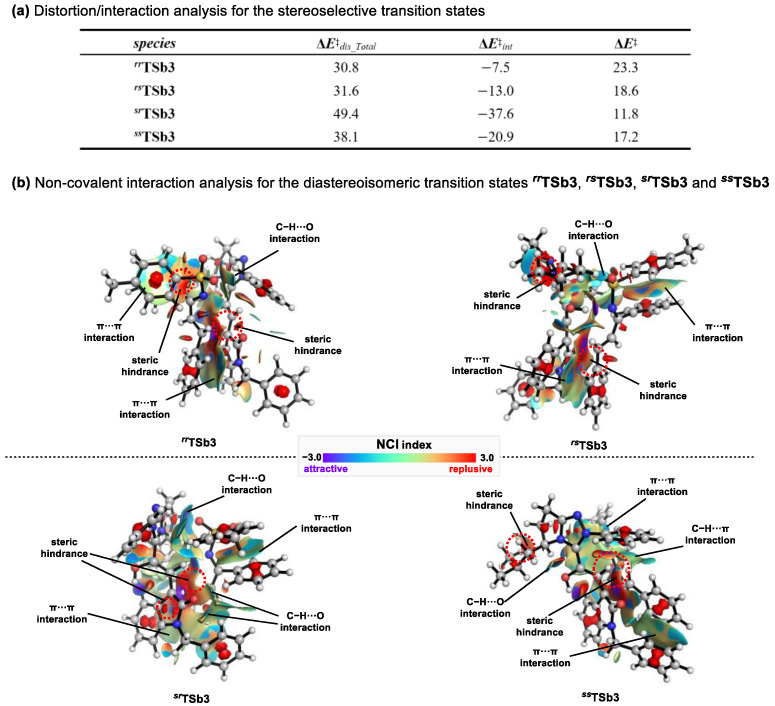
(**a**) Distortion/interaction analysis for the stereoselective transition states; (**b**) Interaction analysis for the diastereoisomeric transition states ***^rr^*TSb3**, ***^rs^*TSb3**, ***^sr^*TSb3** and ***^ss^*TSb3**.

Concerning the origin of stereoselectivity for product **b**, we also performed a distortion-interaction analysis [56] (Figure 11a). The computational findings demonstrate that the activation energies (**Δ*E*^‡^**) are consistent with the Gibbs free energy barriers (**Δ*G*^‡^**) derived from the reaction mechanism. Even though the total distortion energy (**Δ*E*^‡^***_dis_Total_*) of ***^sr^*TSb3** (49.4 kcal/mol) exceeds those of ***^ss^*TSb3** (38.1 kcal/mol), ***^rr^*TSb3** (30.8 kcal/mol), and ***^rs^*TSb3** (31.6 kcal/mol), ***^sr^*TSb3**—possessing the lowest activation energy—displays the most favorable interaction energy (−37.6 kcal/mol), which exerts a decisive effect on lowering the reaction barrier. Overall, the interaction energy controls the energy variations in this reaction.

A further non-covalent interaction (NCI) analysis (Figure 11b) revealed that ***^sr^*TSb3** exhibits the most extensive attractive regions (shown in purple), including two *π*⋯*π* interactions (2.946 and 3.046 Å) and three C-H⋯O interactions (2.334, 2.307, and 2.843 Å). This was followed by ***^ss^*TSb3** and ***^rr^*TSb3**: among them, ***^ss^*TSb3** contains two *π⋯π* interactions (3.159 and 3.254 Å), one C-H⋯*π* interaction (2.669 Å), and one C-H⋯O interaction (2.600 Å); in contrast, ***^rr^*TSb3** exhibits two *π*⋯*π* interactions (3.008 and 2.943 Å) and one C-H⋯O interaction (2.597 Å). Meanwhile, ***^rs^*TSb3**—with the weakest attractive interactions—shows two *π*⋯*π* interactions (3.109 and 3.004 Å) and one C-H⋯O interaction (2.301 Å). Additionally, the repulsive non-covalent interactions in all four transition states are mainly localized in the region between the Breslow intermediate **INTb4** and substrate **2a** (red areas). These results clearly demonstrate that among the four transition states, ***^sr^*TSb3** (6*S*,7*R*) possesses the strongest attractive interactions, a feature that likely accounts for the stereoselectivity observed experimentally.

### 2.4. The Role of Catalysts

**Figure 12:**
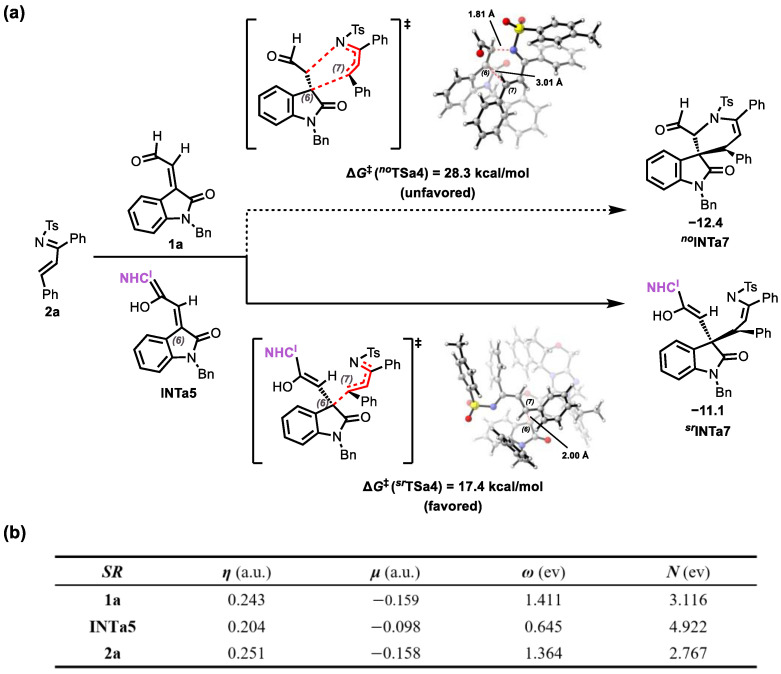
(**a**) Catalytic Effect of **NHC^I^** on Michael Addition; (**b**) Electronic chemical potential (*μ*, in a.u.), chemical hardness (*η*, in a.u.), global electrophilicity (*ω*, in eV) and global nucleophilicity (*N*, in eV) of some reactants (6*S*,7*R*) involved in the key steps.

Figure 12a reveals that the uncatalyzed Michael addition between **2a** and **1a** exhibits an activation barrier of 28.3 kcal/mol, significantly higher than the NHC^I^-catalyzed barrier (17.4 kcal/mol). This 10.9 kcal/mol reduction demonstrates the critical catalytic role of **NHC^I^**.

To elucidate the catalytic role of **NHC^I^** in the reaction of isatin-derived enals with N-sulfonyl ketoimines, global reactivity indices (GRIs) [57,58,59,60,61] analysis was employed. Figure 12b demonstrates that **NHC^I^** activation enhances the nucleophilicity of **1a** from 3.116 eV to 4.922 eV in **INTa5**, consequently reducing the Michael addition activation barrier with electrophile **2a**.

**Figure 13:**
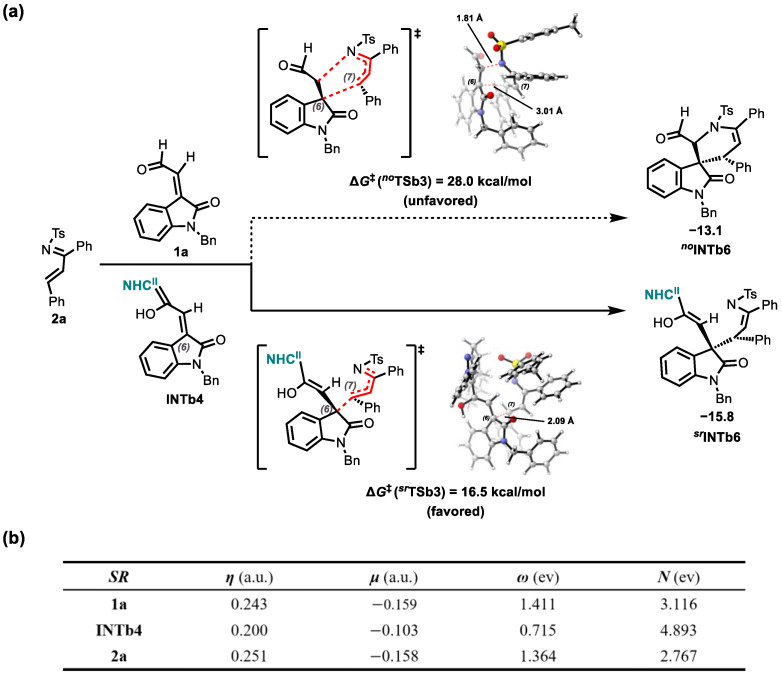
(**a**) Catalytic Effect of **NHC^II^** on Michael Addition; (**b**) Electronic chemical potential (*μ*, in a.u.), chemical hardness (*η*, in a.u.), global electrophilicity (*ω*, in eV) and global nucleophilicity (*N*, in eV) of some reactants (6*S*,7*R*) involved in the key steps.

As shown in Figure 13a, the activation barrier for the uncatalyzed Michael addition between **2a** and **1a** (28.0 kcal/mol) is significantly higher (by 9.1 kcal/mol) than that for the NHC^II^-catalyzed reaction (18.1 kcal/mol), highlighting the critical catalytic function of **NHC^II^**. Analysis of global reactivity indices (GRIs) [57,58,59,60,61] revealed that **NHC^II^** activation enhanced the nucleophilicity of **1a** to 4.893 eV for **INTb4**, a significant increase from 3.116 eV. This enhancement substantially lowered the activation barrier for the Michael addition reaction with electrophile **2a**.

## 3. Computational Methods

All DFT calculations were performed with the Gaussian 09 (Revision E.01) software package [62]. The M06-2X [63]-D3 [64] functional was used for the geometry optimization in the gas phase at 298.15 K and 1 atm with the 6-31G(d,p) basis set [65,66,67] for all elements. Harmonic vibrational frequency calculations were performed for all of the stationary points to determine whether they are local minima or transition structures and to derive the thermochemical corrections for the enthalpies and free energies. The same functional and more accurate 6-311++G(2df,2pd) basis set [68] was used to calculate the single-point energies for the [3+4] annulation in acetonitrile (MeCN, *ε* = 35.688) and the [3+2] annulation in dichloroethane (DCE, *ε* = 10.125) from the gas-phase stationary points with the IEFPCM model [69]. The discussed energies are Gibbs free energies (∆G_298_, kcal/mol). The connectivity of all transition states has been verified by the intrinsic reaction coordinate (IRC) analysis [70,71,72]. NPA [73] charges were computed with the NBO program implemented in Gaussian. Non-covalent interaction analysis [74] was performed using NCIPLOT (version 3.0) [75] and Pymol (version 3.0.0) [76]. All 3D structures were generated using CYLview (version 2.0) [77].

## 4. Conclusions

In our study, DFT calculations were performed to elucidate the reaction pathways and the origins of regio- and stereoselectivity in the switchable formation of spirocyclopentane oxindoles from isatin-derived enals and N-sulfonyl ketimines, catalyzed by NHCs. DFT calculations elucidate the catalytic cycle for product **a** formation, comprising nucleophilic addition, 1,2-proton transfer to afford the Breslow intermediate, Michael addition, (1,3)-proton shift, Mannich-type reaction, cyclization, and catalyst regeneration. The formation of product **b** involves a catalytic cycle comprising nucleophilic addition, Breslow intermediate formation, Michael addition, enol-keto tautomerism, proton transfer, aza-Dieckmann-cyclization, catalyst regeneration, and tautomerization. In pathway a, the K_3_PO_4_, and in pathway b, the DIPEA, effectively facilitate the formation of the Breslow intermediate and proton transfer. The Michael addition step is the decisive step governing the regio- and stereoselectivity. The activation energies for the Michael addition of **INTa5** or **INTb4** to **2a** reveal that C6-C7 bond formation is consistently favored. Parr function analysis rationalizes that the regioselectivity arises from the greater electrophilicity of C7 than C9 in **2a**. Distortion/interaction analysis demonstrates that the stereoselectivity of the Michael addition is governed by both the distortion energy and non-covalent interaction energy. Global reactivity indices show that the catalyst facilitates enhanced electrophilicity of electrophile **2a** and reduced activation barriers for the Michael addition reaction.

## Data Availability

The data are contained within the Supporting Information (SI).

## Supplementary Materials

The following supporting information can be downloaded at: https://www.mdpi.com/article/10.3390/molecules30214218/s1, Figure S1: (**a**) Precatalyst **Pre-NHC^I^** forms active catalyst **NHC^I^**; (**b**) IRC diagram for the formation of the active catalyst **NHC^I^**; Figure S2: Precatalyst **Pre-NHC^II^** forms active catalyst **NHC^II^**. Cartesian coordinates and total energies for all optimized geometries are tabulated in the Supporting Information (PDF).

## References

[1] Chaudhari P.D., Hong B.C., Wen C.L., Lee G.H. (2019). Asymmetric Synthesis of Spirocyclopentane Oxindoles Containing Four Consecutive Stereocenters and Quaternary α-Nitro Esters via Organocatalytic Enantioselective Michael–Michael Cascade Reactions. ACS Omega.

[2] Zeng W., Han C., Mohammed S., Li S.S., Song Y.X., Sun F.X., Du Y.F. (2024). Indole-containing pharmaceuticals: Targets, pharmacological activities, and SAR studies. RSC Med. Chem..

[3] Luo Q.L., Mao T., Luo Y., Zhang T.X., Wang F., Dong S.X., Feng X.M. (2024). Enantioselective Synthesis of Spiro [cyclopentane-1,3’-oxindole] Scaffolds with Five Consecutive Stereocenters. Org. Lett..

[4] Boddy A.J., Bull J.A. (2021). Stereoselective synthesis and applications of spirocyclic oxindoles. Org. Chem. Front..

[5] Zhang J.Q., Li N.K., Yin S.J., Sun B.B., Fan W.T., Wang X.W. (2017). Chiral N-Heterocyclic Carbene-Catalyzed Asymmetric Michael-Intramolecular Aldol-Lactonization Cascade for Enantioselective Construction of *β*-Propiolactone-Fused Spiro [cyclopentane-oxindoles]. Adv. Synth. Catal..

[6] Dočekal V., Vopálenská A., Měrka P., Konečná K., Jand’ourek O., Pour M., Císařová I., Veselý J. (2021). Enantioselective Construction of Spirooxindole-Fused Cyclopentanes. J. Org. Chem..

[7] Ren J., Ding S.H., Li X.N., Zhao Q.S. (2024). Unified strategy enables the collective syntheses of structurally diverse indole alkaloids. J. Am. Chem. Soc..

[8] Chowdhury S., Chafeev M., Liu S.F., Sun J.Y., Raina V., Chui R., Young W., Kwan R., Fu J.M., Cadieux J.A. (2011). Discovery of XEN907, a spirooxindole blocker of NaV1.7 for the treatment of pain. Bioorg. Med. Chem. Lett..

[9] Chowdhury S., Liu S.F., Cadieux J.A., Hsieh T., Chafeev M., Sun S.Y., Jia Q., Sun J.Y., Wood M., Langille J. (2013). Tetracyclic spirooxindole blockers of hNa V 1.7: Activity in vitro and in CFA-induced inflammatory pain model. Med. Chem. Res..

[10] Gu J., Peng R.K., Guo C.L., Zhang M., Yang J., Yan X., Zhou Q., Li H.W., Wang N., Zhu J.W. (2022). Construction of a synthetic methodology-based library and its application in identifying a GIT/PIX protein–protein interaction inhibitor. Nat. Commun..

[11] Sakhuja R., Panda S.S., Khanna L., Khurana S., Jain S.C. (2011). Design and synthesis of spiro [indole-thiazolidine] spiro [indole-pyrans] as antimicrobial agents. Bioorg. Med. Chem. Lett..

[12] Bhaskar G., Arun Y., Balachandran C., Saikumar C., Perumal P.T. (2012). Synthesis of novel spirooxindole derivatives by one pot multicomponent reaction and their antimicrobial activity. Eur. J. Med. Chem..

[13] Jiang K., Tiwari B., Chi Y.R. (2012). Access to spirocyclic oxindoles via N-heterocyclic carbene-catalyzed reactions of enals and oxindole-derived α,*β*-unsaturated imines. Org. Lett..

[14] Jin J.M., Lv Y., Tang W.L., Teng K.P., Huang Y.X., Ding J.X., Li T.T., Wang G.J., Chi Y.R. (2024). Enantioselective Transformation of Hydrazones via Remote NHC Catalysis: Activation Across C=N and N–N Bonds. ACS Catal..

[15] Lv H., Tiwari B., Mo J.M., Xing C., Chi Y.R. (2012). Highly Enantioselective Addition of Enals to Isatin-Derived Ketimines Catalyzed by N-Heterocyclic Carbenes: Synthesis of Spirocyclic *γ*-Lactams. Org. Lett..

[16] Xu J.F., Mou C.L., Zhu T.S., Song B.A., Chi Y.R. (2014). N-Heterocyclic Carbene-Catalyzed Chemoselective Cross-Aza-Benzoin Reaction of Enals with Isatin-Derived Ketimines: Access to Chiral Quaternary Aminooxindoles. Org. Lett..

[17] Bera S., Daniliuc C.G., Studer A. (2017). Oxidative N-heterocyclic carbene catalyzed dearomatization of indoles to spirocyclic indolenines with a quaternary carbon stereocenter. Angew. Chem. Int. Ed..

[18] Yao C.S., Xiao Z.X., Liu R., Li T.J., Jiao W.H., Yu C.X. (2013). N-Heterocyclic-Carbene-Catalyzed Reaction of *α*-Bromo-*α*,*β*-Unsaturated Aldehyde or *α*,*β*-Dibromoaldehyde with Isatins: An Efficient Synthesis of Spirocyclic Oxindole-Dihydropyranones. Chem. Eur. J..

[19] Liu R., Yu C.X., Xiao Z.X., Li T.J., Wang X.S., Xie Y.W., Yao C.S. (2014). NHC-catalyzed oxidative *γ*-addition of *α*,*β*-unsaturated aldehydes to isatins: A high-efficiency synthesis of spirocyclic oxindole-dihydropyranones. Org. Biomol. Chem..

[20] Zhu L., Yu C.X., Li T.J., Wang Y.H., Lu Y.N., Wang W.J., Yao C.S. (2016). N-Heterocyclic carbene-catalyzed [4+2] cyclization of *α*,*β*-unsaturated carboxylic acids bearing *γ*-H with isatins: An enantioselective synthesis of spirocyclic oxindole–dihydropyranones. Org. Biomol. Chem..

[21] Liu B.H., Duan X.Y., Li J.H., Wu Y.T., Li Y.T., Qi J. (2022). N-heterocyclic carbene-catalyzed [3+2] annulation of 3,3′-bisoxindoles with α-bromoenals: Enantioselective construction of contiguous quaternary stereocenters. Org. Lett..

[22] Breuers C.B.J., Daniliuc C.G., Studer A. (2022). Dearomatizing Cyclization of 2-Iodoindoles by Oxidative NHC Catalysis to Access Spirocyclic Indolenines and Oxindoles Bearing an All Carbon Quaternary Stereocenter. Org. Lett..

[23] Liang Z., Li J.B., Liu C.L., Zhu Y.W., Du D. (2023). N-heterocyclic carbene-catalyzed enantioselective synthesis of spirocyclic ketones bearing gem-difluoromethylenes. Org. Chem. Front..

[24] Maji U., Das S., Baidya A., Roy I., Guin J. (2024). Asymmetric Synthesis of Benzofuranones with a C3 Quaternary Center via an Addition/Cyclization Cascade Using Noncovalent N-Heterocyclic Carbene Catalysis. Org. Lett..

[25] Tang C.H., Wang W., Luo G.Y., Song C.Y., Bao Z.W., Li P., Hao G.F., Chi Y.G.R., Jin Z.C. (2022). Carbene-Catalyzed Activation of C−Si Bonds for Chemo- and Enantioselective Cross Brook–Benzoin Reaction. Angew. Chem..

[26] Reimler J., Yu X.Y., Spreckelmeyer N., Daniliuc C.G., Studer A. (2023). Regiodivergent C-H Acylation of Arenes by Switching from Ionic-to Radical-Type Chemistry Using NHC Catalysis. Angew. Chem. Int. Ed..

[27] Ryan S.J., Candish L., Lupton D.W. (2009). N-heterocyclic carbene-catalyzed generation of *α*,*β*-unsaturated acyl imidazoliums: Synthesis of dihydropyranones by their reaction with enolates. J. Am. Chem. Soc..

[28] Kravina A.G., Mahatthananchai J., Bode J.W. (2012). Enantioselective NHC-Catalyzed Annulations of Trisubstituted Enals and Cyclic N-Sulfonylimines via *α*,*β*-Unsaturated Acyl Azoliums. Angew. Chem. Int. Ed..

[29] Yetra S.R., Kaicharla T., Kunte S.S., Gonnade R.G., Biju A.T. (2013). Asymmetric N-heterocyclic carbene (NHC)-catalyzed annulation of modified enals with enolizable aldehydes. Org. Lett..

[30] Gao Z.H., Chen X.Y., Zhang H.M., Ye S. (2015). N-Heterocyclic carbene-catalyzed [3+3] cyclocondensation of bromoenals with aldimines: Highly enantioselective synthesis of dihydropyridinones. Chem. Commun..

[31] Yi L., Chen K.Q., Liang Z.Q., Sun D.Q., Ye S. (2017). N-Heterocyclic carbene-catalyzed [3+3] annulation of indoline-2-thiones with bromoenals: Synthesis of indolo [2,3-b] dihydrothiopyranones. Adv. Synth. Catal..

[32] Zhao C.G., Blaszczyk S.A., Wang J.M. (2021). Asymmetric reactions of N-heterocyclic carbene (NHC)-based chiral acyl azoliums and azolium enolates. Green. Synth. Catal..

[33] He M., Struble J.R., Bode J.W. (2006). Highly enantioselective azadiene Diels-Alder reactions catalyzed by chiral N-heterocyclic carbenes. J. Am. Chem. Soc..

[34] He L., Lv H., Zhang Y.R., Ye S. (2008). Formal cycloaddition of disubstituted ketenes with 2-oxoaldehydes catalyzed by chiral N-heterocyclic carbenes. J. Org. Chem..

[35] Wang X.N., Shao P.L., Lv H., Ye S. (2009). Enantioselective Synthesis of *β*-Trifluoromethyl-*β*-lactones via NHC-Catalyzed Ketene-Ketone Cycloaddition Reactions. Org. Lett..

[36] Wang X.N., Zhang Y.Y., Ye S. (2010). Enantioselective Synthesis of Spirocyclic Oxindole-*β*-lactones via N-Heterocyclic Carbene-Catalyzed Cycloaddition of Ketenes and Isatins. Adv. Synth. Catal..

[37] Wang X.N., Shen L.T., Ye S. (2011). NHC-catalyzed enantioselective [2+2] and [2+2+2] cycloadditions of ketenes with isothiocyanates. Org. Lett..

[38] Wang L., Ni Q.J., Blümel M., Shu T., Raabe G., Enders D. (2015). NHC-Catalyzed Asymmetric Synthesis of Functionalized Succinimides from Enals and α-Ketoamides. Chem. Eur. J..

[39] Burstein C., Glorius F. (2004). Organocatalyzed conjugate umpolung of *α*,*β*-unsaturated aldehydes for the synthesis of *γ*-butyrolactones. Angew. Chem. Int. Ed..

[40] Matsuoka Y., Ishida Y., Sasaki D., Saigo K. (2008). Cyclophane-type imidazolium salts with planar chirality as a new class of N-heterocyclic carbene precursors. Chem. Eur. J..

[41] Li Y., Wang X.Q., Zheng C., You S.L. (2009). Highly enantioselective intramolecular Michael reactions by D-camphor-derived triazolium salts. Chem. Commun..

[42] Cohen D.T., Eichman C.C., Phillips E.M., Zarefsky E.R., Scheidt K.A. (2012). Catalytic dynamic kinetic resolutions with N-heterocyclic carbenes: Asymmetric synthesis of highly substituted *β*-lactones. Angew. Chem. Int. Ed..

[43] Guo C., Schedler M., Daniliuc C.G., Glorius F. (2014). N-Heterocyclic Carbene Catalyzed Formal [3+2] Annulation Reaction of Enals: An Efficient Enantioselective Access to Spiro-Heterocycles. Angew. Chem. Int. Ed..

[44] Guo C., Sahoo B., Daniliuc C.G., Glorius F. (2014). N-heterocyclic carbene catalyzed switchable reactions of enals with azoalkenes: Formal [4+3] and [4+1] annulations for the synthesis of 1,2-diazepines and pyrazoles. J. Am. Chem. Soc..

[45] Chen X.Y., Xong J.W., Liu Q., Li S., Sheng H., von Essen C., Rissanen K., Enders D. (2018). Control of N-Heterocyclic Carbene Catalyzed Reactions of Enals: Asymmetric Synthesis of Oxindole-γ-Amino Acid Derivatives. Angew. Chem. Int. Ed..

[46] Liu L.X., Zhou C.Y., Wang C.M. (2023). Construction of highly congested quaternary carbon centers by NHC catalysis through dearomatization. Green. Synth. Catal..

[47] Liu C.H., Han P.L., Zhang X.S., Qiao Y., Xu Z.H., Zhang Y.G., Li D.P., Wei D.H., Lan Y. (2022). NHC-Catalyzed transformation reactions of imines: Electrophilic versus nucleophilic attack. J. Org. Chem..

[48] Nong Y.L., Pang C., Teng K.P., Zhang S., Liu Q. (2023). NHC-Catalyzed Chemoselective Reactions of Enals and Cyclopropylcarbaldehydes for Access to Chiral Dihydropyranone Derivatives. J. Org. Chem..

[49] Hou X.X., Wei D.H. (2024). Mechanism and origin of stereoselectivity for the NHC-catalyzed desymmetrization reaction for the synthesis of axially chiral biaryl aldehydes. J. Org. Chem..

[50] Ding Y.M., Long X.W., Zhang J.W., Qu C.L., Wang P., Yang X.D., Puno P.T., Deng J. (2025). Asymmetric total synthesis of penicilfuranone A through an NHC-catalyzed umpolung strategy. Chem. Sci..

[51] Wang L., Li S., Blümel M., Puttreddy R., Peuronen A., Rissanen K., Enders D. (2017). Switchable Access to Different Spirocyclopentane Oxindoles by N-Heterocyclic Carbene Catalyzed Reactions of Isatin-Derived Enals and N-Sulfonyl Ketimines. Angew. Chem. Int. Ed..

[52] Wang Y.Y., Wei D.H., Wang Y., Zhang W.J., Tang M.S. (2016). N-heterocyclic carbene (NHC)-catalyzed sp^3^ *β*-C-H activation of saturated carbonyl compounds: Mechanism, role of NHC, and origin of stereoselectivity. ACS Catal..

[53] He N., Zhu Y.Y., Zhu Z.H., Yang Y.K., Zhang W.J., Wei D.H., Qu L.B., Tang M.S., Chen H.S. (2019). A density functional theory study on mechanisms of [4+2] annulation of enal with α-methylene cycloalkanone catalyzed by N-heterocyclic carbene. Int. J. Quantum Chem..

[54] Cao S.S., Yuan H.Y., Zhang J.P. (2019). A mechanistic investigation into N-heterocyclic carbene (NHC) catalyzed umpolung of ketones and benzonitriles: Is the cyano group better than the classical carbonyl group for the addition of NHC?. Org. Chem. Front..

[55] Domingo L.R., Pérez P., Sáez J.A. (2013). Understanding the local reactivity in polar organic reactions through electrophilic and nucleophilic Parr functions. RSC Adv..

[56] Bickelhaupt F.M., Houk K.N. (2017). Analyzing Reaction Rates with the Distortion/Interaction-Activation Strain Model. Angew. Chem. Int. Ed..

[57] Parr R.G., Pearson R.G. (1983). Absolute hardness: Companion parameter to absolute electronegativity. J. Am. Chem. Soc..

[58] Yepes D., Murray J.S., Pérez P., Domingo L.R., Politzer P., Jaque P. (2014). Complementarity of reaction force and electron localization function analyses of asynchronicity in bond formation in Diels-Alder reactions. Phys. Chem. Chem. Phys..

[59] Sham L.J., Kohn W. (1966). One-particle properties of an inhomogeneous interacting electron gas. Phys. Rev..

[60] Domingo L.R., Picher M.T., Sáez J.A. (2009). Toward an understanding of the unexpected regioselective hetero-diels-alder reactions of asymmetric tetrazines with electron-rich ethylenes: A DFT study. J. Org. Chem..

[61] Domingo L.R., Saézm J.A., Zaragozá R.J., Arnó M. (2008). Understanding the participation of quadricyclane as nucleophile in polar [2*σ*+2*σ*+2*π*] cycloadditions toward electrophilic *π* molecules. J. Org. Chem..

[62] Frisch M.J., Trucks G.W., Schlegel H.B., Scuseria G.E., Robb M.A., Cheeseman J.R., Scalmani G., Barone V., Mennucci B., Petersson G.A. (2013). Gaussian 09, Revision E.01.

[63] Zhao Y., Truhlar D.G. (2008). The M06 suite of density functionals for main group thermochemistry, thermochemical kinetics, noncovalent interactions, excited states, and transition elements: Two new functionals and systematic testing of four M06-class functionals and 12 other functionals. Theor. Chem. Acc..

[64] Schröder H., Hühnert J., Schwabe T. (2017). Evaluation of DFT-D3 dispersion corrections for various structural benchmark sets. J. Chem. Phys..

[65] Hariharan P.C., Pople J.A. (1973). The influence of polarization functions on molecular orbital hydrogenation energies. Theoret. Chim. Acta.

[66] Francl M.M., Pietro W.J., Hehre W.J., Binkley J.S., Gordon M.S., DeFrees D.J., Pople J.A. (1982). Self-consistent molecular orbital methods. XXIII. A polarization-type basis set for second-row elements. J. Chem. Phys..

[67] Rassolov V.A., Pople J.A., Ratner M.A., Windus T.L. (1998). 6-31G* basis set for atoms K through Zn. J. Chem. Phys..

[68] Zhao Y., Truhlar D.G. (2008). Density functionals with broad applicability in chemistry. Acc. Chem. Res..

[69] Bonaccorsi R., Cimiraglia R., Tomasi J. (1983). Ab initio evaluation of absorption and emission transitions for molecular solutes, including separate consideration of orientational and inductive solvent effects. J. Comput. Chem..

[70] Fukui K. (1981). The path of chemical reactions-the IRC approach. Acc. Chem. Res..

[71] Hratchian H.P., Schlegel H.B. (2004). Accurate reaction paths using a Hessian based predictor–corrector integrator. J. Chem. Phys..

[72] Hratchian H.P., Schlegel H.B. (2005). Using Hessian updating to increase the efficiency of a Hessian based predictor-corrector reaction path following method. J. Chem. Theory Comput..

[73] Reed A.E., Curtiss L.A., Weinhold F. (1988). Intermolecular interactions from a natural bond orbital, donor-acceptor viewpoint. Chem. Rev..

[74] Johnson E.R., Keinan S., Mori-Sánchez P., Contreras-García J., Cohen A.J., Yang W. (2010). Revealing Noncovalent Interactions. J. Am. Chem. Soc..

[75] Contreras-García J., Johnson E.R., Keinan S., Chaudret R., Piquemal J.P., Beratan D.N., Yang W.T. (2011). NCIPLOT: A program for plotting noncovalent interaction regions. J. Chem. Theory Comput..

[76] DeLano W., Schrödinger, LLC. (2020). The PyMOLmolecular Graphics System.

[77] Legault C.Y. (2020). CYLview20.

