# Supplementary material for: DFT Insights into NHC-Catalyzed Switchable [3+4] and [3+2] Annulations of Isatin-Derived Enals and N-Sulfonyl Ketimines: Mechanism, Regio- and Stereoselectivity
Source: Molecules. 2025 Oct 29;30(21):4218. doi: 10.3390/molecules30214218 (PMC12609633; doi:10.3390/molecules30214218)
Supplement: Supplementary file 1 [file molecules-30-04218-s001.zip › molecules-3933938-supplementary.pdf]

SUPPORTING INFORMATION

DFT Insights into NHC-Catalyzed Switchable [3+4] and [3+2] Annulations of Isatin-Derived Enals and N-Sulfonyl Ketimines: Mechanism, Regio- and Stereoselectivity

Saisai Yu [1,†], Wenxin Zhou [1,†], Yueming Jiang [1], Hangyu Wang [1,*], Xiaoyu Zhou [2,*] and Shengwen Yang [1,3,*]

1 Key Laboratory of Xinjiang Endemic Phytomedicine Resources, Ministry of Education, School of Pharmacy, Shihezi University, Shihezi 832002, China; 20232115021@stu.shzu.edu.cn (S.Y.); 20222015040@stu.shzu.edu.cn (W.Z.); jiangym@shzu.edu.cn (Y.J.)
2 School of Pharmaceutical and Chemical Engineering, Taizhou University, Taizhou 317700, China
3 Yunnan Key Laboratory of Chiral Functional Substance Research and Application, Yunnan Minzu University, Kunming 650504, China
* Correspondence: 18909932852@189.cn (H.W.); zhouxiaoyu@tzc.edu.cn (X.Z.); shengwenyang@shzu.edu.cn (S.Y.)
† These authors contributed equally to this work.

Table of Contents **Page**

1. Computational Details S1

2. Formation of active catalysts **NHC[I]** and **NHC[II]** S2

3. Energies and Cartesian coordinates for all the intermediates and transition states S4-S189

1. Computational Details

(1) Calculation of enantiomeric excess (ee)

The extent of enantioselectivity, in terms of enantiomeric excess (ee%), was calculated by using the Boltzmann distribution of diastereomeric transition states with the following equations:

$$\frac{[R]}{[S]} = \frac{\exp(-\Delta G^{\ddagger}_{[R]}/RT)}{\exp(-\Delta G^{\ddagger}_{[S]}/RT)} = \exp(\Delta\Delta G^{\ddagger}/RT) \qquad \text{(S1)}$$

$$ee\% = \frac{[R]-[S]}{[R]+[S]} \times 100\% = \frac{\frac{[R]}{[S]}-1}{\frac{[R]}{[S]}+1} \times 100\% \qquad \text{(S2)}$$

Where $\Delta \boldsymbol{G}^{\ddagger}$ is the Gibbs free energy barrier of the competing diastereomeric transition state and $\Delta\Delta \boldsymbol{G}^{\ddagger}$ is the Gibbs free energy difference between the two diastereomeric transition states.

(2) Distortion-interaction model

Distortion/interaction analysis provides a fundamental framework for understanding energy barriers in chemical reactions by partitioning the activation energy ($\Delta E^{\ddagger}$) into two physically meaningful components: the distortion energy ($\Delta E^{\ddagger}_{dis}$) and the interaction energy ($\Delta E^{\ddagger}_{int}$). The distortion term quantifies the energy penalty associated with deforming the reactants from their equilibrium geometries to the configurations they adopt in the transition state. The interaction energy, calculated as $\Delta E^{\ddagger}_{int} = \Delta E^{\ddagger} - \Delta E^{\ddagger}_{dis}$, reflects the stabilizing or destabilizing electronic interactions between the deformed reactants within the transition state complex. This approach offers valuable insights into the factors controlling reaction kinetics.

(3) Global reactivity indexes (GRI) analysis

In global reactivity index (GRI) analysis, the electrophilicity index ω which reflects the molecular global electrophilicity character can be measured with the expression $\omega = (\mu^2/2\eta)$ (eV), where μ is the electronic chemical potential and η is the chemical hardness. The values of μ and η can be determined in terms of the one-electron energy of HOMO and LUMO (E_{HOMO} and E_{LUMO}) using $\mu \approx (E_{HOMO} - E_{LUMO})/2$ and $\eta \approx (E_{HOMO} - E_{LUMO})$. Within the Kohn–Sham scheme, the molecular nucleophilicity character is reflected by the value of the nucleophilicity index N as defined in $N = E_{HOMO}(Nu) - E_{LUMO}(TCE)$, where $E_{LUMO}(TCE)$ is the LUMO energy of tetracyanoethylene (TCE). The local electrophilicity (ω^{loc}) and local nucleophilicity (N^{loc}) are defined as $\omega^{loc} = \omega f^{+}(r)$ and $N^{loc} = N f^{-}(r)$, respectively, where f(r) is the corresponding Fukui function.

2. Formation of active catalysts NHC

(a) Precatalyst **Pre-NHC^{I}** forms active catalyst **NHC^{I}**.

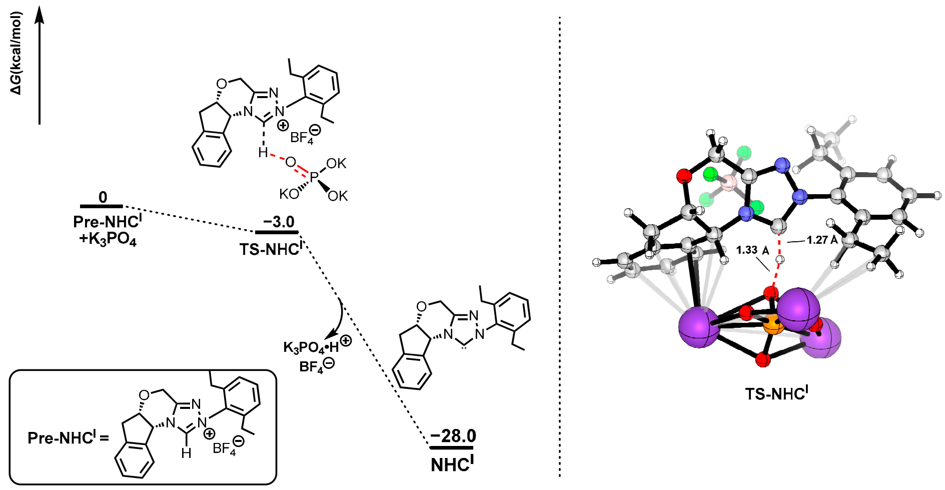

(b) IRC diagram for the formation of the active catalyst **NHC^{I}**

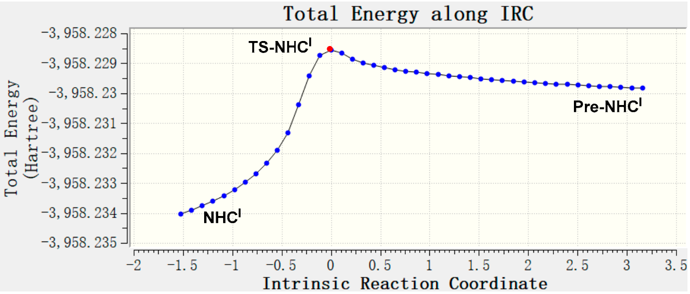

Figure S1. (**a**) Precatalyst **Pre-NHC^{I}** forms active catalyst **NHC^{I};** (**b**) IRC diagram for the formation of the active catalyst **NHC^{I}**.

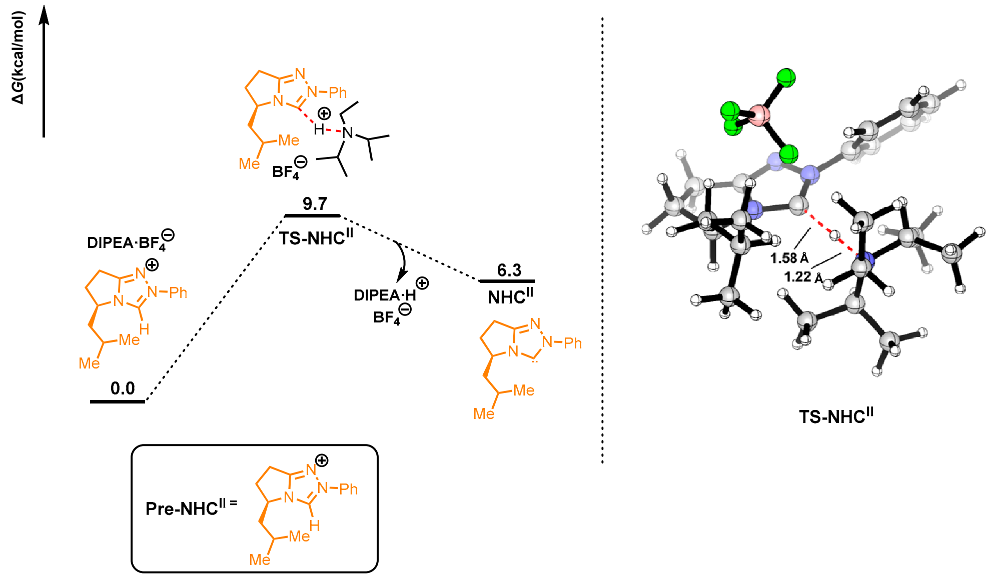

Figure S2. Precatalyst **Pre-NHC^{II}** forms active catalyst **NHC^{II}**.

3. Energies and Cartesian coordinates for all the intermediates and transition states

The process of generating product **a** :

1a

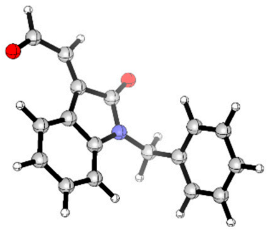

Zero-point correction=	0.261895 (Hartree/Particle)
Thermal correction to Energy=	0.277834
Thermal correction to Enthalpy=	0.278778
Thermal correction to Gibbs Free Energy=	0.216704
Sum of electronic and zero-point Energies=	-860.239191
Sum of electronic and thermal Energies=	-860.223253
Sum of electronic and thermal Enthalpies=	-860.222308
Sum of electronic and thermal Free Energies=	-860.284383

E(RM062X) = -860.712699046

C	0.611200000000	3.127252000000	-0.163952000000
C	-0.142999000000	2.035958000000	-0.603610000000
C	0.451297000000	0.788032000000	-0.546204000000
C	1.763264000000	0.598223000000	-0.055716000000
C	2.495778000000	1.700999000000	0.376866000000
C	1.908538000000	2.965772000000	0.315501000000
N	-0.104204000000	-0.433448000000	-0.942700000000
C	0.772016000000	-1.466572000000	-0.702278000000
C	2.033536000000	-0.836682000000	-0.130787000000
O	0.560285000000	-2.643624000000	-0.904482000000
C	3.049600000000	-1.668001000000	0.163951000000
C	4.364621000000	-1.310814000000	0.722115000000
O	4.728593000000	-0.189110000000	1.003778000000
C	-2.525571000000	-0.435858000000	-0.468408000000
C	-2.510990000000	-1.202536000000	0.699068000000
C	-3.514163000000	-1.050660000000	1.648670000000
C	-4.542027000000	-0.131488000000	1.440128000000
C	-4.563225000000	0.631802000000	0.277929000000
C	-3.555431000000	0.479024000000	-0.672621000000
C	-1.429957000000	-0.618731000000	-1.496565000000
H	0.170516000000	4.118731000000	-0.196901000000
H	-1.159533000000	2.159238000000	-0.960332000000
H	3.500647000000	1.565090000000	0.753068000000
H	2.470003000000	3.831324000000	0.649162000000
H	2.870990000000	-2.723177000000	-0.035388000000
H	5.044918000000	-2.169744000000	0.880633000000
H	-1.711369000000	-1.923783000000	0.851213000000
H	-3.497705000000	-1.651124000000	2.552504000000
H	-5.323270000000	-0.012155000000	2.183729000000
H	-5.359445000000	1.350099000000	0.111176000000
H	-3.570883000000	1.075734000000	-1.582013000000
H	-1.441903000000	-1.637313000000	-1.896070000000
H	-1.567473000000	0.078778000000	-2.330767000000

2a

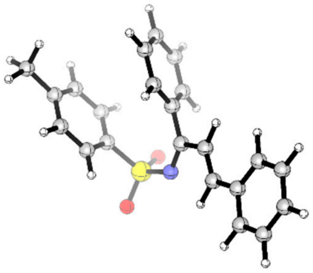

Zero-point correction=	0.358807 (Hartree/Particle)
Thermal correction to Energy=	0.381703
Thermal correction to Enthalpy=	0.382647
Thermal correction to Gibbs Free Energy=	0.303577
Sum of electronic and zero-point Energies=	-1452.272673
Sum of electronic and thermal Energies=	-1452.249777
Sum of electronic and thermal Enthalpies=	-1452.248833
Sum of electronic and thermal Free Energies=	-1452.327903

E(RM062X) = -1452.91352720

C	1.861865000000	0.372190000000	-0.419312000000
C	0.500034000000	-0.133695000000	-0.668982000000
C	2.869626000000	-0.430503000000	-0.038959000000
N	0.313898000000	-1.407883000000	-0.641378000000
C	-0.526385000000	0.922686000000	-0.927772000000
C	-1.179781000000	1.001008000000	-2.159419000000
C	-2.109174000000	2.014527000000	-2.380332000000
C	-2.402258000000	2.930874000000	-1.374377000000
C	-1.749142000000	2.851367000000	-0.145312000000
C	-0.797266000000	1.862034000000	0.071160000000
C	-2.288303000000	-1.191662000000	0.275024000000
C	-2.019040000000	-1.140024000000	1.638330000000
C	-2.827720000000	-0.354319000000	2.452038000000
C	-3.902920000000	0.361984000000	1.919073000000
C	-4.168851000000	0.260295000000	0.550020000000
C	-3.365902000000	-0.511561000000	-0.279859000000
C	-4.755554000000	1.245817000000	2.791487000000
S	-1.207425000000	-2.136173000000	-0.778745000000
O	-1.031141000000	-3.440238000000	-0.169251000000
O	-1.678349000000	-2.016593000000	-2.153784000000
C	4.252627000000	-0.028339000000	0.225346000000
C	5.141383000000	-0.981751000000	0.738874000000
C	6.463153000000	-0.649023000000	1.014434000000
C	6.917376000000	0.644203000000	0.775734000000
C	6.044922000000	1.602175000000	0.258045000000
C	4.726228000000	1.270082000000	-0.015734000000
H	1.998625000000	1.440946000000	-0.552776000000
H	2.636519000000	-1.486529000000	0.092489000000
H	-0.972859000000	0.261878000000	-2.925575000000
H	-2.611365000000	2.080642000000	-3.340085000000
H	-3.137431000000	3.710339000000	-1.547617000000
H	-1.975636000000	3.564140000000	0.641290000000
H	-0.279964000000	1.795548000000	1.024555000000
H	-1.189092000000	-1.705946000000	2.051032000000
H	-2.624814000000	-0.298572000000	3.517753000000
H	-5.009370000000	0.804328000000	0.127068000000
H	-3.546603000000	-0.580927000000	-1.347275000000
H	-4.563329000000	1.065781000000	3.851060000000
H	-4.549231000000	2.301460000000	2.585995000000
H	-5.819026000000	1.077789000000	2.602218000000
H	4.783811000000	-1.991282000000	0.921864000000
H	7.138122000000	-1.399080000000	1.413211000000
H	7.948890000000	0.906534000000	0.987687000000
H	6.398552000000	2.609603000000	0.064035000000
H	4.062121000000	2.021739000000	-0.430134000000

Pre-NHC[I]

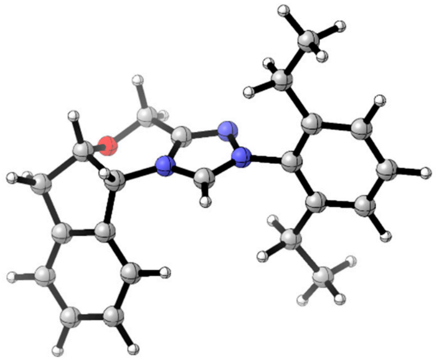

Zero-point correction=	0.429132 (Hartree/Particle)
Thermal correction to Energy=	0.450795
Thermal correction to Enthalpy=	0.451739
Thermal correction to Gibbs Free Energy=	0.376576
Sum of electronic and zero-point Energies=	-1091.169037
Sum of electronic and thermal Energies=	-1091.147374
Sum of electronic and thermal Enthalpies=	-1091.146429
Sum of electronic and thermal Free Energies=	-1091.221593

E(RM062X) = -1091.87366295

C	0.285793000000	-0.261002000000	-0.743274000000
N	1.272204000000	-0.358316000000	0.130588000000
N	0.883328000000	-1.036352000000	1.240087000000
C	-0.363862000000	-1.346585000000	1.013776000000
N	-0.771814000000	-0.881530000000	-0.210157000000
C	-1.326217000000	-2.114395000000	1.869501000000
O	-2.634311000000	-1.743061000000	1.519465000000
C	-2.950353000000	-1.992312000000	0.161125000000
C	-2.136766000000	-1.030803000000	-0.750387000000
C	-4.399584000000	-1.540552000000	-0.042720000000
C	-4.259310000000	-0.065741000000	-0.342945000000
C	-2.955698000000	0.240542000000	-0.732925000000
C	-5.219955000000	0.935741000000	-0.284828000000
C	-4.853649000000	2.239585000000	-0.616476000000
C	-3.547575000000	2.539622000000	-1.005045000000
C	-2.584246000000	1.534574000000	-1.071557000000
C	2.600875000000	0.198251000000	0.002059000000
C	2.761173000000	1.552240000000	0.313038000000
C	4.050891000000	2.066019000000	0.179986000000
C	5.099395000000	1.250461000000	-0.234861000000
C	4.894067000000	-0.095934000000	-0.521437000000
C	3.625001000000	-0.661880000000	-0.404729000000
C	4.535979000000	-2.935283000000	-1.163245000000
C	1.935915000000	3.788118000000	1.260711000000
C	3.337741000000	-2.123558000000	-0.682531000000
C	1.582648000000	2.383210000000	0.783095000000
H	-1.160910000000	-3.193160000000	1.732291000000
H	-1.172652000000	-1.864765000000	2.920217000000
H	-2.768261000000	-3.047398000000	-0.081820000000
H	-2.036087000000	-1.447824000000	-1.758811000000
H	-4.852309000000	-2.076931000000	-0.884443000000
H	-4.986210000000	-1.756670000000	0.853432000000
H	-6.236357000000	0.710770000000	0.023245000000
H	-5.592362000000	3.032722000000	-0.567796000000
H	-3.280246000000	3.560182000000	-1.257127000000
H	-1.569476000000	1.780127000000	-1.376769000000
H	4.241210000000	3.108041000000	0.410172000000
H	6.095609000000	1.669989000000	-0.330099000000
H	5.730052000000	-0.710673000000	-0.834865000000
H	4.235505000000	-3.968547000000	-1.348992000000
H	4.945354000000	-2.532264000000	-2.093868000000
H	5.332255000000	-2.950203000000	-0.414080000000
H	1.037874000000	4.295548000000	1.619067000000
H	2.655430000000	3.756240000000	2.083461000000
H	2.360448000000	4.392036000000	0.454139000000
H	2.934355000000	-2.575743000000	0.232303000000
H	2.535100000000	-2.190438000000	-1.430345000000
H	0.847761000000	2.461012000000	-0.031078000000
H	1.076807000000	1.841661000000	1.592837000000

H 0.341987000000 0.222248000000 -1.708418000000

TS-NHC[I]

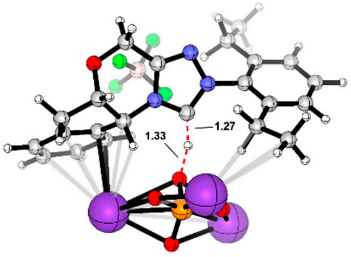

Frequency -847.1906

Zero-point correction= 0.459270 (Hartree/Particle)

Thermal correction to Energy= 0.497753

Thermal correction to Enthalpy= 0.498697

Thermal correction to Gibbs Free Energy= 0.385456

Sum of electronic and zero-point Energies= -3957.776865

Sum of electronic and thermal Energies= -3957.738382

Sum of electronic and thermal Enthalpies= -3957.737438

Sum of electronic and thermal Free Energies= -3957.850679

E(RM062X) = -3958.91232251

C	-0.683234000000	-0.238495000000	0.436382000000
H	-0.376561000000	0.668288000000	-0.391980000000
N	-1.751594000000	-0.931723000000	0.823530000000
N	0.316184000000	-0.763042000000	1.162940000000
N	-1.461165000000	-1.894715000000	1.760873000000
C	-3.099986000000	-0.728989000000	0.378545000000
C	-0.177429000000	-1.777385000000	1.928115000000
C	1.671967000000	-0.264332000000	1.259319000000
C	-3.764233000000	-1.792093000000	-0.249851000000
C	-3.682393000000	0.527993000000	0.598767000000
C	0.723948000000	-2.539508000000	2.851620000000
O	2.074874000000	-2.149877000000	2.708716000000
H	0.384161000000	-2.398365000000	3.888333000000
H	0.675914000000	-3.595913000000	2.586217000000
C	2.301781000000	-0.761646000000	2.571435000000
C	2.673708000000	-0.718020000000	0.225241000000
H	1.605884000000	0.835735000000	1.241725000000
C	3.805277000000	-0.567367000000	2.329419000000
H	1.923571000000	-0.216740000000	3.450901000000
C	3.932397000000	-0.759521000000	0.830861000000
H	4.108391000000	0.439592000000	2.641456000000
H	4.382970000000	-1.295656000000	2.903985000000
C	2.501705000000	-0.988753000000	-1.124965000000
C	5.067949000000	-0.984286000000	0.061072000000
C	4.913318000000	-1.220317000000	-1.308995000000
H	6.053356000000	-1.018789000000	0.517651000000
C	3.642065000000	-1.253592000000	-1.887345000000
H	1.508497000000	-1.026570000000	-1.559530000000
H	5.787261000000	-1.432285000000	-1.917518000000
H	3.527244000000	-1.522807000000	-2.932129000000
C	-5.066973000000	-1.549225000000	-0.689140000000
C	-3.070773000000	-3.116450000000	-0.454172000000
C	-4.984213000000	0.719455000000	0.132706000000
C	-2.922224000000	1.623870000000	1.323012000000
C	-5.667738000000	-0.309503000000	-0.506284000000
H	-5.610790000000	-2.342995000000	-1.189671000000
C	-3.728671000000	-4.061066000000	-1.451877000000
H	-2.048391000000	-2.922890000000	-0.780926000000
H	-2.972181000000	-3.614204000000	0.516941000000
H	-6.681419000000	-0.143628000000	-0.858447000000
H	-5.475954000000	1.674663000000	0.281487000000
C	-3.797035000000	2.771758000000	1.823787000000
H	-2.400676000000	1.163713000000	2.173748000000
H	-2.149858000000	2.038634000000	0.658867000000
H	-3.220494000000	3.453741000000	2.459704000000

H	-4.190663000000	3.366663000000	0.995565000000
H	-4.639114000000	2.410811000000	2.421329000000
H	-3.055496000000	-4.904426000000	-1.615582000000
H	-4.690942000000	-4.447108000000	-1.099715000000
H	-3.890283000000	-3.570368000000	-2.417206000000
O	0.297756000000	1.498912000000	-1.178115000000
P	0.564508000000	2.974398000000	-0.614108000000
K	-0.986610000000	2.989826000000	-2.961841000000
O	1.414013000000	2.855391000000	0.687793000000
O	-0.823518000000	3.620534000000	-0.332496000000
O	1.318036000000	3.681869000000	-1.758360000000
K	3.182403000000	1.906136000000	-1.210142000000
K	-0.323703000000	4.018642000000	2.080000000000
B	0.495606000000	-3.410845000000	-1.098347000000
F	-0.410047000000	-4.463376000000	-1.125346000000
F	1.476375000000	-3.545597000000	-2.071305000000
F	-0.215338000000	-2.191773000000	-1.343658000000
F	1.084750000000	-3.303896000000	0.180108000000

NHC[I]

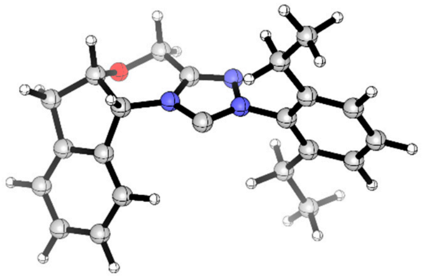

Zero-point correction=	0.414575 (Hartree/Particle)
Thermal correction to Energy=	0.436011
Thermal correction to Enthalpy=	0.436955
Thermal correction to Gibbs Free Energy=	0.362700
Sum of electronic and zero-point Energies=	-1090.787798
Sum of electronic and thermal Energies=	-1090.766362
Sum of electronic and thermal Enthalpies=	-1090.765418
Sum of electronic and thermal Free Energies=	-1090.839673

E(RM062X) = -1091.45876140

C	0.283276000	-0.435084000	-0.758991000
N	1.222530000	-0.356657000	0.206881000
N	0.831297000	-0.734698000	1.477758000
C	-0.408625000	-1.064898000	1.299456000
N	-0.768941000	-0.907426000	-0.014214000
C	-1.396540000	-1.588721000	2.293133000
O	-2.705003000	-1.284138000	1.861917000
C	-2.996765000	-1.814540000	0.579661000
C	-2.118373000	-1.134722000	-0.502483000
C	-4.424224000	-1.371957000	0.236728000
C	-4.218174000	-0.021122000	-0.410953000
C	-2.894841000	0.123751000	-0.823845000
C	-5.130522000	1.004271000	-0.623821000
C	-4.693829000	2.171023000	-1.251274000
C	-3.367434000	2.307806000	-1.661333000
C	-2.450365000	1.277915000	-1.452950000
C	2.561408000	0.116571000	0.017776000
C	2.868592000	1.411127000	0.447403000
C	4.178257000	1.855266000	0.263977000
C	5.128158000	1.029393000	-0.327392000
C	4.790759000	-0.252451000	-0.748651000
C	3.492860000	-0.736300000	-0.581175000
C	4.175754000	-2.997663000	-1.578811000
C	2.179434000	3.706363000	1.358480000
C	3.062885000	-2.119451000	-1.019745000
C	1.784587000	2.262741000	1.071534000
H	-1.270963000	-2.676521000	2.409862000
H	-1.247445000	-1.115673000	3.264570000
H	-2.862898000	-2.906195000	0.592306000

H	-2.049472000	-1.777705000	-1.387510000
H	-4.882724000	-2.079365000	-0.464237000
H	-5.036856000	-1.342092000	1.141039000
H	-6.162001000	0.906889000	-0.297784000
H	-5.393721000	2.984032000	-1.416856000
H	-3.046244000	3.224833000	-2.144848000
H	-1.412774000	1.362197000	-1.763585000
H	4.458897000	2.854210000	0.579742000
H	6.142970000	1.390188000	-0.463423000
H	5.546187000	-0.881096000	-1.207350000
H	3.781342000	-3.979965000	-1.848598000
H	4.617847000	-2.559268000	-2.478219000
H	4.974613000	-3.146110000	-0.846191000
H	1.329891000	4.253983000	1.772643000
H	2.996006000	3.762214000	2.084226000
H	2.500287000	4.220762000	0.447830000
H	2.594030000	-2.618000000	-0.162083000
H	2.264771000	-2.006107000	-1.762567000
H	0.912414000	2.240926000	0.405505000
H	1.453987000	1.776508000	1.996866000

INTa1

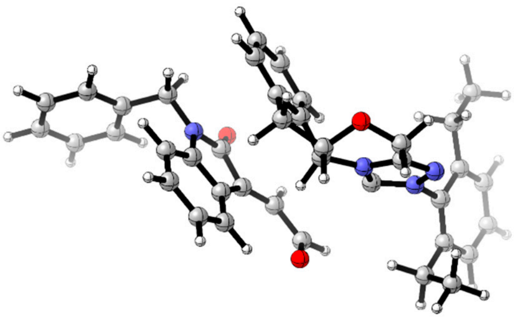

Zero-point correction=	0.678157 (Hartree/Particle)
Thermal correction to Energy=	0.717014
Thermal correction to Enthalpy=	0.717958
Thermal correction to Gibbs Free Energy=	0.604847
Sum of electronic and zero-point Energies=	-1951.058915
Sum of electronic and thermal Energies=	-1951.020058
Sum of electronic and thermal Enthalpies=	-1951.019114
Sum of electronic and thermal Free Energies=	-1951.132225

E(RM062X) = -1952.20429429

C	-3.863670000000	-2.586443000000	-1.659557000000
C	-4.110345000000	-1.578296000000	-0.723529000000
C	-3.226860000000	-0.512195000000	-0.681194000000
C	-2.106839000000	-0.428847000000	-1.538658000000
C	-1.879018000000	-1.446875000000	-2.461656000000
C	-2.767300000000	-2.524312000000	-2.517162000000
N	-3.281530000000	0.599023000000	0.159153000000
C	-2.263593000000	1.481649000000	-0.132013000000
C	-1.410991000000	0.815921000000	-1.195385000000
O	-2.098056000000	2.568499000000	0.382070000000
C	-0.238739000000	1.401128000000	-1.498575000000
C	0.807903000000	0.907507000000	-2.419065000000
O	0.764290000000	-0.148804000000	-3.018574000000
C	-5.606625000000	1.348882000000	0.485873000000
C	-5.595078000000	2.510985000000	-0.290412000000
C	-6.763229000000	2.961778000000	-0.892467000000
C	-7.954934000000	2.257230000000	-0.723146000000
C	-7.972965000000	1.102716000000	0.051756000000
C	-6.799768000000	0.650883000000	0.653664000000
C	-4.322648000000	0.873646000000	1.128896000000
H	-4.545557000000	-3.428971000000	-1.718875000000
H	-4.974817000000	-1.615686000000	-0.069959000000
H	-1.020971000000	-1.391477000000	-3.119071000000
H	-2.602095000000	-3.315582000000	-3.240010000000
H	-0.027449000000	2.338487000000	-0.987023000000
H	1.664345000000	1.591888000000	-2.544491000000

H	-4.662245000000	3.057811000000	-0.408545000000
H	-6.747804000000	3.865779000000	-1.492782000000
H	-8.866899000000	2.610219000000	-1.193850000000
H	-8.898121000000	0.551534000000	0.186979000000
H	-6.813252000000	-0.250253000000	1.262950000000
H	-3.911143000000	1.640435000000	1.792268000000
H	-4.492090000000	-0.030804000000	1.722864000000
C	2.469300000000	0.091558000000	-0.195038000000
N	3.783769000000	-0.109037000000	0.024537000000
N	4.136790000000	-1.358325000000	0.498940000000
C	2.991794000000	-1.965213000000	0.568576000000
N	1.982158000000	-1.136605000000	0.161097000000
C	2.692012000000	-3.383090000000	0.952333000000
O	1.340297000000	-3.507727000000	1.337401000000
C	0.437134000000	-3.056759000000	0.337851000000
C	0.588086000000	-1.533355000000	0.139067000000
C	-0.981178000000	-3.208187000000	0.900911000000
C	-1.174385000000	-1.926510000000	1.680210000000
C	-0.253302000000	-0.966304000000	1.259852000000
C	-2.080345000000	-1.625143000000	2.688343000000
C	-2.036813000000	-0.357604000000	3.274584000000
C	-1.107798000000	0.594083000000	2.854572000000
C	-0.202836000000	0.294259000000	1.835438000000
C	4.805403000000	0.874668000000	-0.176183000000
C	5.256724000000	1.122745000000	-1.474057000000
C	6.245824000000	2.095941000000	-1.636353000000
C	6.754156000000	2.781921000000	-0.541372000000
C	6.282234000000	2.513249000000	0.741092000000
C	5.292881000000	1.553131000000	0.947557000000
C	5.230156000000	2.105426000000	3.450776000000
C	5.457601000000	-1.027015000000	-2.761684000000
C	4.723672000000	1.229153000000	2.311582000000
C	4.749576000000	0.329310000000	-2.653871000000

H	2.917072000000	-4.046420000000	0.102824000000
H	3.306933000000	-3.685862000000	1.801109000000
H	0.602416000000	-3.613284000000	-0.596046000000
H	0.186688000000	-1.252958000000	-0.838585000000
H	-1.703430000000	-3.272510000000	0.076675000000
H	-1.059959000000	-4.113427000000	1.508091000000
H	-2.796415000000	-2.366725000000	3.032058000000
H	-2.732271000000	-0.111887000000	4.072042000000
H	-1.094572000000	1.577949000000	3.311874000000
H	0.519515000000	1.026890000000	1.484205000000
H	6.616145000000	2.308949000000	-2.635457000000
H	7.521833000000	3.535892000000	-0.684497000000
H	6.685220000000	3.062720000000	1.584981000000
H	4.745358000000	1.822287000000	4.387609000000
H	6.309983000000	1.998276000000	3.589375000000
H	5.015358000000	3.162149000000	3.266639000000
H	5.081287000000	-1.594619000000	-3.616712000000
H	6.535686000000	-0.889464000000	-2.885270000000
H	5.296704000000	-1.615207000000	-1.853952000000
H	3.630336000000	1.300582000000	2.246270000000
H	4.932674000000	0.175473000000	2.530739000000
H	3.670245000000	0.168379000000	-2.569808000000
H	4.919549000000	0.908547000000	-3.567234000000

----

^{f}**INTa1**

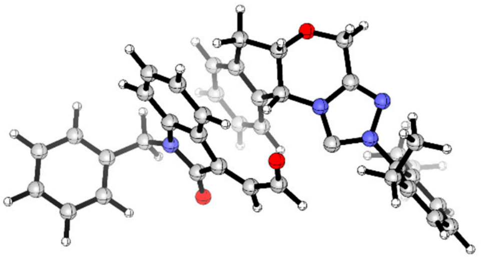

Zero-point correction= 0.678157 (Hartree/Particle)

Thermal correction to Energy= 0.717014

Thermal correction to Enthalpy= 0.717958

Thermal correction to Gibbs Free Energy= 0.604847

Sum of electronic and zero-point Energies= -1951.058915

Sum of electronic and thermal Energies= -1951.020058

Sum of electronic and thermal Enthalpies= -1951.019114

Sum of electronic and thermal Free Energies= -1951.132225

E(RM062X) = -1952.20429429

C	-3.863670000000	-2.586443000000	-1.659557000000
C	-4.110345000000	-1.578296000000	-0.723529000000
C	-3.226860000000	-0.512195000000	-0.681194000000
C	-2.106839000000	-0.428847000000	-1.538658000000
C	-1.879018000000	-1.446875000000	-2.461656000000
C	-2.767300000000	-2.524312000000	-2.517162000000
N	-3.281530000000	0.599023000000	0.159153000000
C	-2.263593000000	1.481649000000	-0.132013000000
C	-1.410991000000	0.815921000000	-1.195385000000
O	-2.098056000000	2.568499000000	0.382070000000
C	-0.238739000000	1.401128000000	-1.498575000000
C	0.807903000000	0.907507000000	-2.419065000000
O	0.764290000000	-0.148804000000	-3.018574000000
C	-5.606625000000	1.348882000000	0.485873000000
C	-5.595078000000	2.510985000000	-0.290412000000
C	-6.763229000000	2.961778000000	-0.892467000000
C	-7.954934000000	2.257230000000	-0.723146000000
C	-7.972965000000	1.102716000000	0.051756000000
C	-6.799768000000	0.650883000000	0.653664000000
C	-4.322648000000	0.873646000000	1.128896000000
H	-4.545557000000	-3.428971000000	-1.718875000000
H	-4.974817000000	-1.615686000000	-0.069959000000
H	-1.020971000000	-1.391477000000	-3.119071000000
H	-2.602095000000	-3.315582000000	-3.240010000000
H	-0.027449000000	2.338487000000	-0.987023000000
H	1.664345000000	1.591888000000	-2.544491000000
H	-4.662245000000	3.057811000000	-0.408545000000
H	-6.747804000000	3.865779000000	-1.492782000000
H	-8.866899000000	2.610219000000	-1.193850000000
H	-8.898121000000	0.551534000000	0.186979000000
H	-6.813252000000	-0.250253000000	1.262950000000
H	-3.911143000000	1.640435000000	1.792268000000
H	-4.492090000000	-0.030804000000	1.722864000000
C	2.469300000000	0.091558000000	-0.195038000000
N	3.783769000000	-0.109037000000	0.024537000000
N	4.136790000000	-1.358325000000	0.498940000000
C	2.991794000000	-1.965213000000	0.568576000000
N	1.982158000000	-1.136605000000	0.161097000000
C	2.692012000000	-3.383090000000	0.952333000000
O	1.340297000000	-3.507727000000	1.337401000000
C	0.437134000000	-3.056759000000	0.337851000000
C	0.588086000000	-1.533355000000	0.139067000000
C	-0.981178000000	-3.208187000000	0.900911000000
C	-1.174385000000	-1.926510000000	1.680210000000
C	-0.253302000000	-0.966304000000	1.259852000000
C	-2.080345000000	-1.625143000000	2.688343000000
C	-2.036813000000	-0.357604000000	3.274584000000
C	-1.107798000000	0.594083000000	2.854572000000
C	-0.202836000000	0.294259000000	1.835438000000
C	4.805403000000	0.874668000000	-0.176183000000
C	5.256724000000	1.122745000000	-1.474057000000
C	6.245824000000	2.095941000000	-1.636353000000
C	6.754156000000	2.781921000000	-0.541372000000
C	6.282234000000	2.513249000000	0.741092000000
C	5.292881000000	1.553131000000	0.947557000000

C	5.230156000000	2.105426000000	3.450776000000
C	5.457601000000	-1.027015000000	-2.761684000000
C	4.723672000000	1.229153000000	2.311582000000
C	4.749576000000	0.329310000000	-2.653871000000
H	2.917072000000	-4.046420000000	0.102824000000
H	3.306933000000	-3.685862000000	1.801109000000
H	0.602416000000	-3.613284000000	-0.596046000000
H	0.186688000000	-1.252958000000	-0.838585000000
H	-1.703430000000	-3.272510000000	0.076675000000
H	-1.059959000000	-4.113427000000	1.508091000000
H	-2.796415000000	-2.366725000000	3.032058000000
H	-2.732271000000	-0.111887000000	4.072042000000
H	-1.094572000000	1.577949000000	3.311874000000
H	0.519515000000	1.026890000000	1.484205000000
H	6.616145000000	2.308949000000	-2.635457000000
H	7.521833000000	3.535892000000	-0.684497000000
H	6.685220000000	3.062720000000	1.584981000000
H	4.745358000000	1.822287000000	4.387609000000
H	6.309983000000	1.998276000000	3.589375000000
H	5.015358000000	3.162149000000	3.266639000000
H	5.081287000000	-1.594619000000	-3.616712000000
H	6.535686000000	-0.889464000000	-2.885270000000
H	5.296704000000	-1.615207000000	-1.853952000000
H	3.630336000000	1.300582000000	2.246270000000
H	4.932674000000	0.175473000000	2.530739000000
H	3.670245000000	0.168379000000	-2.569808000000
H	4.919549000000	0.908547000000	-3.567234000000

TSa1

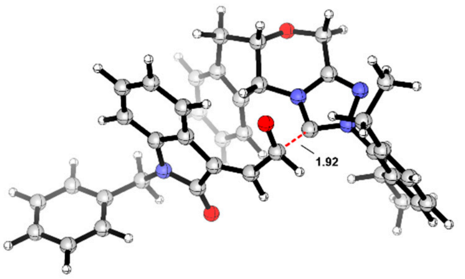

Frequency -189.6421

Zero-point correction= 0.678667 (Hartree/Particle)

Thermal correction to Energy= 0.716297

Thermal correction to Enthalpy= 0.717241

Thermal correction to Gibbs Free Energy= 0.606940

Sum of electronic and zero-point Energies= -1951.049289

Sum of electronic and thermal Energies= -1951.011659

Sum of electronic and thermal Enthalpies= -1951.010715

Sum of electronic and thermal Free Energies= -1951.121016

E(RM062X) = -1952.19330337

C	-1.374182000000	-0.951535000000	-1.504609000000
C	-2.307119000000	-0.026736000000	-0.107886000000
C	-0.188155000000	-1.419195000000	-0.710662000000
C	1.084876000000	-1.000280000000	-0.773464000000
C	1.835293000000	0.018473000000	-1.524937000000
C	3.090854000000	0.142054000000	-0.893652000000
C	4.046958000000	1.051828000000	-1.316120000000
C	3.729937000000	1.857291000000	-2.413026000000
C	2.503819000000	1.738563000000	-3.063384000000
H	4.460711000000	2.576821000000	-2.769038000000
H	5.010790000000	1.122366000000	-0.823270000000
N	3.187232000000	-0.760544000000	0.168341000000
C	1.547635000000	0.815964000000	-2.627923000000
H	0.585330000000	0.711817000000	-3.116180000000
H	2.287856000000	2.362669000000	-3.924195000000
C	2.028792000000	-1.501680000000	0.292305000000

C	4.286043000000	-0.837069000000	1.103101000000
O	1.825129000000	-2.346508000000	1.144016000000
H	-0.399845000000	-2.171702000000	0.051650000000
O	-1.281149000000	-0.092584000000	-2.415316000000
H	-2.132170000000	-1.761076000000	-1.587078000000
C	5.544519000000	-1.420257000000	0.497227000000
C	5.485571000000	-2.618778000000	-0.216415000000
C	6.773207000000	-0.783651000000	0.659561000000
C	6.640583000000	-3.169894000000	-0.758776000000
H	4.525886000000	-3.114136000000	-0.341273000000
C	7.867695000000	-2.529011000000	-0.591846000000
H	6.585606000000	-4.101647000000	-1.312705000000
C	7.932914000000	-1.335520000000	0.119101000000
H	8.768770000000	-2.959201000000	-1.017012000000
H	8.884636000000	-0.830229000000	0.249638000000
H	6.821871000000	0.152194000000	1.211888000000
H	3.926591000000	-1.465237000000	1.925042000000
H	4.482890000000	0.165155000000	1.503402000000
N	-3.625515000000	0.009478000000	0.099913000000
N	-1.925592000000	1.243416000000	0.167627000000
N	-4.100582000000	1.244092000000	0.484767000000
C	-4.532217000000	-1.091889000000	-0.066325000000
C	-3.027852000000	1.978840000000	0.502853000000
C	-2.912103000000	3.460986000000	0.713952000000
C	-0.604562000000	1.817653000000	-0.032183000000
O	-1.566488000000	3.839751000000	0.880750000000
H	-3.362133000000	3.969711000000	-0.152297000000
H	-3.453905000000	3.763700000000	1.611768000000
C	-0.709789000000	3.350137000000	-0.145159000000
C	0.710684000000	3.820210000000	0.180467000000
H	-1.075405000000	3.672792000000	-1.129655000000
C	0.383267000000	1.641388000000	1.099043000000
H	-0.227373000000	1.395297000000	-0.969787000000
C	1.180443000000	2.784672000000	1.178341000000
H	1.324642000000	3.791797000000	-0.728047000000
H	0.704782000000	4.840692000000	0.570686000000
C	2.222584000000	2.848393000000	2.091867000000
C	0.579471000000	0.565708000000	1.951432000000
C	2.442412000000	1.758970000000	2.937413000000
H	2.848196000000	3.733734000000	2.157519000000
C	1.623666000000	0.631891000000	2.875491000000
H	3.251215000000	1.795695000000	3.661183000000
H	1.803048000000	-0.209698000000	3.536996000000
H	-0.047265000000	-0.320313000000	1.894196000000
C	-5.039626000000	-1.349943000000	-1.341589000000
C	-4.849638000000	-1.851779000000	1.066364000000
C	-5.917630000000	-2.429824000000	-1.466427000000
C	-4.714339000000	-0.477299000000	-2.530947000000
C	-6.260423000000	-3.201037000000	-0.363935000000
H	-6.331205000000	-2.658390000000	-2.444614000000
C	-5.731801000000	-2.916093000000	0.893148000000
H	-6.944694000000	-4.035406000000	-0.481253000000
H	-6.007859000000	-3.530996000000	1.742625000000
C	-4.228648000000	-1.494863000000	2.399830000000
C	-4.659109000000	-2.371599000000	3.569477000000
H	-4.169014000000	-2.040806000000	4.487440000000
H	-5.740020000000	-2.320122000000	3.728743000000
H	-4.388666000000	-3.418340000000	3.404415000000
C	-5.628717000000	0.754013000000	-2.584794000000
H	-5.389535000000	1.370948000000	-3.454579000000
H	-6.678505000000	0.453969000000	-2.652318000000
H	-5.512302000000	1.362218000000	-1.683625000000
H	-3.136753000000	-1.537389000000	2.292687000000
H	-4.461402000000	-0.445553000000	2.617478000000
H	-3.664007000000	-0.163958000000	-2.522427000000
H	-4.851392000000	-1.072336000000	-3.439628000000

----

^{f}TSa1

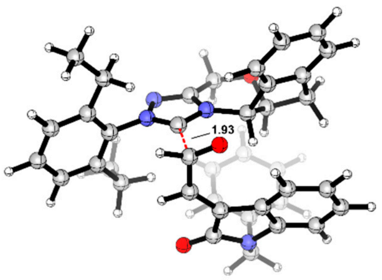

Frequency -179.3941

Zero-point correction= 0.678588 (Hartree/Particle)

Thermal correction to Energy= 0.716111

Thermal correction to Enthalpy= 0.717056

Thermal correction to Gibbs Free Energy= 0.607623

Sum of electronic and zero-point Energies= -1951.045863

Sum of electronic and thermal Energies= -1951.008340

Sum of electronic and thermal Enthalpies= -1951.007396

Sum of electronic and thermal Free Energies= -1951.116829

E(RM062X) = -1952.19005501

C	-1.196568000000	-1.120180000000	-2.009797000000
C	-1.435668000000	-0.838065000000	-0.111083000000
C	-0.800540000000	0.329849000000	-2.154306000000
C	0.435320000000	0.839235000000	-2.295067000000
C	1.798062000000	0.285285000000	-2.375966000000
C	2.676700000000	1.332828000000	-2.033219000000
C	4.045504000000	1.140160000000	-1.928513000000
C	4.543660000000	-0.137173000000	-2.197968000000
C	3.692267000000	-1.177851000000	-2.564174000000
H	5.611951000000	-0.314835000000	-2.122982000000
H	4.711051000000	1.951827000000	-1.654814000000
N	1.959969000000	2.523938000000	-1.847489000000
C	2.312968000000	-0.976706000000	-2.659853000000
H	1.628931000000	-1.783376000000	-2.897395000000
H	4.101290000000	-2.161634000000	-2.768197000000
C	0.602393000000	2.304830000000	-2.014659000000
C	2.433109000000	3.670284000000	-1.088798000000
O	-0.259239000000	3.151564000000	-1.850646000000
H	-1.602916000000	1.061413000000	-2.030695000000
O	-0.390192000000	-2.049463000000	-2.223114000000
H	-2.281091000000	-1.247198000000	-2.224111000000
C	2.360451000000	3.385139000000	0.398118000000
C	1.121756000000	3.439277000000	1.048596000000
C	3.490045000000	2.996241000000	1.118160000000
C	1.023020000000	3.115506000000	2.398061000000
H	0.241618000000	3.735085000000	0.482675000000
C	2.159043000000	2.732162000000	3.112783000000
H	0.057993000000	3.170163000000	2.895274000000
C	3.392200000000	2.672022000000	2.470739000000
H	2.083088000000	2.486566000000	4.167887000000
H	4.279043000000	2.375760000000	3.021748000000
H	4.455145000000	2.951650000000	0.620213000000
H	1.784007000000	4.507465000000	-1.355169000000
H	3.453436000000	3.911510000000	-1.399629000000
N	-2.391977000000	-0.281213000000	0.630979000000
N	-0.419675000000	-1.006409000000	0.765123000000
N	-2.031744000000	-0.067508000000	1.940109000000
C	-3.637899000000	0.219477000000	0.127320000000
C	-0.813487000000	-0.520716000000	1.985534000000
C	0.150033000000	-0.441775000000	3.131118000000
C	0.956302000000	-1.414980000000	0.473430000000
O	1.312941000000	-1.180224000000	2.858054000000
H	0.385942000000	0.617838000000	3.305206000000
H	-0.300095000000	-0.855479000000	4.035875000000
C	1.887847000000	-0.892806000000	1.586853000000
C	3.118927000000	-1.790352000000	1.460840000000

H	2.095215000000	0.183955000000	1.493424000000
C	1.259300000000	-2.894329000000	0.443127000000
H	1.217886000000	-0.968502000000	-0.485541000000
C	2.522514000000	-3.102599000000	1.000041000000
H	3.793676000000	-1.380697000000	0.698341000000
H	3.654989000000	-1.849520000000	2.411090000000
C	3.063849000000	-4.379870000000	1.043467000000
C	0.522591000000	-3.936815000000	-0.098186000000
C	2.319767000000	-5.440498000000	0.526196000000
H	4.043886000000	-4.552323000000	1.478962000000
C	1.064807000000	-5.220791000000	-0.040968000000
H	2.724630000000	-6.447201000000	0.560947000000
H	0.508331000000	-6.056587000000	-0.452835000000
H	-0.423322000000	-3.740561000000	-0.592474000000
C	-3.748701000000	1.595577000000	-0.082325000000
C	-4.660140000000	-0.697224000000	-0.150777000000
C	-4.964006000000	2.064869000000	-0.588682000000
C	-2.630445000000	2.559430000000	0.238306000000
C	-6.001560000000	1.187375000000	-0.870347000000
H	-5.081535000000	3.129870000000	-0.768860000000
C	-5.851845000000	-0.181812000000	-0.656083000000
H	-6.937268000000	1.568096000000	-1.267202000000
H	-6.673644000000	-0.851022000000	-0.885052000000
C	-4.435586000000	-2.173468000000	0.102035000000
C	-5.657903000000	-3.057772000000	-0.113041000000
H	-5.415575000000	-4.095676000000	0.123943000000
H	-6.490155000000	-2.750656000000	0.526873000000
H	-5.996830000000	-3.025277000000	-1.152276000000
C	-2.820260000000	3.218776000000	1.608929000000
H	-2.034256000000	3.958005000000	1.791662000000
H	-3.784048000000	3.733592000000	1.663692000000
H	-2.790374000000	2.465598000000	2.401045000000
H	-3.617806000000	-2.519348000000	-0.543641000000
H	-4.068086000000	-2.292756000000	1.128586000000
H	-1.660595000000	2.051042000000	0.205102000000
H	-2.585558000000	3.321301000000	-0.546119000000

INTa2

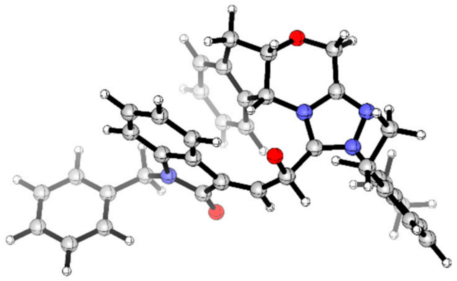

Zero-point correction= 0.680209 (Hartree/Particle)

Thermal correction to Energy= 0.717940

Thermal correction to Enthalpy= 0.718884

Thermal correction to Gibbs Free Energy= 0.608553

Sum of electronic and zero-point Energies= -1951.051857

Sum of electronic and thermal Energies= -1951.014126

Sum of electronic and thermal Enthalpies= -1951.013182

Sum of electronic and thermal Free Energies= -1951.123513

E(RM062X) = -1952.19805840

C	-1.495137000000	-0.927181000000	-1.172098000000
C	-2.303530000000	0.013297000000	-0.204597000000
C	-0.274745000000	-1.395695000000	-0.406434000000
C	1.013796000000	-1.071596000000	-0.584098000000
C	1.773722000000	-0.178159000000	-1.473554000000
C	3.055207000000	-0.036321000000	-0.903535000000
C	4.021381000000	0.794338000000	-1.447980000000
C	3.689627000000	1.487928000000	-2.614644000000
C	2.440200000000	1.336452000000	-3.212311000000
H	4.427200000000	2.144628000000	-3.065647000000
H	5.002128000000	0.888688000000	-0.994161000000

N	3.163215000000	-0.828439000000	0.245237000000
C	1.471858000000	0.498252000000	-2.650935000000
H	0.485792000000	0.363902000000	-3.084478000000
H	2.215232000000	1.870186000000	-4.129857000000
C	1.980555000000	-1.495238000000	0.495384000000
C	4.282279000000	-0.821999000000	1.157353000000
O	1.770722000000	-2.215967000000	1.453877000000
H	-0.470823000000	-2.049006000000	0.449331000000
O	-1.354132000000	-0.187366000000	-2.254166000000
H	-2.156110000000	-1.826988000000	-1.258773000000
C	5.545326000000	-1.405527000000	0.560108000000
C	5.496398000000	-2.592639000000	-0.172159000000
C	6.773011000000	-0.773568000000	0.750725000000
C	6.659765000000	-3.139184000000	-0.702260000000
H	4.537489000000	-3.080421000000	-0.326790000000
C	7.885181000000	-2.504589000000	-0.505339000000
H	6.611540000000	-4.061457000000	-1.272424000000
C	7.940312000000	-1.320590000000	0.222835000000
H	8.792329000000	-2.930159000000	-0.922149000000
H	8.890370000000	-0.818159000000	0.375042000000
H	6.813869000000	0.156283000000	1.313445000000
H	3.956716000000	-1.408574000000	2.023072000000
H	4.463267000000	0.207438000000	1.493817000000
N	-3.608207000000	-0.002504000000	0.064803000000
N	-1.908237000000	1.265935000000	0.112219000000
N	-4.074684000000	1.213500000000	0.504300000000
C	-4.501296000000	-1.123924000000	-0.055300000000
C	-3.013494000000	1.966207000000	0.502021000000
C	-2.934170000000	3.453442000000	0.703222000000
C	-0.613860000000	1.880330000000	-0.154736000000
O	-1.599742000000	3.894509000000	0.738444000000
H	-3.490132000000	3.930815000000	-0.117780000000
H	-3.400954000000	3.739733000000	1.647633000000
C	-0.796736000000	3.398362000000	-0.329127000000
C	0.620491000000	3.936748000000	-0.116424000000
H	-1.242856000000	3.660854000000	-1.297937000000
C	0.413080000000	1.814449000000	0.955148000000
H	-0.261428000000	1.418718000000	-1.085719000000
C	1.171958000000	2.987120000000	0.925456000000
H	1.184053000000	3.862594000000	-1.054294000000
H	0.601221000000	4.981248000000	0.203029000000
C	2.237711000000	3.152927000000	1.797465000000
C	0.663836000000	0.821997000000	1.889066000000
C	2.520278000000	2.138767000000	2.715403000000
H	2.832564000000	4.061296000000	1.779493000000
C	1.733255000000	0.989612000000	2.770022000000
H	3.348551000000	2.255632000000	3.407707000000
H	1.952267000000	0.208641000000	3.491352000000
H	0.061372000000	-0.080075000000	1.933030000000
C	-4.987925000000	-1.456963000000	-1.321720000000
C	-4.822103000000	-1.819022000000	1.117183000000
C	-5.856048000000	-2.549641000000	-1.390879000000
C	-4.640901000000	-0.658034000000	-2.556087000000
C	-6.206752000000	-3.257679000000	-0.248915000000
H	-6.253760000000	-2.841075000000	-2.358537000000
C	-5.695271000000	-2.897980000000	0.995573000000
H	-6.882031000000	-4.104006000000	-0.325611000000
H	-5.973373000000	-3.468186000000	1.874779000000
C	-4.215343000000	-1.387668000000	2.435797000000
C	-4.604669000000	-2.240865000000	3.637125000000
H	-4.116214000000	-1.864829000000	4.538322000000
H	-5.684674000000	-2.218961000000	3.807815000000
H	-4.301492000000	-3.282713000000	3.501233000000
C	-5.486658000000	0.619146000000	-2.649149000000
H	-5.233825000000	1.178972000000	-3.552799000000
H	-6.553818000000	0.380252000000	-2.677252000000

H -5.314116000000 1.266172000000 -1.784299000000

H -3.121969000000 -1.388828000000 2.329727000000

H -4.492302000000 -0.342636000000 2.618603000000

H -3.572578000000 -0.404336000000 -2.584927000000

H -4.836429000000 -1.286349000000 -3.430755000000

----

^{f}INTa2

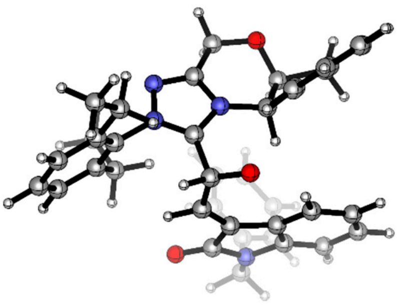

Zero-point correction= 0.679246 (Hartree/Particle)

Thermal correction to Energy= 0.717134

Thermal correction to Enthalpy= 0.718078

Thermal correction to Gibbs Free Energy= 0.606051

Sum of electronic and zero-point Energies= -1951.047804

Sum of electronic and thermal Energies= -1951.009916

Sum of electronic and thermal Enthalpies= -1951.008972

Sum of electronic and thermal Free Energies= -1951.120999

E(RM062X) = -1952.19298363

C -3.854278000000 2.357104000000 -2.229170000000

C -4.150914000000 1.016642000000 -1.968895000000

C -3.089850000000 0.128040000000 -1.905455000000

C -1.751095000000 0.540631000000 -2.063977000000

C -1.476724000000 1.874628000000 -2.351421000000

C -2.541088000000 2.777893000000 -2.427049000000

N -3.145094000000 -1.256463000000 -1.697942000000

C -1.878144000000 -1.799794000000 -1.665679000000

C -0.916898000000 -0.659371000000 -1.876311000000

O -1.628995000000 -2.972423000000 -1.435942000000

C 0.389043000000 -0.928697000000 -1.730984000000

C 1.559008000000 0.056297000000 -1.645457000000

O 1.382666000000 1.290111000000 -2.032363000000

C -4.742539000000 -1.812075000000 0.080284000000

C -3.851939000000 -2.174540000000 1.095506000000

C -4.193672000000 -1.982324000000 2.428276000000

C -5.428806000000 -1.426359000000 2.761832000000

C -6.319190000000 -1.067143000000 1.756268000000

C -5.974481000000 -1.259584000000 0.419119000000

C -4.333303000000 -2.012830000000 -1.363674000000

H -4.665444000000 3.076567000000 -2.283564000000

H -5.172847000000 0.686901000000 -1.814240000000

H -0.439815000000 2.167114000000 -2.494081000000

H -2.337396000000 3.821239000000 -2.644366000000

H 0.624318000000 -1.985819000000 -1.581399000000

H 2.436629000000 -0.505928000000 -2.067558000000

H -2.895567000000 -2.616405000000 0.823381000000

H -3.499514000000 -2.271810000000 3.211555000000

H -5.694816000000 -1.276463000000 3.803334000000

H -7.281496000000 -0.633455000000 2.009274000000

H -6.671087000000 -0.978409000000 -0.367488000000

H -4.081858000000 -3.061084000000 -1.548445000000

H -5.144018000000 -1.726834000000 -2.042316000000

C 1.813653000000 -0.011734000000 -0.093632000000

N 2.413569000000 -0.940762000000 0.646318000000

N 2.311615000000 -0.695757000000 1.988648000000

C 1.639147000000 0.416644000000 2.043075000000

N 1.327331000000 0.880983000000 0.791562000000

C 1.097945000000 1.085158000000 3.270397000000

O 0.605575000000 2.359920000000 2.964104000000

C -0.296312000000 2.376136000000 1.861490000000

C 0.464028000000 2.054593000000 0.554569000000

C	-0.703899000000	3.837059000000	1.681412000000
C	0.459480000000	4.395718000000	0.893971000000
C	1.149782000000	3.369736000000	0.245024000000
C	0.863003000000	5.715580000000	0.748875000000
C	1.972688000000	5.990852000000	-0.049580000000
C	2.648701000000	4.962407000000	-0.705466000000
C	2.236308000000	3.636138000000	-0.576060000000
C	2.972058000000	-2.174844000000	0.166440000000
C	2.169140000000	-3.315447000000	0.232913000000
C	2.721993000000	-4.503979000000	-0.251293000000
C	4.009262000000	-4.527930000000	-0.769065000000
C	4.780834000000	-3.367604000000	-0.815778000000
C	4.275160000000	-2.157866000000	-0.345178000000
C	6.503794000000	-0.990138000000	-0.831590000000
C	0.783486000000	-3.769656000000	2.285568000000
C	5.059185000000	-0.862229000000	-0.363192000000
C	0.779758000000	-3.303332000000	0.825533000000
H	0.309810000000	0.439704000000	3.689071000000
H	1.884267000000	1.198710000000	4.018882000000
H	-1.132320000000	1.686325000000	2.044727000000
H	-0.218787000000	1.777974000000	-0.250942000000
H	-1.634339000000	3.895527000000	1.104141000000
H	-0.862113000000	4.315274000000	2.650854000000
H	0.331720000000	6.516810000000	1.254273000000
H	2.309586000000	7.016303000000	-0.166115000000
H	3.499854000000	5.198336000000	-1.336203000000
H	2.692559000000	2.826692000000	-1.135630000000
H	2.122890000000	-5.409681000000	-0.226003000000
H	4.420196000000	-5.458641000000	-1.146928000000
H	5.784664000000	-3.410357000000	-1.223078000000
H	6.999783000000	-0.018827000000	-0.781624000000
H	7.065749000000	-1.688619000000	-0.205013000000
H	6.558153000000	-1.338472000000	-1.866562000000
H	-0.233167000000	-3.777357000000	2.687954000000
H	1.189885000000	-4.781550000000	2.369793000000
H	1.398397000000	-3.104904000000	2.898126000000
H	4.534031000000	-0.141693000000	-1.004339000000
H	5.034502000000	-0.433951000000	0.646468000000
H	0.343082000000	-2.300674000000	0.760635000000
H	0.132649000000	-3.942122000000	0.217070000000

K_3PO_4

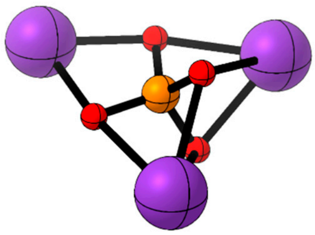

Zero-point correction=	0.018775 (Hartree/Particle)
Thermal correction to Energy=	0.028286
Thermal correction to Enthalpy=	0.029231
Thermal correction to Gibbs Free Energy=	-0.018349
Sum of electronic and zero-point Energies=	-2442.001211
Sum of electronic and thermal Energies=	-2441.991699
Sum of electronic and thermal Enthalpies=	-2441.990755
Sum of electronic and thermal Free Energies=	-2442.038335

E(RM062X) = -2442.30255746

O	-0.132520000000	1.570361000000	1.196139000000
P	-1.540027000000	1.221783000000	1.788933000000
K	-0.636821000000	3.599008000000	2.811496000000
O	-2.051716000000	-0.139293000000	1.202347000000
O	-2.556531000000	2.378621000000	1.494386000000
O	-1.311634000000	1.089201000000	3.334288000000

K	-4.156733000000	0.857030000000	0.404071000000
K	0.231123000000	-0.800692000000	2.315296000000

H^+ • K_3PO_4

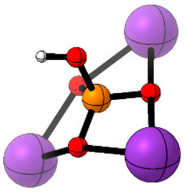

Zero-point correction= 0.029824 (Hartree/Particle)

Thermal correction to Energy= 0.040467

Thermal correction to Enthalpy= 0.041411

Thermal correction to Gibbs Free Energy= -0.009920

Sum of electronic and zero-point Energies= -2442.471462

Sum of electronic and thermal Energies= -2442.460818

Sum of electronic and thermal Enthalpies= -2442.459874

Sum of electronic and thermal Free Energies= -2442.511205

E(RM062X) = -2442.77625546

O	-0.814430000000	0.002975000000	-1.673373000000
P	-0.072914000000	0.000057000000	-0.198500000000
K	-1.333885000000	-2.556754000000	0.752730000000
O	0.781874000000	1.274717000000	-0.100330000000
O	0.774416000000	-1.279731000000	-0.101649000000
O	-1.273069000000	0.003088000000	0.755949000000
K	2.984020000000	-0.008977000000	0.095576000000
K	-1.319784000000	2.562942000000	0.754606000000
H	-0.167829000000	0.001349000000	-2.393865000000

INTa3''

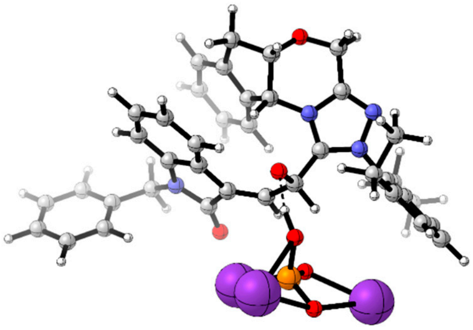

Zero-point correction= 0.712779 (Hartree/Particle)

Thermal correction to Energy= 0.761774

Thermal correction to Enthalpy= 0.762718

Thermal correction to Gibbs Free Energy= 0.625880

Sum of electronic and zero-point Energies= -4393.597492

Sum of electronic and thermal Energies= -4393.548497

Sum of electronic and thermal Enthalpies= -4393.547553

Sum of electronic and thermal Free Energies= -4393.684391

E(RM062X) = -4395.03700856

C	-1.135623000000	0.177541000000	-0.339409000000
C	-1.559975000000	1.622389000000	-0.080625000000
C	0.032313000000	-0.227943000000	0.524528000000
C	1.315524000000	-0.422474000000	0.173112000000
C	2.174616000000	-0.083813000000	-0.975429000000
C	3.485193000000	0.007122000000	-0.465580000000
C	4.555569000000	0.438521000000	-1.229699000000
C	4.300213000000	0.757165000000	-2.565322000000
C	3.022756000000	0.619894000000	-3.106994000000
H	5.118536000000	1.093176000000	-3.193727000000
H	5.554140000000	0.508663000000	-0.812213000000
N	3.512622000000	-0.403876000000	0.879423000000
C	1.948842000000	0.195335000000	-2.319392000000
H	0.943038000000	0.088438000000	-2.712034000000
H	2.859490000000	0.837547000000	-4.157467000000
C	2.263768000000	-0.756566000000	1.294792000000

C	4.693552000000	-0.454413000000	1.713989000000
O	1.975574000000	-1.260259000000	2.379216000000
H	-0.204902000000	-0.419580000000	1.573992000000
O	-0.963672000000	0.056101000000	-1.676737000000
H	-1.974685000000	-0.422285000000	0.063804000000
C	5.749707000000	-1.415598000000	1.209251000000
C	5.400512000000	-2.585970000000	0.535870000000
C	7.096732000000	-1.138143000000	1.440264000000
C	6.385374000000	-3.470472000000	0.107235000000
H	4.353253000000	-2.795323000000	0.331469000000
C	7.728572000000	-3.193192000000	0.349264000000
H	6.104729000000	-4.374885000000	-0.422983000000
C	8.082905000000	-2.024532000000	1.017364000000
H	8.495779000000	-3.881924000000	0.011647000000
H	9.127681000000	-1.798392000000	1.203460000000
H	7.375628000000	-0.222312000000	1.955757000000
H	4.341760000000	-0.747877000000	2.708381000000
H	5.109220000000	0.558638000000	1.788046000000
N	-2.786473000000	2.052749000000	0.210348000000
N	-0.874411000000	2.729962000000	-0.411964000000
N	-2.915817000000	3.407191000000	0.049593000000
C	-3.923087000000	1.264272000000	0.616817000000
C	-1.734939000000	3.789388000000	-0.347151000000
C	-1.305543000000	5.136634000000	-0.861309000000
C	0.471752000000	2.806954000000	-0.965817000000
O	0.083946000000	5.176904000000	-1.073149000000
H	-1.859218000000	5.331513000000	-1.791921000000
H	-1.552525000000	5.919386000000	-0.141874000000
C	0.593670000000	4.079627000000	-1.822424000000
C	2.109505000000	4.263631000000	-1.939753000000
H	0.071539000000	3.995286000000	-2.785027000000
C	1.617418000000	2.968711000000	0.009460000000
H	0.585868000000	1.906142000000	-1.577305000000

C	2.607210000000	3.734433000000	-0.611397000000
H	2.490696000000	3.663152000000	-2.774222000000
H	2.362250000000	5.311565000000	-2.116013000000
C	3.825325000000	3.943476000000	0.018409000000
C	1.789070000000	2.477904000000	1.294509000000
C	4.026325000000	3.405046000000	1.290941000000
H	4.602232000000	4.532748000000	-0.459015000000
C	3.009472000000	2.700499000000	1.934261000000
H	4.971044000000	3.569578000000	1.799739000000
H	3.163518000000	2.322469000000	2.940491000000
H	0.998809000000	1.930537000000	1.799549000000
C	-4.637396000000	0.568910000000	-0.363920000000
C	-4.256284000000	1.272804000000	1.977442000000
C	-5.781057000000	-0.116275000000	0.064370000000
C	-4.259820000000	0.603957000000	-1.824571000000
C	-6.164416000000	-0.106255000000	1.402928000000
H	-6.383361000000	-0.635631000000	-0.677092000000
C	-5.397949000000	0.565042000000	2.356475000000
H	-7.077833000000	-0.610925000000	1.708235000000
H	-5.708021000000	0.560531000000	3.395509000000
C	-3.395656000000	2.038455000000	2.959458000000
C	-3.815677000000	1.923395000000	4.419834000000
H	-3.127312000000	2.487952000000	5.051074000000
H	-4.818615000000	2.327836000000	4.580346000000
H	-3.804831000000	0.883789000000	4.759987000000
C	-4.800571000000	1.868431000000	-2.505161000000
H	-4.530214000000	1.874378000000	-3.563455000000
H	-5.890292000000	1.920580000000	-2.429261000000
H	-4.393392000000	2.771603000000	-2.041086000000
H	-2.358985000000	1.692320000000	2.848873000000
H	-3.389872000000	3.092569000000	2.657950000000
H	-3.173913000000	0.529438000000	-1.951052000000
H	-4.675559000000	-0.287273000000	-2.303365000000

O	-2.262405000000	-2.104506000000	-1.724590000000
P	-2.052443000000	-3.166299000000	-0.500425000000
K	-4.530199000000	-2.871142000000	1.196669000000
O	-0.733812000000	-3.910712000000	-0.777183000000
O	-3.294638000000	-4.065021000000	-0.659398000000
O	-2.035801000000	-2.437649000000	0.866865000000
K	-2.081559000000	-4.601826000000	-2.959964000000
K	0.364652000000	-3.278572000000	1.444328000000
H	-1.636418000000	-1.284080000000	-1.794153000000

INTa3

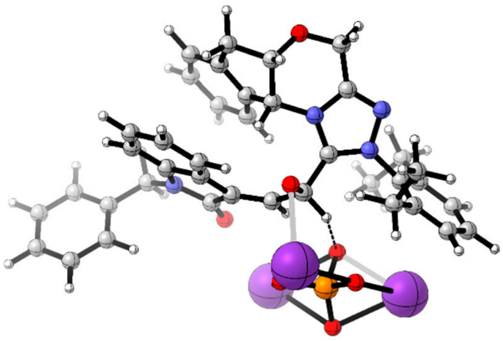

Zero-point correction= 0.700800 (Hartree/Particle)

Thermal correction to Energy= 0.749412

Thermal correction to Enthalpy= 0.750357

Thermal correction to Gibbs Free Energy= 0.616466

Sum of electronic and zero-point Energies= -4393.127559

Sum of electronic and thermal Energies= -4393.078946

Sum of electronic and thermal Enthalpies= -4393.078002

Sum of electronic and thermal Free Energies= -4393.211893

E(RM062X) = -4394.56524786

C	1.062437000000	0.223397000000	0.892796000000
C	1.606415000000	1.412318000000	0.061127000000
C	-0.061816000000	-0.433262000000	0.132585000000
C	-1.392826000000	-0.399516000000	0.333424000000
C	-2.323248000000	0.319248000000	1.215413000000
C	-3.598275000000	0.219500000000	0.621285000000
C	-4.714601000000	0.863628000000	1.129100000000
C	-4.547494000000	1.619510000000	2.291621000000
C	-3.306629000000	1.697930000000	2.921789000000
H	-5.403082000000	2.134145000000	2.717414000000
H	-5.683771000000	0.769886000000	0.649890000000
N	-3.528277000000	-0.602206000000	-0.509704000000
C	-2.187184000000	1.046417000000	2.393848000000
H	-1.206183000000	1.100001000000	2.857907000000
H	-3.207869000000	2.267256000000	3.841139000000
C	-2.239195000000	-1.042073000000	-0.711952000000
C	-4.631806000000	-0.903937000000	-1.385136000000
O	-1.904075000000	-1.795904000000	-1.626393000000
H	0.303906000000	-0.992869000000	-0.728098000000
O	0.822866000000	0.730306000000	2.120528000000
H	1.897101000000	-0.501196000000	0.815550000000
C	-5.723923000000	-1.720053000000	-0.722618000000
C	-5.428194000000	-2.615811000000	0.304755000000
C	-7.043686000000	-1.593252000000	-1.155976000000
C	-6.437902000000	-3.379440000000	0.882699000000
H	-4.404811000000	-2.702014000000	0.659687000000
C	-7.752450000000	-3.255245000000	0.440104000000
H	-6.197346000000	-4.068780000000	1.685909000000
C	-8.053916000000	-2.359633000000	-0.582049000000
H	-8.539305000000	-3.848445000000	0.894797000000
H	-9.077011000000	-2.250737000000	-0.927967000000
H	-7.280898000000	-0.886942000000	-1.948361000000
H	-4.198719000000	-1.452187000000	-2.228589000000
H	-5.047067000000	0.036820000000	-1.771491000000
N	2.864433000000	1.677144000000	-0.298151000000
N	0.967357000000	2.594348000000	-0.056527000000
N	3.057357000000	3.009010000000	-0.582855000000

C	3.993938000000	0.791967000000	-0.452909000000
C	1.881479000000	3.538861000000	-0.413986000000
C	1.499509000000	4.993349000000	-0.408674000000
C	-0.400584000000	2.894030000000	0.330454000000
O	0.103641000000	5.155656000000	-0.335814000000
H	2.000287000000	5.468573000000	0.448409000000
H	1.840572000000	5.483380000000	-1.322486000000
C	-0.518245000000	4.383930000000	0.688506000000
C	-2.027035000000	4.634811000000	0.604704000000
H	-0.073821000000	4.619518000000	1.665332000000
C	-1.466196000000	2.736649000000	-0.732435000000
H	-0.595145000000	2.263186000000	1.204254000000
C	-2.454643000000	3.695349000000	-0.502992000000
H	-2.498433000000	4.359361000000	1.555572000000
H	-2.238712000000	5.686106000000	0.395290000000
C	-3.612650000000	3.701451000000	-1.266709000000
C	-1.571561000000	1.829194000000	-1.775400000000
C	-3.750850000000	2.756604000000	-2.285515000000
H	-4.390421000000	4.438195000000	-1.089104000000
C	-2.730639000000	1.844452000000	-2.552678000000
H	-4.649017000000	2.752060000000	-2.895792000000
H	-2.837297000000	1.130816000000	-3.364271000000
H	-0.783499000000	1.110146000000	-1.979100000000
C	4.654933000000	0.327989000000	0.683946000000
C	4.429585000000	0.536484000000	-1.760460000000
C	5.848336000000	-0.375067000000	0.481869000000
C	4.207335000000	0.651188000000	2.085958000000
C	6.347479000000	-0.591626000000	-0.796689000000
H	6.389514000000	-0.735438000000	1.352391000000
C	5.628515000000	-0.158721000000	-1.912382000000
H	7.299520000000	-1.100073000000	-0.928885000000
H	6.006517000000	-0.340439000000	-2.915301000000
C	3.612999000000	0.929658000000	-2.966769000000

C	2.619865000000	-0.183232000000	-3.332458000000
H	1.927131000000	0.159544000000	-4.106706000000
H	3.157925000000	-1.055711000000	-3.718071000000
H	2.075295000000	-0.536737000000	-2.449587000000
C	4.866447000000	1.945137000000	2.579803000000
H	4.549808000000	2.176110000000	3.600393000000
H	5.957598000000	1.864497000000	2.567406000000
H	4.588308000000	2.787223000000	1.937757000000
H	3.078781000000	1.863269000000	-2.773136000000
H	4.290219000000	1.127491000000	-3.804247000000
H	3.119421000000	0.754285000000	2.152217000000
H	4.486477000000	-0.197536000000	2.717925000000
O	3.199529000000	-2.330193000000	1.418838000000
P	2.114368000000	-2.929601000000	0.459959000000
K	4.572707000000	-3.086945000000	-0.780666000000
O	0.693507000000	-2.813744000000	1.099984000000
O	2.434693000000	-4.383984000000	0.027723000000
O	2.136763000000	-2.078797000000	-0.882382000000
K	1.454582000000	-1.610899000000	3.170369000000
K	-0.000727000000	-3.715136000000	-1.245738000000

TSa2'

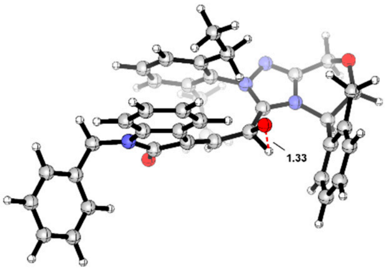

Frequency -1803.9460

Zero-point correction= 0.675709 (Hartree/Particle)

Thermal correction to Energy= 0.713180

Thermal correction to Enthalpy= 0.714124

Thermal correction to Gibbs Free Energy= 0.605354

Sum of electronic and zero-point Energies= -1950.973025

Sum of electronic and thermal Energies= -1950.935554

Sum of electronic and thermal Enthalpies= -1950.934610

Sum of electronic and thermal Free Energies= -1951.043380

E(RM062X) = -1952.11594317

C	-1.296711000000	0.447782000000	-0.592661000000
H	-1.688941000000	1.486200000000	-0.976280000000
C	0.045661000000	0.013824000000	-0.899804000000
C	1.244229000000	0.314989000000	-0.351090000000
C	1.702554000000	1.045948000000	0.830802000000
C	3.052985000000	0.681825000000	1.029471000000
C	3.808776000000	1.175957000000	2.080563000000
C	3.185960000000	2.064392000000	2.961116000000
C	1.856002000000	2.439933000000	2.779647000000
C	1.101962000000	1.934070000000	1.716761000000
H	1.397200000000	3.135544000000	3.475157000000
H	3.752504000000	2.470516000000	3.793362000000
H	4.850729000000	0.897623000000	2.202046000000
N	3.445169000000	-0.224963000000	0.042726000000
H	0.062228000000	2.205532000000	1.559886000000
C	2.413482000000	-0.456516000000	-0.852744000000
C	4.738708000000	-0.857972000000	-0.062173000000
O	2.497889000000	-1.193806000000	-1.827289000000
H	0.119380000000	-0.711626000000	-1.710113000000
C	-2.265340000000	-0.635635000000	-0.476085000000
O	-1.601416000000	1.515168000000	0.347936000000
N	-2.023743000000	-1.886274000000	-0.026987000000
N	-3.617948000000	-0.582029000000	-0.582428000000
C	-0.747114000000	-2.372615000000	0.439889000000
N	-3.166766000000	-2.609295000000	0.178080000000
C	-4.112038000000	-1.789967000000	-0.162949000000
C	-5.594786000000	-1.895330000000	-0.013293000000
C	-4.593033000000	0.387971000000	-1.121477000000
C	-0.051163000000	-3.289023000000	-0.347157000000
C	-0.249879000000	-1.819163000000	1.628307000000
O	-6.023664000000	-0.710026000000	0.629653000000
H	-6.087447000000	-2.028819000000	-0.987378000000
H	-5.847888000000	-2.737638000000	0.630102000000
C	-5.737624000000	0.486980000000	-0.071955000000
C	-5.248645000000	1.593968000000	0.875191000000
H	-6.634572000000	0.820828000000	-0.611792000000
C	-4.137735000000	1.816101000000	-1.218209000000
H	-4.931892000000	-0.018104000000	-2.080220000000
C	-4.519146000000	2.512287000000	-0.071837000000
H	-6.061864000000	2.066331000000	1.429675000000
H	-4.537894000000	1.160218000000	1.590118000000
C	-4.188126000000	3.852951000000	0.062670000000
C	-3.455189000000	2.430837000000	-2.259469000000
C	-3.472901000000	4.471937000000	-0.961001000000
H	-4.469952000000	4.408598000000	0.951403000000
C	-3.112756000000	3.773671000000	-2.114302000000
H	-3.196588000000	5.516906000000	-0.864361000000
H	-2.568084000000	4.280731000000	-2.903420000000
H	-3.176838000000	1.876378000000	-3.150963000000
C	1.240113000000	-3.624638000000	0.063322000000
C	-0.635919000000	-3.880319000000	-1.608996000000
C	1.787669000000	-3.062058000000	1.208623000000
H	1.821179000000	-4.315172000000	-0.540345000000
C	1.045807000000	-2.180584000000	1.991994000000
H	2.802437000000	-3.315591000000	1.500646000000
H	1.489974000000	-1.751340000000	2.883276000000
C	-1.077036000000	-0.833887000000	2.424989000000
C	-0.508015000000	-0.494799000000	3.798618000000
H	-1.195241000000	0.096903000000	1.847463000000

H	-2.087483000000	-1.251012000000	2.539209000000
C	-0.489287000000	-2.968327000000	-2.833734000000
H	-0.118389000000	-4.824582000000	-1.803856000000
H	-1.690517000000	-4.127604000000	-1.448336000000
H	-0.752185000000	-3.509305000000	-3.746557000000
H	-1.153452000000	-2.100333000000	-2.763981000000
H	0.537583000000	-2.599992000000	-2.920076000000
H	-1.194641000000	0.168199000000	4.330054000000
H	-0.360004000000	-1.392155000000	4.407839000000
H	0.448304000000	0.028554000000	3.708633000000
C	5.800703000000	0.066152000000	-0.617960000000
C	5.602544000000	0.653743000000	-1.870220000000
C	6.966304000000	0.344541000000	0.090483000000
C	6.559750000000	1.509637000000	-2.400593000000
H	4.692131000000	0.425045000000	-2.419558000000
C	7.725185000000	1.786798000000	-1.685937000000
H	6.399827000000	1.962536000000	-3.374083000000
C	7.928595000000	1.202137000000	-0.440827000000
H	8.470299000000	2.458658000000	-2.099996000000
H	8.831957000000	1.417137000000	0.121249000000
H	7.122719000000	-0.111190000000	1.065895000000
H	4.592212000000	-1.713676000000	-0.729484000000
H	5.036470000000	-1.232647000000	0.924827000000

TSa2''

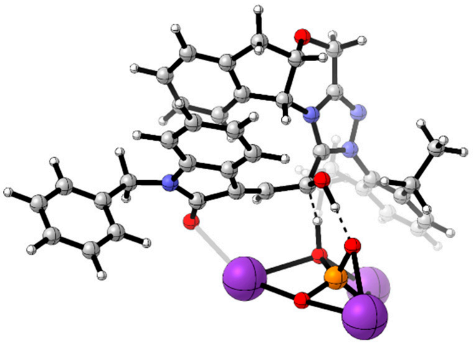

Frequency -1207.2946

Zero-point correction= 0.707381 (Hartree/Particle)

Thermal correction to Energy= 0.755954

Thermal correction to Enthalpy= 0.756898

Thermal correction to Gibbs Free Energy= 0.620996

Sum of electronic and zero-point Energies= -4393.578258

Sum of electronic and thermal Energies= -4393.529685

Sum of electronic and thermal Enthalpies= -4393.528740

Sum of electronic and thermal Free Energies= -4393.664643

E(RM062X) = -4395.01120218

O	-2.174008000000	-1.965707000000	0.653718000000
H	-1.768509000000	-0.889152000000	0.215840000000
C	-0.996573000000	0.144018000000	-0.322200000000
C	-1.539389000000	1.509249000000	-0.076492000000
C	0.187864000000	-0.141650000000	0.475690000000
O	-0.854262000000	0.021726000000	-1.710207000000
N	-2.774653000000	1.914761000000	0.221254000000
N	-0.871919000000	2.648186000000	-0.368543000000
C	1.459501000000	-0.458124000000	0.119885000000
H	0.000589000000	-0.198262000000	1.554723000000
C	2.323849000000	-0.123297000000	-1.021510000000
C	2.376174000000	-0.784729000000	1.246369000000
C	3.634949000000	-0.058941000000	-0.502628000000
C	2.112135000000	0.213232000000	-2.355693000000
C	4.715532000000	0.375829000000	-1.251562000000

N	3.643250000000	-0.478699000000	0.837612000000	C	1.632282000000	2.943737000000	0.040978000000
C	4.475439000000	0.734139000000	-2.579152000000	H	0.624594000000	1.876888000000	-1.543107000000
H	5.711998000000	0.421682000000	-0.825205000000	C	2.066289000000	4.258226000000	-1.910704000000
C	3.196835000000	0.639970000000	-3.126795000000	H	0.017530000000	3.962673000000	-2.719402000000
H	5.302576000000	1.072170000000	-3.194490000000	C	2.600469000000	3.720153000000	-0.600001000000
H	3.040015000000	0.892883000000	-4.170443000000	H	2.439509000000	3.674686000000	-2.760706000000
C	4.808571000000	-0.526220000000	1.692758000000	H	2.299247000000	5.312126000000	-2.078497000000
H	1.113798000000	0.137573000000	-2.772138000000	C	1.844718000000	2.440934000000	1.315246000000
O	2.059425000000	-1.251175000000	2.345047000000	C	3.837826000000	3.923907000000	-0.006272000000
C	5.876392000000	-1.484667000000	1.207877000000	C	4.079074000000	3.372255000000	1.252940000000
H	4.438770000000	-0.820319000000	2.680519000000	H	4.599373000000	4.517552000000	-0.502518000000
H	5.222728000000	0.487173000000	1.775564000000	C	3.083044000000	2.660606000000	1.920337000000
C	5.541549000000	-2.656095000000	0.529025000000	H	1.072654000000	1.887763000000	1.840664000000
C	7.218821000000	-1.203814000000	1.461014000000	H	5.039874000000	3.530271000000	1.732789000000
C	6.535691000000	-3.538020000000	0.117044000000	H	3.267777000000	2.271536000000	2.917085000000
H	4.498122000000	-2.867660000000	0.308966000000	C	-5.862321000000	-0.105743000000	-0.042904000000
C	8.214248000000	-2.087496000000	1.054409000000	C	-4.217998000000	0.493430000000	-1.863390000000
H	7.486783000000	-0.286946000000	1.980473000000	C	-5.530546000000	0.553264000000	2.263222000000
C	7.874086000000	-3.257197000000	0.380813000000	C	-3.456731000000	1.882685000000	2.953778000000
H	6.266060000000	-4.443393000000	-0.417229000000	C	-6.308726000000	-0.052679000000	1.274129000000
H	8.648715000000	-3.943789000000	0.055895000000	H	-6.463585000000	-0.588497000000	-0.808626000000
H	9.255317000000	-1.858372000000	1.257186000000	C	-4.691010000000	1.767078000000	-2.576001000000
N	-2.921954000000	3.270403000000	0.106666000000	H	-3.134062000000	0.383726000000	-1.945435000000
C	-3.930248000000	1.133833000000	0.589837000000	H	-4.637005000000	-0.393298000000	-2.347008000000
C	-1.746512000000	3.688053000000	-0.275382000000	H	-7.272875000000	-0.480606000000	1.536671000000
C	0.475461000000	2.764993000000	-0.920220000000	H	-5.887989000000	0.580701000000	3.286837000000
C	-4.649836000000	0.487153000000	-0.418884000000	C	-3.907092000000	1.742792000000	4.403092000000
C	-4.320856000000	1.169510000000	1.935781000000	H	-2.428466000000	1.510637000000	2.851546000000
C	-1.345090000000	5.057632000000	-0.754404000000	H	-3.416785000000	2.943933000000	2.681065000000
O	0.038748000000	5.125390000000	-0.994101000000	H	-3.205135000000	2.256495000000	5.062233000000
H	-1.922006000000	5.272727000000	-1.666219000000	H	-4.893276000000	2.186797000000	4.561844000000
H	-1.587303000000	5.814475000000	-0.006058000000	H	-3.948519000000	0.693921000000	4.712225000000
C	0.556559000000	4.047387000000	-1.766077000000	H	-4.377638000000	1.754686000000	-3.622184000000

H -5.780693000000 1.854141000000 -2.545365000000

H -4.275249000000 2.661507000000 -2.102114000000

P -2.248735000000 -2.957083000000 -0.628813000000

O -2.127534000000 -2.098218000000 -1.920718000000

H -1.339665000000 -0.881460000000 -1.919411000000

O -1.083603000000 -3.961557000000 -0.539069000000

O -3.613370000000 -3.674009000000 -0.612142000000

K -2.348239000000 -4.666999000000 -2.842545000000

K -4.567213000000 -2.566686000000 1.403182000000

K 0.225500000000 -3.107222000000 1.430066000000

TSa2

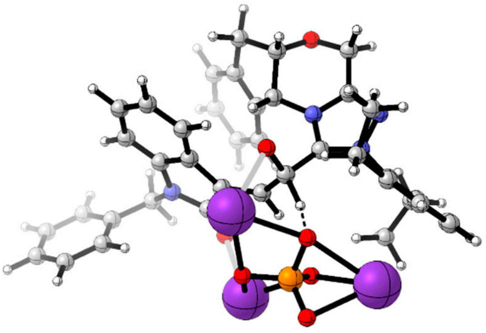

Frequency -989.9873

Zero-point correction= 0.694823 (Hartree/Particle)

Thermal correction to Energy= 0.743054

Thermal correction to Enthalpy= 0.743998

Thermal correction to Gibbs Free Energy= 0.609494

Sum of electronic and zero-point Energies= -4393.122670

Sum of electronic and thermal Energies= -4393.074439

Sum of electronic and thermal Enthalpies= -4393.073495

Sum of electronic and thermal Free Energies= -4393.207999

E(RM062X) = -4394.55281115

O 2.496013000000 -2.040816000000 1.231645000000

H 1.832435000000 -0.877926000000 0.787694000000

C 1.086196000000 0.146760000000 0.736761000000

C 1.806311000000 1.254548000000 -0.005218000000

C -0.056396000000 -0.293049000000 -0.068080000000

O 0.878652000000 0.646607000000 1.985211000000

N 3.091172000000 1.438478000000 -0.322959000000

N 1.253206000000 2.486855000000 -0.142175000000

C -1.388927000000 -0.287249000000 0.229835000000

H 0.218323000000 -0.743799000000 -1.025217000000

C -2.230989000000 0.364415000000 1.247159000000

C -2.315525000000 -0.740811000000 -0.829341000000

C -3.554227000000 0.337138000000 0.752605000000

C -2.002327000000 0.996242000000 2.470952000000

C -4.625014000000 0.914201000000 1.416449000000

N -3.580693000000 -0.329504000000 -0.473865000000

C -4.366771000000 1.543402000000 2.635093000000

H -5.627819000000 0.867528000000 1.003919000000

C -3.074391000000 1.576261000000 3.155356000000

H -5.183369000000 2.006821000000 3.179354000000

H -2.892260000000 2.065901000000 4.107199000000

C -4.732855000000 -0.449032000000 -1.330559000000

H -0.984753000000 1.048511000000 2.845599000000

O -2.064568000000 -1.341041000000 -1.881901000000

C -5.819527000000 -1.340452000000 -0.764401000000

H -4.357053000000 -0.854161000000 -2.276003000000

H -5.134976000000 0.553300000000 -1.530269000000

K 0.512209000000 -1.779860000000 2.904581000000

C -5.509830000000 -2.407625000000 0.079194000000

C -7.151888000000 -1.111020000000 -1.107767000000

C -6.517176000000 -3.237840000000 0.561913000000

H -4.475575000000 -2.575620000000 0.368090000000

C -8.160192000000 -1.942388000000 -0.629114000000

H -7.401198000000 -0.271973000000 -1.753167000000

C -7.844402000000 -3.009579000000 0.207330000000

H	-6.264445000000	-4.062739000000	1.220705000000
H	-8.628912000000	-3.656115000000	0.586939000000
H	-9.193164000000	-1.751071000000	-0.902377000000
N	3.381112000000	2.754441000000	-0.608110000000
C	4.204053000000	0.523671000000	-0.348716000000
C	2.241078000000	3.364106000000	-0.465704000000
C	-0.087400000000	2.887314000000	0.266350000000
C	4.774522000000	0.124483000000	0.864894000000
C	4.748058000000	0.208825000000	-1.599868000000
C	1.979813000000	4.844404000000	-0.446449000000
O	0.606750000000	5.120926000000	-0.323409000000
H	2.549274000000	5.270345000000	0.393608000000
H	2.328308000000	5.310560000000	-1.370210000000
C	-0.053798000000	4.365256000000	0.689514000000
C	-1.185633000000	2.882878000000	-0.776950000000
H	-0.324777000000	2.236551000000	1.119047000000
C	-1.530806000000	4.761920000000	0.659492000000
H	0.438211000000	4.514878000000	1.660264000000
C	-2.065955000000	3.927842000000	-0.483984000000
H	-2.004923000000	4.477235000000	1.606535000000
H	-1.647935000000	5.838432000000	0.514131000000
C	-1.426193000000	2.024478000000	-1.839957000000
C	-3.235628000000	4.087542000000	-1.212046000000
C	-3.503644000000	3.201023000000	-2.256387000000
H	-3.925102000000	4.894435000000	-0.981833000000
C	-2.599438000000	2.189856000000	-2.578038000000
H	-0.734763000000	1.226599000000	-2.092255000000
H	-4.414593000000	3.312893000000	-2.836748000000
H	-2.807366000000	1.512974000000	-3.401264000000
C	5.982828000000	-0.578139000000	0.793561000000
C	4.185361000000	0.501234000000	2.199364000000
C	5.964795000000	-0.479128000000	-1.619451000000
C	4.014722000000	0.506719000000	-2.883639000000
C	6.589516000000	-0.851003000000	-0.429915000000
H	6.456692000000	-0.889746000000	1.720010000000
C	4.614338000000	1.914424000000	2.614796000000
H	3.092618000000	0.434901000000	2.185608000000
H	4.533690000000	-0.229535000000	2.935508000000
H	7.547920000000	-1.361866000000	-0.457459000000
H	6.424230000000	-0.707390000000	-2.577837000000
C	3.117056000000	-0.680217000000	-3.267471000000
H	3.416603000000	1.415059000000	-2.778368000000
H	4.744799000000	0.701001000000	-3.675802000000
H	2.493393000000	-0.429417000000	-4.129956000000
H	3.732131000000	-1.543414000000	-3.548282000000
H	2.475075000000	-1.006337000000	-2.438509000000
H	4.181551000000	2.171452000000	3.584802000000
H	5.703101000000	1.996097000000	2.689065000000
H	4.275718000000	2.655893000000	1.884156000000
P	1.761422000000	-3.229440000000	0.417392000000
K	4.332923000000	-2.965172000000	-0.623910000000
O	0.306490000000	-3.356682000000	0.950091000000
O	2.630936000000	-4.479972000000	0.531948000000
O	1.732530000000	-2.715345000000	-1.074187000000
K	-0.644537000000	-3.490675000000	-1.397954000000

INTa4

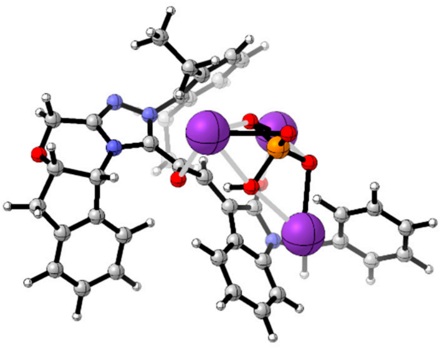

Zero-point correction= 0.701004 (Hartree/Particle)

Thermal correction to Energy= 0.749511

Thermal correction to Enthalpy= 0.750455

Thermal correction to Gibbs Free Energy= 0.617726

Sum of electronic and zero-point Energies= -4393.203174

Sum of electronic and thermal Energies= -4393.154667

Sum of electronic and thermal Enthalpies= -4393.153723

Sum of electronic and thermal Free Energies= -4393.286453

E(RM062X) = -4394.63754684

O	1.256800000000	0.397319000000	-2.522031000000
H	0.519929000000	0.535841000000	-1.886750000000
P	1.799225000000	-1.225399000000	-2.530657000000
O	-1.238364000000	0.669925000000	-1.396771000000
C	-1.229950000000	0.008240000000	-0.267186000000
C	-2.492028000000	-0.568140000000	0.160425000000
C	-0.258510000000	-0.048448000000	0.711366000000
N	-2.782756000000	-1.697924000000	0.848623000000
N	-3.709803000000	-0.005779000000	-0.090508000000
C	0.927219000000	0.714202000000	0.889277000000
H	-0.416552000000	-0.759600000000	1.516654000000
N	-4.133335000000	-1.836630000000	1.077310000000
C	-1.877018000000	-2.773036000000	1.128631000000
C	-4.656976000000	-0.801670000000	0.500349000000
C	-3.992625000000	1.289908000000	-0.714345000000
C	1.411779000000	1.954488000000	0.343137000000
C	1.859261000000	0.306811000000	1.903551000000
C	2.636835000000	2.254971000000	1.010172000000
C	0.958719000000	2.843449000000	-0.641803000000
N	2.866081000000	1.282083000000	1.968066000000
O	1.887139000000	-0.738740000000	2.591767000000
C	3.430011000000	3.341858000000	0.664851000000
C	1.749212000000	3.942235000000	-0.986388000000
H	0.017358000000	2.642751000000	-1.145041000000
C	2.975712000000	4.188794000000	-0.354801000000
H	4.374825000000	3.525645000000	1.168653000000
C	4.118974000000	1.060383000000	2.644482000000
H	3.568904000000	5.052651000000	-0.637179000000
H	1.402408000000	4.621804000000	-1.759152000000
C	5.221498000000	0.633599000000	1.691979000000
H	3.923904000000	0.269409000000	3.375949000000
H	4.416454000000	1.962481000000	3.191815000000
C	4.907734000000	-0.111860000000	0.553407000000
C	6.555163000000	0.957240000000	1.936087000000
C	5.901040000000	-0.528226000000	-0.330148000000
H	3.875712000000	-0.360890000000	0.319167000000
C	7.555716000000	0.539295000000	1.059700000000
H	6.814649000000	1.543031000000	2.814915000000
C	7.233375000000	-0.201468000000	-0.075616000000
H	5.601804000000	-1.105960000000	-1.201401000000
H	8.590294000000	0.799147000000	1.262466000000
H	8.015741000000	-0.523759000000	-0.755609000000
C	-1.397972000000	-3.519804000000	0.042956000000
C	-1.518562000000	-3.030076000000	2.458066000000
C	-6.103234000000	-0.441466000000	0.375186000000
C	-5.520743000000	1.437307000000	-0.920508000000
C	-3.634521000000	2.522280000000	0.094236000000
H	-3.443042000000	1.304496000000	-1.659952000000
C	-0.488606000000	-4.543677000000	0.318100000000
C	-1.912565000000	-3.318378000000	-1.360462000000
C	-0.598039000000	-4.057185000000	2.681465000000
C	-2.108597000000	-2.260213000000	3.618910000000
O	-6.230014000000	0.948876000000	0.209078000000
H	-6.541137000000	-0.979900000000	-0.480506000000
H	-6.640860000000	-0.727048000000	1.280064000000
C	-5.746493000000	2.950003000000	-0.950113000000
H	-5.867816000000	0.915915000000	-1.825014000000

C	-4.640169000000	3.477044000000	-0.065803000000
C	-2.485993000000	2.806717000000	0.825629000000
H	-5.645526000000	3.329204000000	-1.973754000000
H	-6.752640000000	3.187340000000	-0.595666000000
C	-4.525065000000	4.729019000000	0.523570000000
C	-2.376685000000	4.063479000000	1.420725000000
H	-1.688187000000	2.080647000000	0.939969000000
C	-3.385366000000	5.014731000000	1.273804000000
H	-5.309352000000	5.470882000000	0.404474000000
H	-3.280472000000	5.987306000000	1.744202000000
H	-1.487235000000	4.300756000000	1.995242000000
C	-0.081970000000	-4.803593000000	1.624987000000
H	-0.102450000000	-5.134162000000	-0.508288000000
C	-3.252430000000	-4.035383000000	-1.562413000000
H	-2.012251000000	-2.247333000000	-1.559381000000
H	-1.149229000000	-3.677849000000	-2.054133000000
H	-0.292690000000	-4.273594000000	3.700854000000
C	-1.559673000000	-0.838056000000	3.798495000000
H	-3.196911000000	-2.221559000000	3.502876000000
H	-1.908001000000	-2.836975000000	4.527038000000
H	0.625997000000	-5.603106000000	1.823248000000
H	-3.627059000000	-3.888201000000	-2.580038000000
H	-3.143468000000	-5.110868000000	-1.396501000000
H	-4.005702000000	-3.665964000000	-0.859675000000
H	-1.908611000000	-0.427651000000	4.749586000000
H	-0.464790000000	-0.830129000000	3.790995000000
H	-1.904929000000	-0.169508000000	3.004265000000
O	0.644683000000	-1.976409000000	-1.834812000000
O	3.077549000000	-1.179273000000	-1.674419000000
O	1.904828000000	-1.501134000000	-4.011519000000
K	-0.781075000000	-0.790661000000	-3.716117000000
K	3.521392000000	1.376505000000	-1.847169000000
K	1.804922000000	-2.306542000000	0.408915000000

TSa3

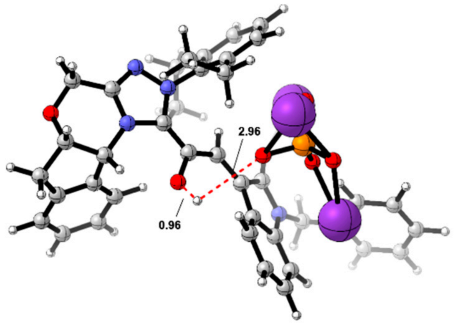

Frequency -326.9591

Zero-point correction= 0.699729 (Hartree/Particle)

Thermal correction to Energy= 0.747974

Thermal correction to Enthalpy= 0.748918

Thermal correction to Gibbs Free Energy= 0.617372

Sum of electronic and zero-point Energies= -4393.149330

Sum of electronic and thermal Energies= -4393.101086

Sum of electronic and thermal Enthalpies= -4393.100142

Sum of electronic and thermal Free Energies= -4393.231688

E(RM062X) = -4394.58865475

O	0.792310000000	-0.886263000000	-2.035483000000
H	-0.811734000000	1.552452000000	-1.543750000000
P	2.115484000000	-1.571066000000	-1.554556000000
O	-1.664402000000	1.149044000000	-1.346406000000
C	-1.499858000000	0.285673000000	-0.286942000000
C	-2.659040000000	-0.404269000000	0.060102000000
C	-0.368784000000	0.269159000000	0.524587000000
N	-2.822806000000	-1.479975000000	0.890849000000
N	-3.943475000000	-0.099947000000	-0.350252000000
C	0.798073000000	0.996912000000	0.442714000000
H	-0.456597000000	-0.315021000000	1.438463000000
N	-4.156621000000	-1.808350000000	1.047953000000

C	-1.854478000000	-2.425461000000	1.360149000000
C	-4.786870000000	-0.969195000000	0.299861000000
C	-4.428545000000	1.075800000000	-1.075482000000
C	1.445272000000	1.831806000000	-0.552892000000
C	1.673871000000	1.000341000000	1.611570000000
C	2.559293000000	2.438567000000	0.084473000000
C	1.293963000000	2.025711000000	-1.927344000000
N	2.658423000000	1.954629000000	1.383033000000
O	1.611810000000	0.312277000000	2.639994000000
C	3.413395000000	3.314920000000	-0.569513000000
C	2.135780000000	2.923581000000	-2.596294000000
H	0.636647000000	1.368884000000	-2.491766000000
C	3.169032000000	3.580512000000	-1.924767000000
H	4.258321000000	3.760088000000	-0.052969000000
C	3.748906000000	2.214192000000	2.291310000000
H	3.808510000000	4.277829000000	-2.457226000000
H	1.998255000000	3.085541000000	-3.661214000000
C	5.092514000000	1.738796000000	1.769997000000
H	3.489256000000	1.686517000000	3.214902000000
H	3.794445000000	3.287065000000	2.517074000000
C	5.178102000000	0.555752000000	1.029880000000
C	6.251541000000	2.464960000000	2.038644000000
C	6.417994000000	0.113079000000	0.571102000000
H	4.275557000000	-0.003043000000	0.772941000000
C	7.490190000000	2.018273000000	1.580353000000
H	6.187154000000	3.389516000000	2.608291000000
C	7.577051000000	0.840477000000	0.843588000000
H	6.472289000000	-0.814658000000	0.005779000000
H	8.385256000000	2.592956000000	1.797710000000
H	8.540621000000	0.489693000000	0.487124000000
C	-1.181023000000	-3.241946000000	0.438627000000
C	-1.695613000000	-2.546422000000	2.750980000000
C	-6.255356000000	-0.920272000000	0.026415000000

C	-5.935018000000	0.917151000000	-1.400510000000
C	-4.390602000000	2.387869000000	-0.314869000000
H	-3.853425000000	1.157154000000	-2.000467000000
C	-0.325534000000	-4.220036000000	0.963079000000
C	-1.313862000000	-3.112595000000	-1.059752000000
C	-0.839452000000	-3.542115000000	3.222040000000
C	-2.421038000000	-1.636866000000	3.716587000000
O	-6.642835000000	0.395199000000	-0.287748000000
H	-6.489984000000	-1.603807000000	-0.805203000000
H	-6.809220000000	-1.238316000000	0.910105000000
C	-6.418374000000	2.357949000000	-1.579620000000
H	-6.099720000000	0.276065000000	-2.278860000000
C	-5.532701000000	3.127179000000	-0.626537000000
C	-3.419730000000	2.894830000000	0.540479000000
H	-6.262762000000	2.684090000000	-2.614720000000
H	-7.484731000000	2.430862000000	-1.352603000000
C	-5.725813000000	4.389647000000	-0.082473000000
C	-3.622657000000	4.158340000000	1.095927000000
H	-2.524479000000	2.328233000000	0.775186000000
C	-4.761979000000	4.899728000000	0.786414000000
H	-6.616332000000	4.964412000000	-0.319643000000
H	-4.901570000000	5.880978000000	1.228915000000
H	-2.881544000000	4.567589000000	1.774625000000
C	-0.167898000000	-4.382784000000	2.335047000000
H	0.245249000000	-4.824155000000	0.265393000000
C	-2.735837000000	-3.283282000000	-1.610456000000
H	-0.846205000000	-2.165231000000	-1.380062000000
H	-0.673325000000	-3.897526000000	-1.472179000000
H	-0.711313000000	-3.662478000000	4.294132000000
C	-1.945407000000	-0.177959000000	3.675493000000
H	-3.496305000000	-1.677342000000	3.513614000000
H	-2.273126000000	-2.036018000000	4.724862000000
H	0.487414000000	-5.161305000000	2.715749000000

H -2.696249000000 -3.426963000000 -2.695445000000

H -3.233786000000 -4.155443000000 -1.175642000000

H -3.371279000000 -2.412429000000 -1.434720000000

H -2.392800000000 0.384747000000 4.499040000000

H -0.855781000000 -0.107150000000 3.758451000000

H -2.241466000000 0.314520000000 2.744030000000

O 1.745292000000 -3.004941000000 -1.041147000000

O 2.843055000000 -0.773672000000 -0.416569000000

O 3.072635000000 -1.657277000000 -2.796785000000

K 1.053325000000 -3.005322000000 -3.645876000000

K 4.164311000000 0.534283000000 -2.102856000000

K 1.819411000000 -2.128949000000 1.420768000000

INTa5

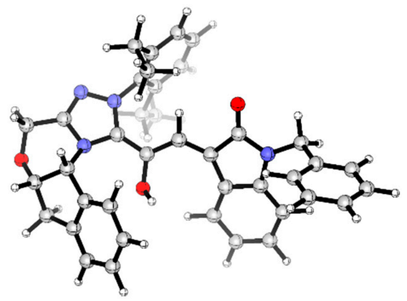

Zero-point correction= 0.679664 (Hartree/Particle)

Thermal correction to Energy= 0.718010

Thermal correction to Enthalpy= 0.718954

Thermal correction to Gibbs Free Energy= 0.605370

Sum of electronic and zero-point Energies= -1951.063426

Sum of electronic and thermal Energies= -1951.025080

Sum of electronic and thermal Enthalpies= -1951.024135

Sum of electronic and thermal Free Energies= -1951.137720

E(RM062X) = -1952.20923259

C -1.075031000000 0.132939000000 0.198526000000

H -0.463079000000 1.939245000000 0.476251000000

C 0.105952000000 -0.602094000000 0.004315000000

C 1.384411000000 -0.364285000000 0.434158000000

C 2.041566000000 0.536143000000 1.378987000000

C 3.430048000000 0.270901000000 1.288641000000

C 4.371083000000 0.934356000000 2.059272000000

C 3.909824000000 1.886325000000 2.971781000000

C 2.547287000000 2.141898000000 3.104633000000

C 1.607852000000 1.468215000000 2.320409000000

H 2.205097000000 2.869991000000 3.833142000000

H 4.624171000000 2.422883000000 3.587926000000

H 5.430177000000 0.723229000000 1.949395000000

N 3.651976000000 -0.740215000000 0.355550000000

H 0.548641000000 1.653579000000 2.455764000000

C 2.448067000000 -1.187481000000 -0.182701000000

C 4.932549000000 -1.303103000000 0.014707000000

O 2.355083000000 -2.073666000000 -1.021853000000

H 0.041853000000 -1.458002000000 -0.662805000000

C -2.322626000000 -0.312751000000 -0.236943000000

O -1.086043000000 1.329170000000 0.896164000000

N -2.759066000000 -1.604424000000 -0.395980000000

N -3.459361000000 0.451270000000 -0.443166000000

C -2.037488000000 -2.802051000000 -0.090170000000

N -4.119854000000 -1.668898000000 -0.645199000000

C -4.493698000000 -0.438438000000 -0.669855000000

C -5.846322000000 0.159000000000 -0.915470000000

C -3.638465000000 1.731807000000 -1.160916000000

C -1.569264000000 -3.583847000000 -1.148864000000

C -1.824668000000 -3.123272000000 1.257728000000

O -5.916883000000 1.379401000000 -0.206980000000

H -6.012331000000 0.311584000000 -1.993074000000

H -6.628356000000 -0.498816000000 -0.535039000000

C -4.968079000000 2.338903000000 -0.630592000000

C -4.524025000000 3.227501000000 0.543199000000

H	-5.391740000000	2.957172000000	-1.435970000000
C	-2.656138000000	2.829900000000	-0.882900000000
H	-3.682432000000	1.494935000000	-2.231912000000
C	-3.157627000000	3.687988000000	0.095042000000
H	-5.223679000000	4.041158000000	0.745827000000
H	-4.448804000000	2.602575000000	1.440738000000
C	-2.398188000000	4.774771000000	0.511739000000
C	-1.431239000000	3.052976000000	-1.498581000000
C	-1.148439000000	4.986036000000	-0.070730000000
H	-2.768354000000	5.451050000000	1.276154000000
C	-0.669057000000	4.140476000000	-1.074696000000
H	-0.544159000000	5.828839000000	0.248883000000
H	0.297733000000	4.334090000000	-1.527352000000
H	-1.065733000000	2.379036000000	-2.268769000000
C	-0.827283000000	-4.721557000000	-0.829685000000
C	-1.813746000000	-3.197461000000	-2.588903000000
C	-0.575468000000	-5.053575000000	0.494973000000
H	-0.438501000000	-5.340908000000	-1.632469000000
C	-1.075108000000	-4.267109000000	1.528799000000
H	0.012190000000	-5.935601000000	0.728109000000
H	-0.879903000000	-4.550469000000	2.557108000000
C	-2.395185000000	-2.237799000000	2.343713000000
C	-2.295683000000	-2.803712000000	3.756026000000
H	-1.883806000000	-1.267276000000	2.306237000000
H	-3.445633000000	-2.033057000000	2.101589000000
C	-0.886703000000	-2.074562000000	-3.074304000000
H	-1.658245000000	-4.086187000000	-3.208539000000
H	-2.859448000000	-2.898513000000	-2.718267000000
H	-0.995266000000	-1.929084000000	-4.152538000000
H	-1.123802000000	-1.125020000000	-2.583049000000
H	0.160374000000	-2.305562000000	-2.854577000000
H	-2.778545000000	-2.127748000000	4.464989000000
H	-2.783530000000	-3.779918000000	3.831716000000
H	-1.253656000000	-2.917847000000	4.067076000000
C	5.868219000000	-0.312100000000	-0.649028000000
C	5.372716000000	0.681212000000	-1.494250000000
C	7.243632000000	-0.396139000000	-0.434734000000
C	6.240784000000	1.570130000000	-2.119476000000
H	4.299939000000	0.756061000000	-1.650129000000
C	7.614293000000	1.477276000000	-1.906371000000
H	5.844084000000	2.340150000000	-2.773841000000
C	8.114657000000	0.490387000000	-1.062286000000
H	8.290424000000	2.174304000000	-2.391132000000
H	9.182903000000	0.415055000000	-0.884533000000
H	7.634866000000	-1.159967000000	0.233234000000
H	4.712275000000	-2.136082000000	-0.661761000000
H	5.410706000000	-1.715049000000	0.913386000000

----

^{rr}INTa6

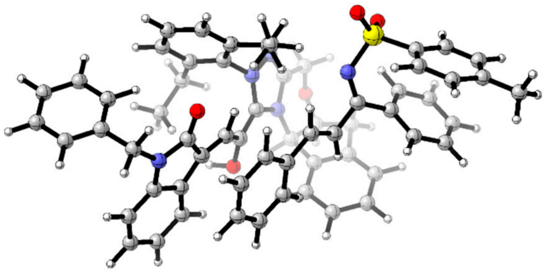

Zero-point correction=	1.041402 (Hartree/Particle)
Thermal correction to Energy=	1.103227
Thermal correction to Enthalpy=	1.104171
Thermal correction to Gibbs Free Energy=	0.941428
Sum of electronic and zero-point Energies=	-3403.383800
Sum of electronic and thermal Energies=	-3403.321975
Sum of electronic and thermal Enthalpies=	-3403.321031
Sum of electronic and thermal Free Energies=	-3403.483774

E(RM062X) = -3405.17363735

C	3.080635000000	0.089601000000	0.997775000000
C	-0.949278000000	-1.299765000000	1.360935000000
C	1.986237000000	0.445754000000	0.251032000000
C	1.347456000000	1.660374000000	0.008810000000
C	4.028515000000	0.763711000000	1.873042000000
C	5.095331000000	-0.145290000000	2.087161000000
C	6.200271000000	0.174133000000	2.859517000000
C	6.231521000000	1.431239000000	3.468751000000
C	5.174919000000	2.324818000000	3.309419000000
C	4.070250000000	1.997829000000	2.518431000000
H	5.208029000000	3.288970000000	3.806896000000
H	7.086445000000	1.706983000000	4.077615000000
H	7.019128000000	-0.527929000000	2.981515000000
N	4.812457000000	-1.347092000000	1.443962000000
H	3.240419000000	2.689413000000	2.422170000000
C	3.599615000000	-1.264640000000	0.764154000000
C	5.736871000000	-2.437121000000	1.231322000000
O	3.137403000000	-2.178612000000	0.083614000000
H	1.586979000000	-0.402672000000	-0.303809000000
C	0.481194000000	1.786642000000	-1.088007000000
O	1.674718000000	2.849585000000	0.668386000000
H	2.531358000000	3.143149000000	0.326225000000
N	0.461064000000	1.022912000000	-2.217066000000
N	-0.461325000000	2.758724000000	-1.315665000000
C	1.514535000000	0.157892000000	-2.681996000000
N	-0.446401000000	1.493275000000	-3.137757000000
C	-0.969070000000	2.531956000000	-2.578253000000
C	-1.971434000000	3.459062000000	-3.192394000000
C	-0.885809000000	3.858681000000	-0.431208000000
C	2.727626000000	0.738729000000	-3.069795000000
C	1.285473000000	-1.223245000000	-2.707158000000
O	-1.956877000000	4.697082000000	-2.517091000000
H	-1.691569000000	3.652528000000	-4.229127000000
H	-2.969510000000	3.002317000000	-3.192079000000
C	-2.079003000000	4.583583000000	-1.118496000000
C	-3.342503000000	3.866110000000	-0.602877000000
H	-2.080918000000	5.610346000000	-0.744148000000
C	-1.515120000000	3.409937000000	0.875798000000
H	-0.024808000000	4.511744000000	-0.280107000000
C	-2.910044000000	3.421160000000	0.767123000000
H	-4.215174000000	4.523709000000	-0.593575000000
H	-3.595304000000	2.999426000000	-1.221701000000
C	-3.709785000000	3.098468000000	1.856577000000
C	-0.901920000000	3.122091000000	2.091625000000
C	-3.098761000000	2.793780000000	3.071131000000
H	-4.791871000000	3.091438000000	1.763265000000
C	-1.709299000000	2.822930000000	3.190347000000
H	-3.710351000000	2.546031000000	3.933311000000
H	-1.247620000000	2.610571000000	4.150268000000
H	0.178753000000	3.137941000000	2.178199000000
C	3.733379000000	-0.116201000000	-3.523366000000
C	3.016770000000	2.218310000000	-2.959049000000
C	3.532158000000	-1.489253000000	-3.574811000000
H	4.683159000000	0.310304000000	-3.836883000000
C	2.323087000000	-2.036546000000	-3.160418000000
H	4.328702000000	-2.141282000000	-3.921619000000
H	2.191776000000	-3.112250000000	-3.166588000000
C	-0.021885000000	-1.787076000000	-2.204354000000
C	0.078678000000	-3.202877000000	-1.646894000000
H	-0.409670000000	-1.126321000000	-1.421225000000
H	-0.775100000000	-1.749252000000	-3.002487000000
C	4.074652000000	2.507586000000	-1.884335000000
H	3.378639000000	2.581662000000	-3.927338000000
H	2.100985000000	2.777957000000	-2.747077000000
H	4.205108000000	3.584675000000	-1.740233000000

H	3.809330000000	2.036839000000	-0.931774000000
H	5.045061000000	2.091474000000	-2.168710000000
H	-0.885310000000	-3.486297000000	-1.216405000000
H	0.305966000000	-3.936010000000	-2.425958000000
H	0.867566000000	-3.251399000000	-0.887513000000
C	6.737820000000	-2.119205000000	0.139138000000
C	6.265212000000	-1.762025000000	-1.128508000000
C	8.109602000000	-2.157012000000	0.370807000000
C	7.160618000000	-1.448652000000	-2.142996000000
H	5.192401000000	-1.739491000000	-1.309491000000
C	8.535236000000	-1.489285000000	-1.906865000000
H	6.786108000000	-1.173769000000	-3.125377000000
C	9.008343000000	-1.845022000000	-0.649020000000
H	9.232773000000	-1.243438000000	-2.701408000000
H	10.076220000000	-1.874443000000	-0.456380000000
H	8.479876000000	-2.430613000000	1.356316000000
H	5.121446000000	-3.294775000000	0.943060000000
H	6.247587000000	-2.675869000000	2.170729000000
C	-1.946314000000	-0.405075000000	1.196209000000
C	-3.024725000000	-0.668485000000	0.234475000000
N	-2.779688000000	-1.503351000000	-0.720990000000
C	0.158564000000	-1.247909000000	2.309021000000
C	0.394638000000	-0.146686000000	3.148115000000
C	1.485999000000	-0.136628000000	4.000379000000
C	2.358286000000	-1.228243000000	4.035908000000
C	2.126625000000	-2.329494000000	3.219853000000
C	1.035971000000	-2.336350000000	2.355726000000
C	-4.322416000000	0.026373000000	0.490114000000
C	-4.928564000000	-0.111216000000	1.742445000000
C	-6.158041000000	0.488864000000	1.991499000000
C	-6.766107000000	1.267214000000	1.007045000000
C	-6.144266000000	1.437858000000	-0.227973000000
C	-4.932182000000	0.806348000000	-0.495025000000
S	-3.931419000000	-2.136639000000	-1.771324000000
C	-5.293361000000	-2.600471000000	-0.722253000000
C	-6.531582000000	-1.996268000000	-0.888088000000
C	-7.559497000000	-2.320091000000	-0.007480000000
C	-7.358164000000	-3.233593000000	1.028448000000
C	-6.102134000000	-3.838596000000	1.160448000000
C	-5.067883000000	-3.534233000000	0.286978000000
O	-4.387589000000	-1.113734000000	-2.708150000000
O	-3.313463000000	-3.352625000000	-2.270479000000
H	-0.980561000000	-2.186153000000	0.726890000000
H	-2.038351000000	0.490981000000	1.805371000000
H	-0.267996000000	0.709917000000	3.100103000000
H	1.684288000000	0.731765000000	4.620813000000
H	3.230804000000	-1.201916000000	4.681462000000
H	2.814846000000	-3.168651000000	3.223367000000
H	0.880681000000	-3.175091000000	1.683392000000
H	-4.442310000000	-0.710818000000	2.506915000000
H	-6.640397000000	0.351572000000	2.953979000000
H	-7.722636000000	1.741608000000	1.203341000000
H	-6.612506000000	2.046962000000	-0.995169000000
H	-4.471858000000	0.883371000000	-1.474337000000
H	-6.669167000000	-1.270209000000	-1.682205000000
H	-8.530391000000	-1.845896000000	-0.120837000000
H	-5.938222000000	-4.558753000000	1.957439000000
H	-4.093597000000	-4.004245000000	0.384043000000
C	-8.472871000000	-3.586216000000	1.978255000000
H	-8.887057000000	-4.571180000000	1.740180000000
H	-8.113952000000	-3.623475000000	3.010200000000
H	-9.286210000000	-2.859650000000	1.922885000000

----

^{rs}INTa6

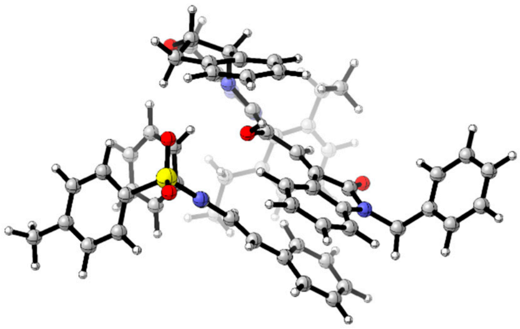

Zero-point correction= 1.041353 (Hartree/Particle)

Thermal correction to Energy= 1.103178

Thermal correction to Enthalpy= 1.104123

Thermal correction to Gibbs Free Energy= 0.941620

Sum of electronic and zero-point Energies= -3403.386460

Sum of electronic and thermal Energies= -3403.324635

Sum of electronic and thermal Enthalpies= -3403.323691

Sum of electronic and thermal Free Energies= -3403.486193

E(RM062X) = -3405.17385564

C	-2.309728000000	-0.043763000000	-0.143627000000
C	-0.417168000000	-2.533941000000	1.285376000000
C	-1.479865000000	1.013123000000	0.115345000000
C	-0.208930000000	1.336502000000	-0.397403000000
C	-2.270807000000	-1.295353000000	-0.900878000000
C	-3.531346000000	-1.915261000000	-0.717238000000
C	-3.850073000000	-3.142379000000	-1.276073000000
C	-2.864688000000	-3.797091000000	-2.020379000000
C	-1.603284000000	-3.229216000000	-2.184138000000
C	-1.302173000000	-1.982611000000	-1.630751000000
H	-0.828993000000	-3.758300000000	-2.730357000000
H	-3.086001000000	-4.762321000000	-2.465007000000
H	-4.832835000000	-3.580703000000	-1.134365000000
N	-4.317143000000	-1.134274000000	0.126524000000
H	-0.293451000000	-1.599981000000	-1.720346000000
C	-3.647932000000	0.022350000000	0.490862000000
C	-5.665626000000	-1.433001000000	0.550324000000
O	-4.129376000000	0.903895000000	1.197134000000
H	-1.940054000000	1.740018000000	0.779827000000
C	0.471828000000	2.519052000000	-0.104207000000
O	0.455163000000	0.468844000000	-1.244362000000
H	-0.034188000000	0.376106000000	-2.074553000000
N	0.409490000000	3.321138000000	1.012668000000
N	1.535620000000	3.042622000000	-0.829412000000
C	-0.514403000000	3.205369000000	2.095330000000
N	1.445215000000	4.240017000000	1.052997000000
C	2.082108000000	4.044883000000	-0.047676000000
C	3.277846000000	4.748719000000	-0.619811000000
C	1.632948000000	3.353428000000	-2.278845000000
C	-1.717577000000	3.913553000000	2.022944000000
C	-0.207048000000	2.327102000000	3.143897000000
O	3.940493000000	3.852429000000	-1.490228000000
H	2.972079000000	5.663265000000	-1.150616000000
H	3.974965000000	5.026057000000	0.172362000000
C	3.150025000000	3.445869000000	-2.596704000000
C	3.532924000000	2.031481000000	-3.071322000000
H	3.270463000000	4.167252000000	-3.418104000000
C	1.163078000000	2.300311000000	-3.233689000000
H	1.112040000000	4.307338000000	-2.435813000000
C	2.236991000000	1.517844000000	-3.653020000000
H	4.353374000000	2.043918000000	-3.792727000000
H	3.846641000000	1.415967000000	-2.220522000000
C	2.008671000000	0.416750000000	-4.471351000000
C	-0.137245000000	2.064008000000	-3.668440000000
C	0.703139000000	0.137827000000	-4.870665000000
H	2.825892000000	-0.235107000000	-4.759367000000
C	-0.362730000000	0.961193000000	-4.490312000000
H	0.507753000000	-0.731349000000	-5.490129000000
H	-1.368413000000	0.731116000000	-4.826033000000

H	-0.959218000000	2.695947000000	-3.342586000000
C	-2.668366000000	3.679041000000	3.017673000000
C	-2.029971000000	4.858162000000	0.886163000000
C	-2.412025000000	2.780166000000	4.042948000000
H	-3.622192000000	4.196096000000	2.968592000000
C	-1.186139000000	2.122445000000	4.116560000000
H	-3.167759000000	2.596031000000	4.799974000000
H	-0.997019000000	1.438031000000	4.937028000000
C	1.147934000000	1.656688000000	3.185388000000
C	1.334372000000	0.670139000000	4.332611000000
H	1.323951000000	1.155537000000	2.224584000000
H	1.918432000000	2.436589000000	3.238519000000
C	-2.712060000000	4.160538000000	-0.299694000000
H	-2.692977000000	5.638621000000	1.273027000000
H	-1.113601000000	5.356040000000	0.553086000000
H	-3.094493000000	4.898538000000	-1.009890000000
H	-2.011829000000	3.508474000000	-0.829370000000
H	-3.542708000000	3.534588000000	0.042388000000
H	2.331265000000	0.229402000000	4.285427000000
H	1.226136000000	1.162595000000	5.303520000000
H	0.602622000000	-0.144788000000	4.287738000000
C	-6.696255000000	-1.092444000000	-0.503505000000
C	-6.804048000000	0.229620000000	-0.944670000000
C	-7.533334000000	-2.063673000000	-1.046032000000
C	-7.736730000000	0.568872000000	-1.916575000000
H	-6.148439000000	0.980633000000	-0.509308000000
C	-8.572084000000	-0.408671000000	-2.457943000000
H	-7.816770000000	1.597715000000	-2.253743000000
C	-8.470271000000	-1.724695000000	-2.021118000000
H	-9.299574000000	-0.142673000000	-3.218479000000
H	-9.116798000000	-2.489453000000	-2.440051000000
H	-7.452667000000	-3.092903000000	-0.703193000000
H	-5.830944000000	-0.834947000000	1.452366000000
H	-5.723850000000	-2.491536000000	0.827785000000
C	0.572571000000	-1.626695000000	1.363148000000
C	1.753345000000	-1.636230000000	0.499311000000
N	1.754686000000	-2.394906000000	-0.546044000000
C	-1.656559000000	-2.485551000000	2.063544000000
C	-2.038241000000	-1.344348000000	2.786214000000
C	-3.243356000000	-1.313028000000	3.474035000000
C	-4.087866000000	-2.422737000000	3.452675000000
C	-3.727261000000	-3.557693000000	2.728266000000
C	-2.523925000000	-3.584782000000	2.032398000000
C	2.854810000000	-0.730123000000	0.951720000000
C	3.164933000000	0.426245000000	0.234346000000
C	4.157087000000	1.285472000000	0.701914000000
C	4.852530000000	0.979573000000	1.869928000000
C	4.542583000000	-0.176704000000	2.584524000000
C	3.529219000000	-1.020183000000	2.139901000000
S	3.066870000000	-2.488658000000	-1.596107000000
C	4.486500000000	-2.779088000000	-0.559958000000
C	4.520097000000	-3.939740000000	0.205519000000
C	5.583408000000	-4.136933000000	1.078736000000
C	6.612272000000	-3.196310000000	1.181260000000
C	6.563035000000	-2.052693000000	0.377778000000
C	5.504497000000	-1.835328000000	-0.494991000000
C	7.767055000000	-3.410107000000	2.124686000000
O	2.826910000000	-3.691491000000	-2.374597000000
O	3.258290000000	-1.211025000000	-2.283205000000
H	-0.307385000000	-3.355829000000	0.579219000000
H	0.527383000000	-0.815106000000	2.081878000000
H	-1.419529000000	-0.450873000000	2.764209000000
H	-3.537780000000	-0.405392000000	3.990700000000
H	-5.034664000000	-2.394925000000	3.983621000000
H	-4.389306000000	-4.417539000000	2.696898000000
H	-2.252486000000	-4.454511000000	1.440239000000

H 2.617143000000 0.634493000000 -0.677421000000

H 4.387994000000 2.184134000000 0.135378000000

H 5.634580000000 1.642765000000 2.226514000000

H 5.084311000000 -0.418399000000 3.493961000000

H 3.273582000000 -1.916430000000 2.699131000000

H 3.720530000000 -4.669496000000 0.120089000000

H 5.616272000000 -5.033889000000 1.690681000000

H 7.356397000000 -1.313600000000 0.451953000000

H 5.438318000000 -0.936933000000 -1.099613000000

H 8.667389000000 -3.703645000000 1.575592000000

H 7.547946000000 -4.195056000000 2.851418000000

H 8.001491000000 -2.492161000000 2.670673000000

----

^{sr}INTa6

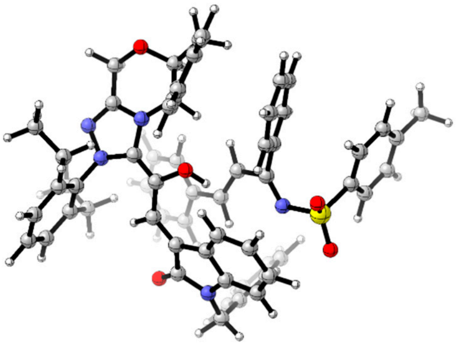

Zero-point correction= 1.040393 (Hartree/Particle)

Thermal correction to Energy= 1.102538

Thermal correction to Enthalpy= 1.103482

Thermal correction to Gibbs Free Energy= 0.940462

Sum of electronic and zero-point Energies= -3403.395114

Sum of electronic and thermal Energies= -3403.332969

Sum of electronic and thermal Enthalpies= -3403.332024

Sum of electronic and thermal Free Energies= -3403.495044

E(RM062X) = -3405.18158912

O 0.861115000000 0.930862000000 -1.159449000000

H 0.042487000000 0.418770000000 -1.078115000000

C 1.895265000000 0.097057000000 -0.761961000000

C 1.855994000000 -1.280654000000 -1.024293000000

C 0.889741000000 -1.976152000000 -1.706881000000

C -0.633634000000 -1.290103000000 1.113693000000

C -1.145398000000 -0.047516000000 1.067636000000

C -2.218929000000 0.364124000000 0.150315000000

N -2.950046000000 -0.553149000000 -0.386270000000

C -0.236850000000 -1.596254000000 -2.553738000000

C -1.090287000000 -2.722859000000 -2.627115000000

C -2.290894000000 -2.695017000000 -3.319213000000

C -2.627691000000 -1.524850000000 -4.000487000000

C -0.564988000000 -0.461160000000 -3.299019000000

C -1.765767000000 -0.430866000000 -4.008968000000

N -0.512365000000 -3.782207000000 -1.927225000000

C 0.701298000000 -3.400427000000 -1.360919000000

O 1.381642000000 -4.136106000000 -0.654870000000

C 0.417116000000 -1.730368000000 2.032643000000

C 1.188262000000 -0.832964000000 2.790742000000

C 2.080434000000 -1.303328000000 3.742911000000

C 2.221149000000 -2.678771000000 3.947650000000

C 1.505216000000 -3.575159000000 3.161671000000

C 0.614009000000 -3.105292000000 2.200564000000

C -2.299012000000 1.847038000000 -0.039117000000

C -2.575730000000 2.663378000000 1.062293000000

C -2.592874000000 4.046931000000 0.914663000000

C -2.279105000000 4.618372000000 -0.317622000000

C -1.970379000000 3.805882000000 -1.406130000000

C -1.990924000000 2.420749000000 -1.275824000000

S -4.337205000000 -0.202083000000 -1.301037000000

C -5.405512000000 0.536717000000 -0.083968000000

C -6.052469000000 -0.289371000000 0.832104000000

C -6.855840000000 0.289305000000 1.804553000000

C	-7.026880000000	1.677755000000	1.864428000000
C	-6.376556000000	2.478207000000	0.923565000000
C	-5.564451000000	1.917184000000	-0.057837000000
C	-7.924752000000	2.286588000000	2.909707000000
O	-4.099746000000	0.788615000000	-2.343218000000
O	-4.877356000000	-1.510572000000	-1.632448000000
C	-1.139814000000	-5.038565000000	-1.577801000000
C	-1.788522000000	-4.972061000000	-0.209366000000
C	-2.714706000000	-3.963648000000	0.077421000000
C	-3.237100000000	-3.839573000000	1.361063000000
C	-2.863729000000	-4.733425000000	2.363044000000
C	-1.961104000000	-5.753748000000	2.075591000000
C	-1.420113000000	-5.865754000000	0.795602000000
C	2.827500000000	0.673308000000	0.088344000000
N	2.699077000000	1.849750000000	0.800341000000
C	3.834339000000	1.974176000000	1.568812000000
N	4.678405000000	1.019952000000	1.372761000000
N	4.065175000000	0.196703000000	0.440867000000
C	3.931100000000	3.062453000000	2.594313000000
O	3.024214000000	4.095879000000	2.305075000000
C	1.690519000000	3.636980000000	2.153463000000
C	1.569261000000	2.772713000000	0.877351000000
C	0.843384000000	4.870441000000	1.838926000000
C	1.022485000000	5.018550000000	0.346191000000
C	1.462806000000	3.819675000000	-0.215989000000
C	0.795651000000	6.130104000000	-0.454483000000
C	1.021668000000	6.026327000000	-1.826680000000
C	1.471506000000	4.828862000000	-2.382816000000
C	1.693409000000	3.709335000000	-1.580514000000
C	4.880985000000	-0.672058000000	-0.360494000000
C	5.034939000000	-2.007256000000	0.025146000000
C	5.816748000000	-2.831329000000	-0.784851000000
C	6.418967000000	-2.335255000000	-1.935132000000
C	6.254788000000	-1.001443000000	-2.290515000000
C	5.483683000000	-0.141191000000	-1.508453000000
C	4.420292000000	-2.532801000000	1.296608000000
C	5.345713000000	-2.274712000000	2.490710000000
C	5.379390000000	1.320042000000	-1.873330000000
C	6.547866000000	2.125641000000	-1.291645000000
H	4.936173000000	3.487502000000	2.599602000000
H	3.729527000000	2.630590000000	3.587437000000
H	1.375168000000	3.092098000000	3.056413000000
H	0.649356000000	2.178468000000	0.905005000000
H	1.187466000000	5.730475000000	2.418301000000
H	-0.208122000000	4.676333000000	2.083098000000
H	0.457645000000	7.066684000000	-0.019536000000
H	0.856691000000	6.887827000000	-2.466413000000
H	1.651103000000	4.765758000000	-3.451243000000
H	2.021955000000	2.771194000000	-2.014117000000
H	-3.581135000000	-1.465059000000	-4.513671000000
H	-2.967307000000	-3.543525000000	-3.315110000000
H	0.104161000000	0.391985000000	-3.319512000000
H	-2.043872000000	0.463873000000	-4.556021000000
H	-1.051299000000	-2.042702000000	0.445804000000
H	5.937775000000	-3.875009000000	-0.509576000000
H	7.014614000000	-2.991927000000	-2.561224000000
H	6.725626000000	-0.613127000000	-3.189756000000
H	4.221125000000	-3.602877000000	1.182065000000
H	3.453092000000	-2.051739000000	1.477848000000
H	5.487242000000	-1.199081000000	2.634013000000
H	6.328237000000	-2.726048000000	2.323464000000
H	4.925365000000	-2.695843000000	3.407021000000
H	4.432317000000	1.743613000000	-1.524284000000
H	5.375982000000	1.410225000000	-2.964382000000
H	7.500468000000	1.752949000000	-1.678611000000
H	6.567515000000	2.034247000000	-0.202596000000

H	6.459521000000	3.183443000000	-1.553119000000
H	1.089716000000	0.238235000000	2.628546000000
H	2.673239000000	-0.604560000000	4.325551000000
H	2.905586000000	-3.045324000000	4.706436000000
H	1.634206000000	-4.644576000000	3.293179000000
H	0.059695000000	-3.801149000000	1.577659000000
H	-2.814817000000	2.210165000000	2.021226000000
H	-2.845510000000	4.677007000000	1.762205000000
H	-2.263992000000	5.697881000000	-0.429440000000
H	-1.704223000000	4.251832000000	-2.358512000000
H	-1.774620000000	1.776954000000	-2.123154000000
H	-5.932310000000	-1.365874000000	0.761123000000
H	-7.366574000000	-0.342289000000	2.526299000000
H	-6.506991000000	3.556242000000	0.957121000000
H	-5.054502000000	2.530842000000	-0.794560000000
H	-8.976843000000	2.162199000000	2.633756000000
H	-7.734230000000	3.355848000000	3.023591000000
H	-7.782048000000	1.806071000000	3.881027000000
H	-3.010621000000	-3.246350000000	-0.686556000000
H	-3.927919000000	-3.029191000000	1.574431000000
H	-3.270116000000	-4.631926000000	3.364434000000
H	-1.659836000000	-6.449889000000	2.852445000000
H	-0.682202000000	-6.634138000000	0.580459000000
H	-0.362679000000	-5.807317000000	-1.583085000000
H	-1.870433000000	-5.285130000000	-2.354425000000
H	2.558057000000	-1.909539000000	-0.483922000000
H	-0.763153000000	0.737637000000	1.714570000000

----

^{SS}**INTa6**

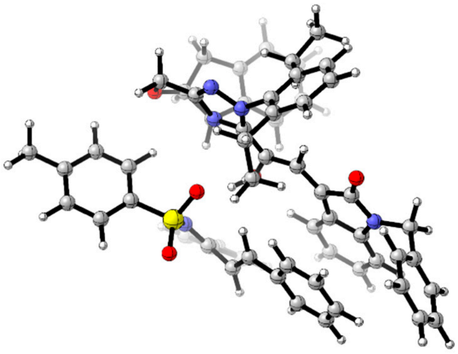

Zero-point correction= 1.041905 (Hartree/Particle)

Thermal correction to Energy= 1.103656

Thermal correction to Enthalpy= 1.104600

Thermal correction to Gibbs Free Energy= 0.941786

Sum of electronic and zero-point Energies= -3403.398978

Sum of electronic and thermal Energies= -3403.337228

Sum of electronic and thermal Enthalpies= -3403.336284

Sum of electronic and thermal Free Energies= -3403.499097

E(RM062X) = -3405.18440022

C	-2.204904000000	0.695467000000	0.657593000000
C	-0.962505000000	-2.523006000000	-0.666190000000
C	-1.051664000000	1.296526000000	0.223646000000
C	0.300437000000	0.953116000000	0.366013000000
C	-2.573050000000	-0.406058000000	1.541992000000
C	-3.989783000000	-0.420899000000	1.602050000000
C	-4.696671000000	-1.339856000000	2.360746000000
C	-3.969360000000	-2.261947000000	3.115282000000
C	-2.576045000000	-2.248027000000	3.097268000000
C	-1.870514000000	-1.331192000000	2.311232000000
H	-2.027125000000	-2.948426000000	3.720683000000
H	-4.496991000000	-2.987444000000	3.726491000000
H	-5.781928000000	-1.348264000000	2.355562000000
N	-4.502317000000	0.611557000000	0.822749000000
H	-0.786059000000	-1.334977000000	2.293356000000
C	-3.470317000000	1.322606000000	0.217482000000
C	-5.864378000000	1.100819000000	0.850501000000

O	-3.649632000000	2.282012000000	-0.524834000000
H	-1.253076000000	2.202853000000	-0.345579000000
C	1.350784000000	1.692676000000	-0.169400000000
O	0.682593000000	-0.237342000000	0.976011000000
N	1.365923000000	2.713657000000	-1.087087000000
N	2.686696000000	1.395860000000	0.040461000000
C	0.338869000000	3.692979000000	-1.285726000000
N	2.655103000000	3.018148000000	-1.482573000000
C	3.402486000000	2.207900000000	-0.815848000000
C	4.887682000000	2.057346000000	-0.945936000000
C	3.278454000000	0.881178000000	1.300692000000
C	-0.402629000000	3.673330000000	-2.467136000000
C	0.120419000000	4.618481000000	-0.252539000000
O	5.395148000000	1.186252000000	0.046315000000
H	5.122306000000	1.592877000000	-1.908946000000
H	5.369143000000	3.042168000000	-0.909387000000
C	4.770594000000	1.307750000000	1.307061000000
C	4.797248000000	2.716959000000	1.938844000000
H	5.302905000000	0.612661000000	1.961143000000
C	2.740550000000	1.623975000000	2.508843000000
H	3.143303000000	-0.201571000000	1.343073000000
C	3.590926000000	2.683618000000	2.842247000000
H	5.734529000000	2.908701000000	2.467082000000
H	4.685257000000	3.499233000000	1.177788000000
C	3.274706000000	3.537749000000	3.892437000000
C	1.576531000000	1.388868000000	3.235519000000
C	2.096431000000	3.319614000000	4.605605000000
H	3.935736000000	4.358411000000	4.156342000000
C	1.259784000000	2.251545000000	4.283696000000
H	1.833790000000	3.981107000000	5.425416000000
H	0.351477000000	2.085857000000	4.853581000000
H	0.933232000000	0.553771000000	2.986763000000
C	-1.424556000000	4.615481000000	-2.598893000000
C	-0.172511000000	2.624599000000	-3.526197000000
C	-1.676770000000	5.534775000000	-1.590427000000
H	-2.031723000000	4.611593000000	-3.499319000000
C	-0.907301000000	5.541990000000	-0.429482000000
H	-2.481124000000	6.254113000000	-1.704986000000
H	-1.114819000000	6.273478000000	0.343800000000
C	0.967224000000	4.563256000000	1.002596000000
C	0.819277000000	5.759438000000	1.935858000000
H	0.721959000000	3.645436000000	1.556554000000
H	2.020177000000	4.458019000000	0.707088000000
C	-0.850204000000	1.295861000000	-3.163731000000
H	-0.573560000000	3.000237000000	-4.472841000000
H	0.900794000000	2.463661000000	-3.667301000000
H	-0.836339000000	0.610901000000	-4.016520000000
H	-0.324724000000	0.805558000000	-2.338708000000
H	-1.888753000000	1.466095000000	-2.856860000000
H	1.516083000000	5.662813000000	2.771356000000
H	1.029430000000	6.699596000000	1.417118000000
H	-0.190020000000	5.818492000000	2.352696000000
C	-6.778359000000	0.463259000000	-0.171494000000
C	-6.614287000000	0.781570000000	-1.522191000000
C	-7.788536000000	-0.419244000000	0.203173000000
C	-7.446064000000	0.215602000000	-2.482105000000
H	-5.817897000000	1.469952000000	-1.798779000000
C	-8.454026000000	-0.671647000000	-2.101212000000
H	-7.313277000000	0.467696000000	-3.530009000000
C	-8.625321000000	-0.987632000000	-0.756798000000
H	-9.105745000000	-1.109620000000	-2.850675000000
H	-9.411048000000	-1.672476000000	-0.453324000000
H	-7.930741000000	-0.656873000000	1.255045000000
H	-5.798350000000	2.175786000000	0.654763000000
H	-6.262862000000	0.955564000000	1.861118000000
C	-0.267774000000	-3.504615000000	-0.062276000000

C	1.136270000000	-3.381023000000	0.332137000000
N	2.115425000000	-2.810943000000	-0.305558000000
C	-2.400863000000	-2.514359000000	-0.914271000000
C	-3.296439000000	-3.360625000000	-0.250068000000
C	-4.660129000000	-3.256342000000	-0.488989000000
C	-5.145654000000	-2.310850000000	-1.392180000000
C	-4.259417000000	-1.464240000000	-2.055068000000
C	-2.897656000000	-1.556014000000	-1.807270000000
C	1.504598000000	-3.931359000000	1.666913000000
C	2.844206000000	-4.206664000000	1.966718000000
C	3.192606000000	-4.686457000000	3.221580000000
C	2.211269000000	-4.887276000000	4.192472000000
C	0.878720000000	-4.609481000000	3.902755000000
C	0.524474000000	-4.137172000000	2.643319000000
S	1.988271000000	-2.222403000000	-1.864062000000
C	3.701251000000	-2.214007000000	-2.311798000000
C	4.147690000000	-3.040615000000	-3.331534000000
C	5.493349000000	-2.986965000000	-3.689555000000
C	6.379086000000	-2.125835000000	-3.040520000000
C	5.893320000000	-1.299656000000	-2.017380000000
C	4.557103000000	-1.337195000000	-1.651118000000
C	7.838041000000	-2.090904000000	-3.413491000000
O	1.277269000000	-3.102544000000	-2.779215000000
O	1.562983000000	-0.819073000000	-1.729454000000
H	-0.455894000000	-1.610660000000	-0.976433000000
H	-0.777515000000	-4.397755000000	0.286171000000
H	-2.931338000000	-4.059265000000	0.496743000000
H	-5.351363000000	-3.894200000000	0.052759000000
H	-6.213935000000	-2.213323000000	-1.563205000000
H	-4.636502000000	-0.719056000000	-2.746947000000
H	-2.201474000000	-0.881223000000	-2.298043000000
H	3.594671000000	-4.041274000000	1.201376000000
H	4.231133000000	-4.907341000000	3.445813000000
H	2.487812000000	-5.259076000000	5.174029000000
H	0.113344000000	-4.755058000000	4.658263000000
H	-0.511260000000	-3.897096000000	2.425314000000
H	3.447175000000	-3.700765000000	-3.832115000000
H	5.858905000000	-3.624369000000	-4.489449000000
H	6.562291000000	-0.617972000000	-1.497293000000
H	4.181817000000	-0.687114000000	-0.867287000000
H	8.438307000000	-2.627768000000	-2.671864000000
H	8.209380000000	-1.063695000000	-3.453344000000
H	8.011422000000	-2.557116000000	-4.385479000000
H	1.103830000000	-0.759814000000	0.273196000000

----

^{rr}**TSa4**

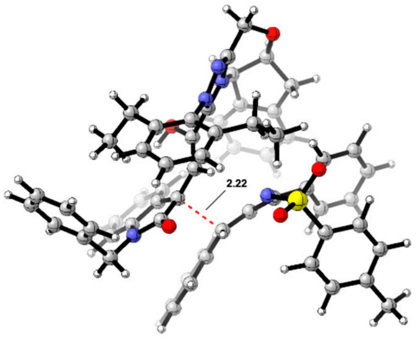

Frequency -262.9722

Zero-point correction= 1.042142 (Hartree/Particle)

Thermal correction to Energy= 1.102365

Thermal correction to Enthalpy= 1.103309

Thermal correction to Gibbs Free Energy= 0.947820

Sum of electronic and zero-point Energies= -3403.352979

Sum of electronic and thermal Energies= -3403.292756

Sum of electronic and thermal Enthalpies= -3403.291812

Sum of electronic and thermal Free Energies= -3403.447301

E(RM062X) = -3405.14418353

C	1.713340000000	-0.682520000000	0.560193000000

C	0.025732000000	-2.099172000000	0.300291000000
C	1.005666000000	0.526718000000	0.241220000000
C	0.928899000000	1.634090000000	1.002144000000
C	2.366252000000	-1.031826000000	1.815835000000
C	3.502124000000	-1.822322000000	1.503448000000
C	4.280641000000	-2.405577000000	2.489536000000
C	3.910947000000	-2.208691000000	3.824643000000
C	2.783306000000	-1.462795000000	4.154633000000
C	1.996081000000	-0.886721000000	3.152343000000
H	2.501504000000	-1.341595000000	5.195855000000
H	4.509401000000	-2.657726000000	4.610632000000
H	5.145580000000	-3.011592000000	2.239640000000
N	3.606383000000	-1.916300000000	0.124956000000
H	1.080613000000	-0.357143000000	3.400452000000
C	2.554238000000	-1.255869000000	-0.508595000000
C	4.662427000000	-2.537899000000	-0.644537000000
O	2.400089000000	-1.239254000000	-1.722125000000
H	0.508348000000	0.538471000000	-0.728839000000
C	0.305315000000	2.850438000000	0.515065000000
O	1.575577000000	1.905645000000	2.187000000000
H	2.173770000000	1.167994000000	2.386371000000
N	0.530607000000	3.435371000000	-0.669501000000
N	-0.416064000000	3.745691000000	1.225563000000
C	1.277782000000	2.880809000000	-1.778046000000
N	-0.032613000000	4.672345000000	-0.743472000000
C	-0.588781000000	4.839146000000	0.417930000000
C	-1.415307000000	5.963878000000	0.938474000000
C	-1.043082000000	3.737295000000	2.570104000000
C	2.655629000000	2.685689000000	-1.636294000000
C	0.542030000000	2.514603000000	-2.915352000000
O	-2.605907000000	5.428857000000	1.474684000000
H	-0.850349000000	6.529367000000	1.694192000000
H	-1.687540000000	6.635280000000	0.124205000000
C	-2.463942000000	4.369611000000	2.404026000000
C	-3.346766000000	3.176766000000	2.005298000000
H	-2.751995000000	4.729542000000	3.399972000000
C	-1.399896000000	2.375581000000	3.122973000000
H	-0.386314000000	4.313973000000	3.227187000000
C	-2.718002000000	2.053553000000	2.785587000000
H	-4.401113000000	3.340038000000	2.238669000000
H	-3.265028000000	3.020673000000	0.922991000000
C	-3.285170000000	0.864873000000	3.230161000000
C	-0.655483000000	1.561703000000	3.969395000000
C	-2.523063000000	0.020748000000	4.034462000000
H	-4.302375000000	0.599049000000	2.958494000000
C	-1.228313000000	0.373577000000	4.417401000000
H	-2.952495000000	-0.913301000000	4.384237000000
H	-0.663137000000	-0.280114000000	5.075220000000
H	0.351631000000	1.838488000000	4.257430000000
C	3.310667000000	2.071223000000	-2.704535000000
C	3.456175000000	3.090619000000	-0.415737000000
C	2.613949000000	1.663729000000	-3.832617000000
H	4.378754000000	1.893796000000	-2.631949000000
C	1.246099000000	1.889092000000	-3.942100000000
H	3.140525000000	1.158529000000	-4.636362000000
H	0.709907000000	1.556631000000	-4.822681000000
C	-0.947668000000	2.768452000000	-2.994694000000
C	-1.569323000000	2.528771000000	-4.366125000000
H	-1.450815000000	2.128922000000	-2.255931000000
H	-1.140354000000	3.802465000000	-2.691962000000
C	4.295104000000	1.946135000000	0.165534000000
H	4.122092000000	3.912900000000	-0.705139000000
H	2.805352000000	3.487407000000	0.368269000000
H	4.836019000000	2.285630000000	1.053096000000
H	3.662345000000	1.100411000000	0.448659000000
H	5.031680000000	1.572051000000	-0.552054000000

H	-2.633199000000	2.767464000000	-4.324959000000
H	-1.093125000000	3.159179000000	-5.124784000000
H	-1.491871000000	1.480046000000	-4.659639000000
C	5.660199000000	-1.506753000000	-1.127424000000
C	5.486106000000	-0.900306000000	-2.373034000000
C	6.717463000000	-1.100484000000	-0.310978000000
C	6.374799000000	0.082341000000	-2.803270000000
H	4.634733000000	-1.187505000000	-2.983266000000
C	7.429912000000	0.481996000000	-1.986001000000
H	6.240580000000	0.539437000000	-3.779826000000
C	7.597359000000	-0.108757000000	-0.735026000000
H	8.120507000000	1.248609000000	-2.323120000000
H	8.417001000000	0.198614000000	-0.093250000000
H	6.848972000000	-1.561928000000	0.665007000000
H	4.191617000000	-3.034607000000	-1.498245000000
H	5.142316000000	-3.299930000000	-0.023459000000
C	-1.168832000000	-1.538166000000	0.781986000000
C	-2.043326000000	-0.873945000000	-0.095533000000
N	-1.601328000000	-0.473093000000	-1.294981000000
C	0.667753000000	-3.266345000000	0.925330000000
C	0.543578000000	-3.560055000000	2.289828000000
C	1.214180000000	-4.642941000000	2.844431000000
C	2.012258000000	-5.458803000000	2.044365000000
C	2.132699000000	-5.187508000000	0.683276000000
C	1.468631000000	-4.098434000000	0.129905000000
C	-3.459049000000	-0.699206000000	0.352529000000
C	-4.118019000000	-1.725552000000	1.037938000000
C	-5.443631000000	-1.572454000000	1.431747000000
C	-6.124130000000	-0.388044000000	1.152261000000
C	-5.471653000000	0.641720000000	0.475125000000
C	-4.149085000000	0.485237000000	0.072963000000
S	-2.549711000000	-0.462528000000	-2.629473000000
C	-3.617996000000	-1.884892000000	-2.433370000000
C	-4.996048000000	-1.713303000000	-2.403043000000
C	-5.807701000000	-2.816022000000	-2.159638000000
C	-5.256576000000	-4.081662000000	-1.943234000000
C	-3.866323000000	-4.226217000000	-1.983930000000
C	-3.041610000000	-3.135381000000	-2.231437000000
C	-6.148047000000	-5.270656000000	-1.693619000000
O	-3.428250000000	0.713674000000	-2.726111000000
O	-1.659181000000	-0.711244000000	-3.760877000000
H	0.164329000000	-2.008356000000	-0.778381000000
H	-1.498436000000	-1.710259000000	1.801063000000
H	-0.061515000000	-2.919172000000	2.924241000000
H	1.122782000000	-4.845624000000	3.906870000000
H	2.535362000000	-6.303763000000	2.481086000000
H	2.747132000000	-5.822399000000	0.052103000000
H	1.575049000000	-3.865342000000	-0.927703000000
H	-3.594872000000	-2.659838000000	1.218248000000
H	-5.952189000000	-2.384725000000	1.941758000000
H	-7.161256000000	-0.272269000000	1.451861000000
H	-5.997256000000	1.564442000000	0.247558000000
H	-3.648955000000	1.264097000000	-0.490364000000
H	-5.406778000000	-0.718569000000	-2.541296000000
H	-6.886831000000	-2.690720000000	-2.122239000000
H	-3.426034000000	-5.205369000000	-1.815612000000
H	-1.960590000000	-3.242709000000	-2.252845000000
H	-5.611929000000	-6.066576000000	-1.171617000000
H	-6.518029000000	-5.683902000000	-2.637792000000
H	-7.018782000000	-4.992250000000	-1.094264000000

***rs*Tsa4**

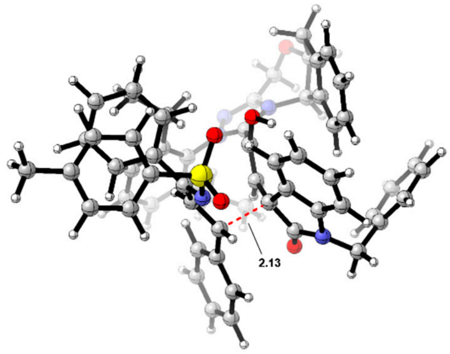

Frequency -375.4284

Zero-point correction= 1.042099 (Hartree/Particle)

Thermal correction to Energy= 1.102052

Thermal correction to Enthalpy= 1.102996

Thermal correction to Gibbs Free Energy= 0.947844

Sum of electronic and zero-point Energies= -3403.341565

Sum of electronic and thermal Energies= -3403.281612

Sum of electronic and thermal Enthalpies= -3403.280668

Sum of electronic and thermal Free Energies= -3403.435820

E(RM062X) = -3405.13288420

C	-0.801838000000	-0.992751000000	1.209210000000
C	1.022437000000	-0.434015000000	2.156644000000
C	-1.264423000000	0.267766000000	0.703733000000
C	-1.264225000000	0.756924000000	-0.552634000000
C	-0.438723000000	-2.191320000000	0.427351000000
C	-1.052131000000	-3.300269000000	1.046366000000
C	-0.869934000000	-4.596470000000	0.584407000000
C	-0.040448000000	-4.788193000000	-0.523617000000
C	0.602710000000	-3.710468000000	-1.129118000000
C	0.412804000000	-2.413507000000	-0.650847000000
H	1.304878000000	-3.867365000000	-1.940208000000
H	0.129315000000	-5.794312000000	-0.892712000000
H	-1.335179000000	-5.441830000000	1.081722000000
N	-1.788262000000	-2.871640000000	2.160764000000
H	0.968157000000	-1.596288000000	-1.100323000000
C	-1.740246000000	-1.481727000000	2.251259000000
C	-2.958835000000	-3.564431000000	2.680301000000
O	-2.422542000000	-0.824595000000	3.027912000000
H	-1.775579000000	0.868966000000	1.454500000000
C	-2.103894000000	1.915426000000	-0.821820000000
O	-0.767721000000	0.206998000000	-1.702881000000
H	-0.822328000000	-0.760360000000	-1.641162000000
N	-1.964011000000	3.175251000000	-0.390649000000
N	-3.215473000000	1.931251000000	-1.592781000000
C	-0.924010000000	3.700877000000	0.456276000000
N	-2.940261000000	4.001259000000	-0.874338000000
C	-3.680763000000	3.218514000000	-1.598670000000
C	-4.860348000000	3.501322000000	-2.475043000000
C	-4.123317000000	0.876014000000	-2.111636000000
C	-1.308814000000	4.271930000000	1.677593000000
C	0.398622000000	3.652785000000	-0.003153000000
O	-4.674750000000	2.769866000000	-3.668363000000
H	-5.796076000000	3.225082000000	-1.966841000000
H	-4.896886000000	4.560372000000	-2.730113000000
C	-4.597335000000	1.362395000000	-3.510905000000
C	-3.560293000000	0.766312000000	-4.476582000000
H	-5.584558000000	0.914040000000	-3.685473000000
C	-3.498411000000	-0.454055000000	-2.421253000000
H	-4.936560000000	0.803973000000	-1.381522000000
C	-3.157847000000	-0.507533000000	-3.774438000000
H	-3.965090000000	0.611586000000	-5.478957000000
H	-2.714627000000	1.460346000000	-4.554262000000
C	-2.522318000000	-1.632922000000	-4.282678000000
C	-3.274022000000	-1.524991000000	-1.561529000000
C	-2.245757000000	-2.692772000000	-3.418812000000
H	-2.238929000000	-1.685622000000	-5.329371000000
C	-2.629159000000	-2.649640000000	-2.076107000000
H	-1.727897000000	-3.571067000000	-3.790820000000

H	-2.406317000000	-3.487481000000	-1.421588000000
H	-3.566187000000	-1.491286000000	-0.513229000000
C	-0.295586000000	4.829605000000	2.456328000000
C	-2.740387000000	4.290116000000	2.170030000000
C	1.035142000000	4.767418000000	2.056490000000
H	-0.558571000000	5.299876000000	3.399309000000
C	1.377577000000	4.176892000000	0.847018000000
H	1.810206000000	5.178434000000	2.694881000000
H	2.418436000000	4.126791000000	0.544770000000
C	0.775252000000	3.072523000000	-1.347360000000
C	1.928171000000	3.808405000000	-2.028157000000
H	1.040509000000	2.015107000000	-1.216356000000
H	-0.097110000000	3.084840000000	-2.009409000000
C	-3.356972000000	2.904341000000	2.406388000000
H	-2.749483000000	4.843220000000	3.114114000000
H	-3.363386000000	4.857294000000	1.470813000000
H	-4.316112000000	3.007336000000	2.920104000000
H	-3.554695000000	2.374622000000	1.468509000000
H	-2.711060000000	2.269709000000	3.020997000000
H	2.057051000000	3.432384000000	-3.045084000000
H	1.735861000000	4.884439000000	-2.074777000000
H	2.875570000000	3.639944000000	-1.510790000000
C	-4.130972000000	-3.397051000000	1.733730000000
C	-4.960347000000	-2.273499000000	1.829186000000
C	-4.347127000000	-4.312137000000	0.700832000000
C	-5.986280000000	-2.077747000000	0.906852000000
H	-4.776998000000	-1.552823000000	2.620492000000
C	-6.189218000000	-2.992665000000	-0.124955000000
H	-6.632858000000	-1.209571000000	0.997903000000
C	-5.366653000000	-4.110794000000	-0.226522000000
H	-6.987041000000	-2.838033000000	-0.844606000000
H	-5.517616000000	-4.828615000000	-1.026510000000
H	-3.710608000000	-5.189093000000	0.620394000000
H	-3.179352000000	-3.119975000000	3.652837000000
H	-2.713367000000	-4.619936000000	2.826486000000
C	1.791059000000	0.295724000000	1.223681000000
C	2.770365000000	-0.318976000000	0.429220000000
N	3.052608000000	-1.608204000000	0.590826000000
C	0.474878000000	0.189559000000	3.379444000000
C	0.151286000000	1.549210000000	3.441089000000
C	-0.367851000000	2.098300000000	4.607457000000
C	-0.567910000000	1.301597000000	5.733538000000
C	-0.248501000000	-0.051557000000	5.682279000000
C	0.262892000000	-0.601899000000	4.512879000000
C	3.516025000000	0.573778000000	-0.527376000000
C	3.270727000000	0.535254000000	-1.902949000000
C	4.038646000000	1.307992000000	-2.770911000000
C	5.054664000000	2.122399000000	-2.277373000000
C	5.282759000000	2.186786000000	-0.904441000000
C	4.508574000000	1.425128000000	-0.032595000000
S	3.980119000000	-2.469602000000	-0.428896000000
C	5.580147000000	-1.665424000000	-0.462204000000
C	6.269219000000	-1.492688000000	0.734849000000
C	7.484534000000	-0.819370000000	0.725268000000
C	8.022153000000	-0.322622000000	-0.466572000000
C	7.322652000000	-0.533414000000	-1.656238000000
C	6.103376000000	-1.202948000000	-1.662017000000
C	9.314779000000	0.452398000000	-0.466665000000
O	4.174345000000	-3.776533000000	0.183861000000
O	3.456505000000	-2.418245000000	-1.804503000000
H	1.330819000000	-1.473128000000	2.269985000000
H	1.664733000000	1.369163000000	1.122673000000
H	0.303719000000	2.177689000000	2.568102000000
H	-0.615867000000	3.155793000000	4.636935000000
H	-0.971862000000	1.733493000000	6.643668000000
H	-0.405253000000	-0.682057000000	6.551767000000

H	0.495120000000	-1.662984000000	4.464753000000
H	2.511616000000	-0.134757000000	-2.292956000000
H	3.849886000000	1.260536000000	-3.839328000000
H	5.664338000000	2.708330000000	-2.958465000000
H	6.074355000000	2.817877000000	-0.511409000000
H	4.699037000000	1.447175000000	1.036650000000
H	5.846039000000	-1.875197000000	1.659031000000
H	8.027241000000	-0.675107000000	1.655893000000
H	7.732279000000	-0.156760000000	-2.590417000000
H	5.540601000000	-1.357143000000	-2.576572000000
H	9.902074000000	0.242196000000	-1.364212000000
H	9.924542000000	0.208874000000	0.406513000000
H	9.120921000000	1.530587000000	-0.446102000000

----

*sr*Tsa4

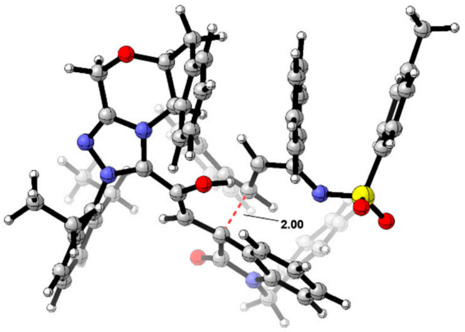

Frequency -432.7211

Zero-point correction= 1.041080 (Hartree/Particle)

Thermal correction to Energy= 1.101892

Thermal correction to Enthalpy= 1.102836

Thermal correction to Gibbs Free Energy= 0.941869

Sum of electronic and zero-point Energies= -3403.357554

Sum of electronic and thermal Energies= -3403.296742

Sum of electronic and thermal Enthalpies= -3403.295798

Sum of electronic and thermal Free Energies= -3403.456765

E(RM062X) = -3405.14596759

C	-0.548084000000	1.878067000000	-1.047656000000
C	0.572405000000	1.501417000000	0.560327000000
C	-1.748114000000	1.160903000000	-0.702194000000
C	-1.930809000000	-0.180106000000	-0.734106000000
O	-1.065043000000	-1.110602000000	-1.245359000000
H	-0.157499000000	-0.827300000000	-1.017387000000
C	0.968884000000	0.124952000000	0.524650000000
C	2.129498000000	-0.321300000000	-0.110762000000
N	2.962735000000	0.542072000000	-0.687946000000
C	0.384033000000	1.653076000000	-2.174871000000
C	1.080248000000	2.863815000000	-2.355228000000
C	2.164901000000	2.975836000000	-3.207241000000
C	2.535391000000	1.844935000000	-3.936647000000
C	0.704695000000	0.571465000000	-2.989526000000
C	1.790500000000	0.673352000000	-3.859906000000
N	0.491395000000	3.863026000000	-1.567415000000
C	-0.597881000000	3.363958000000	-0.879519000000
O	-1.374678000000	4.020825000000	-0.203249000000
C	-0.173464000000	1.967874000000	1.763449000000
C	-1.016564000000	1.124660000000	2.501203000000
C	-1.648826000000	1.575851000000	3.654578000000
C	-1.456221000000	2.884698000000	4.093149000000
C	-0.617903000000	3.731101000000	3.373270000000
C	0.017775000000	3.275787000000	2.223230000000
C	2.278274000000	-1.819350000000	-0.065120000000
C	2.461857000000	-2.463806000000	1.161340000000
C	2.480390000000	-3.854298000000	1.227449000000
C	2.276079000000	-4.610499000000	0.074293000000
C	2.081307000000	-3.971410000000	-1.148304000000
C	2.094597000000	-2.581028000000	-1.223519000000
S	4.355883000000	0.078935000000	-1.390491000000
C	5.171750000000	-0.956150000000	-0.172748000000

C	5.422426000000	-0.446458000000	1.096673000000
C	5.972875000000	-1.278106000000	2.065709000000
C	6.281375000000	-2.611061000000	1.777159000000
C	6.033213000000	-3.093652000000	0.489639000000
C	5.480458000000	-2.274144000000	-0.487894000000
C	6.886220000000	-3.509626000000	2.825355000000
O	4.158831000000	-0.775373000000	-2.568364000000
O	5.151046000000	1.294603000000	-1.543143000000
C	1.004617000000	5.199615000000	-1.358311000000
C	1.969465000000	5.257739000000	-0.188754000000
C	2.982988000000	4.302149000000	-0.043160000000
C	3.818325000000	4.335484000000	1.069271000000
C	3.657172000000	5.319548000000	2.043173000000
C	2.656164000000	6.275881000000	1.898888000000
C	1.816607000000	6.242598000000	0.787087000000
C	-3.025220000000	-0.801554000000	-0.028252000000
N	-2.937595000000	-1.976602000000	0.649272000000
C	-4.124423000000	-2.131981000000	1.317588000000
N	-4.949912000000	-1.161003000000	1.065274000000
N	-4.263213000000	-0.346241000000	0.206734000000
C	-4.297233000000	-3.238435000000	2.316727000000
O	-3.306624000000	-4.216892000000	2.150879000000
C	-1.984959000000	-3.693880000000	2.122627000000
C	-1.784650000000	-2.876441000000	0.828045000000
C	-1.048868000000	-4.885472000000	1.926861000000
C	-1.123250000000	-5.109653000000	0.434739000000
C	-1.581549000000	-3.961428000000	-0.213895000000
C	-0.792772000000	-6.244186000000	-0.292776000000
C	-0.938955000000	-6.218705000000	-1.679022000000
C	-1.410717000000	-5.074595000000	-2.321049000000
C	-1.732831000000	-3.929325000000	-1.592981000000
C	-4.919107000000	0.776768000000	-0.416342000000
C	-4.906547000000	2.014396000000	0.231440000000

C	-5.543107000000	3.075740000000	-0.415420000000
C	-6.162489000000	2.893886000000	-1.645114000000
C	-6.154836000000	1.645526000000	-2.259624000000
C	-5.527673000000	0.556134000000	-1.658380000000
C	-4.214884000000	2.229829000000	1.555209000000
C	-5.198071000000	2.519480000000	2.691244000000
C	-5.562362000000	-0.803765000000	-2.312304000000
C	-6.770511000000	-1.626451000000	-1.847490000000
H	-5.268881000000	-3.717530000000	2.185316000000
H	-4.259225000000	-2.796305000000	3.324174000000
H	-1.788497000000	-3.102387000000	3.028185000000
H	-0.888333000000	-2.251569000000	0.902522000000
H	-1.376315000000	-5.738545000000	2.525259000000
H	-0.028233000000	-4.611639000000	2.221555000000
H	-0.435270000000	-7.138152000000	0.210018000000
H	-0.690348000000	-7.099246000000	-2.262796000000
H	-1.524938000000	-5.070970000000	-3.400017000000
H	-2.072578000000	-3.032056000000	-2.096895000000
H	3.413332000000	1.884096000000	-4.571707000000
H	2.730975000000	3.898482000000	-3.285056000000
H	0.128080000000	-0.344051000000	-2.965653000000
H	2.083777000000	-0.186015000000	-4.451798000000
H	1.371058000000	2.180945000000	0.257535000000
H	-5.526087000000	4.056830000000	0.050054000000
H	-6.643005000000	3.733314000000	-2.136936000000
H	-6.631324000000	1.509169000000	-3.226190000000
H	-3.495564000000	3.053237000000	1.449245000000
H	-3.620546000000	1.350739000000	1.814729000000
H	-5.887377000000	1.681410000000	2.828482000000
H	-5.791164000000	3.414377000000	2.483891000000
H	-4.652937000000	2.682255000000	3.624676000000
H	-4.637398000000	-1.354838000000	-2.109275000000
H	-5.605028000000	-0.665612000000	-3.396720000000

H	-7.701211000000	-1.108885000000	-2.094935000000
H	-6.746772000000	-1.772998000000	-0.764462000000
H	-6.782028000000	-2.605884000000	-2.332014000000
H	-1.179523000000	0.100248000000	2.174025000000
H	-2.295065000000	0.904957000000	4.214705000000
H	-1.946729000000	3.235289000000	4.996178000000
H	-0.449266000000	4.750122000000	3.706698000000
H	0.678705000000	3.938992000000	1.672870000000
H	2.621890000000	-1.865030000000	2.053690000000
H	2.659655000000	-4.346980000000	2.179378000000
H	2.271861000000	-5.695195000000	0.125726000000
H	1.913023000000	-4.557337000000	-2.046551000000
H	1.979612000000	-2.072428000000	-2.176395000000
H	5.165799000000	0.584547000000	1.322342000000
H	6.162933000000	-0.890786000000	3.063408000000
H	6.262245000000	-4.130470000000	0.255856000000
H	5.250792000000	-2.642611000000	-1.482410000000
H	7.962866000000	-3.627683000000	2.664573000000
H	6.440032000000	-4.507608000000	2.793278000000
H	6.743212000000	-3.102025000000	3.828849000000
H	3.121321000000	3.506769000000	-0.773696000000
H	4.594186000000	3.582563000000	1.164595000000
H	4.306080000000	5.338016000000	2.913007000000
H	2.520065000000	7.042345000000	2.655673000000
H	1.019599000000	6.974857000000	0.685953000000
H	0.146041000000	5.848405000000	-1.165070000000
H	1.478092000000	5.534472000000	-2.287511000000
H	-2.488497000000	1.767284000000	-0.190210000000
H	0.428488000000	-0.601733000000	1.120002000000

----

^{ss}Tsa4

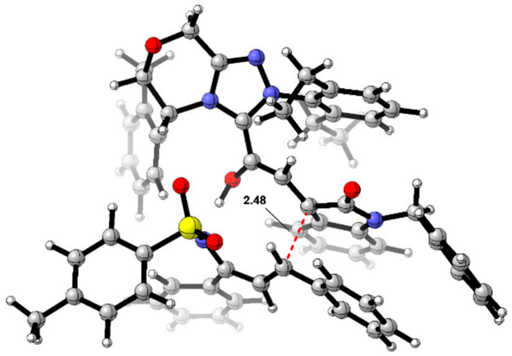

Frequency -205.1711

Zero-point correction=	1.041625 (Hartree/Particle)
Thermal correction to Energy=	1.101959
Thermal correction to Enthalpy=	1.102903
Thermal correction to Gibbs Free Energy=	0.943058
Sum of electronic and zero-point Energies=	-3403.363256
Sum of electronic and thermal Energies=	-3403.302922
Sum of electronic and thermal Enthalpies=	-3403.301978
Sum of electronic and thermal Free Energies=	-3403.461822

E(RM062X) = -3405.15071741

C	-1.567963000000	0.213242000000	0.225439000000
C	-1.327363000000	-2.076753000000	-0.692015000000
C	-0.545543000000	1.000440000000	-0.347032000000
C	0.714495000000	1.325152000000	0.091726000000
C	-1.793799000000	-0.165511000000	1.611183000000
C	-3.196922000000	-0.154885000000	1.821974000000
C	-3.758488000000	-0.485486000000	3.045022000000
C	-2.896787000000	-0.827810000000	4.093032000000
C	-1.516721000000	-0.835147000000	3.908150000000
C	-0.954946000000	-0.505405000000	2.669440000000
H	-0.862666000000	-1.098060000000	4.734015000000
H	-3.314854000000	-1.087385000000	5.060413000000
H	-4.834503000000	-0.483653000000	3.187552000000
N	-3.839945000000	0.243941000000	0.651114000000
H	0.121050000000	-0.487357000000	2.536839000000

C	-2.904420000000	0.506047000000	-0.343119000000	H	3.614398000000	-0.416491000000	0.442816000000
C	-5.182409000000	0.789460000000	0.569442000000	C	-3.100213000000	4.272825000000	-1.286517000000
O	-3.191572000000	0.954997000000	-1.447330000000	C	-1.087749000000	4.299425000000	-2.794713000000
H	-0.889480000000	1.524619000000	-1.234283000000	C	-3.712838000000	4.045828000000	-0.063474000000
C	1.234134000000	2.611488000000	-0.268171000000	H	-3.696052000000	4.521861000000	-2.159205000000
O	1.445106000000	0.637579000000	0.999336000000	C	-2.952358000000	3.702291000000	1.050027000000
N	0.452319000000	3.705703000000	-0.431219000000	H	-4.791146000000	4.135477000000	0.027476000000
N	2.518642000000	3.068447000000	-0.396473000000	H	-3.446247000000	3.519954000000	1.998077000000
C	-0.980543000000	3.827211000000	-0.284841000000	C	-0.726135000000	3.275093000000	2.194747000000
N	1.181176000000	4.807225000000	-0.761466000000	C	-1.510366000000	3.086558000000	3.489471000000
C	2.410625000000	4.399529000000	-0.738585000000	H	-0.169178000000	2.350617000000	2.012777000000
C	3.584007000000	5.247464000000	-1.114724000000	H	0.026919000000	4.064546000000	2.320153000000
C	3.810172000000	2.316900000000	-0.537257000000	C	-0.681584000000	2.950224000000	-3.405127000000
C	-1.717901000000	4.154770000000	-1.427830000000	H	-1.823959000000	4.777154000000	-3.448153000000
C	-1.562277000000	3.604632000000	0.975567000000	H	-0.224045000000	4.968354000000	-2.749880000000
O	4.628342000000	4.437227000000	-1.590417000000	H	-0.400888000000	3.076485000000	-4.453818000000
H	3.281076000000	5.911562000000	-1.925612000000	H	0.178272000000	2.506312000000	-2.891967000000
H	3.901760000000	5.866840000000	-0.265733000000	H	-1.509337000000	2.236831000000	-3.346412000000
C	4.933894000000	3.377482000000	-0.717995000000	H	-0.819285000000	2.864148000000	4.305404000000
C	5.334676000000	3.768492000000	0.721567000000	H	-2.080056000000	3.980291000000	3.760789000000
H	5.773490000000	2.852461000000	-1.179870000000	H	-2.199829000000	2.238891000000	3.406864000000
C	4.334408000000	1.602906000000	0.695358000000	C	-6.223003000000	-0.081240000000	-0.097917000000
H	3.681578000000	1.652449000000	-1.392877000000	C	-6.385976000000	-0.014832000000	-1.482389000000
C	5.189662000000	2.441876000000	1.416423000000	C	-7.077703000000	-0.890745000000	0.650062000000
H	6.344506000000	4.182971000000	0.768506000000	C	-7.407818000000	-0.726367000000	-2.104403000000
H	4.648391000000	4.510527000000	1.147007000000	H	-5.692969000000	0.594015000000	-2.057404000000
C	5.850872000000	1.980230000000	2.548129000000	C	-8.269708000000	-1.518669000000	-1.349257000000
C	4.210649000000	0.261238000000	1.042192000000	H	-7.535092000000	-0.658668000000	-3.180844000000
C	5.668433000000	0.655163000000	2.936252000000	C	-8.096051000000	-1.609832000000	0.029930000000
H	6.514665000000	2.634380000000	3.105733000000	H	-9.073617000000	-2.064400000000	-1.834022000000
C	4.871688000000	-0.202074000000	2.176847000000	H	-8.761547000000	-2.228684000000	0.624103000000
H	6.174363000000	0.278615000000	3.819912000000	H	-6.956624000000	-0.944418000000	1.729872000000
H	4.760478000000	-1.242357000000	2.467271000000	H	-5.097862000000	1.722698000000	0.003095000000

H	-5.497214000000	1.037708000000	1.588534000000
C	-0.497835000000	-2.726470000000	0.221381000000
C	0.862479000000	-2.505743000000	0.450033000000
N	1.712236000000	-1.685642000000	-0.204330000000
C	-2.730171000000	-2.460685000000	-0.815788000000
C	-3.446861000000	-3.037905000000	0.246260000000
C	-4.755162000000	-3.460182000000	0.059684000000
C	-5.363731000000	-3.325317000000	-1.186972000000
C	-4.673669000000	-2.722434000000	-2.236150000000
C	-3.374549000000	-2.272200000000	-2.047966000000
C	1.499164000000	-3.145461000000	1.640076000000
C	2.869798000000	-3.435387000000	1.596714000000
C	3.497800000000	-4.041908000000	2.678640000000
C	2.771862000000	-4.349257000000	3.827407000000
C	1.412286000000	-4.051827000000	3.885200000000
C	0.778304000000	-3.457117000000	2.800269000000
S	1.921878000000	-1.452662000000	-1.796518000000
C	3.198633000000	-2.649542000000	-2.141878000000
C	2.835389000000	-3.920984000000	-2.572595000000
C	3.827656000000	-4.876415000000	-2.760122000000
C	5.172023000000	-4.577398000000	-2.520053000000
C	5.507548000000	-3.288799000000	-2.095099000000
C	4.528307000000	-2.320360000000	-1.901427000000
C	6.232661000000	-5.632893000000	-2.698094000000
O	0.798787000000	-1.793342000000	-2.668164000000
O	2.524465000000	-0.120606000000	-1.914391000000
H	-0.904848000000	-1.516776000000	-1.514103000000
H	-0.958424000000	-3.454785000000	0.878534000000
H	-2.986535000000	-3.127566000000	1.226109000000
H	-5.306226000000	-3.893949000000	0.888411000000
H	-6.385281000000	-3.660324000000	-1.331303000000
H	-5.158605000000	-2.588455000000	-3.197823000000
H	-2.834282000000	-1.786505000000	-2.855020000000
H	3.428884000000	-3.187732000000	0.698998000000
H	4.556761000000	-4.275637000000	2.624293000000
H	3.264324000000	-4.814062000000	4.675721000000
H	0.844078000000	-4.273453000000	4.782964000000
H	-0.270661000000	-3.187847000000	2.872925000000
H	1.790758000000	-4.141088000000	-2.768065000000
H	3.555750000000	-5.871244000000	-3.102340000000
H	6.550443000000	-3.040364000000	-1.918216000000
H	4.783692000000	-1.315695000000	-1.577671000000
H	6.282354000000	-6.283224000000	-1.818592000000
H	7.219011000000	-5.185064000000	-2.837245000000
H	6.017488000000	-6.265749000000	-3.562573000000
H	1.632372000000	-0.268303000000	0.599679000000

----

^{rr}INTa7

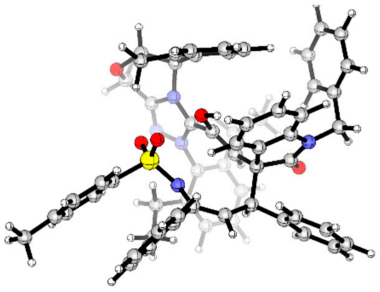

Zero-point correction= 1.043752 (Hartree/Particle)

Thermal correction to Energy= 1.103952

Thermal correction to Enthalpy= 1.104896

Thermal correction to Gibbs Free Energy= 0.948046

Sum of electronic and zero-point Energies= -3403.369827

Sum of electronic and thermal Energies= -3403.309627

Sum of electronic and thermal Enthalpies= -3403.308683

Sum of electronic and thermal Free Energies= -3403.465533

E(RM062X) = -3405.16251627

C	-1.363636000000	-1.435474000000	0.383531000000

C	-0.435587000000	-2.546624000000	1.057306000000
C	-1.079823000000	-0.081163000000	0.974136000000
C	-0.660313000000	1.021111000000	0.347715000000
C	-1.478439000000	-1.587034000000	-1.121397000000
C	-2.809193000000	-1.889846000000	-1.442816000000
C	-3.228014000000	-2.118167000000	-2.745256000000
C	-2.264689000000	-2.058971000000	-3.754851000000
C	-0.935959000000	-1.772997000000	-3.458175000000
C	-0.529537000000	-1.543830000000	-2.137892000000
H	-0.198849000000	-1.711356000000	-4.251859000000
H	-2.564081000000	-2.234203000000	-4.783675000000
H	-4.265206000000	-2.338563000000	-2.972505000000
N	-3.585853000000	-1.940915000000	-0.281329000000
H	0.500449000000	-1.274101000000	-1.930615000000
C	-2.835148000000	-1.650894000000	0.830941000000
C	-5.035837000000	-1.963481000000	-0.262419000000
O	-3.276303000000	-1.506762000000	1.955767000000
H	-1.315719000000	-0.035493000000	2.030091000000
C	-0.300670000000	2.267182000000	1.042346000000
O	-0.319233000000	1.145069000000	-0.940389000000
H	-0.699798000000	0.441898000000	-1.485985000000
N	0.213065000000	2.410994000000	2.273091000000
N	-0.171015000000	3.478064000000	0.461864000000
C	0.033774000000	1.499953000000	3.374891000000
N	0.771296000000	3.639831000000	2.455641000000
C	0.547956000000	4.252063000000	1.330673000000
C	1.046598000000	5.561140000000	0.798571000000
C	-0.767211000000	4.210684000000	-0.695396000000
C	-1.220079000000	1.514583000000	3.997293000000
C	1.069297000000	0.627683000000	3.726498000000
O	1.446395000000	5.343072000000	-0.537729000000
H	0.273860000000	6.341377000000	0.872687000000
H	1.923001000000	5.876617000000	1.364474000000
C	0.406855000000	4.929880000000	-1.406575000000
C	0.878770000000	3.887722000000	-2.432831000000
H	-0.021631000000	5.807310000000	-1.913080000000
C	-1.365426000000	3.385732000000	-1.789306000000
H	-1.480067000000	4.904994000000	-0.237968000000
C	-0.411650000000	3.188927000000	-2.788797000000
H	1.401617000000	4.334078000000	-3.280997000000
H	1.545140000000	3.172649000000	-1.928227000000
C	-0.692686000000	2.312524000000	-3.828757000000
C	-2.603132000000	2.757931000000	-1.803199000000
C	-1.919925000000	1.644894000000	-3.834540000000
H	0.047965000000	2.110103000000	-4.595782000000
C	-2.874965000000	1.863651000000	-2.836721000000
H	-2.133113000000	0.930179000000	-4.623244000000
H	-3.819926000000	1.325940000000	-2.854183000000
H	-3.326184000000	2.913329000000	-1.006621000000
C	-1.464965000000	0.540173000000	4.965179000000
C	-2.306511000000	2.509289000000	3.648702000000
C	-0.486998000000	-0.390313000000	5.290812000000
H	-2.435075000000	0.512675000000	5.453298000000
C	0.768286000000	-0.333803000000	4.693447000000
H	-0.695498000000	-1.156774000000	6.030470000000
H	1.527329000000	-1.053555000000	4.976779000000
C	2.440155000000	0.754435000000	3.109703000000
C	3.280646000000	-0.514489000000	3.205776000000
H	2.326513000000	1.036754000000	2.060038000000
H	2.951936000000	1.587562000000	3.611655000000
C	-3.365917000000	1.950016000000	2.689117000000
H	-2.794843000000	2.798024000000	4.585096000000
H	-1.868054000000	3.425505000000	3.238508000000
H	-4.223019000000	2.627485000000	2.629221000000
H	-2.963343000000	1.814187000000	1.680961000000
H	-3.715895000000	0.969746000000	3.028106000000

H	4.214969000000	-0.382989000000	2.654293000000
H	3.540995000000	-0.763972000000	4.239222000000
H	2.742428000000	-1.355457000000	2.756955000000
C	-5.595466000000	-0.638409000000	-0.736779000000
C	-5.307796000000	0.514514000000	0.000352000000
C	-6.345812000000	-0.529634000000	-1.904833000000
C	-5.769465000000	1.754244000000	-0.425743000000
H	-4.726089000000	0.421942000000	0.914557000000
C	-6.512173000000	1.859203000000	-1.602676000000
H	-5.553577000000	2.641498000000	0.163261000000
C	-6.801837000000	0.715155000000	-2.339365000000
H	-6.866779000000	2.827817000000	-1.939795000000
H	-7.381477000000	0.788274000000	-3.253999000000
H	-6.578975000000	-1.422891000000	-2.479618000000
H	-5.318476000000	-2.160970000000	0.774908000000
H	-5.392850000000	-2.791779000000	-0.882928000000
C	0.909179000000	-2.785946000000	0.402398000000
C	1.896469000000	-1.927246000000	0.050128000000
N	1.859335000000	-0.565963000000	0.293464000000
C	-1.218887000000	-3.850968000000	1.097300000000
C	-1.340916000000	-4.641409000000	-0.050595000000
C	-2.116941000000	-5.796450000000	-0.037330000000
C	-2.777956000000	-6.182576000000	1.126007000000
C	-2.659450000000	-5.404936000000	2.274235000000
C	-1.888655000000	-4.245691000000	2.256865000000
C	3.120491000000	-2.555954000000	-0.554818000000
C	4.316583000000	-2.604224000000	0.168442000000
C	5.448310000000	-3.196148000000	-0.377951000000
C	5.404495000000	-3.733480000000	-1.664193000000
C	4.219854000000	-3.685456000000	-2.392408000000
C	3.080559000000	-3.105087000000	-1.836508000000
S	2.495864000000	0.500380000000	-0.705478000000
C	4.289679000000	0.403373000000	-0.751115000000
C	4.925965000000	-0.148343000000	-1.856602000000
C	6.309382000000	-0.281274000000	-1.848415000000
C	7.066540000000	0.127446000000	-0.747840000000
C	6.407831000000	0.713393000000	0.337017000000
C	5.025099000000	0.862833000000	0.336124000000
C	8.558825000000	-0.082178000000	-0.725823000000
O	2.098482000000	0.315544000000	-2.117499000000
O	2.233389000000	1.826785000000	-0.094936000000
H	-0.306298000000	-2.197565000000	2.092069000000
H	1.133499000000	-3.835446000000	0.240706000000
H	-0.817549000000	-4.347027000000	-0.956914000000
H	-2.200287000000	-6.397947000000	-0.937327000000
H	-3.379476000000	-7.086135000000	1.138133000000
H	-3.170545000000	-5.698171000000	3.186027000000
H	-1.820079000000	-3.622417000000	3.144208000000
H	4.350453000000	-2.165063000000	1.160941000000
H	6.370725000000	-3.226661000000	0.194709000000
H	6.291524000000	-4.187447000000	-2.095595000000
H	4.178610000000	-4.103431000000	-3.393835000000
H	2.149522000000	-3.067573000000	-2.396183000000
H	4.326471000000	-0.479381000000	-2.697711000000
H	6.809747000000	-0.727546000000	-2.704345000000
H	6.988148000000	1.064568000000	1.186665000000
H	4.510760000000	1.349657000000	1.159661000000
H	9.044685000000	0.584031000000	-0.008968000000
H	8.998797000000	0.094241000000	-1.710825000000
H	8.800564000000	-1.111760000000	-0.439601000000

----

^{rs}INTa7

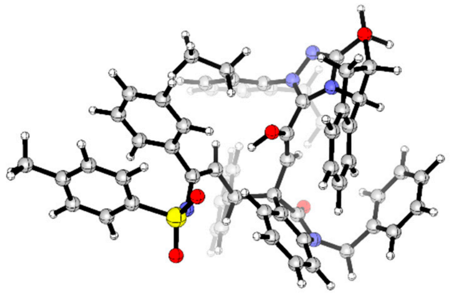

Zero-point correction= 1.043781 (Hartree/Particle)

Thermal correction to Energy= 1.103890

Thermal correction to Enthalpy= 1.104835

Thermal correction to Gibbs Free Energy= 0.948170

Sum of electronic and zero-point Energies= -3403.359541

Sum of electronic and thermal Energies= -3403.299431

Sum of electronic and thermal Enthalpies= -3403.298487

Sum of electronic and thermal Free Energies= -3403.455152

E(RM062X) = -3405.15087112

C	1.279619000000	-0.525856000000	-0.488676000000
H	-0.419066000000	0.166350000000	-0.890346000000
C	1.416565000000	-0.196384000000	0.796150000000
C	0.670876000000	0.861692000000	1.579332000000
C	0.443693000000	2.218851000000	0.918847000000
C	1.223989000000	3.174426000000	1.579929000000
C	1.121756000000	4.534515000000	1.320650000000
C	0.164819000000	4.948009000000	0.389751000000
C	-0.642933000000	4.018182000000	-0.257683000000
C	-0.498396000000	2.652447000000	-0.002367000000
H	-1.425899000000	4.326980000000	-0.942263000000
H	0.038079000000	6.007869000000	0.193129000000
H	1.723632000000	5.257403000000	1.862278000000
N	1.994759000000	2.562905000000	2.589087000000
H	-1.172424000000	1.979256000000	-0.512693000000
C	1.720888000000	1.219133000000	2.663200000000
C	3.296330000000	3.050733000000	3.026093000000
O	2.331080000000	0.431437000000	3.362018000000
H	2.143230000000	-0.755318000000	1.375764000000
C	2.187183000000	-1.556087000000	-1.049457000000
O	0.398895000000	-0.045201000000	-1.395181000000
N	2.188076000000	-2.867213000000	-0.781035000000
N	3.174073000000	-1.383903000000	-1.947246000000
C	1.340513000000	-3.592772000000	0.136113000000
N	3.129856000000	-3.540862000000	-1.497688000000
C	3.715696000000	-2.614709000000	-2.196449000000
C	4.745463000000	-2.692940000000	-3.278388000000
C	3.850080000000	-0.201464000000	-2.534281000000
C	1.941222000000	-4.155144000000	1.268800000000
C	-0.008674000000	-3.750730000000	-0.202222000000
O	4.272022000000	-1.899024000000	-4.344696000000
H	5.723656000000	-2.348716000000	-2.912299000000
H	4.839755000000	-3.718672000000	-3.634630000000
C	4.095972000000	-0.524726000000	-4.038814000000
C	2.852893000000	0.032949000000	-4.749270000000
H	4.990927000000	0.037905000000	-4.336098000000
C	3.033754000000	1.050248000000	-2.593708000000
H	4.775884000000	-0.081213000000	-1.961453000000
C	2.464504000000	1.190043000000	-3.860788000000
H	3.059833000000	0.316752000000	-5.783303000000
H	2.076444000000	-0.741767000000	-4.760344000000
C	1.677269000000	2.298946000000	-4.140215000000
C	2.874116000000	2.004933000000	-1.598592000000
C	1.495129000000	3.255918000000	-3.141975000000
H	1.216284000000	2.423413000000	-5.115096000000
C	2.098440000000	3.120932000000	-1.890666000000
H	0.874081000000	4.124170000000	-3.338514000000
H	1.952658000000	3.880418000000	-1.130942000000
H	3.328069000000	1.883864000000	-0.617731000000
C	1.107977000000	-4.882100000000	2.118371000000

C	3.413444000000	-4.016081000000	1.590166000000	C	-1.480679000000	-0.378176000000	1.015892000000
C	-0.252102000000	-5.003829000000	1.858457000000	C	-2.632714000000	0.143588000000	0.495763000000
H	1.538420000000	-5.343742000000	3.001989000000	N	-3.140702000000	1.324919000000	0.955103000000
C	-0.804031000000	-4.446252000000	0.711141000000	C	-0.536408000000	-0.540085000000	3.362977000000
H	-0.887058000000	-5.546172000000	2.551381000000	C	-0.025047000000	-1.838712000000	3.307372000000
H	-1.861742000000	-4.567166000000	0.508272000000	C	0.169316000000	-2.572959000000	4.472240000000
C	-0.556274000000	-3.239025000000	-1.517795000000	C	-0.153244000000	-2.025667000000	5.712096000000
C	-1.885462000000	-3.864845000000	-1.926301000000	C	-0.673664000000	-0.738049000000	5.776903000000
H	-0.681548000000	-2.150681000000	-1.474306000000	C	-0.859401000000	-0.002786000000	4.609338000000
H	0.195523000000	-3.428971000000	-2.295006000000	C	-3.375242000000	-0.669330000000	-0.532132000000
C	3.852969000000	-2.596770000000	1.974099000000	C	-3.092037000000	-0.570639000000	-1.896245000000
H	3.625269000000	-4.687310000000	2.427084000000	C	-3.859159000000	-1.269892000000	-2.827022000000
H	4.011293000000	-4.374721000000	0.745963000000	C	-4.913236000000	-2.074047000000	-2.404401000000
H	4.862217000000	-2.617484000000	2.393536000000	C	-5.197563000000	-2.182573000000	-1.043962000000
H	3.882941000000	-1.930307000000	1.104417000000	C	-4.431247000000	-1.486270000000	-0.114517000000
H	3.183132000000	-2.153427000000	2.718495000000	S	-3.945218000000	2.302202000000	-0.027410000000
H	-2.141914000000	-3.557696000000	-2.941254000000	C	-5.545914000000	1.549526000000	-0.333992000000
H	-1.836585000000	-4.957651000000	-1.895742000000	C	-6.357176000000	1.238207000000	0.753459000000
H	-2.699848000000	-3.523824000000	-1.280079000000	C	-7.563734000000	0.582930000000	0.538235000000
C	4.319731000000	2.806063000000	1.934425000000	C	-7.974288000000	0.238616000000	-0.753269000000
C	4.876298000000	1.530408000000	1.774627000000	C	-7.149276000000	0.574991000000	-1.828490000000
C	4.650156000000	3.809473000000	1.021758000000	C	-5.939232000000	1.230787000000	-1.627165000000
C	5.756207000000	1.275006000000	0.725274000000	C	-9.284114000000	-0.469925000000	-0.988748000000
H	4.610784000000	0.747495000000	2.480170000000	O	-4.206329000000	3.547546000000	0.690611000000
C	6.086255000000	2.285737000000	-0.177307000000	O	-3.303136000000	2.406977000000	-1.358211000000
H	6.199647000000	0.287726000000	0.624850000000	H	-1.291843000000	1.197275000000	2.394986000000
C	5.529566000000	3.553337000000	-0.028361000000	H	-1.216137000000	-1.405166000000	0.767855000000
H	6.780097000000	2.089672000000	-0.989416000000	H	0.225542000000	-2.275945000000	2.343407000000
H	5.775808000000	4.342762000000	-0.731000000000	H	0.572011000000	-3.579435000000	4.412663000000
H	4.213928000000	4.798658000000	1.133519000000	H	-0.002110000000	-2.601555000000	6.619829000000
H	3.549321000000	2.499095000000	3.933394000000	H	-0.935688000000	-0.302599000000	6.736288000000
H	3.220909000000	4.112993000000	3.271999000000	H	-1.264786000000	1.004285000000	4.661347000000
C	-0.712944000000	0.309248000000	2.121984000000	H	-2.311310000000	0.105450000000	-2.233820000000

H -3.640528000000 -1.170941000000 -3.886440000000

H -5.518775000000 -2.606631000000 -3.131609000000

H -6.029244000000 -2.794911000000 -0.708374000000

H -4.665709000000 -1.538624000000 0.944875000000

H -6.025600000000 1.491095000000 1.756370000000

H -8.197017000000 0.327976000000 1.384276000000

H -7.451901000000 0.304185000000 -2.837572000000

H -5.278783000000 1.477617000000 -2.451705000000

H -10.040559000000 0.220908000000 -1.375632000000

H -9.672953000000 -0.903866000000 -0.064471000000

H -9.170527000000 -1.273368000000 -1.722434000000

----

^{sr}**INTa7**

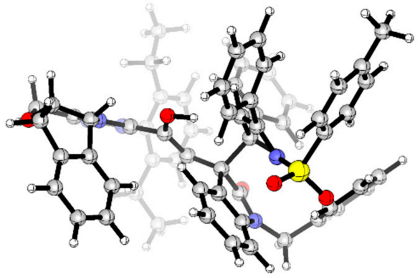

Zero-point correction= 1.041080 (Hartree/Particle)

Thermal correction to Energy= 1.101892

Thermal correction to Enthalpy= 1.102836

Thermal correction to Gibbs Free Energy= 0.941869

Sum of electronic and zero-point Energies= -3403.357554

Sum of electronic and thermal Energies= -3403.296742

Sum of electronic and thermal Enthalpies= -3403.295798

Sum of electronic and thermal Free Energies= -3403.456765

E(RM062X) = -3405.14596759

C -0.548084000000 1.878067000000 -1.047656000000

C 0.572405000000 1.501417000000 0.560327000000

C -1.748114000000 1.160903000000 -0.702194000000

C -1.930809000000 -0.180106000000 -0.734106000000

O -1.065043000000 -1.110602000000 -1.245359000000

H -0.157499000000 -0.827300000000 -1.017387000000

C 0.968884000000 0.124952000000 0.524650000000

C 2.129498000000 -0.321300000000 -0.110762000000

N 2.962735000000 0.542072000000 -0.687946000000

C 0.384033000000 1.653076000000 -2.174871000000

C 1.080248000000 2.863815000000 -2.355228000000

C 2.164901000000 2.975836000000 -3.207241000000

C 2.535391000000 1.844935000000 -3.936647000000

C 0.704695000000 0.571465000000 -2.989526000000

C 1.790500000000 0.673352000000 -3.859906000000

N 0.491395000000 3.863026000000 -1.567415000000

C -0.597881000000 3.363958000000 -0.879519000000

O -1.374678000000 4.020825000000 -0.203249000000

C -0.173464000000 1.967874000000 1.763449000000

C -1.016564000000 1.124660000000 2.501203000000

C -1.648826000000 1.575851000000 3.654578000000

C -1.456221000000 2.884698000000 4.093149000000

C -0.617903000000 3.731101000000 3.373270000000

C 0.017775000000 3.275787000000 2.223230000000

C 2.278274000000 -1.819350000000 -0.065120000000

C 2.461857000000 -2.463806000000 1.161340000000

C 2.480390000000 -3.854298000000 1.227449000000

C 2.276079000000 -4.610499000000 0.074293000000

C 2.081307000000 -3.971410000000 -1.148304000000

C 2.094597000000 -2.581028000000 -1.223519000000

S 4.355883000000 0.078935000000 -1.390491000000

C 5.171750000000 -0.956150000000 -0.172748000000

C 5.422426000000 -0.446458000000 1.096673000000

C 5.972875000000 -1.278106000000 2.065709000000

C 6.281375000000 -2.611061000000 1.777159000000

C 6.033213000000 -3.093652000000 0.489639000000

C	5.480458000000	-2.274144000000	-0.487894000000	C	-4.214884000000	2.229829000000	1.555209000000
C	6.886220000000	-3.509626000000	2.825355000000	C	-5.198071000000	2.519480000000	2.691244000000
O	4.158831000000	-0.775373000000	-2.568364000000	C	-5.562362000000	-0.803765000000	-2.312304000000
O	5.151046000000	1.294603000000	-1.543143000000	C	-6.770511000000	-1.626451000000	-1.847490000000
C	1.004617000000	5.199615000000	-1.358311000000	H	-5.268881000000	-3.717530000000	2.185316000000
C	1.969465000000	5.257739000000	-0.188754000000	H	-4.259225000000	-2.796305000000	3.324174000000
C	2.982988000000	4.302149000000	-0.043160000000	H	-1.788497000000	-3.102387000000	3.028185000000
C	3.818325000000	4.335484000000	1.069271000000	H	-0.888333000000	-2.251569000000	0.902522000000
C	3.657172000000	5.319548000000	2.043173000000	H	-1.376315000000	-5.738545000000	2.525259000000
C	2.656164000000	6.275881000000	1.898888000000	H	-0.028233000000	-4.611639000000	2.221555000000
C	1.816607000000	6.242598000000	0.787087000000	H	-0.435270000000	-7.138152000000	0.210018000000
C	-3.025220000000	-0.801554000000	-0.028252000000	H	-0.690348000000	-7.099246000000	-2.262796000000
N	-2.937595000000	-1.976602000000	0.649272000000	H	-1.524938000000	-5.070970000000	-3.400017000000
C	-4.124423000000	-2.131981000000	1.317588000000	H	-2.072578000000	-3.032056000000	-2.096895000000
N	-4.949912000000	-1.161003000000	1.065274000000	H	3.413332000000	1.884096000000	-4.571707000000
N	-4.263213000000	-0.346241000000	0.206734000000	H	2.730975000000	3.898482000000	-3.285056000000
C	-4.297233000000	-3.238435000000	2.316727000000	H	0.128080000000	-0.344051000000	-2.965653000000
O	-3.306624000000	-4.216892000000	2.150879000000	H	2.083777000000	-0.186015000000	-4.451798000000
C	-1.984959000000	-3.693880000000	2.122627000000	H	1.371058000000	2.180945000000	0.257535000000
C	-1.784650000000	-2.876441000000	0.828045000000	H	-5.526087000000	4.056830000000	0.050054000000
C	-1.048868000000	-4.885472000000	1.926861000000	H	-6.643005000000	3.733314000000	-2.136936000000
C	-1.123250000000	-5.109653000000	0.434739000000	H	-6.631324000000	1.509169000000	-3.226190000000
C	-1.581549000000	-3.961428000000	-0.213895000000	H	-3.495564000000	3.053237000000	1.449245000000
C	-0.792772000000	-6.244186000000	-0.292776000000	H	-3.620546000000	1.350739000000	1.814729000000
C	-0.938955000000	-6.218705000000	-1.679022000000	H	-5.887377000000	1.681410000000	2.828482000000
C	-1.410717000000	-5.074595000000	-2.321049000000	H	-5.791164000000	3.414377000000	2.483891000000
C	-1.732831000000	-3.929325000000	-1.592981000000	H	-4.652937000000	2.682255000000	3.624676000000
C	-4.919107000000	0.776768000000	-0.416342000000	H	-4.637398000000	-1.354838000000	-2.109275000000
C	-4.906547000000	2.014396000000	0.231440000000	H	-5.605028000000	-0.665612000000	-3.396720000000
C	-5.543107000000	3.075740000000	-0.415420000000	H	-7.701211000000	-1.108885000000	-2.094935000000
C	-6.162489000000	2.893886000000	-1.645114000000	H	-6.746772000000	-1.772998000000	-0.764462000000
C	-6.154836000000	1.645526000000	-2.259624000000	H	-6.782028000000	-2.605884000000	-2.332014000000
C	-5.527673000000	0.556134000000	-1.658380000000	H	-1.179523000000	0.100248000000	2.174025000000

H	-2.295065000000	0.904957000000	4.214705000000
H	-1.946729000000	3.235289000000	4.996178000000
H	-0.449266000000	4.750122000000	3.706698000000
H	0.678705000000	3.938992000000	1.672870000000
H	2.621890000000	-1.865030000000	2.053690000000
H	2.659655000000	-4.346980000000	2.179378000000
H	2.271861000000	-5.695195000000	0.125726000000
H	1.913023000000	-4.557337000000	-2.046551000000
H	1.979612000000	-2.072428000000	-2.176395000000
H	5.165799000000	0.584547000000	1.322342000000
H	6.162933000000	-0.890786000000	3.063408000000
H	6.262245000000	-4.130470000000	0.255856000000
H	5.250792000000	-2.642611000000	-1.482410000000
H	7.962866000000	-3.627683000000	2.664573000000
H	6.440032000000	-4.507608000000	2.793278000000
H	6.743212000000	-3.102025000000	3.828849000000
H	3.121321000000	3.506769000000	-0.773696000000
H	4.594186000000	3.582563000000	1.164595000000
H	4.306080000000	5.338016000000	2.913007000000
H	2.520065000000	7.042345000000	2.655673000000
H	1.019599000000	6.974857000000	0.685953000000
H	0.146041000000	5.848405000000	-1.165070000000
H	1.478092000000	5.534472000000	-2.287511000000
H	-2.488497000000	1.767284000000	-0.190210000000
H	0.428488000000	-0.601733000000	1.120002000000

----

^{SS}INTa7

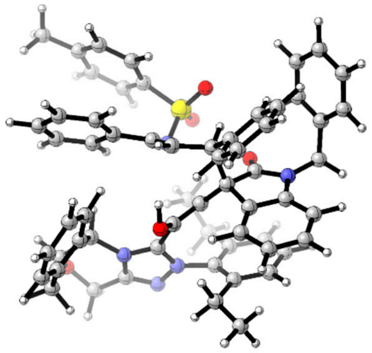

Zero-point correction= 1.044268 (Hartree/Particle)

Thermal correction to Energy= 1.104410

Thermal correction to Enthalpy= 1.105354

Thermal correction to Gibbs Free Energy= 0.948346

Sum of electronic and zero-point Energies= -3403.353558

Sum of electronic and thermal Energies= -3403.293416

Sum of electronic and thermal Enthalpies= -3403.292471

Sum of electronic and thermal Free Energies= -3403.449480

E(RM062X) = -3405.14570237

C	1.661294000000	-0.572980000000	0.195850000000
C	1.602096000000	0.820033000000	1.046732000000
C	0.366952000000	-1.000569000000	-0.410418000000
C	-0.694268000000	-1.554601000000	0.184734000000
C	2.450827000000	-1.667287000000	0.892118000000
C	3.764549000000	-1.643143000000	0.383199000000
C	4.758361000000	-2.473493000000	0.876220000000
C	4.412744000000	-3.372197000000	1.890125000000
C	3.115226000000	-3.430076000000	2.384906000000
C	2.126011000000	-2.569846000000	1.888270000000
H	2.866501000000	-4.135192000000	3.171347000000
H	5.173689000000	-4.032386000000	2.293936000000
H	5.771144000000	-2.432839000000	0.489858000000
N	3.839700000000	-0.745578000000	-0.684552000000
H	1.119160000000	-2.605397000000	2.290904000000
C	2.592350000000	-0.247662000000	-0.983600000000

C	4.997466000000	-0.352626000000	-1.475278000000
O	2.302182000000	0.345243000000	-1.997823000000
H	0.319748000000	-0.857252000000	-1.481067000000
C	-1.680426000000	-2.210496000000	-0.673957000000
O	-0.898011000000	-1.709916000000	1.531004000000
N	-1.336618000000	-3.017158000000	-1.690271000000
N	-3.034840000000	-2.202017000000	-0.659302000000
C	0.018701000000	-3.301628000000	-2.109478000000
N	-2.416563000000	-3.488542000000	-2.356098000000
C	-3.432474000000	-2.981054000000	-1.722043000000
C	-4.870240000000	-3.166020000000	-2.090391000000
C	-3.979124000000	-1.292108000000	0.066284000000
C	0.493852000000	-2.659575000000	-3.256181000000
C	0.811680000000	-4.056257000000	-1.237350000000
O	-5.628447000000	-2.072615000000	-1.637778000000
H	-4.951155000000	-3.199309000000	-3.177803000000
H	-5.244280000000	-4.116962000000	-1.688395000000
C	-5.417689000000	-1.759871000000	-0.283335000000
C	-5.715877000000	-2.872155000000	0.747618000000
H	-6.069904000000	-0.908102000000	-0.074790000000
C	-4.013818000000	-1.416751000000	1.570635000000
H	-3.750878000000	-0.280491000000	-0.278337000000
C	-5.001063000000	-2.327662000000	1.956955000000
H	-6.788200000000	-3.015436000000	0.897333000000
H	-5.290059000000	-3.834701000000	0.437625000000
C	-5.273197000000	-2.538711000000	3.303029000000
C	-3.351041000000	-0.639707000000	2.511677000000
C	-4.565904000000	-1.802604000000	4.251988000000
H	-6.042088000000	-3.241218000000	3.609988000000
C	-3.627999000000	-0.846901000000	3.859770000000
H	-4.772513000000	-1.950164000000	5.307266000000
H	-3.123843000000	-0.243779000000	4.607753000000
H	-2.655642000000	0.131539000000	2.198686000000
C	1.869819000000	-2.734267000000	-3.482045000000
C	-0.401219000000	-1.867059000000	-4.184399000000
C	2.704454000000	-3.411907000000	-2.600613000000
H	2.284831000000	-2.220349000000	-4.343666000000
C	2.180191000000	-4.088595000000	-1.502078000000
H	3.776474000000	-3.422708000000	-2.773256000000
H	2.845259000000	-4.620740000000	-0.829403000000
C	0.197722000000	-4.745655000000	-0.038136000000
C	1.045137000000	-5.871461000000	0.547227000000
H	0.008457000000	-3.998224000000	0.744401000000
H	-0.783181000000	-5.140672000000	-0.329432000000
C	-0.794122000000	-0.476833000000	-3.662928000000
H	0.139363000000	-1.745768000000	-5.127819000000
H	-1.296802000000	-2.454224000000	-4.412558000000
H	-1.340203000000	0.068332000000	-4.436549000000
H	-1.446550000000	-0.522359000000	-2.782423000000
H	0.070730000000	0.134366000000	-3.388257000000
H	0.492002000000	-6.383406000000	1.337948000000
H	1.307708000000	-6.606888000000	-0.218564000000
H	1.967772000000	-5.483494000000	0.986100000000
C	5.478232000000	1.016581000000	-1.045849000000
C	4.755616000000	2.153136000000	-1.429381000000
C	6.559995000000	1.150588000000	-0.177031000000
C	5.108492000000	3.403445000000	-0.930998000000
H	3.888204000000	2.058391000000	-2.076294000000
C	6.192134000000	3.532611000000	-0.063407000000
H	4.513884000000	4.267348000000	-1.208677000000
C	6.922009000000	2.405688000000	0.307741000000
H	6.465022000000	4.508425000000	0.326102000000
H	7.767359000000	2.500679000000	0.982845000000
H	7.115264000000	0.268052000000	0.132759000000
H	4.676114000000	-0.340560000000	-2.521338000000
H	5.777014000000	-1.109507000000	-1.356561000000

C	0.210190000000	1.209949000000	1.468107000000
C	-0.679415000000	1.681502000000	0.557385000000
N	-0.455846000000	1.563666000000	-0.810227000000
C	2.581227000000	0.828014000000	2.199549000000
C	2.297879000000	0.189361000000	3.411315000000
C	3.218647000000	0.184172000000	4.451416000000
C	4.446597000000	0.825526000000	4.297025000000
C	4.736737000000	1.472987000000	3.101178000000
C	3.809454000000	1.476078000000	2.060862000000
C	-2.008449000000	2.209408000000	1.004525000000
C	-3.090435000000	2.177658000000	0.117421000000
C	-4.348001000000	2.626382000000	0.510240000000
C	-4.545873000000	3.119328000000	1.798464000000
C	-3.469305000000	3.181805000000	2.681079000000
C	-2.211084000000	2.740327000000	2.285456000000
S	0.183863000000	2.785548000000	-1.605917000000
C	-0.998212000000	4.124812000000	-1.429183000000
C	-0.983021000000	4.905230000000	-0.277051000000
C	-2.038504000000	5.774544000000	-0.026542000000
C	-3.107444000000	5.883719000000	-0.920371000000
C	-3.079878000000	5.123521000000	-2.092607000000
C	-2.035593000000	4.240166000000	-2.347510000000
C	-4.279270000000	6.775326000000	-0.600316000000
O	1.428477000000	3.291033000000	-0.992712000000
O	0.210769000000	2.453033000000	-3.032029000000
H	1.920644000000	1.577965000000	0.324319000000
H	-0.023812000000	1.230542000000	2.531344000000
H	1.347185000000	-0.324530000000	3.538539000000
H	2.981258000000	-0.323420000000	5.381334000000
H	5.168596000000	0.821782000000	5.107742000000
H	5.685369000000	1.983527000000	2.966580000000
H	4.036235000000	1.999173000000	1.135502000000
H	-2.903017000000	1.825496000000	-0.893561000000

H	-5.172222000000	2.607053000000	-0.197639000000
H	-5.525316000000	3.471797000000	2.106357000000
H	-3.606011000000	3.596202000000	3.675387000000
H	-1.368237000000	2.838541000000	2.963256000000
H	-0.152343000000	4.809022000000	0.416180000000
H	-2.040871000000	6.371853000000	0.881988000000
H	-3.893497000000	5.218784000000	-2.807703000000
H	-2.009881000000	3.627389000000	-3.243560000000
H	-4.957265000000	6.276709000000	0.101738000000
H	-4.850687000000	7.022805000000	-1.498113000000
H	-3.953746000000	7.708877000000	-0.133513000000
H	-0.667344000000	-0.851020000000	1.926927000000

TSa5

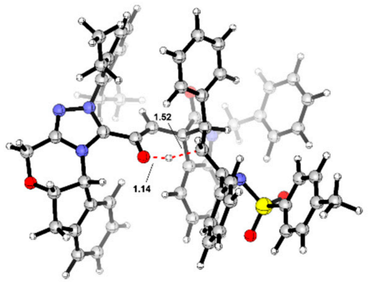

Frequency -716.3775

Zero-point correction= 1.038234 (Hartree/Particle)

Thermal correction to Energy= 1.098536

Thermal correction to Enthalpy= 1.099480

Thermal correction to Gibbs Free Energy= 0.940224

Sum of electronic and zero-point Energies= -3403.372046

Sum of electronic and thermal Energies= -3403.311744

Sum of electronic and thermal Enthalpies= -3403.310800

Sum of electronic and thermal Free Energies= -3403.470056

E(RM062X) = -3405.15708845

C	1.055370000000	-0.109128000000	0.927374000000
H	-0.239687000000	-0.892874000000	0.779235000000
O	-1.331367000000	-1.210489000000	0.808471000000
C	-2.098498000000	-0.252054000000	0.288049000000
C	-1.725501000000	0.818156000000	-0.438498000000
C	-0.329520000000	1.099104000000	-0.886349000000
C	0.718213000000	1.244565000000	0.327500000000
C	2.226894000000	-0.764763000000	0.467426000000
N	2.883605000000	-0.300878000000	-0.580307000000
C	0.247541000000	0.145625000000	-1.913513000000
C	0.896155000000	0.910120000000	-2.890160000000
C	1.673644000000	0.330259000000	-3.879352000000
C	1.765373000000	-1.062960000000	-3.894884000000
C	0.316354000000	-1.236261000000	-1.963621000000
C	1.075504000000	-1.839557000000	-2.970010000000
N	0.601339000000	2.270109000000	-2.693663000000
C	-0.262339000000	2.432891000000	-1.636124000000
O	-0.827648000000	3.469287000000	-1.341524000000
C	0.244576000000	2.247234000000	1.357770000000
C	-0.570065000000	1.870625000000	2.430614000000
C	-0.946761000000	2.796528000000	3.398611000000
C	-0.534591000000	4.122931000000	3.299157000000
C	0.265655000000	4.511443000000	2.228282000000
C	0.656222000000	3.579650000000	1.271774000000
C	2.621303000000	-1.991126000000	1.240097000000
C	3.062667000000	-1.878232000000	2.560552000000
C	3.440409000000	-3.012431000000	3.271508000000
C	3.350130000000	-4.269510000000	2.678245000000
C	2.889032000000	-4.386947000000	1.369107000000
C	2.532561000000	-3.252252000000	0.646013000000
S	4.259981000000	-0.959726000000	-1.177007000000
C	5.337385000000	-1.195683000000	0.232325000000
C	5.640058000000	-0.105918000000	1.042777000000
C	6.420677000000	-0.302905000000	2.175403000000
C	6.903585000000	-1.573639000000	2.503147000000
C	6.593274000000	-2.646800000000	1.664385000000
C	5.814130000000	-2.465798000000	0.527286000000
C	7.757127000000	-1.782786000000	3.727199000000
O	4.052012000000	-2.277687000000	-1.782588000000
O	4.865668000000	0.087821000000	-2.000280000000
C	1.295348000000	3.405284000000	-3.276538000000
C	2.453563000000	3.820227000000	-2.391936000000
C	2.497973000000	5.091936000000	-1.822329000000
C	3.540081000000	5.441307000000	-0.963539000000
C	4.539835000000	4.518331000000	-0.669452000000
C	4.494963000000	3.243466000000	-1.232586000000
C	3.455706000000	2.893783000000	-2.087280000000
H	2.399347000000	-1.543122000000	-4.632302000000
H	2.216442000000	0.929856000000	-4.602417000000
H	-0.171213000000	-1.844391000000	-1.209781000000
H	1.167131000000	-2.920235000000	-2.999911000000
H	1.623727000000	1.643719000000	-0.151722000000
H	-0.886446000000	0.833493000000	2.521900000000
H	-1.543056000000	2.473671000000	4.247676000000
H	-0.823937000000	4.844150000000	4.058096000000
H	0.600791000000	5.540424000000	2.141873000000
H	1.294371000000	3.884004000000	0.445024000000
H	3.142811000000	-0.893442000000	3.012327000000
H	3.810652000000	-2.913436000000	4.287405000000
H	3.642401000000	-5.155280000000	3.234012000000
H	2.816914000000	-5.364994000000	0.903234000000
H	2.213200000000	-3.328971000000	-0.388449000000
H	5.240629000000	0.875240000000	0.800547000000
H	6.654099000000	0.539273000000	2.821596000000
H	6.952918000000	-3.641372000000	1.915883000000
H	5.538285000000	-3.294875000000	-0.115992000000

H	8.820722000000	-1.779278000000	3.466421000000
H	7.539016000000	-2.744176000000	4.199992000000
H	7.594771000000	-0.993464000000	4.464964000000
H	1.705032000000	5.803990000000	-2.037440000000
H	3.565734000000	6.432752000000	-0.521429000000
H	5.350873000000	4.791815000000	-0.001443000000
H	5.262769000000	2.503241000000	-1.029037000000
H	3.435491000000	1.882014000000	-2.485135000000
H	0.570841000000	4.217944000000	-3.372460000000
H	1.630882000000	3.125457000000	-4.279441000000
H	-2.468444000000	1.549721000000	-0.727694000000
H	0.945729000000	-0.192084000000	2.010326000000
C	-3.533436000000	-0.494856000000	0.507973000000
N	-4.539167000000	0.375303000000	0.660577000000
N	-5.739976000000	-0.253401000000	0.819949000000
C	-5.448524000000	-1.519009000000	0.773801000000
N	-4.107352000000	-1.716873000000	0.587064000000
C	-6.363887000000	-2.690258000000	0.958390000000
O	-5.801374000000	-3.833598000000	0.368942000000
C	-4.506927000000	-4.137530000000	0.869364000000
C	-3.497968000000	-3.048223000000	0.435889000000
C	-4.029385000000	-5.382522000000	0.116395000000
C	-3.438722000000	-4.803096000000	-1.149475000000
C	-3.167527000000	-3.444115000000	-0.985985000000
C	-3.153164000000	-5.430906000000	-2.353543000000
C	-2.594582000000	-4.678128000000	-3.387312000000
C	-2.334083000000	-3.319199000000	-3.220426000000
C	-2.624181000000	-2.685265000000	-2.011817000000
C	-4.459696000000	1.810176000000	0.793029000000
C	-4.588199000000	2.598628000000	-0.353202000000
C	-4.450067000000	3.975898000000	-0.180991000000
C	-4.211974000000	4.514855000000	1.077008000000
C	-4.118616000000	3.697487000000	2.197567000000
C	-4.243946000000	2.314719000000	2.079643000000
C	-4.380051000000	2.037152000000	4.619560000000
C	-4.164230000000	2.754987000000	-2.862011000000
C	-4.134962000000	1.379081000000	3.264576000000
C	-4.826115000000	1.984665000000	-1.717692000000
H	-6.538493000000	-2.841599000000	2.034816000000
H	-7.321144000000	-2.493279000000	0.473801000000
H	-4.547163000000	-4.260216000000	1.960662000000
H	-2.601327000000	-3.054231000000	1.060184000000
H	-3.269464000000	-5.910412000000	0.703838000000
H	-4.865795000000	-6.064113000000	-0.055497000000
H	-3.364593000000	-6.486911000000	-2.492507000000
H	-2.363495000000	-5.156166000000	-4.333714000000
H	-1.885662000000	-2.746425000000	-4.025580000000
H	-2.411159000000	-1.627499000000	-1.889117000000
H	-4.506104000000	4.631112000000	-1.042506000000
H	-4.089414000000	5.587330000000	1.185466000000
H	-3.932025000000	4.140182000000	3.169280000000
H	-4.345481000000	1.287639000000	5.413291000000
H	-3.625869000000	2.793931000000	4.847547000000
H	-5.361024000000	2.519921000000	4.646239000000
H	-4.226616000000	2.179635000000	-3.788164000000
H	-4.654525000000	3.715235000000	-3.039962000000
H	-3.110058000000	2.952757000000	-2.641850000000
H	-4.856150000000	0.564255000000	3.136056000000
H	-3.138510000000	0.915395000000	3.250326000000
H	-4.451618000000	0.955044000000	-1.721822000000
H	-5.907033000000	1.907233000000	-1.889044000000

INTa8

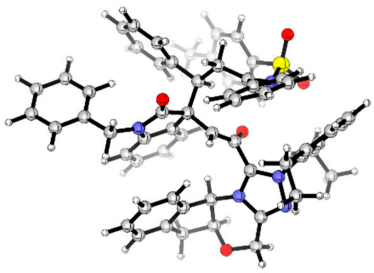

Zero-point correction= 1.044856 (Hartree/Particle)

Thermal correction to Energy= 1.105031

Thermal correction to Enthalpy= 1.105976

Thermal correction to Gibbs Free Energy= 0.950654

Sum of electronic and zero-point Energies= -3403.403580

Sum of electronic and thermal Energies= -3403.343404

Sum of electronic and thermal Enthalpies= -3403.342460

Sum of electronic and thermal Free Energies= -3403.497782

E(RM062X) = -3405.19412654

C	-0.205577000000	0.395153000000	0.709844000000
C	-1.481632000000	-2.176127000000	1.286000000000
N	-2.415974000000	-2.330785000000	0.419859000000
C	-0.021098000000	-2.594834000000	1.186547000000
C	0.919815000000	-1.878267000000	0.193984000000
C	1.092552000000	-0.354250000000	0.471605000000
C	1.859093000000	0.349645000000	-0.643324000000
C	2.948910000000	1.028317000000	-0.088121000000
C	3.797475000000	1.828789000000	-0.836776000000
C	3.530726000000	1.938417000000	-2.204226000000
C	1.630100000000	0.435080000000	-2.008958000000
C	2.471050000000	1.245426000000	-2.785285000000
N	3.016191000000	0.759009000000	1.285156000000
C	2.008760000000	-0.089322000000	1.678714000000
O	1.875478000000	-0.517925000000	2.812233000000
C	2.279891000000	-2.568626000000	0.138705000000
C	2.852291000000	-3.183554000000	1.258358000000
C	4.097353000000	-3.804760000000	1.179207000000
C	4.804630000000	-3.810633000000	-0.018490000000
C	4.264932000000	-3.171137000000	-1.132744000000
C	3.016420000000	-2.561514000000	-1.052994000000
C	-1.971170000000	-1.756263000000	2.643040000000
C	-3.240588000000	-2.190698000000	3.039648000000
C	-3.705749000000	-1.930917000000	4.324940000000
C	-2.916281000000	-1.218982000000	5.224635000000
C	-1.651252000000	-0.782730000000	4.835247000000
C	-1.172515000000	-1.063537000000	3.560080000000
S	-2.517138000000	-3.340252000000	-0.903746000000
C	-1.075796000000	-3.413844000000	-1.946025000000
C	-0.926017000000	-2.474376000000	-2.960181000000
C	0.183144000000	-2.566960000000	-3.794344000000
C	1.123803000000	-3.588480000000	-3.635468000000
C	0.927732000000	-4.535926000000	-2.625618000000
C	-0.171129000000	-4.459004000000	-1.780111000000
C	2.334238000000	-3.675298000000	-4.526979000000
O	-2.664506000000	-4.671081000000	-0.320402000000
O	-3.573233000000	-2.778614000000	-1.732735000000
C	4.070494000000	1.209690000000	2.162540000000
C	5.437811000000	0.679190000000	1.777779000000
C	5.579296000000	-0.526679000000	1.091575000000
C	6.848063000000	-1.000563000000	0.770983000000
C	7.982867000000	-0.279497000000	1.132479000000
C	7.844944000000	0.925907000000	1.815818000000
C	6.576736000000	1.403691000000	2.131107000000
C	-1.128705000000	0.508230000000	-0.285366000000
O	-1.225554000000	-0.083522000000	-1.400225000000
C	-2.068423000000	1.696129000000	-0.239988000000
N	-1.672250000000	2.886675000000	-0.740023000000
C	-2.796560000000	3.636446000000	-0.941182000000
N	-3.861469000000	2.991614000000	-0.560542000000

N	-3.385697000000	1.781634000000	-0.129835000000
C	-2.708933000000	4.947619000000	-1.668493000000
O	-1.366824000000	5.331042000000	-1.847452000000
C	-0.535641000000	4.300816000000	-2.369156000000
C	-0.356633000000	3.209803000000	-1.294256000000
C	0.871666000000	4.883341000000	-2.511745000000
C	1.427210000000	4.745776000000	-1.112288000000
C	0.679774000000	3.823632000000	-0.376574000000
C	2.514756000000	5.387456000000	-0.536990000000
C	2.829941000000	5.114441000000	0.794504000000
C	2.055910000000	4.224001000000	1.537370000000
C	0.973441000000	3.565709000000	0.953721000000
C	-4.298539000000	0.723172000000	0.229266000000
C	-4.820060000000	-0.052595000000	-0.808115000000
C	-5.745455000000	-1.034347000000	-0.452675000000
C	-6.137116000000	-1.199984000000	0.869112000000
C	-5.579519000000	-0.415180000000	1.873770000000
C	-4.619347000000	0.549202000000	1.576953000000
C	-4.447295000000	0.161279000000	-2.253643000000
C	-5.405493000000	1.133255000000	-2.952397000000
C	-3.981386000000	1.375998000000	2.665078000000
C	-4.678048000000	2.727728000000	2.855886000000
H	-3.203805000000	5.738640000000	-1.102187000000
H	-3.224806000000	4.834575000000	-2.633745000000
H	-0.958432000000	3.905160000000	-3.302902000000
H	0.024045000000	2.282742000000	-1.738774000000
H	0.832937000000	5.912849000000	-2.875139000000
H	1.451239000000	4.278599000000	-3.219822000000
H	3.100996000000	6.101509000000	-1.107762000000
H	3.671220000000	5.616014000000	1.262491000000
H	2.289961000000	4.043578000000	2.582100000000
H	0.376063000000	2.871687000000	1.531468000000
H	4.175529000000	2.556211000000	-2.821761000000
H	4.639398000000	2.338277000000	-0.379832000000
H	0.798019000000	-0.102955000000	-2.451400000000
H	2.302161000000	1.319489000000	-3.855542000000
H	0.473077000000	-1.921963000000	-0.802096000000
H	-6.133994000000	-1.686217000000	-1.228371000000
H	-6.864252000000	-1.965019000000	1.123114000000
H	-5.860489000000	-0.569265000000	2.911595000000
H	-4.472691000000	-0.816001000000	-2.742400000000
H	-3.409790000000	0.504306000000	-2.324395000000
H	-5.397017000000	2.114054000000	-2.468073000000
H	-6.431192000000	0.754695000000	-2.915398000000
H	-5.129940000000	1.260788000000	-4.003099000000
H	-2.918150000000	1.524536000000	2.441028000000
H	-4.013566000000	0.801267000000	3.595983000000
H	-5.727468000000	2.578428000000	3.124864000000
H	-4.654199000000	3.317347000000	1.935076000000
H	-4.198762000000	3.304903000000	3.650896000000
H	2.333212000000	-3.174722000000	2.210317000000
H	4.514295000000	-4.280396000000	2.061117000000
H	5.770950000000	-4.301971000000	-0.082176000000
H	4.815564000000	-3.147461000000	-2.068750000000
H	2.593818000000	-2.081271000000	-1.930358000000
H	-3.840281000000	-2.747874000000	2.327657000000
H	-4.683676000000	-2.294370000000	4.626786000000
H	-3.278783000000	-1.017544000000	6.228235000000
H	-1.023322000000	-0.236805000000	5.532182000000
H	-0.169956000000	-0.752811000000	3.286974000000
H	-1.654123000000	-1.678877000000	-3.062852000000
H	0.323002000000	-1.831597000000	-4.582573000000
H	1.655429000000	-5.331553000000	-2.491591000000
H	-0.345605000000	-5.200468000000	-1.007090000000
H	2.195431000000	-4.434010000000	-5.303889000000
H	3.218955000000	-3.955352000000	-3.947776000000

H	2.532745000000	-2.721116000000	-5.020705000000
H	4.699035000000	-1.093361000000	0.797300000000
H	6.941354000000	-1.938609000000	0.232804000000
H	8.970231000000	-0.651710000000	0.878408000000
H	8.723562000000	1.498268000000	2.096364000000
H	6.468084000000	2.350297000000	2.655776000000
H	3.788328000000	0.868350000000	3.163846000000
H	4.085743000000	2.307533000000	2.166033000000
H	-0.335288000000	0.954102000000	1.628315000000
H	-0.029206000000	-3.660813000000	0.920619000000
H	0.400926000000	-2.523029000000	2.189474000000

TSa6

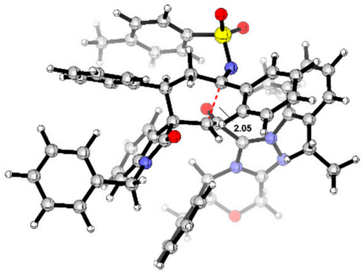

Frequency -314.4142

Zero-point correction= 1.044078 (Hartree/Particle)

Thermal correction to Energy= 1.103586

Thermal correction to Enthalpy= 1.104530

Thermal correction to Gibbs Free Energy= 0.948997

Sum of electronic and zero-point Energies= -3403.389321

Sum of electronic and thermal Energies= -3403.329813

Sum of electronic and thermal Enthalpies= -3403.328869

Sum of electronic and thermal Free Energies= -3403.484402

E(RM062X) = -3405.17716337

C	-0.359920000000	0.031755000000	0.622954000000
C	-1.234579000000	-1.756547000000	1.115377000000
N	-2.067930000000	-2.098672000000	0.103563000000
C	0.118275000000	-2.450354000000	1.318626000000
C	1.141784000000	-1.941553000000	0.302835000000
C	1.108476000000	-0.388124000000	0.414210000000
C	1.837555000000	0.317001000000	-0.721294000000
C	2.847089000000	1.124118000000	-0.181411000000
C	3.667676000000	1.928333000000	-0.957513000000
C	3.472991000000	1.894793000000	-2.339865000000
C	1.710852000000	0.238324000000	-2.102036000000
C	2.522576000000	1.049108000000	-2.907511000000
N	2.880511000000	0.973303000000	1.207861000000
C	1.914310000000	0.096038000000	1.638813000000
O	1.739172000000	-0.207914000000	2.804510000000
C	2.536479000000	-2.520234000000	0.432145000000
C	3.049368000000	-2.968152000000	1.654198000000
C	4.335367000000	-3.498548000000	1.735174000000
C	5.134714000000	-3.581676000000	0.599104000000
C	4.645708000000	-3.115078000000	-0.619928000000
C	3.358305000000	-2.593067000000	-0.699860000000
C	-1.958913000000	-1.420065000000	2.410451000000
C	-3.238381000000	-1.943570000000	2.596160000000
C	-3.902704000000	-1.781104000000	3.810813000000
C	-3.305875000000	-1.083509000000	4.853989000000
C	-2.023562000000	-0.561238000000	4.679172000000
C	-1.352436000000	-0.737711000000	3.475906000000
S	-2.164550000000	-3.532145000000	-0.600620000000
C	-0.814311000000	-3.719558000000	-1.763415000000
C	-0.757071000000	-2.884266000000	-2.875111000000
C	0.357422000000	-2.935424000000	-3.704382000000
C	1.412355000000	-3.816757000000	-3.441149000000
C	1.312951000000	-4.670918000000	-2.340949000000
C	0.204439000000	-4.628172000000	-1.500055000000
C	2.631648000000	-3.849305000000	-4.326538000000

O	-1.996846000000	-4.625339000000	0.366639000000
O	-3.382112000000	-3.512492000000	-1.415425000000
C	3.862447000000	1.571868000000	2.082911000000
C	5.282282000000	1.131028000000	1.788604000000
C	5.557204000000	-0.129062000000	1.258203000000
C	6.874313000000	-0.511231000000	1.020603000000
C	7.924394000000	0.355293000000	1.312619000000
C	7.652854000000	1.614540000000	1.840761000000
C	6.336098000000	2.000668000000	2.070960000000
C	-1.112274000000	0.348140000000	-0.530895000000
O	-1.041382000000	-0.185188000000	-1.633804000000
C	-2.121119000000	1.493550000000	-0.521120000000
N	-1.767892000000	2.669978000000	-1.075176000000
C	-2.902661000000	3.417783000000	-1.200055000000
N	-3.933565000000	2.773190000000	-0.735908000000
N	-3.429811000000	1.569835000000	-0.321387000000
C	-2.860578000000	4.723394000000	-1.940889000000
O	-1.534635000000	5.171713000000	-2.085568000000
C	-0.641511000000	4.201024000000	-2.616763000000
C	-0.456601000000	3.068498000000	-1.585642000000
C	0.745058000000	4.848210000000	-2.659095000000
C	1.214245000000	4.697226000000	-1.228984000000
C	0.482318000000	3.700398000000	-0.580863000000
C	2.192892000000	5.401055000000	-0.543688000000
C	2.404074000000	5.115623000000	0.806651000000
C	1.637308000000	4.151924000000	1.459278000000
C	0.670195000000	3.425029000000	0.763585000000
C	-4.341027000000	0.572720000000	0.198293000000
C	-4.739134000000	-0.463791000000	-0.650389000000
C	-5.688060000000	-1.353849000000	-0.145481000000
C	-6.243625000000	-1.166360000000	1.115128000000
C	-5.822116000000	-0.116199000000	1.923445000000
C	-4.829983000000	0.761860000000	1.492279000000
C	-4.270505000000	-0.583859000000	-2.077230000000
C	-5.125933000000	0.281002000000	-3.011871000000
C	-4.324953000000	1.849785000000	2.407418000000
C	-5.261082000000	3.062119000000	2.444238000000
H	-3.411393000000	5.491178000000	-1.395412000000
H	-3.341618000000	4.575308000000	-2.919141000000
H	-1.004010000000	3.834769000000	-3.586574000000
H	-0.005414000000	2.183980000000	-2.051750000000
H	0.681744000000	5.884428000000	-2.998385000000
H	1.392376000000	4.288546000000	-3.345062000000
H	2.767607000000	6.177458000000	-1.039540000000
H	3.155836000000	5.668884000000	1.360915000000
H	1.788841000000	3.963170000000	2.517479000000
H	0.079734000000	2.669800000000	1.272521000000
H	4.098269000000	2.511482000000	-2.978262000000
H	4.440719000000	2.542268000000	-0.507701000000
H	0.991319000000	-0.439975000000	-2.546244000000
H	2.422115000000	0.997417000000	-3.987176000000
H	0.780810000000	-2.156913000000	-0.709083000000
H	-5.977148000000	-2.200934000000	-0.759087000000
H	-6.993079000000	-1.861089000000	1.481661000000
H	-6.225045000000	0.002294000000	2.925571000000
H	-4.324269000000	-1.638897000000	-2.355645000000
H	-3.215919000000	-0.311376000000	-2.160164000000
H	-5.084705000000	1.338115000000	-2.727161000000
H	-6.174757000000	-0.026929000000	-2.974139000000
H	-4.781745000000	0.189344000000	-4.045570000000
H	-3.319126000000	2.163761000000	2.105435000000
H	-4.218190000000	1.418657000000	3.408958000000
H	-6.259319000000	2.764250000000	2.776860000000
H	-5.357552000000	3.502622000000	1.448372000000
H	-4.885631000000	3.824781000000	3.131374000000
H	2.443819000000	-2.905644000000	2.552094000000

H 4.710546000000 -3.849724000000 2.691074000000
H 6.132348000000 -4.005741000000 0.663799000000
H 5.263765000000 -3.162270000000 -1.511748000000
H 2.970868000000 -2.247696000000 -1.654856000000
H -3.704639000000 -2.478936000000 1.775940000000
H -4.894332000000 -2.207046000000 3.934470000000
H -3.822936000000 -0.959497000000 5.800862000000
H -1.536219000000 -0.028489000000 5.490131000000
H -0.338223000000 -0.364160000000 3.375991000000
H -1.565491000000 -2.184248000000 -3.058958000000
H 0.418029000000 -2.275277000000 -4.566820000000
H 2.127380000000 -5.356952000000 -2.122779000000
H 0.123103000000 -5.273819000000 -0.631241000000
H 2.522675000000 -4.594647000000 -5.121263000000
H 3.524002000000 -4.109473000000 -3.750265000000
H 2.801129000000 -2.879364000000 -4.801667000000
H 4.743833000000 -0.810897000000 1.020916000000
H 7.071017000000 -1.492966000000 0.601518000000
H 8.949739000000 0.053478000000 1.124024000000
H 8.464600000000 2.299191000000 2.065664000000
H 6.123065000000 2.988061000000 2.474898000000
H 3.565450000000 1.286044000000 3.097339000000
H 3.793956000000 2.663537000000 2.000635000000
H -0.551659000000 0.662434000000 1.485912000000
H -0.036306000000 -3.531477000000 1.232226000000
H 0.467364000000 -2.233732000000 2.329972000000

INTa9

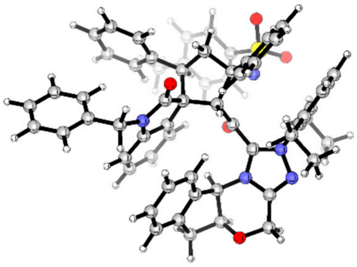

Zero-point correction= 1.044995 (Hartree/Particle)

Thermal correction to Energy= 1.104619

Thermal correction to Enthalpy= 1.105563

Thermal correction to Gibbs Free Energy= 0.951006

Sum of electronic and zero-point Energies= -3403.399186

Sum of electronic and thermal Energies= -3403.339563

Sum of electronic and thermal Enthalpies= -3403.338619

Sum of electronic and thermal Free Energies= -3403.493175

E(RM062X) = -3405.18748166

C -1.160404000000 0.322998000000 -0.711242000000
N -2.090802000000 -1.911508000000 -0.141519000000
C -1.151217000000 -1.615096000000 0.925150000000
C 0.099840000000 -2.524052000000 1.073947000000
C 1.147929000000 -1.979626000000 0.106606000000
C 1.051098000000 -0.434864000000 0.301966000000
C 1.817234000000 0.354373000000 -0.748048000000
C 2.813327000000 1.107501000000 -0.110318000000
C 3.689915000000 1.935797000000 -0.796160000000
C 3.576251000000 1.979756000000 -2.185837000000
C 1.788090000000 0.338947000000 -2.137859000000
C 2.654514000000 1.175452000000 -2.852278000000
N 2.775398000000 0.878985000000 1.266738000000
C 1.767616000000 -0.000094000000 1.597802000000
O 1.495678000000 -0.336068000000 2.733892000000
C 2.549230000000 -2.534157000000 0.244256000000
C 3.054666000000 -2.991974000000 1.465914000000

C	4.344477000000	-3.512004000000	1.551113000000
C	5.153892000000	-3.574347000000	0.420580000000
C	4.670799000000	-3.099495000000	-0.797634000000
C	3.378977000000	-2.588972000000	-0.882552000000
C	-1.875443000000	-1.550703000000	2.288065000000
C	-2.798893000000	-2.563590000000	2.574455000000
C	-3.424550000000	-2.633270000000	3.814280000000
C	-3.165030000000	-1.679323000000	4.794953000000
C	-2.242834000000	-0.674006000000	4.527386000000
C	-1.593641000000	-0.620413000000	3.293229000000
S	-2.308140000000	-3.338214000000	-0.773608000000
C	-0.973291000000	-3.604928000000	-1.955305000000
C	-0.850931000000	-2.728806000000	-3.030444000000
C	0.276197000000	-2.789966000000	-3.842086000000
C	1.290274000000	-3.723257000000	-3.595715000000
C	1.128527000000	-4.619563000000	-2.537407000000
C	0.000787000000	-4.569317000000	-1.721063000000
C	2.527813000000	-3.771065000000	-4.455276000000
O	-2.178941000000	-4.474829000000	0.161465000000
O	-3.531630000000	-3.287743000000	-1.588626000000
C	3.713253000000	1.413723000000	2.227756000000
C	5.151552000000	1.017451000000	1.958933000000
C	5.472349000000	-0.168335000000	1.298905000000
C	6.805529000000	-0.508931000000	1.087931000000
C	7.826564000000	0.325843000000	1.533122000000
C	7.509695000000	1.511840000000	2.190393000000
C	6.177511000000	1.856522000000	2.395666000000
C	-0.492045000000	-0.204514000000	0.519721000000
O	-0.924180000000	0.037346000000	-1.857456000000
C	-2.109647000000	1.526997000000	-0.616122000000
N	-1.774183000000	2.689321000000	-1.205608000000
C	-2.877279000000	3.491276000000	-1.167756000000
N	-3.870290000000	2.883967000000	-0.582656000000
N	-3.371289000000	1.660602000000	-0.239675000000
C	-2.851506000000	4.816486000000	-1.872690000000
O	-1.528330000000	5.271268000000	-2.025187000000
C	-0.648962000000	4.338148000000	-2.639144000000
C	-0.472405000000	3.117921000000	-1.713366000000
C	0.747058000000	4.970854000000	-2.619322000000
C	1.197206000000	4.706333000000	-1.198143000000
C	0.452273000000	3.662310000000	-0.647062000000
C	2.147117000000	5.361634000000	-0.429651000000
C	2.308431000000	4.982997000000	0.905366000000
C	1.520668000000	3.977406000000	1.462630000000
C	0.585326000000	3.298050000000	0.681491000000
C	-4.197783000000	0.663985000000	0.403191000000
C	-4.700584000000	-0.365186000000	-0.392371000000
C	-5.460853000000	-1.336802000000	0.258982000000
C	-5.716470000000	-1.241258000000	1.620755000000
C	-5.210408000000	-0.183964000000	2.372522000000
C	-4.413986000000	0.790082000000	1.778646000000
C	-4.500003000000	-0.410338000000	-1.885719000000
C	-5.486802000000	0.510888000000	-2.612997000000
C	-3.866004000000	1.939986000000	2.587729000000
C	-4.852567000000	3.110470000000	2.667919000000
H	-3.396106000000	5.564551000000	-1.295194000000
H	-3.346811000000	4.695441000000	-2.847837000000
H	-1.013388000000	4.064484000000	-3.638231000000
H	-0.022108000000	2.275279000000	-2.249517000000
H	0.697218000000	6.030833000000	-2.877281000000
H	1.395092000000	4.460279000000	-3.341850000000
H	2.734289000000	6.174466000000	-0.846112000000
H	3.034052000000	5.500416000000	1.525091000000
H	1.626176000000	3.722189000000	2.512318000000
H	-0.029866000000	2.518381000000	1.123812000000
H	4.244610000000	2.620146000000	-2.753201000000

H 4.444597000000 2.510554000000 -0.270241000000

H 1.103048000000 -0.316514000000 -2.664363000000

H 2.622444000000 1.173672000000 -3.937099000000

H 0.816308000000 -2.154115000000 -0.924656000000

H -5.806584000000 -2.189785000000 -0.315732000000

H -6.293580000000 -2.017434000000 2.113645000000

H -5.383642000000 -0.138817000000 3.443676000000

H -4.618391000000 -1.446883000000 -2.203955000000

H -3.467533000000 -0.149643000000 -2.143198000000

H -5.369447000000 1.555709000000 -2.306300000000

H -6.517283000000 0.220137000000 -2.390522000000

H -5.344321000000 0.451437000000 -3.695347000000

H -2.910879000000 2.284074000000 2.169045000000

H -3.640235000000 1.569862000000 3.591832000000

H -5.791167000000 2.781237000000 3.122114000000

H -5.080767000000 3.498147000000 1.671783000000

H -4.444784000000 3.923247000000 3.274979000000

H 2.439224000000 -2.947390000000 2.358166000000

H 4.715314000000 -3.871139000000 2.505773000000

H 6.154589000000 -3.990566000000 0.488570000000

H 5.295462000000 -3.134945000000 -1.685324000000

H 2.992204000000 -2.241212000000 -1.837469000000

H -3.009959000000 -3.314197000000 1.821280000000

H -4.128007000000 -3.437768000000 4.009172000000

H -3.660595000000 -1.729133000000 5.760054000000

H -2.004929000000 0.066177000000 5.286635000000

H -0.826809000000 0.134816000000 3.160003000000

H -1.626255000000 -1.987998000000 -3.201754000000

H 0.380733000000 -2.097865000000 -4.675214000000

H 1.907368000000 -5.350998000000 -2.335767000000

H -0.127875000000 -5.250119000000 -0.885567000000

H 2.429119000000 -4.517809000000 -5.250092000000

H 3.405136000000 -4.039296000000 -3.859186000000

H 2.718534000000 -2.804574000000 -4.929677000000

H 4.684001000000 -0.823983000000 0.937817000000

H 7.037337000000 -1.433007000000 0.568086000000

H 8.864452000000 0.057410000000 1.363924000000

H 8.298406000000 2.172308000000 2.536718000000

H 5.930425000000 2.787169000000 2.901863000000

H 3.377798000000 1.040434000000 3.200973000000

H 3.635390000000 2.508252000000 2.238007000000

H -0.655078000000 0.522596000000 1.317121000000

H -0.153124000000 -3.570626000000 0.895160000000

H 0.459145000000 -2.428694000000 2.100389000000

TSa7

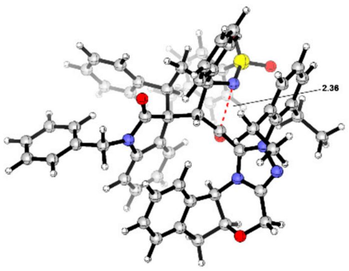

Frequency -46.4637

Zero-point correction= 1.045608 (Hartree/Particle)

Thermal correction to Energy= 1.104085

Thermal correction to Enthalpy= 1.105029

Thermal correction to Gibbs Free Energy= 0.954076

Sum of electronic and zero-point Energies= -3403.398428

Sum of electronic and thermal Energies= -3403.339951

Sum of electronic and thermal Enthalpies= -3403.339007

Sum of electronic and thermal Free Energies= -3403.489960

E(RM062X) = -3405.18699462

C -1.151174000000 0.264911000000 -0.723153000000

C -2.129854000000 1.451357000000 -0.614480000000

C	-0.436441000000	-0.206938000000	0.520697000000
C	-1.098420000000	-1.584975000000	0.948488000000
N	-2.110271000000	-1.793245000000	-0.090841000000
C	0.122234000000	-2.545278000000	1.003886000000
C	1.162756000000	-1.988181000000	0.031532000000
C	1.097948000000	-0.449500000000	0.274304000000
C	1.857303000000	0.368517000000	-0.760126000000
C	2.838927000000	1.126065000000	-0.106369000000
C	3.706847000000	1.978461000000	-0.773404000000
C	3.599494000000	2.044551000000	-2.162427000000
C	1.836927000000	0.375776000000	-2.150168000000
C	2.693635000000	1.236830000000	-2.846334000000
N	2.803712000000	0.868544000000	1.265682000000
C	1.834359000000	-0.060592000000	1.573770000000
O	1.605384000000	-0.464457000000	2.697087000000
C	2.557671000000	-2.566491000000	0.131541000000
C	3.087268000000	-3.034908000000	1.339050000000
C	4.375973000000	-3.561068000000	1.393324000000
C	5.161057000000	-3.619618000000	0.245355000000
C	4.652891000000	-3.137720000000	-0.959695000000
C	3.361586000000	-2.621877000000	-1.013765000000
C	-1.758783000000	-1.547442000000	2.340650000000
C	-2.696523000000	-2.542847000000	2.642995000000
C	-3.269026000000	-2.625866000000	3.906928000000
C	-2.939650000000	-1.703638000000	4.897489000000
C	-2.006819000000	-0.713741000000	4.611379000000
C	-1.412972000000	-0.645222000000	3.350013000000
S	-2.393562000000	-3.194619000000	-0.772502000000
C	-1.093644000000	-3.489940000000	-1.985051000000
C	-0.978095000000	-2.619859000000	-3.065001000000
C	0.127338000000	-2.708298000000	-3.904109000000
C	1.121959000000	-3.667934000000	-3.684182000000
C	0.961105000000	-4.564516000000	-2.625142000000
C	-0.142567000000	-4.484641000000	-1.780145000000
C	2.336971000000	-3.747406000000	-4.572905000000
O	-2.290761000000	-4.353621000000	0.135673000000
O	-3.624439000000	-3.069611000000	-1.564228000000
C	3.746250000000	1.383104000000	2.232088000000
C	5.180933000000	0.978186000000	1.955037000000
C	5.489628000000	-0.196199000000	1.268702000000
C	6.819298000000	-0.548571000000	1.054863000000
C	7.848728000000	0.263607000000	1.521957000000
C	7.544050000000	1.438643000000	2.204267000000
C	6.215502000000	1.794521000000	2.413648000000
O	-0.856296000000	0.047983000000	-1.872980000000
N	-1.793882000000	2.619758000000	-1.199144000000
C	-2.889138000000	3.429994000000	-1.140367000000
N	-3.876523000000	2.829153000000	-0.540728000000
N	-3.383280000000	1.599275000000	-0.213390000000
C	-2.876073000000	4.754707000000	-1.846819000000
O	-1.558203000000	5.211293000000	-2.031386000000
C	-0.688714000000	4.271467000000	-2.648937000000
C	-0.498021000000	3.060170000000	-1.715109000000
C	0.707995000000	4.902631000000	-2.653259000000
C	1.170053000000	4.659198000000	-1.232411000000
C	0.429815000000	3.622961000000	-0.659748000000
C	2.125504000000	5.327483000000	-0.482658000000
C	2.300524000000	4.969142000000	0.856298000000
C	1.517907000000	3.972715000000	1.435999000000
C	0.573361000000	3.283144000000	0.674140000000
C	-4.209687000000	0.635267000000	0.475630000000
C	-4.838124000000	-0.342035000000	-0.294077000000
C	-5.593019000000	-1.285278000000	0.401430000000
C	-5.721225000000	-1.211585000000	1.783733000000
C	-5.098322000000	-0.199261000000	2.507957000000
C	-4.304390000000	0.745905000000	1.864306000000

C	-4.754291000000	-0.354646000000	-1.798757000000
C	-5.741982000000	0.632195000000	-2.433243000000
C	-3.637936000000	1.857327000000	2.638509000000
C	-4.557010000000	3.073279000000	2.799982000000
H	-3.408894000000	5.503107000000	-1.258760000000
H	-3.393635000000	4.631215000000	-2.810257000000
H	-1.067046000000	3.988443000000	-3.640276000000
H	-0.040375000000	2.221260000000	-2.248884000000
H	0.658090000000	5.958590000000	-2.927177000000
H	1.348667000000	4.378372000000	-3.372731000000
H	2.707325000000	6.134659000000	-0.917344000000
H	3.032375000000	5.495809000000	1.460718000000
H	1.634016000000	3.733258000000	2.488333000000
H	-0.041069000000	2.514021000000	1.134306000000
H	4.260618000000	2.704601000000	-2.715656000000
H	4.450574000000	2.555131000000	-0.234114000000
H	1.162527000000	-0.278552000000	-2.691102000000
H	2.667279000000	1.251744000000	-3.931238000000
H	0.812681000000	-2.126191000000	-0.999794000000
H	-6.047815000000	-2.097753000000	-0.155674000000
H	-6.297299000000	-1.966612000000	2.309486000000
H	-5.180852000000	-0.166704000000	3.590347000000
H	-4.949394000000	-1.371895000000	-2.136038000000
H	-3.726999000000	-0.141008000000	-2.119048000000
H	-5.558643000000	1.661498000000	-2.109738000000
H	-6.767483000000	0.378744000000	-2.149932000000
H	-5.674134000000	0.595581000000	-3.523934000000
H	-2.702505000000	2.159527000000	2.148228000000
H	-3.350481000000	1.465677000000	3.618013000000
H	-5.466826000000	2.789291000000	3.335780000000
H	-4.853343000000	3.470737000000	1.825718000000
H	-4.060233000000	3.866464000000	3.365143000000
H	2.494224000000	-2.986377000000	2.245947000000
H	4.766041000000	-3.925659000000	2.338257000000
H	6.161975000000	-4.038563000000	0.289480000000
H	5.257924000000	-3.171336000000	-1.860889000000
H	2.956331000000	-2.265651000000	-1.957948000000
H	-2.966851000000	-3.263792000000	1.879915000000
H	-3.986674000000	-3.414801000000	4.113467000000
H	-3.392170000000	-1.765281000000	5.882801000000
H	-1.718809000000	0.003989000000	5.374759000000
H	-0.641909000000	0.100838000000	3.192576000000
H	-1.742096000000	-1.865083000000	-3.223320000000
H	0.228083000000	-2.018139000000	-4.739077000000
H	1.722681000000	-5.319692000000	-2.446807000000
H	-0.270635000000	-5.167864000000	-0.946650000000
H	2.207657000000	-4.504386000000	-5.353441000000
H	3.224360000000	-4.020510000000	-3.994039000000
H	2.530768000000	-2.791193000000	-5.066183000000
H	4.695233000000	-0.834788000000	0.889763000000
H	7.041969000000	-1.463835000000	0.515785000000
H	8.883890000000	-0.013746000000	1.350427000000
H	8.339528000000	2.081741000000	2.567389000000
H	5.977803000000	2.716032000000	2.940567000000
H	3.411399000000	0.997755000000	3.200713000000
H	3.673643000000	2.477905000000	2.256487000000
H	-0.587830000000	0.550396000000	1.294028000000
H	-0.167073000000	-3.574578000000	0.787172000000
H	0.515248000000	-2.511853000000	2.021468000000

INTa10

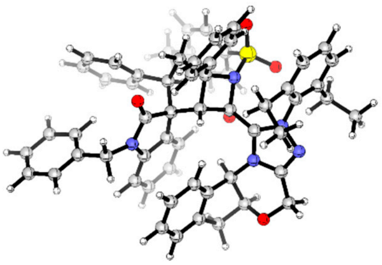

Zero-point correction= 1.046241 (Hartree/Particle)

Thermal correction to Energy= 1.105007

Thermal correction to Enthalpy= 1.105951

Thermal correction to Gibbs Free Energy= 0.954356

Sum of electronic and zero-point Energies= -3403.429666

Sum of electronic and thermal Energies= -3403.370900

Sum of electronic and thermal Enthalpies= -3403.369955

Sum of electronic and thermal Free Energies= -3403.521551

E(RM062X) = -3405.21630151

N	-3.569296000000	1.300662000000	-0.234446000000
N	-4.049476000000	2.505250000000	-0.682547000000
C	-2.996819000000	3.121659000000	-1.127625000000
N	-1.876194000000	2.362861000000	-0.958449000000
C	-2.954176000000	4.405437000000	-1.907529000000
O	-1.634132000000	4.835380000000	-2.126829000000
C	-0.761969000000	3.811355000000	-2.592650000000
C	-0.572731000000	2.763728000000	-1.481866000000
C	0.630464000000	4.436560000000	-2.710093000000
C	1.122841000000	4.392042000000	-1.280060000000
C	0.365133000000	3.483156000000	-0.535005000000
C	2.128494000000	5.128721000000	-0.674539000000
C	2.354147000000	4.966441000000	0.694187000000
C	1.555649000000	4.106447000000	1.445258000000
C	0.547107000000	3.360094000000	0.832998000000
C	-4.521818000000	0.399861000000	0.368587000000
C	3.587048000000	2.081468000000	-2.371155000000
C	3.786804000000	2.066563000000	-0.991011000000
C	2.967039000000	1.237615000000	-0.240146000000
C	1.942033000000	0.461996000000	-0.796656000000
C	1.809466000000	0.435883000000	-2.179339000000
C	2.627098000000	1.261261000000	-2.960086000000
N	3.047256000000	1.003056000000	1.136979000000
C	2.146473000000	0.045688000000	1.528828000000
C	1.270330000000	-0.327855000000	0.315314000000
C	1.261411000000	-1.870283000000	0.076321000000
C	0.292164000000	-2.399668000000	1.139929000000
C	-0.823559000000	-1.350197000000	1.268421000000
N	-1.850335000000	-1.290943000000	0.179674000000
O	2.062702000000	-0.394987000000	2.662252000000
C	-5.437464000000	-0.239076000000	-0.469091000000
C	-6.389786000000	-1.052204000000	0.145823000000
C	-6.428718000000	-1.192112000000	1.527945000000
C	-5.518994000000	-0.510509000000	2.328819000000
C	-4.541851000000	0.306256000000	1.761871000000
C	-5.460195000000	-0.038919000000	-1.963791000000
C	-6.530174000000	0.975092000000	-2.381239000000
C	-3.614424000000	1.115547000000	2.634025000000
C	-4.144181000000	2.537802000000	2.850231000000
C	2.638136000000	-2.495999000000	0.043203000000
C	3.297325000000	-2.929593000000	1.198508000000
C	4.578268000000	-3.473000000000	1.124404000000
C	5.226603000000	-3.582904000000	-0.102354000000
C	4.587019000000	-3.139975000000	-1.258467000000
C	3.302528000000	-2.609337000000	-1.184157000000
C	-1.445116000000	-1.355786000000	2.655062000000
C	-2.607254000000	-2.086620000000	2.907949000000
C	-3.138404000000	-2.140184000000	4.195344000000
C	-2.525932000000	-1.459121000000	5.243252000000
C	-1.366395000000	-0.727806000000	4.995093000000

C	-0.826885000000	-0.680707000000	3.713161000000	H	4.566933000000	2.657721000000	-0.523579000000
C	-1.154999000000	-3.242198000000	-1.792365000000	H	1.050773000000	-0.193200000000	-2.630163000000
C	-0.820966000000	-2.604206000000	-2.982721000000	H	2.516019000000	1.251703000000	-4.040028000000
C	0.237242000000	-3.103185000000	-3.734957000000	H	0.811391000000	-1.989619000000	-0.915913000000
C	0.954388000000	-4.228733000000	-3.318423000000	H	-7.101972000000	-1.584368000000	-0.478418000000
C	0.553160000000	-4.886661000000	-2.151785000000	H	-7.174092000000	-1.835880000000	1.984131000000
C	-0.506640000000	-4.407550000000	-1.391751000000	H	-5.548240000000	-0.613479000000	3.409721000000
C	2.151905000000	-4.720545000000	-4.086691000000	H	-5.648751000000	-1.009832000000	-2.429592000000
S	-2.429223000000	-2.548840000000	-0.747250000000	H	-4.472579000000	0.269191000000	-2.316945000000
O	-2.785985000000	-3.611945000000	0.188145000000	H	-6.334574000000	1.950956000000	-1.929297000000
O	-3.415002000000	-1.948349000000	-1.633630000000	H	-7.522731000000	0.647587000000	-2.057866000000
C	5.466264000000	1.094237000000	1.658226000000	H	-6.549664000000	1.090888000000	-3.468429000000
C	5.701086000000	-0.090665000000	0.960955000000	H	-2.612245000000	1.153889000000	2.193584000000
C	7.005501000000	-0.489434000000	0.682171000000	H	-3.497855000000	0.602786000000	3.593703000000
C	8.083852000000	0.288062000000	1.094948000000	H	-5.123414000000	2.510146000000	3.336356000000
C	7.853260000000	1.473618000000	1.788184000000	H	-4.263389000000	3.063183000000	1.897915000000
C	6.550006000000	1.874943000000	2.063063000000	H	-3.463751000000	3.114640000000	3.482218000000
C	4.060858000000	1.545450000000	2.009322000000	H	2.820457000000	-2.825104000000	2.167227000000
C	-0.204071000000	-0.027334000000	0.713512000000	H	5.072393000000	-3.803806000000	2.032473000000
O	-0.891563000000	-0.128287000000	-1.704839000000	H	6.223497000000	-4.009820000000	-0.158210000000
C	-2.255811000000	1.174853000000	-0.421604000000	H	5.086139000000	-3.211194000000	-2.220552000000
C	-1.256614000000	-0.055158000000	-0.471748000000	H	2.796311000000	-2.276028000000	-2.087490000000
H	-3.474320000000	5.198244000000	-1.367043000000	H	-3.098544000000	-2.608271000000	2.093914000000
H	-3.479522000000	4.231438000000	-2.858831000000	H	-4.041088000000	-2.717434000000	4.374021000000
H	-1.145626000000	3.373643000000	-3.524133000000	H	-2.943907000000	-1.501606000000	6.244419000000
H	-0.119770000000	1.849464000000	-1.877367000000	H	-0.873628000000	-0.195202000000	5.802734000000
H	0.570020000000	5.447013000000	-3.121216000000	H	0.094022000000	-0.135923000000	3.538189000000
H	1.260484000000	3.821728000000	-3.362995000000	H	-1.359627000000	-1.711595000000	-3.273985000000
H	2.719927000000	5.834488000000	-1.250529000000	H	0.518428000000	-2.603988000000	-4.658508000000
H	3.135055000000	5.540415000000	1.183567000000	H	1.082784000000	-5.780489000000	-1.833881000000
H	1.709176000000	4.020671000000	2.516641000000	H	-0.836106000000	-4.921811000000	-0.495293000000
H	-0.097239000000	2.726628000000	1.432704000000	H	2.101903000000	-5.800625000000	-4.249163000000
H	4.210482000000	2.717709000000	-2.992099000000	H	3.068486000000	-4.516877000000	-3.521301000000

H 2.234325000000 -4.229208000000 -5.058444000000

H 4.867333000000 -0.702453000000 0.624649000000

H 7.170021000000 -1.413699000000 0.137193000000

H 9.099339000000 -0.024511000000 0.873425000000

H 8.687833000000 2.089403000000 2.108513000000

H 6.370001000000 2.804857000000 2.597855000000

H 3.791116000000 1.215145000000 3.017659000000

H 4.001848000000 2.641097000000 1.980593000000

H -0.290329000000 0.821083000000 1.391434000000

H -0.094906000000 -3.397338000000 0.923768000000

H 0.786156000000 -2.437494000000 2.113360000000

TSa8

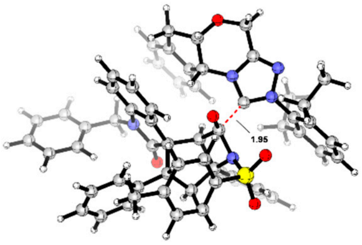

Frequency -200.2348

Zero-point correction= 1.044786 (Hartree/Particle)

Thermal correction to Energy= 1.103838

Thermal correction to Enthalpy= 1.104782

Thermal correction to Gibbs Free Energy= 0.951034

Sum of electronic and zero-point Energies= -3403.424521

Sum of electronic and thermal Energies= -3403.365469

Sum of electronic and thermal Enthalpies= -3403.364525

Sum of electronic and thermal Free Energies= -3403.518273

E(RM062X) = -3405.21069190

C -1.174712000000 -0.365337000000 -0.552155000000

C -2.387462000000 1.162641000000 -0.461203000000

N -1.935829000000 2.228478000000 -1.175115000000

C -3.001468000000 2.881698000000 -1.728888000000

N -4.119746000000 2.323155000000 -1.374863000000

N -3.710302000000 1.271318000000 -0.583307000000

C -2.781514000000 3.998759000000 -2.707748000000

O -1.460022000000 4.484366000000 -2.631650000000

C -0.474695000000 3.462853000000 -2.710124000000

C -0.554148000000 2.577741000000 -1.453746000000

C 0.895859000000 4.132415000000 -2.575586000000

C 1.056700000000 4.263589000000 -1.077400000000

C 0.160629000000 3.419694000000 -0.416395000000

C 1.908802000000 5.085267000000 -0.352989000000

C 1.833986000000 5.072993000000 1.041826000000

C 0.904733000000 4.266489000000 1.696092000000

C 0.050577000000 3.436104000000 0.967204000000

C -4.688278000000 0.449317000000 0.072845000000

C -5.439983000000 -0.438435000000 -0.699411000000

C -6.384328000000 -1.220520000000 -0.032512000000

C -6.562884000000 -1.110613000000 1.340297000000

C -5.796837000000 -0.212409000000 2.077291000000

C -4.838133000000 0.588116000000 1.458699000000

C -5.278212000000 -0.546851000000 -2.194937000000

C -6.309486000000 0.305813000000 -2.941620000000

C -4.024614000000 1.573198000000 2.265452000000

C -4.429435000000 3.030992000000 2.023535000000

O -1.033936000000 -0.450590000000 -1.777874000000

N -1.708556000000 -1.431189000000 0.289705000000

C -0.744806000000 -1.171449000000 1.407810000000

C 0.403480000000 -2.181002000000 1.574291000000

C 1.382549000000 -1.911853000000 0.420867000000

C 1.333595000000 -0.365447000000 0.244106000000

C 1.940080000000 0.155099000000 -1.043980000000

C 2.918296000000 1.105054000000 -0.725113000000

C	3.677478000000	1.753508000000	-1.689072000000
C	3.450057000000	1.406930000000	-3.022379000000
C	1.751784000000	-0.200437000000	-2.372458000000
C	2.506191000000	0.439563000000	-3.361484000000
N	3.015845000000	1.239522000000	0.664289000000
C	2.182844000000	0.357702000000	1.305803000000
O	2.120191000000	0.216146000000	2.514132000000
C	2.787738000000	-2.455488000000	0.560718000000
C	3.426243000000	-2.589566000000	1.797569000000
C	4.737244000000	-3.055702000000	1.873092000000
C	5.435305000000	-3.384230000000	0.714560000000
C	4.816225000000	-3.237013000000	-0.525361000000
C	3.502696000000	-2.782757000000	-0.598123000000
C	-1.442767000000	-0.820926000000	2.707077000000
C	-2.648476000000	-1.429740000000	3.057689000000
C	-3.243195000000	-1.152032000000	4.286542000000
C	-2.656276000000	-0.249478000000	5.168122000000
C	-1.462777000000	0.374710000000	4.813577000000
C	-0.853562000000	0.086317000000	3.595352000000
S	-2.321428000000	-2.866106000000	-0.294472000000
C	-1.015190000000	-3.698070000000	-1.173764000000
C	-0.650943000000	-3.271272000000	-2.447122000000
C	0.470583000000	-3.842151000000	-3.044509000000
C	1.216300000000	-4.828755000000	-2.393909000000
C	0.790294000000	-5.275508000000	-1.138003000000
C	-0.326435000000	-4.722549000000	-0.526908000000
C	2.464189000000	-5.401562000000	-3.011539000000
O	-2.612069000000	-3.686615000000	0.873881000000
O	-3.345296000000	-2.506010000000	-1.258298000000
C	3.992607000000	2.052245000000	1.352355000000
C	5.420296000000	1.605197000000	1.103318000000
C	5.723852000000	0.277576000000	0.799899000000
C	7.046572000000	-0.108805000000	0.604331000000
C	8.076056000000	0.822728000000	0.709190000000
C	7.777004000000	2.149286000000	1.008569000000
C	6.454281000000	2.537091000000	1.199131000000
C	-0.139449000000	-0.010762000000	0.551241000000
H	-3.451044000000	4.833815000000	-2.494990000000
H	-3.003868000000	3.621808000000	-3.717545000000
H	-0.599801000000	2.880162000000	-3.633372000000
H	-0.014955000000	1.642176000000	-1.639282000000
H	0.918258000000	5.088475000000	-3.104037000000
H	1.667539000000	3.474986000000	-2.994250000000
H	2.605507000000	5.745852000000	-0.861278000000
H	2.487712000000	5.716952000000	1.622148000000
H	0.833195000000	4.290867000000	2.778465000000
H	-0.702932000000	2.843802000000	1.481508000000
H	4.031497000000	1.889794000000	-3.801871000000
H	4.438885000000	2.475287000000	-1.412476000000
H	1.009902000000	-0.946015000000	-2.633607000000
H	2.358098000000	0.172430000000	-4.402429000000
H	0.964771000000	-2.318658000000	-0.507951000000
H	-6.970346000000	-1.933014000000	-0.606271000000
H	-7.297007000000	-1.733313000000	1.841885000000
H	-5.931254000000	-0.129758000000	3.152077000000
H	-5.384280000000	-1.600064000000	-2.468902000000
H	-4.260927000000	-0.263263000000	-2.480904000000
H	-6.190162000000	1.362198000000	-2.689201000000
H	-7.326857000000	0.005498000000	-2.672694000000
H	-6.196577000000	0.189847000000	-4.023242000000
H	-2.959305000000	1.435530000000	2.048478000000
H	-4.144482000000	1.327700000000	3.324550000000
H	-5.484048000000	3.183549000000	2.269369000000
H	-4.290262000000	3.321612000000	0.978732000000
H	-3.832081000000	3.702054000000	2.646866000000
H	2.908011000000	-2.310608000000	2.708540000000

H	5.214324000000	-3.155900000000	2.842750000000
H	6.455301000000	-3.751262000000	0.776845000000
H	5.354233000000	-3.478996000000	-1.437221000000
H	3.015893000000	-2.674991000000	-1.565131000000
H	-3.120574000000	-2.126365000000	2.374346000000
H	-4.174927000000	-1.645103000000	4.547686000000
H	-3.124681000000	-0.033022000000	6.123413000000
H	-0.996097000000	1.082991000000	5.491288000000
H	0.096076000000	0.549107000000	3.342389000000
H	-1.214316000000	-2.480833000000	-2.929660000000
H	0.778349000000	-3.507282000000	-4.031526000000
H	1.347282000000	-6.059492000000	-0.632417000000
H	-0.668411000000	-5.064088000000	0.444280000000
H	2.390170000000	-6.488148000000	-3.112999000000
H	3.330450000000	-5.188409000000	-2.376009000000
H	2.651109000000	-4.977382000000	-4.000305000000
H	4.928211000000	-0.458146000000	0.705666000000
H	7.264831000000	-1.145061000000	0.365594000000
H	9.106076000000	0.518364000000	0.553159000000
H	8.572014000000	2.884151000000	1.087556000000
H	6.220312000000	3.574945000000	1.425430000000
H	3.742525000000	1.975658000000	2.415527000000
H	3.861403000000	3.098105000000	1.049916000000
H	-0.244971000000	0.984121000000	0.970425000000
H	0.048651000000	-3.213725000000	1.613852000000
H	0.875257000000	-1.948390000000	2.531842000000

----

a

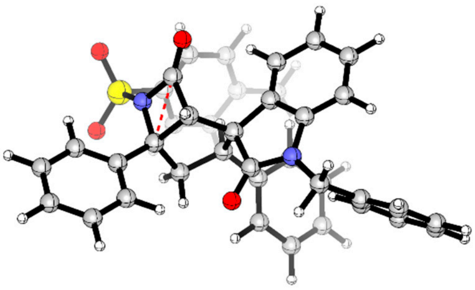

Zero-point correction= 0.629032 (Hartree/Particle)

Thermal correction to Energy= 0.666017

Thermal correction to Enthalpy= 0.666961

Thermal correction to Gibbs Free Energy= 0.560085

Sum of electronic and zero-point Energies= -2312.611748

Sum of electronic and thermal Energies= -2312.574762

Sum of electronic and thermal Enthalpies= -2312.573818

Sum of electronic and thermal Free Energies= -2312.680694

E(RM062X) = -2313.72993866

C	2.165117000000	0.137775000000	1.795125000000
O	2.196630000000	-0.572284000000	2.762053000000
N	3.003912000000	0.214069000000	0.684138000000
C	2.183550000000	1.241487000000	-0.025627000000
C	1.373376000000	0.671382000000	-1.191916000000
C	0.287259000000	-0.200919000000	-0.548592000000
C	-0.126974000000	0.604122000000	0.728861000000
C	-0.958724000000	-0.190455000000	1.708015000000
C	-2.222010000000	0.403127000000	1.805538000000
C	-3.236067000000	-0.125282000000	2.588842000000
C	-2.955982000000	-1.301584000000	3.287715000000
C	-0.704579000000	-1.369085000000	2.391039000000
C	-1.712915000000	-1.919313000000	3.188622000000
N	-2.271582000000	1.547848000000	0.994440000000
C	-1.085417000000	1.742558000000	0.335910000000
O	-0.852190000000	2.657933000000	-0.432696000000
C	-0.865491000000	-0.615399000000	-1.435875000000

C	-1.290797000000	0.155978000000	-2.521370000000
C	-2.347598000000	-0.269011000000	-3.323891000000
C	-3.003606000000	-1.464514000000	-3.047361000000
C	-2.607378000000	-2.228694000000	-1.950929000000
C	-1.544468000000	-1.808352000000	-1.156746000000
C	2.930029000000	2.528142000000	-0.292746000000
C	4.307405000000	2.632213000000	-0.103636000000
C	4.960958000000	3.836590000000	-0.353480000000
C	4.246164000000	4.947438000000	-0.786829000000
C	2.868444000000	4.850026000000	-0.967286000000
C	2.211690000000	3.649387000000	-0.722779000000
S	4.088915000000	-0.942290000000	0.086872000000
C	3.022177000000	-2.325651000000	-0.217212000000
C	2.529241000000	-3.043678000000	0.868359000000
C	1.532564000000	-3.988920000000	0.638315000000
C	1.040936000000	-4.222920000000	-0.648939000000
C	1.590361000000	-3.512038000000	-1.722869000000
C	2.581440000000	-2.562015000000	-1.517323000000
C	-0.066730000000	-5.212778000000	-0.892930000000
O	4.536603000000	-0.409744000000	-1.186353000000
O	4.997164000000	-1.263203000000	1.165570000000
C	-3.419577000000	2.414546000000	0.841708000000
C	-4.632870000000	1.708976000000	0.269560000000
C	-4.496996000000	0.612585000000	-0.581463000000
C	-5.627036000000	-0.003011000000	-1.111171000000
C	-6.898808000000	0.469521000000	-0.798961000000
C	-7.038220000000	1.562137000000	0.052570000000
C	-5.908963000000	2.174546000000	0.587807000000
C	1.216815000000	1.194629000000	1.218821000000
H	-3.727778000000	-1.742386000000	3.910252000000
H	-4.213054000000	0.343469000000	2.637302000000
H	0.263019000000	-1.852897000000	2.319458000000
H	-1.520112000000	-2.835283000000	3.736465000000

H	0.742088000000	-1.125822000000	-0.169519000000
H	-0.803290000000	1.098975000000	-2.744536000000
H	-2.658290000000	0.339956000000	-4.166531000000
H	-3.822433000000	-1.796330000000	-3.678281000000
H	-3.124656000000	-3.153766000000	-1.714008000000
H	-1.229554000000	-2.408447000000	-0.305492000000
H	4.879016000000	1.776535000000	0.237141000000
H	6.034415000000	3.900460000000	-0.207875000000
H	4.757591000000	5.884968000000	-0.980224000000
H	2.298968000000	5.713778000000	-1.295708000000
H	1.134660000000	3.582007000000	-0.846616000000
H	2.896821000000	-2.837483000000	1.869001000000
H	1.122056000000	-4.546065000000	1.475902000000
H	1.219267000000	-3.693391000000	-2.727722000000
H	3.004456000000	-1.992986000000	-2.339034000000
H	0.291373000000	-6.062643000000	-1.481610000000
H	-0.879140000000	-4.741160000000	-1.455124000000
H	-0.472722000000	-5.596021000000	0.045332000000
H	-3.507875000000	0.230696000000	-0.821299000000
H	-5.505827000000	-0.858836000000	-1.767927000000
H	-7.777928000000	-0.014299000000	-1.212297000000
H	-8.025787000000	1.933426000000	0.307264000000
H	-6.018886000000	3.021576000000	1.260991000000
H	-3.090586000000	3.222721000000	0.180615000000
H	-3.670121000000	2.856088000000	1.814280000000
H	1.124237000000	2.109490000000	1.805838000000
H	2.004718000000	0.127991000000	-1.899908000000
H	0.922811000000	1.516018000000	-1.715985000000

----

^{sr}INTa6'

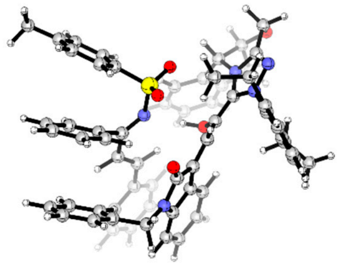

Zero-point correction= 1.040704 (Hartree/Particle)

Thermal correction to Energy= 1.102442

Thermal correction to Enthalpy= 1.103386

Thermal correction to Gibbs Free Energy= 0.942463

Sum of electronic and zero-point Energies= -3403.385369

Sum of electronic and thermal Energies= -3403.323632

Sum of electronic and thermal Enthalpies= -3403.322688

Sum of electronic and thermal Free Energies= -3403.483611

E(RM062X) = -3405.17470299

C	-0.117502000000	-1.404352000000	-1.519942000000
C	1.999419000000	0.531344000000	0.766179000000
C	-1.175795000000	-0.525427000000	-1.417739000000
C	-2.012168000000	-0.360728000000	-0.309679000000
C	0.228026000000	-2.641431000000	-0.837356000000
C	1.457538000000	-3.087287000000	-1.389406000000
C	2.069406000000	-4.264897000000	-0.987617000000
C	1.407735000000	-5.059663000000	-0.045413000000
C	0.174379000000	-4.668793000000	0.468450000000
C	-0.418066000000	-3.461055000000	0.088422000000
H	-0.324757000000	-5.299991000000	1.197274000000
H	1.865233000000	-5.985790000000	0.288195000000
H	3.028678000000	-4.565899000000	-1.399498000000
N	1.870401000000	-2.179264000000	-2.360151000000'
H	-1.363084000000	-3.149572000000	0.518289000000
C	0.924604000000	-1.159282000000	-2.518854000000
C	3.036858000000	-2.267984000000	-3.200911000000
O	1.048486000000	-0.252900000000	-3.338318000000
H	-1.280722000000	0.175891000000	-2.244028000000
C	-3.177393000000	0.411125000000	-0.342100000000
O	-1.679855000000	-0.904788000000	0.913158000000
H	-0.870858000000	-0.434505000000	1.185104000000
N	-3.921610000000	0.682788000000	-1.459266000000
N	-3.873297000000	1.023884000000	0.683401000000
C	-3.856023000000	0.045358000000	-2.742688000000
N	-4.999979000000	1.491050000000	-1.185309000000
C	-4.931139000000	1.680720000000	0.086330000000
C	-5.876382000000	2.441311000000	0.950932000000
C	-3.647174000000	1.378739000000	2.112486000000
C	-3.615766000000	0.842673000000	-3.867919000000
C	-4.097288000000	-1.335928000000	-2.822364000000
O	-6.174241000000	1.633358000000	2.068834000000
H	-5.439686000000	3.403968000000	1.256303000000
H	-6.803239000000	2.629051000000	0.408905000000
C	-5.037912000000	1.255155000000	2.817902000000
C	-5.110805000000	-0.227227000000	3.211860000000
H	-4.975323000000	1.872875000000	3.724933000000
C	-2.842491000000	0.451158000000	2.994784000000
H	-3.240097000000	2.393331000000	2.116520000000
C	-3.679112000000	-0.488850000000	3.598497000000
H	-5.829203000000	-0.414211000000	4.013056000000
H	-5.413941000000	-0.809929000000	2.333439000000
C	-3.158560000000	-1.426400000000	4.482672000000
C	-1.498386000000	0.536608000000	3.337240000000
C	-1.796788000000	-1.389803000000	4.775433000000
H	-3.805121000000	-2.161131000000	4.953168000000
C	-0.975904000000	-0.404023000000	4.222456000000
H	-1.374674000000	-2.112767000000	5.467485000000
H	0.077080000000	-0.368504000000	4.487651000000
H	-0.873255000000	1.301060000000	2.890342000000

C	-3.559433000000	0.205830000000	-5.107251000000	C	2.465729000000	-0.698632000000	1.407073000000
C	-3.405885000000	2.335330000000	-3.765469000000	C	1.649919000000	-1.625495000000	1.940978000000
C	-3.741879000000	-1.166436000000	-5.215070000000	N	0.746584000000	0.856675000000	0.854271000000
H	-3.360884000000	0.801566000000	-5.993300000000	C	3.073366000000	1.317940000000	0.089503000000
C	-4.016537000000	-1.925990000000	-4.083962000000	C	3.094024000000	1.459825000000	-1.300731000000
H	-3.680979000000	-1.647095000000	-6.186250000000	C	4.137001000000	2.171923000000	-1.888805000000
H	-4.184308000000	-2.992310000000	-4.186207000000	C	5.128387000000	2.755779000000	-1.105021000000
C	-4.435808000000	-2.144549000000	-1.589081000000	C	5.104458000000	2.605569000000	0.280390000000
C	-5.147094000000	-3.467248000000	-1.862598000000	C	4.091423000000	1.864219000000	0.876459000000
H	-3.511416000000	-2.338386000000	-1.030921000000	S	0.149847000000	2.301693000000	0.212382000000
H	-5.059815000000	-1.527467000000	-0.930612000000	C	1.226480000000	3.582963000000	0.817542000000
C	-2.097849000000	2.714501000000	-3.066353000000	C	1.338171000000	3.795513000000	2.187250000000
H	-3.412870000000	2.743438000000	-4.781285000000	C	2.233485000000	4.755562000000	2.644305000000
H	-4.251701000000	2.786620000000	-3.236368000000	C	3.002695000000	5.504524000000	1.748613000000
H	-1.927792000000	3.793202000000	-3.126047000000	C	2.843520000000	5.287499000000	0.376902000000
H	-2.108653000000	2.446553000000	-2.007644000000	C	1.959967000000	4.327264000000	-0.098616000000
H	-1.238472000000	2.208743000000	-3.516491000000	C	4.007899000000	6.509596000000	2.246514000000
H	-5.453128000000	-3.927193000000	-0.920122000000	O	-1.141760000000	2.466969000000	0.879249000000
H	-6.039837000000	-3.320642000000	-2.477467000000	O	0.220096000000	2.298417000000	-1.239913000000
H	-4.493021000000	-4.178213000000	-2.373903000000	C	2.071226000000	-2.879327000000	2.560744000000
C	4.264420000000	-1.602463000000	-2.609722000000	C	1.148540000000	-3.561447000000	3.363632000000
C	5.176135000000	-0.948722000000	-3.437847000000	C	1.493760000000	-4.756928000000	3.980442000000
C	4.499220000000	-1.630460000000	-1.234291000000	C	2.762996000000	-5.296478000000	3.784391000000
C	6.303402000000	-0.330997000000	-2.903357000000	C	3.683153000000	-4.636289000000	2.970578000000
H	4.991041000000	-0.909734000000	-4.508414000000	C	3.341818000000	-3.436225000000	2.361836000000
C	6.526500000000	-0.354314000000	-1.529419000000	H	3.541942000000	-0.839673000000	1.398738000000
H	6.999196000000	0.184058000000	-3.558950000000	H	0.576812000000	-1.452669000000	1.929979000000
C	5.621231000000	-1.007067000000	-0.696743000000	H	2.310219000000	1.025737000000	-1.917415000000
H	7.394596000000	0.142414000000	-1.107517000000	H	4.169556000000	2.262850000000	-2.969584000000
H	5.789306000000	-1.020526000000	0.377593000000	H	5.930635000000	3.316341000000	-1.575550000000
H	3.780999000000	-2.128425000000	-0.587063000000	H	5.877905000000	3.056424000000	0.894596000000
H	2.780236000000	-1.779278000000	-4.145374000000	H	4.065832000000	1.736301000000	1.955767000000
H	3.238100000000	-3.325985000000	-3.410184000000	H	0.738155000000	3.215829000000	2.882857000000

H	2.337236000000	4.928850000000	3.711670000000
H	3.432791000000	5.867681000000	-0.327965000000
H	1.848706000000	4.127262000000	-1.159009000000
H	3.812931000000	6.789190000000	3.283935000000
H	5.020168000000	6.094678000000	2.195453000000
H	3.994769000000	7.416400000000	1.636827000000
H	0.151433000000	-3.144150000000	3.484458000000
H	0.770920000000	-5.274984000000	4.602456000000
H	3.033021000000	-6.236010000000	4.256164000000
H	4.665169000000	-5.066412000000	2.802456000000
H	4.053630000000	-2.945315000000	1.703894000000

----

^{sr}TSa4'

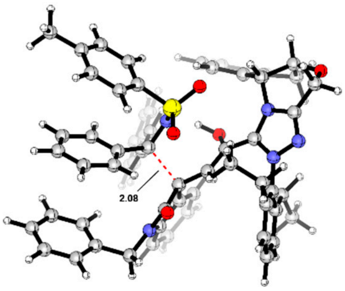

Frequency -290.5641

Zero-point correction= 1.041211 (Hartree/Particle)

Thermal correction to Energy= 1.101537

Thermal correction to Enthalpy= 1.102482

Thermal correction to Gibbs Free Energy= 0.943532

Sum of electronic and zero-point Energies= -3403.352873

Sum of electronic and thermal Energies= -3403.292546

Sum of electronic and thermal Enthalpies= -3403.291602

Sum of electronic and thermal Free Energies= -3403.450552

E(RM062X) = -3405.14080532

C	-0.517845000000	1.357582000000	0.353129000000
C	-1.330739000000	-0.542864000000	0.102886000000
C	0.788265000000	1.291707000000	-0.292862000000
C	1.812317000000	0.486601000000	0.057366000000
C	-0.647083000000	1.683188000000	1.802175000000
C	-1.779160000000	2.510777000000	1.951912000000
C	-2.249436000000	2.903634000000	3.195215000000
C	-1.516541000000	2.508449000000	4.320954000000
C	-0.366926000000	1.736327000000	4.189490000000
C	0.066710000000	1.302978000000	2.930178000000
H	0.183899000000	1.435518000000	5.074556000000
H	-1.854323000000	2.813323000000	5.306417000000
H	-3.144628000000	3.509966000000	3.292643000000
N	-2.263999000000	2.853789000000	0.686742000000
H	0.927179000000	0.649440000000	2.832191000000
C	-1.397377000000	2.388532000000	-0.290662000000
C	-3.361324000000	3.737720000000	0.373766000000
O	-1.367305000000	2.814100000000	-1.432977000000
H	0.879374000000	1.900137000000	-1.186648000000
C	3.068893000000	0.567333000000	-0.675449000000
O	1.771254000000	-0.463645000000	1.022578000000
H	1.016400000000	-1.045749000000	0.697828000000
N	3.580396000000	1.702865000000	-1.184942000000
N	3.957359000000	-0.404967000000	-1.004087000000
C	3.110006000000	3.053989000000	-0.993725000000
N	4.731544000000	1.486870000000	-1.875668000000
C	4.925054000000	0.210434000000	-1.764023000000
C	6.061077000000	-0.598310000000	-2.284134000000
C	4.097885000000	-1.895757000000	-0.859036000000
C	2.666287000000	3.762016000000	-2.114012000000
C	3.134188000000	3.577905000000	0.307158000000
O	6.570847000000	-1.357710000000	-1.213694000000
H	5.726079000000	-1.236418000000	-3.114877000000
H	6.850391000000	0.063830000000	-2.640393000000
C	5.609691000000	-2.161340000000	-0.556079000000

C	5.704376000000	-1.992674000000	0.967594000000
H	5.781671000000	-3.213108000000	-0.819041000000
C	3.485503000000	-2.579955000000	0.341773000000
H	3.737628000000	-2.309154000000	-1.802646000000
C	4.399565000000	-2.608046000000	1.397736000000
H	6.585744000000	-2.482852000000	1.386687000000
H	5.764269000000	-0.922897000000	1.205959000000
C	4.078472000000	-3.247524000000	2.588448000000
C	2.286383000000	-3.278815000000	0.413128000000
C	2.850256000000	-3.897666000000	2.689440000000
H	4.783222000000	-3.266849000000	3.414150000000
C	1.978111000000	-3.937397000000	1.600740000000
H	2.585504000000	-4.407570000000	3.610784000000
H	1.049776000000	-4.497255000000	1.672534000000
H	1.616926000000	-3.289901000000	-0.438223000000
C	2.181413000000	5.051588000000	-1.889640000000
C	2.667310000000	3.177019000000	-3.508408000000
C	2.152858000000	5.593795000000	-0.612446000000
H	1.815411000000	5.625235000000	-2.735540000000
C	2.632858000000	4.867803000000	0.472954000000
H	1.759608000000	6.593448000000	-0.458901000000
H	2.619876000000	5.312576000000	1.461282000000
C	3.665121000000	2.765883000000	1.470369000000
C	3.902081000000	3.556003000000	2.753416000000
H	2.957520000000	1.956098000000	1.689637000000
H	4.599854000000	2.281332000000	1.158812000000
C	1.636012000000	2.060556000000	-3.710350000000
H	2.458939000000	3.994526000000	-4.205267000000
H	3.669681000000	2.808305000000	-3.748774000000
H	1.583032000000	1.782117000000	-4.765959000000
H	1.881133000000	1.151669000000	-3.154451000000
H	0.637260000000	2.367882000000	-3.386908000000
H	4.363097000000	2.911622000000	3.504865000000
H	4.563333000000	4.410558000000	2.584104000000
H	2.960242000000	3.922861000000	3.170067000000
C	-4.647622000000	2.986817000000	0.084374000000
C	-5.558624000000	3.492299000000	-0.841283000000
C	-4.932945000000	1.782549000000	0.730098000000
C	-6.744020000000	2.812542000000	-1.111164000000
H	-5.331986000000	4.417844000000	-1.364672000000
C	-7.025282000000	1.616617000000	-0.458410000000
H	-7.441962000000	3.213201000000	-1.839834000000
C	-6.115032000000	1.101978000000	0.462312000000
H	-7.943316000000	1.079507000000	-0.675522000000
H	-6.313273000000	0.156327000000	0.956659000000
H	-4.214053000000	1.366030000000	1.430917000000
H	-3.064657000000	4.315872000000	-0.506386000000
H	-3.494130000000	4.438015000000	1.206720000000
C	-1.607574000000	-0.828960000000	1.540509000000
C	-1.008432000000	-1.772273000000	2.274250000000
N	-0.284129000000	-1.306509000000	-0.380326000000
C	-2.649486000000	-0.532758000000	-0.681385000000
C	-2.978784000000	0.291428000000	-1.760491000000
C	-4.191827000000	0.129942000000	-2.428502000000
C	-5.073905000000	-0.882606000000	-2.072274000000
C	-4.737326000000	-1.739028000000	-1.028042000000
C	-3.548481000000	-1.553796000000	-0.332796000000
S	0.030399000000	-1.352174000000	-1.962516000000
C	-1.105729000000	-2.561941000000	-2.623886000000
C	-1.206892000000	-3.810644000000	-2.016938000000
C	-2.140327000000	-4.721672000000	-2.492666000000
C	-2.985725000000	-4.393257000000	-3.557799000000
C	-2.861689000000	-3.135526000000	-4.149209000000
C	-1.923819000000	-2.215706000000	-3.690472000000
C	-4.041095000000	-5.360736000000	-4.027015000000
O	1.397593000000	-1.893629000000	-2.098560000000

O	-0.239865000000	-0.094927000000	-2.668215000000
C	-1.268738000000	-2.036237000000	3.696873000000
C	-0.722371000000	-3.184601000000	4.282784000000
C	-0.937204000000	-3.481108000000	5.624390000000
C	-1.702360000000	-2.625316000000	6.411007000000
C	-2.244344000000	-1.472376000000	5.843959000000
C	-2.030046000000	-1.177912000000	4.503950000000
H	-2.386911000000	-0.209828000000	1.977775000000
H	-0.270501000000	-2.412341000000	1.794689000000
H	-2.283570000000	1.038408000000	-2.109364000000
H	-4.435458000000	0.803440000000	-3.244534000000
H	-6.013413000000	-1.005816000000	-2.602839000000
H	-5.401476000000	-2.550268000000	-0.744795000000
H	-3.295768000000	-2.227822000000	0.479572000000
H	-0.581751000000	-4.050896000000	-1.161474000000
H	-2.229275000000	-5.697743000000	-2.023239000000
H	-3.516159000000	-2.866257000000	-4.973961000000
H	-1.833105000000	-1.226662000000	-4.126636000000
H	-3.750328000000	-6.395031000000	-3.828219000000
H	-4.988324000000	-5.177089000000	-3.508593000000
H	-4.226913000000	-5.255736000000	-5.098646000000
H	-0.118540000000	-3.847825000000	3.669087000000
H	-0.506121000000	-4.379364000000	6.055597000000
H	-1.871655000000	-2.849792000000	7.459291000000
H	-2.833044000000	-0.793196000000	6.453074000000
H	-2.434674000000	-0.259883000000	4.088568000000

----

^{sr}INTa7'

Zero-point correction= 1.041210 (Hartree/Particle)

Thermal correction to Energy= 1.101378

Thermal correction to Enthalpy= 1.102322

Thermal correction to Gibbs Free Energy= 0.944810

Sum of electronic and zero-point Energies= -3403.356978

Sum of electronic and thermal Energies= -3403.296810

Sum of electronic and thermal Enthalpies= -3403.295866

Sum of electronic and thermal Free Energies= -3403.453378

E(RM062X) = -3405.14389143

C	1.790657000000	-0.442554000000	0.012149000000
H	0.860982000000	1.089847000000	-0.448903000000
C	0.756228000000	-1.230967000000	0.357334000000
C	-0.588671000000	-1.193830000000	-0.306424000000
C	-0.550866000000	-1.679493000000	-1.752555000000
C	-1.596429000000	-2.596416000000	-1.942164000000
C	-1.887733000000	-3.134981000000	-3.186476000000
C	-1.057716000000	-2.779387000000	-4.255440000000
C	0.012824000000	-1.909765000000	-4.074696000000
C	0.263057000000	-1.338159000000	-2.820065000000
H	0.640311000000	-1.641968000000	-4.918116000000
H	-1.255917000000	-3.194784000000	-5.238535000000
H	-2.714816000000	-3.822773000000	-3.327804000000
N	-2.195795000000	-2.872275000000	-0.706863000000
H	1.052385000000	-0.605360000000	-2.683736000000
C	-1.443148000000	-2.314598000000	0.309440000000
C	-3.275241000000	-3.793135000000	-0.423297000000
O	-1.432893000000	-2.729353000000	1.448635000000
H	0.842058000000	-1.926569000000	1.181734000000
C	3.056085000000	-0.565512000000	0.737521000000
O	1.727259000000	0.567497000000	-0.857529000000
N	3.561930000000	-1.712850000000	1.218587000000
N	3.940707000000	0.398105000000	1.092873000000
C	3.037552000000	-3.045976000000	1.046379000000
N	4.711537000000	-1.516959000000	1.917487000000
C	4.905612000000	-0.237254000000	1.840590000000

C	6.032666000000	0.558677000000	2.398861000000
C	4.079907000000	1.895681000000	0.982085000000
C	2.554174000000	-3.709948000000	2.177051000000
C	3.011108000000	-3.578870000000	-0.250478000000
O	6.552696000000	1.356867000000	1.363431000000
H	5.683093000000	1.165819000000	3.246816000000
H	6.820399000000	-0.111550000000	2.743531000000
C	5.596132000000	2.169627000000	0.709293000000
C	5.715562000000	2.032347000000	-0.815755000000
H	5.760993000000	3.216031000000	0.996324000000
C	3.487457000000	2.607090000000	-0.213868000000
H	3.702379000000	2.285750000000	1.929263000000
C	4.416434000000	2.652836000000	-1.254998000000
H	6.602822000000	2.533365000000	-1.208991000000
H	5.783990000000	0.967716000000	-1.074195000000
C	4.109962000000	3.306303000000	-2.442589000000
C	2.287094000000	3.303344000000	-0.292876000000
C	2.879467000000	3.949415000000	-2.554018000000
H	4.826633000000	3.338629000000	-3.257651000000
C	1.991442000000	3.971806000000	-1.477068000000
H	2.623131000000	4.465364000000	-3.474286000000
H	1.058486000000	4.522582000000	-1.557589000000
H	1.602754000000	3.297099000000	0.547078000000
C	1.986423000000	-4.968976000000	1.969714000000
C	2.582630000000	-3.101367000000	3.561313000000
C	1.912428000000	-5.519605000000	0.698068000000
H	1.587731000000	-5.508340000000	2.823434000000
C	2.426009000000	-4.834991000000	-0.399485000000
H	1.454327000000	-6.493258000000	0.557311000000
H	2.367742000000	-5.283571000000	-1.384239000000
C	3.573202000000	-2.806283000000	-1.425939000000
C	3.617824000000	-3.575084000000	-2.742169000000
H	2.965715000000	-1.905805000000	-1.580536000000
H	4.580469000000	-2.457291000000	-1.163356000000
C	1.592756000000	-1.943550000000	3.744737000000
H	2.343502000000	-3.897595000000	4.272868000000
H	3.598535000000	-2.767674000000	3.794553000000
H	1.558747000000	-1.640687000000	4.794383000000
H	1.861792000000	-1.055679000000	3.165144000000
H	0.580813000000	-2.220631000000	3.436566000000
H	4.093903000000	-2.963049000000	-3.511181000000
H	4.184469000000	-4.506110000000	-2.650456000000
H	2.608276000000	-3.812589000000	-3.089771000000
C	-4.555812000000	-3.047476000000	-0.100922000000
C	-5.373137000000	-3.457909000000	0.948157000000
C	-4.911419000000	-1.917367000000	-0.841854000000
C	-6.539602000000	-2.756161000000	1.249183000000
H	-5.088986000000	-4.321925000000	1.543285000000
C	-6.887311000000	-1.632755000000	0.507535000000
H	-7.165880000000	-3.079638000000	2.074765000000
C	-6.068765000000	-1.212237000000	-0.539568000000
H	-7.784978000000	-1.073963000000	0.753016000000
H	-6.320128000000	-0.320735000000	-1.104881000000
H	-4.262004000000	-1.576876000000	-1.645127000000
H	-2.974064000000	-4.409806000000	0.428841000000
H	-3.404243000000	-4.451745000000	-1.288277000000
C	-1.547672000000	0.747625000000	-1.663208000000
C	-1.310103000000	0.364055000000	-0.215478000000
C	-0.930308000000	1.718209000000	-2.338142000000
N	-0.334641000000	1.292003000000	0.326735000000
C	-2.682574000000	0.494647000000	0.506048000000
C	-3.119560000000	-0.265310000000	1.596325000000
C	-4.331424000000	0.013934000000	2.225106000000
C	-5.115557000000	1.085360000000	1.819402000000
C	-4.671583000000	1.883338000000	0.769440000000
C	-3.481139000000	1.581888000000	0.117607000000

C	-1.248463000000	2.548043000000	2.527425000000
C	-1.366216000000	3.768790000000	1.868257000000
C	-2.299571000000	4.693549000000	2.315138000000
C	-3.130010000000	4.406641000000	3.404269000000
C	-2.991092000000	3.176730000000	4.047241000000
C	-2.053759000000	2.242542000000	3.615267000000
C	-4.187674000000	5.387414000000	3.839859000000
S	-0.095855000000	1.326007000000	1.915263000000
O	1.272246000000	1.848205000000	2.127506000000
O	-0.419919000000	0.075800000000	2.615233000000
C	-1.130703000000	2.003414000000	-3.768604000000
C	-0.688297000000	3.225331000000	-4.289891000000
C	-0.850994000000	3.535021000000	-5.635750000000
C	-1.455071000000	2.619144000000	-6.492485000000
C	-1.886404000000	1.392147000000	-5.991064000000
C	-1.722578000000	1.083660000000	-4.646365000000
H	-2.304301000000	0.142030000000	-2.159072000000
H	-0.214426000000	2.349704000000	-1.817033000000
H	-2.502373000000	-1.045987000000	2.008564000000
H	-4.647820000000	-0.611920000000	3.054108000000
H	-6.052857000000	1.305407000000	2.322184000000
H	-5.251143000000	2.744788000000	0.450569000000
H	-3.146712000000	2.223983000000	-0.690132000000
H	-0.756420000000	3.968049000000	0.991272000000
H	-2.402403000000	5.647133000000	1.803832000000
H	-3.635733000000	2.938737000000	4.889319000000
H	-1.955396000000	1.270938000000	4.087620000000
H	-3.885286000000	6.417155000000	3.633863000000
H	-5.125326000000	5.204623000000	3.303705000000
H	-4.396422000000	5.297892000000	4.908630000000
H	-0.214330000000	3.938761000000	-3.620554000000
H	-0.504024000000	4.490838000000	-6.016342000000
H	-1.580852000000	2.854041000000	-7.544627000000
H	-2.343255000000	0.665198000000	-6.655805000000
H	-2.032489000000	0.109844000000	-4.278185000000

----

*no*INTa6

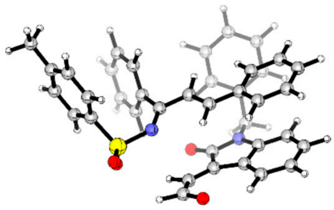

Zero-point correction= 0.622155 (Hartree/Particle)

Thermal correction to Energy= 0.662226

Thermal correction to Enthalpy= 0.663171

Thermal correction to Gibbs Free Energy= 0.546958

Sum of electronic and zero-point Energies= -2312.550768

Sum of electronic and thermal Energies= -2312.510697

Sum of electronic and thermal Enthalpies= -2312.509753

Sum of electronic and thermal Free Energies= -2312.625966

E(RM062X) = -2313.66619096

C	5.488451000000	-1.254862000000	0.058512000000
C	4.757169000000	-0.279122000000	-0.622884000000
C	3.503073000000	-0.630914000000	-1.090083000000
C	2.942797000000	-1.907225000000	-0.857926000000
C	3.693542000000	-2.866938000000	-0.184547000000
C	4.973361000000	-2.532323000000	0.261923000000
N	2.614840000000	0.158515000000	-1.831474000000
C	1.457990000000	-0.535641000000	-2.099570000000
C	1.606043000000	-1.895971000000	-1.458674000000
O	0.503248000000	-0.097347000000	-2.716269000000
C	0.535831000000	-2.710122000000	-1.495237000000
C	0.363409000000	-3.985426000000	-0.765297000000

O	1.240523000000	-4.542545000000	-0.137753000000
C	2.449557000000	2.595641000000	-1.436631000000
C	1.086125000000	2.805232000000	-1.208758000000
C	0.664616000000	3.800471000000	-0.332330000000
C	1.604238000000	4.601818000000	0.317562000000
C	2.962741000000	4.409776000000	0.079103000000
C	3.382616000000	3.407398000000	-0.794472000000
C	2.873758000000	1.482610000000	-2.368338000000
H	6.473562000000	-1.003910000000	0.439449000000
H	5.153208000000	0.720787000000	-0.764036000000
H	3.275583000000	-3.850038000000	-0.015833000000
H	5.565089000000	-3.273648000000	0.788188000000
H	-0.331353000000	-2.351383000000	-2.048532000000
H	-0.658024000000	-4.403649000000	-0.799342000000
H	0.360977000000	2.171739000000	-1.716114000000
H	-0.398840000000	3.944322000000	-0.159656000000
H	1.277214000000	5.378988000000	1.000927000000
H	3.696810000000	5.038454000000	0.573807000000
H	4.444252000000	3.255327000000	-0.975354000000
H	2.308087000000	1.546468000000	-3.302743000000
H	3.939681000000	1.554722000000	-2.606085000000
C	0.131879000000	0.292958000000	0.874991000000
C	-1.118819000000	-0.200115000000	0.291287000000
C	1.072718000000	-0.507567000000	1.410616000000
N	-1.355482000000	-1.469224000000	0.324355000000
C	-1.996801000000	0.867832000000	-0.281740000000
C	-2.229490000000	0.943164000000	-1.657165000000
C	-3.010642000000	1.981068000000	-2.160734000000
C	-3.575948000000	2.920803000000	-1.302717000000
C	-3.345104000000	2.839993000000	0.070062000000
C	-2.538945000000	1.827428000000	0.578827000000
C	-4.109275000000	-1.188988000000	0.379825000000
C	-4.287936000000	-1.120306000000	1.757079000000
C	-5.288816000000	-0.296501000000	2.259843000000
C	-6.110055000000	0.441306000000	1.402772000000
C	-5.924219000000	0.324345000000	0.021495000000
C	-4.925000000000	-0.486823000000	-0.499611000000
C	-7.169174000000	1.363852000000	1.946812000000
S	-2.773031000000	-2.170168000000	-0.263089000000
O	-2.814747000000	-3.472536000000	0.381660000000
O	-2.787194000000	-2.085790000000	-1.721720000000
C	2.343076000000	-0.045843000000	1.971357000000
C	3.157746000000	-0.961631000000	2.647036000000
C	4.370612000000	-0.561373000000	3.197647000000
C	4.791174000000	0.758328000000	3.066177000000
C	3.993068000000	1.677282000000	2.382461000000
C	2.779217000000	1.282900000000	1.841290000000
H	0.268334000000	1.367979000000	0.838678000000
H	0.882519000000	-1.581046000000	1.433466000000
H	-1.787482000000	0.203696000000	-2.315605000000
H	-3.179680000000	2.049908000000	-3.230418000000
H	-4.193166000000	3.719285000000	-1.702443000000
H	-3.786369000000	3.568251000000	0.743435000000
H	-2.346964000000	1.760907000000	1.646505000000
H	-3.652685000000	-1.702273000000	2.418093000000
H	-5.437870000000	-0.226431000000	3.333518000000
H	-6.563036000000	0.888601000000	-0.652721000000
H	-4.752777000000	-0.568728000000	-1.567437000000
H	-7.319299000000	1.212181000000	3.017548000000
H	-6.884309000000	2.409592000000	1.790570000000
H	-8.125559000000	1.206899000000	1.440995000000
H	2.835089000000	-1.996048000000	2.728410000000
H	4.991676000000	-1.283882000000	3.717428000000
H	5.740320000000	1.072251000000	3.489580000000
H	4.318813000000	2.706553000000	2.265298000000
H	2.177049000000	2.008447000000	1.302579000000

***no*TSa4**

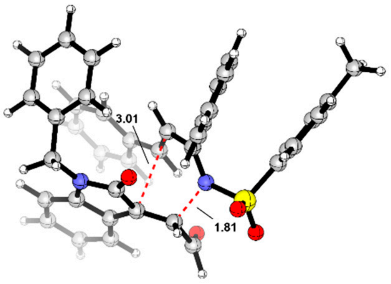

Frequency -407.0606

Zero-point correction= 0.623099 (Hartree/Particle)

Thermal correction to Energy= 0.661162

Thermal correction to Enthalpy= 0.662106

Thermal correction to Gibbs Free Energy= 0.553604

Sum of electronic and zero-point Energies= -2312.514836

Sum of electronic and thermal Energies= -2312.476773

Sum of electronic and thermal Enthalpies= -2312.475829

Sum of electronic and thermal Free Energies= -2312.584331

E(RM062X) = -2313.63035035

C	1.227371000000	-1.526929000000	-1.225361000000
C	0.810038000000	-0.582888000000	1.199570000000
C	2.563839000000	-1.937588000000	-0.826713000000
C	3.413890000000	-0.831073000000	-1.096181000000
C	4.770338000000	-0.854113000000	-0.813311000000
C	5.310564000000	-2.028787000000	-0.288008000000
C	4.503139000000	-3.140226000000	-0.052637000000
H	6.371138000000	-2.072072000000	-0.062741000000
H	5.394296000000	0.015504000000	-0.994366000000
N	2.664316000000	0.196031000000	-1.668823000000
C	3.132291000000	-3.100659000000	-0.306091000000
H	2.517067000000	-3.964655000000	-0.083215000000
H	4.941060000000	-4.047209000000	0.351281000000
C	1.350147000000	-0.206596000000	-1.826283000000
C	3.132324000000	1.482349000000	-2.149574000000
O	0.451467000000	0.480065000000	-2.315515000000
C	-0.050297000000	-2.140426000000	-1.293534000000
C	-0.131166000000	-3.531488000000	-0.709063000000
H	-0.541731000000	-2.007347000000	-2.259759000000
O	0.172145000000	-3.754408000000	0.438907000000
H	-0.430578000000	-4.337606000000	-1.399453000000
C	2.933734000000	2.586865000000	-1.134533000000
C	1.645187000000	3.078342000000	-0.895163000000
C	4.004431000000	3.093852000000	-0.401835000000
C	1.436105000000	4.048304000000	0.081059000000
H	0.811711000000	2.671822000000	-1.464051000000
C	2.511568000000	4.544779000000	0.818726000000
H	0.430684000000	4.416292000000	0.264578000000
C	3.797019000000	4.072640000000	0.569674000000
H	2.348580000000	5.302173000000	1.578797000000
H	4.639413000000	4.462314000000	1.133540000000
H	5.006951000000	2.713182000000	-0.583380000000
H	2.558356000000	1.703663000000	-3.054462000000
H	4.186765000000	1.387602000000	-2.425088000000
C	-0.017526000000	0.403426000000	0.718910000000
C	-1.136199000000	0.086717000000	-0.073656000000
H	0.235826000000	1.454272000000	0.814400000000
N	-1.316308000000	-1.210582000000	-0.399496000000
C	-2.041658000000	1.200821000000	-0.463604000000
C	2.096489000000	-0.369349000000	1.835468000000
H	0.436109000000	-1.604811000000	1.204769000000
S	-2.803762000000	-1.786421000000	-1.011815000000
C	-2.363416000000	1.482587000000	-1.795690000000
C	-2.561464000000	1.997782000000	0.562142000000
C	-3.222188000000	2.538229000000	-2.083639000000
H	-1.918978000000	0.886289000000	-2.582711000000

C	-3.761509000000	3.314001000000	-1.058666000000
H	-3.463845000000	2.761272000000	-3.117873000000
C	-3.425135000000	3.046824000000	0.266215000000
H	-4.434079000000	4.132944000000	-1.294130000000
H	-3.837961000000	3.650105000000	1.068667000000
H	-2.308919000000	1.766379000000	1.593441000000
C	-3.958379000000	-1.101681000000	0.140839000000
C	-3.835957000000	-1.416418000000	1.489886000000
C	-4.942474000000	-0.241154000000	-0.332013000000
C	-4.717054000000	-0.826903000000	2.388587000000
H	-3.059356000000	-2.098816000000	1.822623000000
C	-5.710373000000	0.054355000000	1.949861000000
H	-4.633831000000	-1.053601000000	3.447371000000
C	-5.816936000000	0.329661000000	0.582967000000
C	-6.643253000000	0.717838000000	2.928602000000
H	-6.584314000000	1.014585000000	0.233099000000
H	-4.988612000000	-0.009131000000	-1.390960000000
H	-6.551485000000	0.282253000000	3.925367000000
H	-6.421850000000	1.787213000000	3.005558000000
H	-7.683331000000	0.621745000000	2.605833000000
O	-2.695278000000	-3.227916000000	-0.820883000000
O	-3.104700000000	-1.284745000000	-2.345515000000
C	2.751998000000	-1.482625000000	2.384066000000
C	2.735913000000	0.882025000000	1.862395000000
C	4.016734000000	-1.354941000000	2.942368000000
H	2.261298000000	-2.451193000000	2.341864000000
C	4.640145000000	-0.110402000000	2.963011000000
H	4.520670000000	-2.226670000000	3.346540000000
C	3.995418000000	1.006221000000	2.428850000000
H	5.631539000000	-0.007712000000	3.393558000000
H	4.481757000000	1.976287000000	2.438580000000
H	2.258525000000	1.755312000000	1.427786000000

----

^{no}INTa7

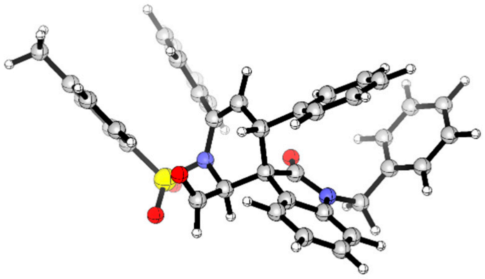

Zero-point correction=	0.626880 (Hartree/Particle)
Thermal correction to Energy=	0.664813
Thermal correction to Enthalpy=	0.665757
Thermal correction to Gibbs Free Energy=	0.555498
Sum of electronic and zero-point Energies=	-2312.571128
Sum of electronic and thermal Energies=	-2312.533195
Sum of electronic and thermal Enthalpies=	-2312.532251
Sum of electronic and thermal Free Energies=	-2312.642510

E(RM062X) = -2313.69058600

C	0.905213000000	-1.140839000000	-0.163959000000
C	0.623167000000	-0.640778000000	1.288297000000
C	1.983868000000	-2.203587000000	-0.168346000000
C	3.093244000000	-1.719185000000	-0.873972000000
C	4.268168000000	-2.448567000000	-0.977143000000
C	4.316836000000	-3.693009000000	-0.344844000000
C	3.231605000000	-4.180268000000	0.375857000000
H	5.225430000000	-4.282657000000	-0.410804000000
H	5.126504000000	-2.068927000000	-1.520651000000
N	2.800251000000	-0.457528000000	-1.398577000000
C	2.057177000000	-3.427136000000	0.476245000000
H	1.220352000000	-3.796912000000	1.063249000000
H	3.297421000000	-5.143874000000	0.868581000000
C	1.539470000000	-0.038508000000	-1.042045000000
C	3.664659000000	0.372155000000	-2.222454000000

O	1.038502000000	1.005766000000	-1.406360000000
C	-0.404844000000	-1.598624000000	-0.844752000000
C	-0.857310000000	-2.929701000000	-0.241286000000
H	-0.194329000000	-1.770307000000	-1.905963000000
O	-1.399431000000	-3.004865000000	0.834729000000
H	-0.605259000000	-3.830680000000	-0.829639000000
C	4.373226000000	1.455350000000	-1.438760000000
C	3.796049000000	2.722301000000	-1.328971000000
C	5.597530000000	1.205538000000	-0.818877000000
C	4.451258000000	3.733076000000	-0.630806000000
H	2.828785000000	2.901539000000	-1.789692000000
C	5.682362000000	3.481765000000	-0.028300000000
H	4.001493000000	4.718452000000	-0.559063000000
C	6.249825000000	2.212736000000	-0.113482000000
H	6.196274000000	4.272221000000	0.509832000000
H	7.204689000000	2.009929000000	0.361051000000
H	6.046717000000	0.218116000000	-0.892384000000
H	3.028726000000	0.824261000000	-2.988714000000
H	4.383453000000	-0.285457000000	-2.719102000000
C	-0.479564000000	0.374624000000	1.243402000000
C	-1.410107000000	0.407290000000	0.282072000000
H	-0.453334000000	1.198985000000	1.946937000000
N	-1.446254000000	-0.575198000000	-0.734298000000
C	-2.348243000000	1.556303000000	0.184480000000
C	1.890217000000	-0.105415000000	1.929630000000
H	0.301448000000	-1.528177000000	1.850788000000
S	-2.811212000000	-0.908654000000	-1.688143000000
C	-2.376443000000	2.339771000000	-0.973064000000
C	-3.171647000000	1.881497000000	1.264727000000
C	-3.244792000000	3.423726000000	-1.051578000000
H	-1.723319000000	2.083743000000	-1.800735000000
C	-4.081621000000	3.733815000000	0.018781000000
H	-3.265434000000	4.029675000000	-1.951746000000

C	-4.038384000000	2.965544000000	1.180156000000
H	-4.763354000000	4.575765000000	-0.050776000000
H	-4.688637000000	3.202859000000	2.016626000000
H	-3.151934000000	1.254869000000	2.151545000000
C	-4.179295000000	-0.647327000000	-0.592526000000
C	-4.223457000000	-1.342430000000	0.611499000000
C	-5.155872000000	0.273396000000	-0.954105000000
C	-5.278933000000	-1.089170000000	1.478665000000
H	-3.430889000000	-2.036706000000	0.877732000000
C	-6.276128000000	-0.164535000000	1.152411000000
H	-5.325113000000	-1.610936000000	2.430286000000
C	-6.202678000000	0.506338000000	-0.071980000000
C	-7.419137000000	0.101051000000	2.096322000000
H	-6.965179000000	1.236985000000	-0.327426000000
H	-5.062664000000	0.815949000000	-1.888768000000
H	-7.165126000000	-0.186599000000	3.118725000000
H	-7.692518000000	1.159361000000	2.093307000000
H	-8.305570000000	-0.468855000000	1.799635000000
O	-2.667880000000	-2.332646000000	-1.986614000000
O	-2.921169000000	0.026435000000	-2.793701000000
C	2.639975000000	-0.908851000000	2.790899000000
C	2.353709000000	1.178561000000	1.629146000000
C	3.833030000000	-0.444932000000	3.337189000000
H	2.287534000000	-1.909695000000	3.027257000000
C	4.290460000000	0.832389000000	3.026395000000
H	4.405437000000	-1.083291000000	4.002879000000
C	3.546267000000	1.643609000000	2.174690000000
H	5.224069000000	1.195817000000	3.444637000000
H	3.898952000000	2.637013000000	1.919261000000
H	1.783451000000	1.809707000000	0.951451000000

The process of generating product **b** :

NHC^{II}

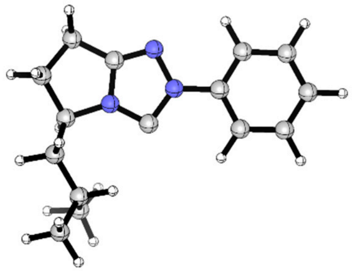

Zero-point correction=	0.320714 (Hartree/Particle)
Thermal correction to Energy=	0.336585
Thermal correction to Enthalpy=	0.337529
Thermal correction to Gibbs Free Energy=	0.276094
Sum of electronic and zero-point Energies=	-746.631927
Sum of electronic and thermal Energies=	-746.616057
Sum of electronic and thermal Enthalpies=	-746.615113
Sum of electronic and thermal Free Energies=	-746.676547

E(RM062X) = -747.125435073

C	0.041449000000	-0.279168000000	0.173524000000
N	-1.091571000000	0.464898000000	0.050376000000
N	-0.921751000000	1.837729000000	-0.031476000000
C	0.363503000000	1.952494000000	0.039594000000
N	0.961617000000	0.724188000000	0.149466000000
C	1.376029000000	3.050484000000	0.077470000000
C	2.682249000000	2.258655000000	-0.183549000000
C	2.418434000000	0.804907000000	0.284742000000
C	3.177003000000	-0.240163000000	-0.522516000000
C	2.899335000000	-1.707444000000	-0.164923000000
C	-2.411268000000	-0.064407000000	-0.002071000000
C	-3.503064000000	0.791498000000	-0.125712000000
C	-4.785578000000	0.252995000000	-0.171868000000
C	-4.981808000000	-1.122221000000	-0.096189000000
C	-3.879309000000	-1.965305000000	0.027096000000
C	-2.592258000000	-1.445319000000	0.074158000000
C	3.876792000000	-2.604910000000	-0.922790000000
C	2.974526000000	-1.957949000000	1.340271000000
H	1.190120000000	3.828109000000	-0.664292000000
H	1.377905000000	3.519547000000	1.066809000000
H	3.548885000000	2.694006000000	0.316536000000
H	2.886309000000	2.243289000000	-1.259116000000
H	2.665862000000	0.701930000000	1.348520000000
H	4.248146000000	-0.033000000000	-0.380083000000
H	2.962040000000	-0.078992000000	-1.587007000000
H	1.877309000000	-1.937664000000	-0.483722000000
H	-3.336817000000	1.859789000000	-0.183399000000
H	-5.636447000000	0.920037000000	-0.267440000000
H	-5.984640000000	-1.534529000000	-0.133461000000
H	-4.019880000000	-3.039848000000	0.087007000000
H	-1.720288000000	-2.080914000000	0.168710000000
H	3.653328000000	-3.661352000000	-0.749835000000
H	3.833060000000	-2.422766000000	-2.001299000000
H	4.906466000000	-2.421905000000	-0.592908000000
H	2.901079000000	-3.028017000000	1.554850000000
H	2.149544000000	-1.462306000000	1.860808000000
H	3.923740000000	-1.598010000000	1.757341000000

Pre-NHC^{II}

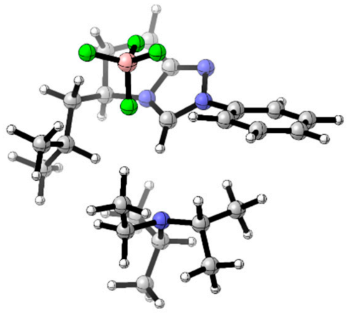

Zero-point correction= 0.618737 (Hartree/Particle)

Thermal correction to Energy= 0.652882

Thermal correction to Enthalpy= 0.653826

Thermal correction to Gibbs Free Energy= 0.554588

Sum of electronic and zero-point Energies= -1542.178361

Sum of electronic and thermal Energies= -1542.144215

Sum of electronic and thermal Enthalpies= -1542.143271

Sum of electronic and thermal Free Energies= -1542.242510

E(RM062X) = -1543.20341377

N	0.139058000000	1.707832000000	1.032705000000
N	1.098450000000	2.301247000000	1.802680000000
C	1.969505000000	1.354924000000	1.966402000000
N	1.586233000000	0.190260000000	1.356621000000
C	3.310495000000	1.217886000000	2.598062000000
C	3.864557000000	0.005101000000	1.819060000000
C	2.653765000000	-0.846007000000	1.355015000000
C	2.915647000000	-1.479955000000	-0.005029000000
C	1.856093000000	-2.459955000000	-0.514238000000
C	-0.939325000000	2.473699000000	0.490710000000
C	-1.648059000000	3.324695000000	1.331156000000
C	-2.714695000000	4.039724000000	0.801474000000
C	-3.057313000000	3.891411000000	-0.542914000000
C	-2.317288000000	3.048530000000	-1.368063000000
C	-1.233057000000	2.335600000000	-0.861163000000
C	2.025116000000	-2.647612000000	-2.021200000000
C	1.948603000000	-3.803095000000	0.211046000000
H	3.909136000000	2.119107000000	2.467742000000
H	3.205309000000	1.016207000000	3.668869000000
H	4.568558000000	-0.592184000000	2.399814000000
H	4.360008000000	0.371980000000	0.916477000000
H	2.372834000000	-1.596937000000	2.103444000000
H	3.870549000000	-2.016809000000	0.094347000000
H	3.079031000000	-0.679661000000	-0.729381000000
H	0.860819000000	-2.024230000000	-0.338040000000
H	-1.362057000000	3.411681000000	2.373671000000
H	-3.284153000000	4.707261000000	1.439142000000
H	-3.894037000000	4.449091000000	-0.950838000000
H	-2.562928000000	2.960708000000	-2.420900000000
H	-0.596654000000	1.723645000000	-1.494964000000
H	1.268970000000	-3.333472000000	-2.417722000000
H	1.949257000000	-1.686399000000	-2.534079000000
H	3.010844000000	-3.077074000000	-2.236737000000
H	1.146770000000	-4.480579000000	-0.103008000000
H	1.889621000000	-3.701888000000	1.300572000000
H	2.901387000000	-4.289701000000	-0.026756000000
N	-2.091211000000	-1.710736000000	0.051316000000
C	-1.773638000000	-2.540546000000	-1.117297000000
C	-1.338279000000	-1.739465000000	-2.338149000000
H	-2.616022000000	-3.193639000000	-1.397984000000
H	-0.954312000000	-3.210590000000	-0.824716000000
H	-0.973504000000	-2.423835000000	-3.108559000000
H	-2.169459000000	-1.178198000000	-2.775186000000
H	-0.530987000000	-1.040269000000	-2.101489000000
C	-3.101186000000	-0.668897000000	-0.225791000000
C	-3.315539000000	0.248273000000	0.980271000000
C	-4.454042000000	-1.172458000000	-0.743197000000
H	-2.657489000000	-0.047438000000	-1.011768000000
H	-2.380880000000	0.406039000000	1.527601000000

H	-3.678581000000	1.223565000000	0.642671000000
H	-4.054236000000	-0.155833000000	1.679631000000
H	-4.347015000000	-1.809533000000	-1.625864000000
H	-4.985674000000	-1.735865000000	0.030367000000
H	-5.082300000000	-0.320691000000	-1.021302000000
C	-2.329923000000	-2.514980000000	1.269869000000
C	-1.000074000000	-2.801311000000	1.967157000000
C	-3.103281000000	-3.826194000000	1.065584000000
H	-2.922134000000	-1.892197000000	1.945474000000
H	-0.473625000000	-1.868432000000	2.195642000000
H	-1.159414000000	-3.346282000000	2.902693000000
H	-0.355760000000	-3.413235000000	1.330476000000
H	-4.029416000000	-3.675833000000	0.505962000000
H	-2.500897000000	-4.566860000000	0.530642000000
H	-3.360506000000	-4.253217000000	2.039167000000
C	0.418424000000	0.434261000000	0.763616000000
H	-0.238765000000	-0.254286000000	0.237500000000
B	2.117157000000	1.271949000000	-1.889301000000
F	2.702863000000	1.587463000000	-0.640948000000
F	3.048851000000	0.649721000000	-2.697690000000
F	1.067881000000	0.315577000000	-1.623144000000
F	1.540983000000	2.386590000000	-2.456400000000

TS-NHC^{II}

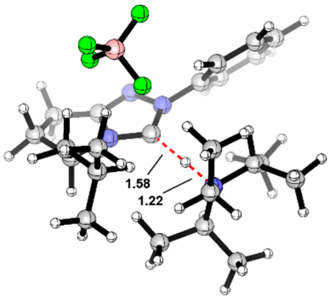

Frequency -416.5168

Zero-point correction= 0.615934 (Hartree/Particle)

Thermal correction to Energy= 0.649495

Thermal correction to Enthalpy= 0.650439

Thermal correction to Gibbs Free Energy= 0.549951

Sum of electronic and zero-point Energies= -1542.154233

Sum of electronic and thermal Energies= -1542.120672

Sum of electronic and thermal Enthalpies= -1542.119728

Sum of electronic and thermal Free Energies= -1542.220216

E(RM062X) = -1543.17685971

C	0.112281000000	-0.198089000000	-0.787967000000
H	-0.718743000000	1.018191000000	-0.220101000000
N	-0.470626000000	-1.375328000000	-1.112130000000
N	0.262521000000	-2.182078000000	-1.951379000000
C	-1.629882000000	-1.948727000000	-0.510564000000
C	1.341627000000	-1.495238000000	-2.115389000000
N	1.303442000000	-0.304413000000	-1.434691000000
C	2.617020000000	-1.686502000000	-2.853391000000
C	2.616230000000	0.391250000000	-1.452084000000
C	3.525575000000	-0.670133000000	-2.135297000000
H	2.979558000000	-2.710914000000	-2.768118000000
H	2.478333000000	-1.452101000000	-3.913657000000
H	4.251022000000	-0.199619000000	-2.800887000000
H	4.064450000000	-1.192169000000	-1.341538000000
C	3.197699000000	0.729666000000	-0.079632000000
H	2.518846000000	1.288343000000	-2.077325000000
C	2.504193000000	1.832115000000	0.714842000000
H	4.241813000000	1.026634000000	-0.262105000000
H	3.227949000000	-0.191502000000	0.505352000000
C	2.897316000000	1.736157000000	2.188653000000
C	2.834087000000	3.220822000000	0.163822000000
H	1.427932000000	1.635342000000	0.646326000000
C	-2.590175000000	-2.549617000000	-1.320022000000
C	-1.742582000000	-1.935646000000	0.876445000000

C	-3.722403000000	-3.096367000000	-0.728945000000
H	-2.436934000000	-2.578668000000	-2.393733000000
C	-3.883110000000	-3.042291000000	0.656090000000
H	-4.480501000000	-3.564468000000	-1.348264000000
C	-2.892267000000	-2.474204000000	1.452631000000
H	-4.768841000000	-3.470295000000	1.114361000000
H	-2.993280000000	-2.477274000000	2.532933000000
H	-0.916700000000	-1.553967000000	1.471950000000
H	2.393849000000	2.509804000000	2.779870000000
H	2.645664000000	0.750173000000	2.584043000000
H	3.977759000000	1.887403000000	2.299723000000
H	2.239124000000	4.002124000000	0.652539000000
H	2.674001000000	3.304278000000	-0.916504000000
H	3.888185000000	3.450316000000	0.356722000000
N	-1.464831000000	1.941976000000	0.037325000000
C	-0.908007000000	2.673020000000	1.213299000000
C	-2.778317000000	1.269582000000	0.370524000000
C	-1.467533000000	2.752684000000	-1.229807000000
C	-0.683842000000	1.765728000000	2.415623000000
H	-1.574117000000	3.506420000000	1.458938000000
H	0.047134000000	3.097948000000	0.902664000000
H	-0.166347000000	2.337468000000	3.189802000000
H	-1.620287000000	1.403934000000	2.847237000000
H	-0.060008000000	0.904206000000	2.161005000000
C	-3.445218000000	0.686439000000	-0.871774000000
C	-3.769349000000	2.120518000000	1.166681000000
H	-2.477900000000	0.430321000000	1.003068000000
H	-2.730466000000	0.179516000000	-1.522313000000
H	-4.174536000000	-0.062494000000	-0.551088000000
H	-3.970136000000	1.452916000000	-1.450392000000
H	-3.371070000000	2.452978000000	2.126472000000
H	-4.117285000000	2.995502000000	0.615033000000
H	-4.641063000000	1.494936000000	1.377086000000
C	-0.051800000000	3.199095000000	-1.581046000000
C	-2.421615000000	3.944459000000	-1.226967000000
H	-1.782446000000	2.047519000000	-2.003539000000
H	0.664716000000	2.386242000000	-1.445834000000
H	-0.029569000000	3.503471000000	-2.630521000000
H	0.273030000000	4.053943000000	-0.982177000000
H	-3.470578000000	3.646576000000	-1.223186000000
H	-2.238469000000	4.603935000000	-0.373805000000
H	-2.247766000000	4.528024000000	-2.134973000000
B	1.856888000000	-1.900095000000	1.651869000000
F	2.256070000000	-2.187946000000	0.329773000000
F	2.976852000000	-1.620766000000	2.423384000000
F	1.057886000000	-0.704588000000	1.605719000000
F	1.079872000000	-2.914916000000	2.168608000000

DIPEA

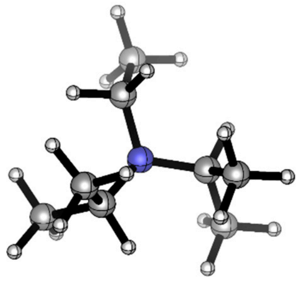

Zero-point correction=	0.264239 (Hartree/Particle)
Thermal correction to Energy=	0.276245
Thermal correction to Enthalpy=	0.277189
Thermal correction to Gibbs Free Energy=	0.227332
Sum of electronic and zero-point Energies=	-370.600359
Sum of electronic and thermal Energies=	-370.588353
Sum of electronic and thermal Enthalpies=	-370.587409
Sum of electronic and thermal Free Energies=	-370.637267

E(RM062X) = -370.950279496

N	0.004381000000	0.271185000000	0.185070000000
C	0.358835000000	1.428146000000	-0.626142000000
C	-0.133818000000	2.735592000000	-0.011680000000
H	-0.004737000000	1.339089000000	-1.666258000000
H	1.449950000000	1.477775000000	-0.691972000000
H	0.171400000000	3.589342000000	-0.623602000000
H	-1.223438000000	2.763811000000	0.074095000000
H	0.284566000000	2.850814000000	0.991263000000
C	-1.394616000000	-0.143847000000	0.051586000000
C	-1.898666000000	-0.776626000000	1.348911000000
C	-1.700918000000	-1.045244000000	-1.152708000000
H	-1.961550000000	0.784357000000	-0.088566000000
H	-1.690441000000	-0.115152000000	2.192814000000
H	-2.976226000000	-0.958095000000	1.294906000000
H	-1.415427000000	-1.740174000000	1.541748000000
H	-1.349621000000	-0.598898000000	-2.088129000000
H	-1.229503000000	-2.027493000000	-1.041020000000
H	-2.779709000000	-1.205448000000	-1.241781000000
C	1.002239000000	-0.804508000000	0.216153000000
C	2.097389000000	-0.456018000000	1.225083000000
C	1.622451000000	-1.178425000000	-1.139706000000
H	0.482512000000	-1.693268000000	0.593118000000
H	1.657663000000	-0.274363000000	2.208184000000
H	2.833111000000	-1.262322000000	1.304601000000
H	2.630374000000	0.451449000000	0.919989000000
H	0.861441000000	-1.390405000000	-1.893765000000
H	2.261786000000	-0.375462000000	-1.520087000000
H	2.249813000000	-2.068277000000	-1.030018000000

DIPEA • H$^+$

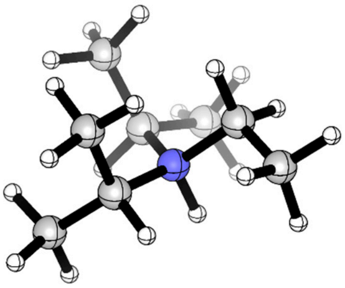

Zero-point correction=	0.280405 (Hartree/Particle)
Thermal correction to Energy=	0.292115
Thermal correction to Enthalpy=	0.293059
Thermal correction to Gibbs Free Energy=	0.244196
Sum of electronic and zero-point Energies=	-370.975859
Sum of electronic and thermal Energies=	-370.964149
Sum of electronic and thermal Enthalpies=	-370.963205
Sum of electronic and thermal Free Energies=	-371.012067

E(RM062X) = -371.338550750

H	-0.094944000000	0.532342000000	-1.406280000000
N	-0.034904000000	0.262637000000	-0.418062000000
C	0.124906000000	1.563228000000	0.333110000000
C	1.225933000000	-0.618617000000	-0.358436000000
C	-1.378504000000	-0.432026000000	-0.159203000000
C	1.315770000000	2.373728000000	-0.151458000000
H	0.197289000000	1.319494000000	1.393184000000
H	-0.796499000000	2.123211000000	0.174534000000
H	1.279370000000	3.354647000000	0.326116000000
H	2.274602000000	1.919220000000	0.103033000000
H	1.277295000000	2.537108000000	-1.233084000000
C	0.991249000000	-1.943639000000	-1.070291000000
C	1.784998000000	-0.797964000000	1.047003000000
H	1.948719000000	-0.041171000000	-0.942296000000
H	0.556011000000	-1.811728000000	-2.065563000000
H	1.958704000000	-2.432290000000	-1.201673000000
H	0.358846000000	-2.618266000000	-0.487764000000

H 2.027509000000 0.147191000000 1.536141000000

H 1.127775000000 -1.375068000000 1.697057000000

H 2.719910000000 -1.355466000000 0.951723000000

C -2.522069000000 0.493112000000 -0.566638000000

C -1.529645000000 -0.934193000000 1.268431000000

H -1.367317000000 -1.281279000000 -0.845006000000

H -2.344697000000 0.981171000000 -1.530796000000

H -3.425166000000 -0.111258000000 -0.672313000000

H -2.728834000000 1.255995000000 0.187198000000

H -0.866834000000 -1.769137000000 1.494693000000

H -1.378694000000 -0.141564000000 2.005678000000

H -2.554539000000 -1.293378000000 1.386747000000

INTb1

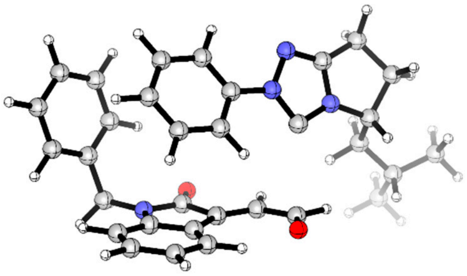

Zero-point correction= 0.584217 (Hartree/Particle)

Thermal correction to Energy= 0.617470

Thermal correction to Enthalpy= 0.618414

Thermal correction to Gibbs Free Energy= 0.516640

Sum of electronic and zero-point Energies -1606.888760

Sum of electronic and thermal Energies= -1606.855507

Sum of electronic and thermal Enthalpies= -1606.854563

Sum of electronic and thermal Free Energies= -1606.956337

E(RM062X) = -1607.85810734

C -2.164417000000 0.426001000000 -0.880841000000

C -1.743303000000 -2.409425000000 -0.678420000000

C -0.800136000000 -1.958862000000 0.366948000000

O -1.427080000000 -2.917185000000 -1.732121000000

H -2.810353000000 -2.285813000000 -0.410972000000

N -1.434999000000 1.563669000000 -1.013479000000

N -3.381909000000 0.971565000000 -0.628629000000

C 0.541301000000 -2.018743000000 0.340912000000

H -1.238952000000 -1.514229000000 1.258578000000

C 1.573767000000 -2.443708000000 -0.609193000000

C 1.282212000000 -1.418314000000 1.515718000000

C 2.815526000000 -2.073797000000 -0.044576000000

C 1.548738000000 -3.036676000000 -1.867174000000

C 4.014992000000 -2.268013000000 -0.707656000000

N 2.624209000000 -1.504091000000 1.215995000000

C 3.966791000000 -2.863705000000 -1.971459000000

H 4.956679000000 -1.953971000000 -0.271970000000

C 2.755605000000 -3.245608000000 -2.541868000000

H 4.892498000000 -3.024761000000 -2.514776000000

H 2.744051000000 -3.704830000000 -3.524152000000

H 0.602058000000 -3.314829000000 -2.310169000000

C 3.659459000000 -0.960235000000 2.073207000000

O 0.814084000000 -0.936349000000 2.528450000000

C 4.053974000000 0.450440000000 1.693110000000

H 4.524798000000 -1.630314000000 2.042835000000

H 3.247786000000 -0.976179000000 3.086103000000

N -2.124174000000 2.753137000000 -0.841727000000

C -0.039006000000 1.613090000000 -1.290403000000

C -3.325546000000 2.336790000000 -0.601562000000

C -4.694921000000 0.423440000000 -0.297834000000

C 0.669568000000 2.791946000000 -1.067609000000

C 0.599522000000 0.472533000000 -1.773201000000

C -4.660513000000 2.915845000000 -0.264495000000

C -5.588647000000 1.686114000000 -0.467590000000

H -4.665480000000 3.261589000000 0.773935000000

H -4.931682000000 3.759442000000 -0.900155000000

C -4.686220000000 -0.188878000000 1.108146000000

H -4.950032000000 -0.347799000000 -1.032502000000

H -5.997053000000 1.705755000000 -1.481365000000

H -6.424783000000 1.684927000000 0.232365000000

C -5.723057000000 -1.297207000000 1.319714000000

H -3.686848000000 -0.605715000000 1.281986000000

H -4.826867000000 0.606292000000 1.855256000000

C 2.038046000000 2.813619000000 -1.312608000000

H 0.145091000000 3.667498000000 -0.705534000000

C 1.972630000000 0.508275000000 -1.998366000000

H 0.020529000000 -0.425328000000 -1.964352000000

C -5.586338000000 -1.875697000000 2.727663000000

C -7.156523000000 -0.827987000000 1.067773000000

H -5.496211000000 -2.095101000000 0.597118000000

H -6.276966000000 -2.709845000000 2.879166000000

H -4.570391000000 -2.236571000000 2.912705000000

H -5.813452000000 -1.111385000000 3.479203000000

H -7.865490000000 -1.639036000000 1.256459000000

H -7.306581000000 -0.491877000000 0.037020000000

H -7.414345000000 0.001389000000 1.737503000000

C 2.698257000000 1.672744000000 -1.761364000000

H 2.596923000000 3.725993000000 -1.131053000000

H 3.771880000000 1.691055000000 -1.918601000000

H 2.471166000000 -0.384049000000 -2.366023000000

C 5.293682000000 0.728422000000 1.121428000000

C 3.154231000000 1.494956000000 1.923579000000

C 5.639041000000 2.036481000000 0.784643000000

H 6.001656000000 -0.079436000000 0.951259000000

C 3.501249000000 2.799785000000 1.596238000000

H 2.184530000000 1.270411000000 2.360813000000

C 4.744117000000 3.073744000000 1.025896000000

H 6.608391000000 2.243376000000 0.342280000000

H 5.013315000000 4.094332000000 0.771606000000

H 2.797248000000 3.605650000000 1.779094000000

----

^{f}INTb1

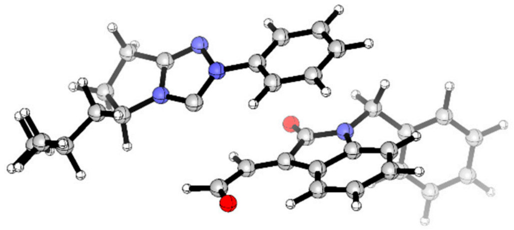

Zero-point correction= 0.583465 (Hartree/Particle)

Thermal correction to Energy= 0.617165

Thermal correction to Enthalpy= 0.618109

Thermal correction to Gibbs Free Energy= 0.512897

Sum of electronic and zero-point Energies= -1606.888529

Sum of electronic and thermal Energies= -1606.854830

Sum of electronic and thermal Enthalpies= -1606.853886

Sum of electronic and thermal Free Energies= -1606.959098

E(RM062X) = -1607.85760299

C -2.552238000000 0.159128000000 0.455458000000

C -1.481862000000 -2.074700000000 -1.147953000000

C -0.508611000000 -0.968892000000 -1.246086000000

O -1.265164000000 -3.159382000000 -0.654007000000

H -2.472054000000 -1.847221000000 -1.587124000000

N -1.861347000000 1.185393000000 1.019952000000

N -3.583617000000 0.859909000000 -0.083290000000

C 0.741573000000 -0.929353000000 -0.756288000000

H -0.855289000000 -0.058326000000 -1.731908000000

C 1.634739000000 -1.828153000000 -0.021863000000

C 1.477704000000 0.389551000000 -0.870278000000

C 2.799063000000 -1.084921000000 0.272210000000

C	1.528775000000	-3.136760000000	0.437580000000
C	3.853281000000	-1.613672000000	0.996610000000
N	2.687678000000	0.213663000000	-0.232154000000
C	3.724300000000	-2.929429000000	1.450795000000
H	4.745030000000	-1.029553000000	1.198799000000
C	2.582352000000	-3.678774000000	1.178984000000
H	4.533693000000	-3.371999000000	2.023111000000
H	2.508627000000	-4.697660000000	1.543307000000
H	0.635385000000	-3.708951000000	0.225087000000
C	3.660336000000	1.264631000000	-0.056491000000
O	1.093637000000	1.412456000000	-1.398420000000
C	4.959370000000	1.005233000000	-0.792150000000
H	3.853573000000	1.399103000000	1.015221000000
H	3.177092000000	2.175636000000	-0.425748000000
N	-2.413848000000	2.447411000000	0.873961000000
C	-0.624629000000	1.059600000000	1.709285000000
C	-3.481441000000	2.195868000000	0.188786000000
C	-4.781544000000	0.486680000000	-0.833305000000
C	-0.213673000000	-0.198724000000	2.146746000000
C	0.167083000000	2.186367000000	1.921864000000
C	-4.659041000000	2.942667000000	-0.342077000000
C	-5.310480000000	1.884891000000	-1.276484000000
H	-5.323090000000	3.223729000000	0.480936000000
H	-4.377496000000	3.854314000000	-0.870600000000
C	-5.728474000000	-0.348363000000	0.033250000000
H	-4.478576000000	-0.105747000000	-1.704289000000
H	-5.009258000000	2.076925000000	-2.309394000000
H	-6.398961000000	1.927452000000	-1.233962000000
C	-6.682068000000	-1.245129000000	-0.764075000000
H	-5.106493000000	-0.982083000000	0.675920000000
H	-6.300863000000	0.317616000000	0.696228000000
C	1.004549000000	-0.324692000000	2.805478000000
H	-0.839247000000	-1.061055000000	1.940571000000

C	1.378844000000	2.044939000000	2.590103000000
H	-0.170801000000	3.148192000000	1.557494000000
C	-7.533498000000	-2.079856000000	0.192325000000
C	-7.573736000000	-0.465829000000	-1.731241000000
H	-6.059697000000	-1.932494000000	-1.355448000000
H	-8.186101000000	-2.762874000000	-0.358935000000
H	-6.908907000000	-2.674238000000	0.865077000000
H	-8.169167000000	-1.431758000000	0.806126000000
H	-8.261810000000	-1.141300000000	-2.247731000000
H	-6.995276000000	0.062602000000	-2.495439000000
H	-8.177605000000	0.272722000000	-1.189845000000
C	1.802549000000	0.793524000000	3.033880000000
H	1.339749000000	-1.309373000000	3.118428000000
H	2.754232000000	0.686529000000	3.545569000000
H	1.997537000000	2.921335000000	2.758042000000
C	6.171209000000	1.414285000000	-0.236823000000
C	4.959661000000	0.375962000000	-2.037093000000
C	7.367567000000	1.208889000000	-0.919143000000
H	6.177622000000	1.896630000000	0.737998000000
C	6.154013000000	0.165587000000	-2.718167000000
H	4.018013000000	0.045105000000	-2.466652000000
C	7.361042000000	0.582638000000	-2.161809000000
H	8.304464000000	1.531552000000	-0.475997000000
H	8.292843000000	0.415083000000	-2.692210000000
H	6.142689000000	-0.328277000000	-3.684659000000

TSb1

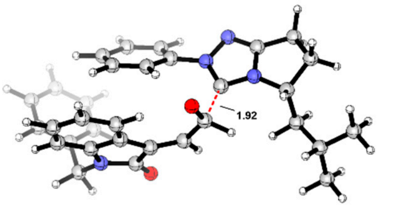

Frequency -176.4358

Zero-point correction= 0.585158 (Hartree/Particle)

Thermal correction to Energy= 0.616925

Thermal correction to Enthalpy= 0.617869

Thermal correction to Gibbs Free Energy= 0.520636

Sum of electronic and zero-point Energies= -1606.877640

Sum of electronic and thermal Energies= -1606.845873

Sum of electronic and thermal Enthalpies= -1606.844929

Sum of electronic and thermal Free Energies= -1606.942162

E(RM062X) = -1607.84660877

C	-2.184175000000	0.376681000000	-0.603149000000
C	-1.709691000000	-1.487129000000	-0.578480000000
C	-0.656045000000	-1.387600000000	0.490547000000
O	-1.472936000000	-1.875945000000	-1.742206000000
H	-2.685609000000	-1.759762000000	-0.098127000000
N	-1.543702000000	1.510243000000	-0.942415000000
N	-3.473031000000	0.766899000000	-0.623206000000
C	0.643438000000	-1.705141000000	0.416269000000
H	-0.985992000000	-0.970854000000	1.444790000000
C	1.550747000000	-2.214945000000	-0.622809000000
C	1.520041000000	-1.324121000000	1.579927000000
C	2.860462000000	-2.069721000000	-0.119788000000
C	1.363458000000	-2.721091000000	-1.903330000000
C	3.979150000000	-2.391715000000	-0.871442000000
N	2.820987000000	-1.568068000000	1.185391000000
C	3.771670000000	-2.887985000000	-2.161511000000
H	4.981388000000	-2.257816000000	-0.479684000000
C	2.485432000000	-3.056469000000	-2.667845000000
H	4.630809000000	-3.145625000000	-2.772991000000
H	2.349564000000	-3.447343000000	-3.670585000000
H	0.355234000000	-2.819154000000	-2.288561000000
C	3.963858000000	-1.167116000000	1.981604000000
O	1.193753000000	-0.850499000000	2.653454000000
C	4.439174000000	0.230934000000	1.644819000000
H	4.764820000000	-1.899746000000	1.840390000000
H	3.636520000000	-1.211409000000	3.023850000000
N	-2.372875000000	2.594281000000	-1.142285000000
C	-0.132046000000	1.665969000000	-1.112603000000
C	-3.551121000000	2.093828000000	-0.933158000000
C	-4.763063000000	0.135312000000	-0.332766000000
C	0.481322000000	2.830462000000	-0.658966000000
C	0.586288000000	0.636203000000	-1.706013000000
C	-4.971122000000	2.556282000000	-0.905148000000
C	-5.734457000000	1.203020000000	-0.914942000000
H	-5.159318000000	3.124818000000	0.010894000000
H	-5.225053000000	3.190932000000	-1.754539000000
C	-4.908389000000	-0.136761000000	1.169273000000
H	-4.821618000000	-0.805154000000	-0.890275000000
H	-5.985530000000	0.938066000000	-1.945051000000
H	-6.660994000000	1.251237000000	-0.342362000000
C	-5.850200000000	-1.299607000000	1.500382000000
H	-3.915077000000	-0.369455000000	1.572130000000
H	-5.246618000000	0.779597000000	1.675517000000
C	1.860902000000	2.942287000000	-0.776291000000
H	-0.119600000000	3.616389000000	-0.215325000000
C	1.970963000000	0.761693000000	-1.804849000000
H	0.067269000000	-0.255914000000	-2.054602000000
C	-5.892748000000	-1.517312000000	3.012301000000
C	-7.261246000000	-1.099656000000	0.946396000000

H	-5.424431000000	-2.200171000000	1.034505000000
H	-6.508031000000	-2.384636000000	3.266492000000
H	-4.891087000000	-1.680387000000	3.419980000000
H	-6.322996000000	-0.642813000000	3.513430000000
H	-7.904669000000	-1.937923000000	1.227573000000
H	-7.273419000000	-1.029134000000	-0.145998000000
H	-7.710530000000	-0.185323000000	1.353065000000
C	2.608667000000	1.904379000000	-1.332712000000
H	2.360397000000	3.834835000000	-0.413625000000
H	3.690232000000	1.985871000000	-1.383575000000
H	2.544566000000	-0.049471000000	-2.245037000000
C	5.593834000000	0.447399000000	0.894767000000
C	3.690429000000	1.328534000000	2.079828000000
C	6.002497000000	1.742913000000	0.582188000000
H	6.187072000000	-0.401335000000	0.563295000000
C	4.101487000000	2.621205000000	1.775235000000
H	2.782964000000	1.151729000000	2.651743000000
C	5.257433000000	2.832215000000	1.024878000000
H	6.906103000000	1.900201000000	0.001422000000
H	5.578422000000	3.842422000000	0.789809000000
H	3.517322000000	3.467712000000	2.123375000000

----

^{f}TSb1

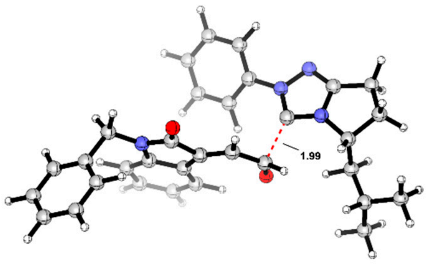

Frequency -163.2334

Zero-point correction= 0.585100 (Hartree/Particle)

Thermal correction to Energy= 0.617136

Thermal correction to Enthalpy= 0.618080

Thermal correction to Gibbs Free Energy= 0.518484

Sum of electronic and zero-point Energies= -1606.877604

Sum of electronic and thermal Energies= -1606.845568

Sum of electronic and thermal Enthalpies= -1606.844624

Sum of electronic and thermal Free Energies= -1606.944220

E(RM062X) = -1607.84505711

C	-2.574098000000	0.498910000000	-0.561135000000
C	-1.470611000000	-1.026113000000	0.080626000000
C	-0.215012000000	-0.709731000000	-0.672024000000
O	-1.553647000000	-1.051149000000	1.324347000000
H	-2.097112000000	-1.727219000000	-0.522428000000
N	-2.548255000000	1.836762000000	-0.416264000000
N	-3.880213000000	0.276596000000	-0.811152000000
C	1.010308000000	-0.457962000000	-0.192150000000
H	-0.310723000000	-0.692182000000	-1.758575000000
C	1.630036000000	-0.327646000000	1.134530000000
C	2.090261000000	-0.117174000000	-1.187889000000
C	2.954710000000	0.107592000000	0.918987000000
C	1.177307000000	-0.510911000000	2.436124000000
C	3.830511000000	0.360758000000	1.961873000000
N	3.198407000000	0.249611000000	-0.450162000000
C	3.353414000000	0.179699000000	3.263014000000
H	4.852133000000	0.673989000000	1.774289000000
C	2.049346000000	-0.249114000000	3.497341000000
H	4.016705000000	0.372138000000	4.100518000000
H	1.703998000000	-0.387378000000	4.516313000000
H	0.158827000000	-0.843542000000	2.600619000000
C	4.452422000000	0.651452000000	-1.049673000000
O	2.036168000000	-0.142938000000	-2.402887000000
C	5.483712000000	-0.455858000000	-1.039999000000
H	4.830270000000	1.537301000000	-0.526550000000

H	4.209043000000	0.935972000000	-2.077727000000
N	-3.772507000000	2.456639000000	-0.568702000000
C	-1.408554000000	2.617925000000	-0.055808000000
C	-4.565939000000	1.456953000000	-0.802294000000
C	-4.734574000000	-0.912526000000	-0.906587000000
C	-0.544470000000	2.116717000000	0.910724000000
C	-1.198688000000	3.850463000000	-0.664809000000
C	-6.029942000000	1.218042000000	-0.980809000000
C	-6.049973000000	-0.269186000000	-1.430655000000
H	-6.542403000000	1.362750000000	-0.024973000000
H	-6.483948000000	1.888497000000	-1.711274000000
C	-4.826499000000	-1.604127000000	0.461284000000
H	-4.308119000000	-1.599052000000	-1.645181000000
H	-6.067626000000	-0.316264000000	-2.522631000000
H	-6.926352000000	-0.798212000000	-1.055135000000
C	-5.093268000000	-3.110289000000	0.374247000000
H	-3.881443000000	-1.448595000000	0.997912000000
H	-5.615317000000	-1.119189000000	1.056049000000
C	0.583892000000	2.859069000000	1.248956000000
H	-0.759660000000	1.160557000000	1.383490000000
C	-0.077096000000	4.588333000000	-0.303833000000
H	-1.905159000000	4.212685000000	-1.403193000000
C	-5.069060000000	-3.714961000000	1.777100000000
C	-6.402224000000	-3.444321000000	-0.341590000000
H	-4.265426000000	-3.552246000000	-0.200035000000
H	-5.200610000000	-4.800067000000	1.739643000000
H	-4.121757000000	-3.499151000000	2.278897000000
H	-5.879438000000	-3.299963000000	2.387362000000
H	-6.572709000000	-4.524580000000	-0.353688000000
H	-6.406771000000	-3.100254000000	-1.381412000000
H	-7.251639000000	-2.979533000000	0.173962000000
C	0.816734000000	4.091569000000	0.644126000000
H	1.272923000000	2.464625000000	1.990752000000
H	1.694090000000	4.670135000000	0.914180000000
H	0.102664000000	5.550910000000	-0.770918000000
C	6.716705000000	-0.297200000000	-0.412842000000
C	5.188569000000	-1.663710000000	-1.677653000000
C	7.650111000000	-1.332591000000	-0.419027000000
H	6.950554000000	0.642989000000	0.081950000000
C	6.116859000000	-2.697030000000	-1.683041000000
H	4.226703000000	-1.777125000000	-2.172676000000
C	7.350350000000	-2.533454000000	-1.052416000000
H	8.608303000000	-1.200226000000	0.073410000000
H	8.074523000000	-3.341887000000	-1.056986000000
H	5.881841000000	-3.631905000000	-2.181870000000

INTb2

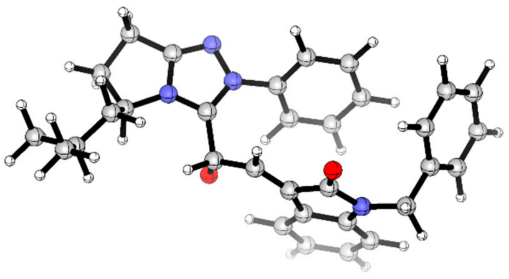

Zero-point correction= 0.585862 (Hartree/Particle)

Thermal correction to Energy= 0.617938

Thermal correction to Enthalpy= 0.618882

Thermal correction to Gibbs Free Energy= 0.521362

Sum of electronic and zero-point Energies= -1606.882556

Sum of electronic and thermal Energies= -1606.850481

Sum of electronic and thermal Enthalpies= -1606.849537

Sum of electronic and thermal Free Energies= -1606.947056

E(RM062X) = -1607.85392938

C	1.920568000000	0.187965000000	-1.329838000000
C	2.615621000000	0.183350000000	0.060169000000

C	2.869554000000	0.830840000000	-2.324424000000	C	4.774546000000	3.001658000000	0.410795000000
C	2.674791000000	1.876018000000	-3.137476000000	C	2.457284000000	3.204641000000	-0.317421000000
C	1.590526000000	2.840431000000	-3.369968000000	C	1.688352000000	-4.298738000000	-0.245721000000
C	2.137296000000	3.886178000000	-4.142582000000	H	3.116686000000	-2.713024000000	-0.481164000000
C	1.399743000000	5.006979000000	-4.489196000000	H	3.261735000000	-3.561187000000	1.057269000000
C	0.071992000000	5.067407000000	-4.055053000000	C	2.593340000000	-5.328953000000	-0.919971000000
C	-0.488028000000	4.035056000000	-3.306650000000	C	0.798554000000	-4.968577000000	0.802310000000
C	0.265870000000	2.909895000000	-2.957818000000	H	1.038116000000	-3.854698000000	-1.012635000000
H	-1.521891000000	4.105151000000	-2.985548000000	C	5.039402000000	4.259445000000	-0.116646000000
H	-0.527603000000	5.935514000000	-4.310790000000	H	5.544880000000	2.411001000000	0.894739000000
H	1.832087000000	5.812723000000	-5.072425000000	C	4.030791000000	4.982031000000	-0.756874000000
N	3.468930000000	3.598093000000	-4.461148000000	H	6.040045000000	4.673171000000	-0.051974000000
H	-0.129297000000	2.097711000000	-2.355073000000	C	2.743200000000	4.461078000000	-0.846728000000
C	3.864369000000	2.401169000000	-3.895401000000	H	4.261029000000	5.947499000000	-1.198294000000
C	4.393772000000	4.463594000000	-5.164387000000	H	1.958477000000	5.012249000000	-1.357043000000
O	4.982456000000	1.925741000000	-3.987492000000	H	1.483900000000	2.728859000000	-0.429986000000
H	3.868933000000	0.388678000000	-2.375529000000	H	2.003090000000	-6.119410000000	-1.391165000000
O	0.723908000000	0.679677000000	-1.083653000000	H	3.218342000000	-4.867464000000	-1.689695000000
H	1.958838000000	-0.906480000000	-1.615085000000	H	3.255130000000	-5.798716000000	-0.183616000000
N	3.199426000000	1.170043000000	0.758287000000	H	0.229081000000	-5.787208000000	0.353965000000
N	2.404164000000	-0.771829000000	0.975982000000	H	0.078568000000	-4.271450000000	1.242657000000
N	3.359355000000	0.863185000000	2.092758000000	H	1.405287000000	-5.391371000000	1.612447000000
C	3.486898000000	2.492372000000	0.283119000000	C	5.111477000000	5.424815000000	-4.239604000000
C	2.856947000000	-0.330266000000	2.182835000000	C	4.701828000000	6.751896000000	-4.111789000000
C	2.592016000000	-1.360681000000	3.231981000000	C	6.197436000000	4.969667000000	-3.486045000000
C	1.705724000000	-2.058127000000	1.000892000000	C	5.368071000000	7.619025000000	-3.247564000000
C	1.498510000000	-2.220755000000	2.536791000000	H	3.862948000000	7.114977000000	-4.700062000000
H	3.500422000000	-1.937884000000	3.430078000000	C	6.457039000000	7.163372000000	-2.508546000000
H	2.254947000000	-0.923019000000	4.171412000000	H	5.042216000000	8.650530000000	-3.158721000000
H	0.512531000000	-1.836590000000	2.809015000000	C	6.870332000000	5.837409000000	-2.631635000000
H	1.551010000000	-3.267629000000	2.836272000000	H	6.984160000000	7.839866000000	-1.843193000000
C	2.536313000000	-3.160717000000	0.333796000000	H	7.722310000000	5.478952000000	-2.061642000000
H	0.757413000000	-1.915698000000	0.470642000000	H	6.496588000000	3.928645000000	-3.574650000000

H 3.843094000000 5.005028000000 -5.939802000000

H 5.118429000000 3.806246000000 -5.652224000000

----

^{f}INTb2

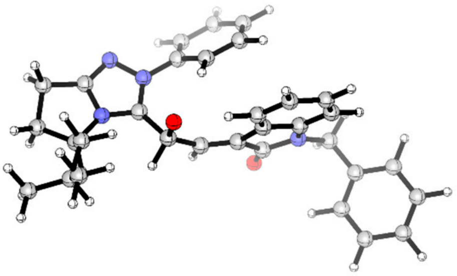

Zero-point correction= 0.586182 (Hartree/Particle)

Thermal correction to Energy= 0.618407

Thermal correction to Enthalpy= 0.619351

Thermal correction to Gibbs Free Energy= 0.519528

Sum of electronic and zero-point Energies= -1606.883945

Sum of electronic and thermal Energies= -1606.851720

Sum of electronic and thermal Enthalpies= -1606.850776

Sum of electronic and thermal Free Energies= -1606.950599

E(RM062X) = -1607.85314955

C -1.564383000000 -0.629354000000 -0.201827000000

C -2.576812000000 0.503975000000 -0.523103000000

C -0.235799000000 -0.303131000000 -0.852731000000

C 0.987912000000 -0.258868000000 -0.311893000000

C 1.563191000000 -0.398776000000 1.034591000000

C 2.920117000000 -0.028599000000 0.933499000000

C 3.769829000000 -0.020745000000 2.027811000000

C 3.231339000000 -0.392853000000 3.262589000000

C 1.895168000000 -0.767216000000 3.381723000000

C 1.049018000000 -0.779929000000 2.268463000000

H 1.505255000000 -1.061698000000 4.350436000000

H 3.872464000000 -0.397717000000 4.138661000000

H 4.816419000000 0.246245000000 1.925856000000

N 3.227171000000 0.318756000000 -0.386406000000

H 0.003987000000 -1.068831000000 2.325697000000

C 2.126975000000 0.158702000000 -1.206600000000

C 4.520832000000 0.750716000000 -0.865374000000

O 2.120340000000 0.343836000000 -2.409500000000

H -0.271037000000 -0.092309000000 -1.925372000000

O -1.664557000000 -0.813719000000 1.103568000000

H -1.960793000000 -1.468796000000 -0.845557000000

N -2.524169000000 1.827868000000 -0.306171000000

N -3.873048000000 0.274142000000 -0.781243000000

N -3.753788000000 2.436982000000 -0.409220000000

C -1.386221000000 2.599302000000 0.095916000000

C -4.551523000000 1.451597000000 -0.690149000000

C -6.018873000000 1.223228000000 -0.854803000000

C -4.722409000000 -0.915827000000 -0.925201000000

C -6.054657000000 -0.243654000000 -1.368369000000

H -6.512440000000 1.330536000000 0.115451000000

H -6.480846000000 1.927023000000 -1.547936000000

H -6.118212000000 -0.244125000000 -2.459475000000

H -6.914446000000 -0.789558000000 -0.979482000000

C -4.755285000000 -1.694538000000 0.396393000000

H -4.311379000000 -1.545203000000 -1.721198000000

C -0.572229000000 2.102492000000 1.106317000000

C -1.143789000000 3.817102000000 -0.530017000000

C -5.029755000000 -3.190162000000 0.211495000000

H -3.783737000000 -1.565084000000 0.896244000000

H -5.520295000000 -1.251420000000 1.051931000000

C -4.980428000000 -3.889423000000 1.569023000000

C -6.354550000000 -3.474489000000 -0.497397000000

H -4.215077000000 -3.593236000000 -0.407999000000

C 0.551016000000 2.840933000000 1.470077000000

H -0.815796000000 1.140663000000 1.557984000000

C 0.819978000000 4.059159000000 0.852031000000

H 1.213336000000 2.448539000000 2.236268000000

C -0.030399000000 4.550501000000 -0.138658000000

H 1.695333000000 4.631542000000 1.141456000000

H 0.179648000000 5.501533000000 -0.616166000000

H -1.815839000000 4.170358000000 -1.304326000000

H -5.114956000000 -4.969407000000 1.459809000000

H -4.024168000000 -3.709142000000 2.067181000000

H -5.778676000000 -3.516399000000 2.220921000000

H -6.525477000000 -4.551509000000 -0.581731000000

H -6.383043000000 -3.058990000000 -1.510834000000

H -7.192994000000 -3.048641000000 0.067779000000

C 5.520944000000 -0.381645000000 -0.959690000000

C 6.740986000000 -0.327811000000 -0.290874000000

C 5.215198000000 -1.501355000000 -1.737327000000

C 7.650220000000 -1.379373000000 -0.393091000000

H 6.983231000000 0.543020000000 0.314437000000

C 7.339772000000 -2.492568000000 -1.166420000000

H 8.597081000000 -1.328884000000 0.135226000000

C 6.119650000000 -2.551090000000 -1.839625000000

H 8.044925000000 -3.313746000000 -1.245731000000

H 5.874692000000 -3.417307000000 -2.446057000000

H 4.264203000000 -1.534877000000 -2.263940000000

H 4.899621000000 1.541609000000 -0.207152000000

H 4.339386000000 1.182845000000 -1.854309000000

INTb3''

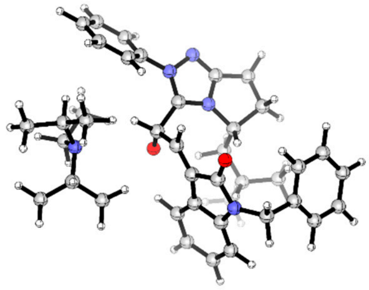

Zero-point correction= 0.852126 (Hartree/Particle)

Thermal correction to Energy= 0.897325

Thermal correction to Enthalpy= 0.898269

Thermal correction to Gibbs Free Energy= 0.773534

Sum of electronic and zero-point Energies= -1977.505899

Sum of electronic and thermal Energies= -1977.460701

Sum of electronic and thermal Enthalpies= -1977.459756

Sum of electronic and thermal Free Energies= -1977.584491

E(RM062X) = -1978.82526378

C -1.232316000000 0.172989000000 -0.095132000000

C -0.933719000000 1.705798000000 -0.031954000000

C -0.335902000000 -0.379366000000 0.999378000000

C 0.727715000000 -1.186308000000 0.873818000000

C 1.376081000000 -1.909109000000 -0.231083000000

C 2.639349000000 -2.316724000000 0.241808000000

C 3.544853000000 -2.993567000000 -0.560415000000

C 3.147793000000 -3.300493000000 -1.864762000000

C 1.887386000000 -2.940042000000 -2.335530000000

C 0.991641000000 -2.234809000000 -1.527529000000

H 1.602633000000 -3.193356000000 -3.351586000000

H 3.835030000000 -3.833076000000 -2.515008000000

H 4.523729000000 -3.281721000000 -0.192629000000

N 2.794449000000 -1.946064000000 1.585350000000

H 0.027684000000 -1.886559000000 -1.881279000000

C 1.673138000000 -1.277357000000 2.041146000000

C 4.055659000000 -1.896970000000 2.298446000000

O	1.559391000000	-0.785010000000	3.150399000000
H	-0.494652000000	0.018638000000	2.006898000000
O	-1.024652000000	-0.166395000000	-1.352218000000
H	-2.282142000000	0.059878000000	0.269943000000
N	-1.784112000000	2.744394000000	-0.009317000000
N	0.255641000000	2.270811000000	-0.317715000000
N	-1.183359000000	3.946710000000	-0.305874000000
C	-3.174825000000	2.726029000000	0.307084000000
C	0.057702000000	3.611357000000	-0.483537000000
C	1.340429000000	4.301287000000	-0.807095000000
C	1.654064000000	1.853735000000	-0.600486000000
C	2.365257000000	3.217056000000	-0.391546000000
H	1.385845000000	4.515860000000	-1.879263000000
H	1.456634000000	5.240267000000	-0.265372000000
H	2.611342000000	3.337869000000	0.667509000000
H	3.286909000000	3.278123000000	-0.967026000000
C	1.754958000000	1.278365000000	-2.015911000000
H	1.962471000000	1.112906000000	0.143159000000
C	-4.049046000000	3.442887000000	-0.503103000000
C	-3.621056000000	2.020195000000	1.418622000000
C	3.013438000000	0.440215000000	-2.282851000000
H	0.867166000000	0.649238000000	-2.165413000000
H	1.703995000000	2.111041000000	-2.735993000000
C	2.997512000000	-0.021102000000	-3.739230000000
C	4.333490000000	1.135745000000	-1.953880000000
H	2.953379000000	-0.450109000000	-1.647844000000
C	-5.405264000000	3.431585000000	-0.200586000000
H	-3.659199000000	3.989327000000	-1.354437000000
C	-5.874713000000	2.709827000000	0.895546000000
H	-6.097907000000	3.980892000000	-0.828962000000
C	-4.983234000000	2.009517000000	1.703917000000
H	-6.935253000000	2.698450000000	1.123305000000
H	-5.341937000000	1.455584000000	2.565372000000
H	-2.912243000000	1.497822000000	2.053154000000
H	3.809677000000	-0.729054000000	-3.930678000000
H	2.052925000000	-0.516123000000	-3.982011000000
H	3.122019000000	0.832417000000	-4.416903000000
H	5.173709000000	0.483089000000	-2.211368000000
H	4.425867000000	1.362179000000	-0.886526000000
H	4.450815000000	2.066891000000	-2.524166000000
C	4.883958000000	-0.699627000000	1.875525000000
C	6.047004000000	-0.846562000000	1.123410000000
C	4.458437000000	0.586335000000	2.230950000000
C	6.787017000000	0.270093000000	0.735667000000
H	6.384224000000	-1.842035000000	0.845523000000
C	6.366110000000	1.544429000000	1.099892000000
H	7.693083000000	0.141721000000	0.151897000000
C	5.198055000000	1.700479000000	1.846861000000
H	6.944494000000	2.414107000000	0.804551000000
H	4.873989000000	2.693000000000	2.145873000000
H	3.552564000000	0.694322000000	2.823415000000
H	4.600828000000	-2.831031000000	2.131434000000
H	3.801987000000	-1.830966000000	3.359628000000
N	-4.043465000000	-1.917294000000	-0.147260000000
C	-4.527292000000	-1.473535000000	-1.451725000000
C	-4.298846000000	0.013880000000	-1.701439000000
H	-5.592906000000	-1.728836000000	-1.603660000000
H	-3.970035000000	-2.022058000000	-2.216440000000
H	-4.741528000000	0.301804000000	-2.660920000000
H	-4.765887000000	0.636502000000	-0.930774000000
H	-3.221202000000	0.208275000000	-1.737603000000
C	-4.905311000000	-1.523219000000	0.967676000000
C	-4.113533000000	-1.460445000000	2.275822000000
C	-6.177722000000	-2.363618000000	1.156874000000
H	-5.222233000000	-0.499055000000	0.737968000000
H	-3.169656000000	-0.929329000000	2.122808000000

H	-4.691954000000	-0.951081000000	3.054226000000
H	-3.875998000000	-2.458716000000	2.656891000000
H	-6.759800000000	-2.425994000000	0.232501000000
H	-5.932849000000	-3.381335000000	1.479083000000
H	-6.817154000000	-1.918187000000	1.926032000000
C	-3.555274000000	-3.304562000000	-0.121132000000
C	-2.112885000000	-3.341750000000	-0.621309000000
C	-4.416466000000	-4.325209000000	-0.884060000000
H	-3.547999000000	-3.610869000000	0.931626000000
H	-1.477798000000	-2.713433000000	0.007536000000
H	-1.719077000000	-4.363599000000	-0.623735000000
H	-2.049827000000	-2.944216000000	-1.639123000000
H	-5.470578000000	-4.271030000000	-0.601669000000
H	-4.347391000000	-4.173367000000	-1.965613000000
H	-4.059630000000	-5.338307000000	-0.674945000000

INTb3

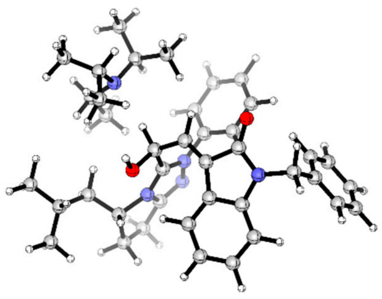

Zero-point correction=	0.866910 (Hartree/Particle)
Thermal correction to Energy=	0.912348
Thermal correction to Enthalpy=	0.913292
Thermal correction to Gibbs Free Energy=	0.787101
Sum of electronic and zero-point Energies=	-1977.939807
Sum of electronic and thermal Energies=	-1977.894368
Sum of electronic and thermal Enthalpies=	-1977.893424
Sum of electronic and thermal Free Energies=	-1978.019616

E(RM062X) = -1979.26664939

N	-3.437932000000	2.173900000000	0.648542000000
H	-1.777625000000	0.689333000000	0.613059000000
C	-1.007412000000	-0.104763000000	0.703197000000
C	-4.430579000000	1.436241000000	-0.134529000000
C	-2.999899000000	3.443875000000	0.033692000000
C	-3.820644000000	2.290305000000	2.077273000000
C	-0.785378000000	-0.629936000000	-0.691069000000
C	0.237717000000	0.498556000000	1.303896000000
O	-1.508557000000	-1.215434000000	1.426324000000
C	-3.960801000000	1.041422000000	-1.531584000000
H	-5.386572000000	1.976317000000	-0.227672000000
H	-4.657769000000	0.518792000000	0.423538000000
C	-1.638977000000	3.867384000000	0.583421000000
C	-4.017295000000	4.586646000000	0.117060000000
H	-2.840744000000	3.218759000000	-1.026880000000
C	-3.270047000000	1.096419000000	2.860324000000
C	-5.320755000000	2.458427000000	2.353775000000
H	-3.321227000000	3.184574000000	2.461439000000
N	-0.226024000000	0.001643000000	-1.729905000000
N	-1.234353000000	-1.788242000000	-1.185724000000
C	1.374300000000	-0.152725000000	1.574212000000
H	0.233090000000	1.568931000000	1.501000000000
H	-1.698686000000	-0.915237000000	2.324765000000
N	-0.323618000000	-0.720234000000	-2.887489000000
C	0.275979000000	1.352972000000	-1.784080000000
C	-0.940250000000	-1.804470000000	-2.521727000000
C	-2.077032000000	-2.932833000000	-0.755446000000
C	1.865139000000	-1.515756000000	1.372885000000
C	2.584557000000	0.675039000000	1.962033000000
C	-0.524514000000	2.300462000000	-2.414342000000
C	1.527224000000	1.667086000000	-1.264991000000
C	-1.471948000000	-3.038628000000	-3.170526000000

C	-1.823281000000	-3.907964000000	-1.935612000000
C	-3.526346000000	-2.469530000000	-0.570528000000
H	-1.688030000000	-3.318541000000	0.187193000000
C	3.261479000000	-1.471053000000	1.567282000000
C	1.254480000000	-2.721768000000	1.048199000000
N	3.659226000000	-0.185314000000	1.951385000000
O	2.620280000000	1.873301000000	2.149680000000
C	4.059689000000	-2.588689000000	1.388153000000
C	2.045707000000	-3.857432000000	0.876656000000
H	0.174978000000	-2.775600000000	0.974379000000
C	5.037097000000	0.262736000000	2.081864000000
C	3.428801000000	-3.783373000000	1.034441000000
H	5.135613000000	-2.538598000000	1.510094000000
H	1.583006000000	-4.807322000000	0.632087000000
H	4.031345000000	-4.674344000000	0.891700000000
C	5.696479000000	0.404792000000	0.727559000000
H	5.581826000000	-0.441246000000	2.718650000000
H	4.989650000000	1.225457000000	2.598235000000
C	6.817598000000	-0.339854000000	0.374124000000
C	5.142571000000	1.292674000000	-0.199007000000
C	7.378758000000	-0.204862000000	-0.895695000000
H	7.260466000000	-1.024604000000	1.093740000000
C	5.694759000000	1.424014000000	-1.466596000000
H	4.284319000000	1.889484000000	0.103173000000
C	-0.075089000000	3.614252000000	-2.487767000000
H	-1.475082000000	2.001054000000	-2.844224000000
C	1.953231000000	2.990621000000	-1.328087000000
H	2.157055000000	0.895179000000	-0.834708000000
H	-2.354793000000	-2.784307000000	-3.764433000000
H	-0.743475000000	-3.507851000000	-3.831928000000
H	-0.974077000000	-4.552029000000	-1.693008000000
H	-2.691660000000	-4.541226000000	-2.115003000000
C	-4.310538000000	-3.319252000000	0.435716000000
H	-3.514528000000	-1.437040000000	-0.202549000000
H	-4.036912000000	-2.462780000000	-1.545005000000
C	1.154082000000	3.959598000000	-1.930580000000
H	-0.686880000000	4.365658000000	-2.975381000000
H	2.914926000000	3.265821000000	-0.909187000000
C	-5.724167000000	-2.758656000000	0.584812000000
C	-4.352358000000	-4.801507000000	0.062629000000
H	-3.793688000000	-3.219204000000	1.401131000000
H	-6.285167000000	-3.308841000000	1.344193000000
H	-5.707419000000	-1.703389000000	0.877317000000
H	-6.272018000000	-2.839216000000	-0.360333000000
H	-4.946121000000	-5.361583000000	0.789346000000
H	-3.355968000000	-5.255274000000	0.040014000000
H	-4.815974000000	-4.942394000000	-0.921077000000
H	1.498020000000	4.987300000000	-1.976150000000
C	6.816260000000	0.671441000000	-1.817252000000
H	8.254231000000	-0.787084000000	-1.163262000000
H	5.260533000000	2.117567000000	-2.180440000000
H	7.252713000000	0.775242000000	-2.805011000000
H	-4.650802000000	0.307562000000	-1.956875000000
H	-3.929377000000	1.891729000000	-2.217179000000
H	-2.960634000000	0.591871000000	-1.500363000000
H	-0.891365000000	3.089416000000	0.398262000000
H	-1.298487000000	4.779472000000	0.085256000000
H	-1.668209000000	4.075148000000	1.657214000000
H	-4.992638000000	4.289874000000	-0.280501000000
H	-4.154150000000	4.926131000000	1.148895000000
H	-3.668677000000	5.443278000000	-0.466143000000
H	-2.173197000000	1.123432000000	2.862861000000
H	-3.608947000000	1.116599000000	3.899436000000
H	-3.611754000000	0.153995000000	2.411997000000
H	-5.759257000000	3.266831000000	1.764656000000
H	-5.878157000000	1.539984000000	2.144924000000

H -5.468163000000 2.696243000000 3.410348000000

TSb2'

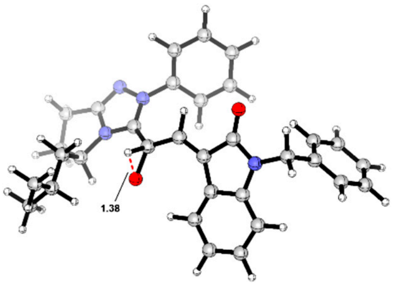

Frequency -1722.3655

Zero-point correction= 0.581662 (Hartree/Particle)

Thermal correction to Energy= 0.614075

Thermal correction to Enthalpy= 0.615019

Thermal correction to Gibbs Free Energy= 0.514270

Sum of electronic and zero-point Energies= -1606.820747

Sum of electronic and thermal Energies= -1606.788334

Sum of electronic and thermal Enthalpies= -1606.787390

Sum of electronic and thermal Free Energies= -1606.888139

E(RM062X) = -1607.78748761

O 1.941322000000 -1.510850000000 0.201099000000

H 2.144656000000 -0.936105000000 -1.036521000000

C 1.563469000000 -0.229971000000 -0.316342000000

C 2.419223000000 0.849386000000 0.122110000000

C 0.217209000000 0.082987000000 -0.760297000000

N 2.288779000000 2.203808000000 0.150103000000

N 3.687479000000 0.648449000000 0.548307000000

C -0.918319000000 -0.640146000000 -0.664387000000

H 0.095362000000 1.015696000000 -1.311461000000

C -1.337449000000 -1.894486000000 -0.032864000000

C -2.140212000000 -0.066941000000 -1.309267000000

C -2.711315000000 -2.049915000000 -0.317732000000

C -0.694485000000 -2.844372000000 0.752104000000

C -3.450374000000 -3.121705000000 0.154075000000

N -3.157085000000 -0.980941000000 -1.102307000000

C -2.782591000000 -4.072651000000 0.930308000000

H -4.511920000000 -3.207534000000 -0.053663000000

C -1.427366000000 -3.937348000000 1.221998000000

H -3.335891000000 -4.922422000000 1.318165000000

H -0.934143000000 -4.686913000000 1.832337000000

H 0.359415000000 -2.712288000000 0.973126000000

C -4.510514000000 -0.773875000000 -1.564363000000

O -2.270403000000 0.998998000000 -1.891852000000

C -5.436860000000 -0.293758000000 -0.468133000000

H -4.886339000000 -1.705487000000 -2.002772000000

H -4.439677000000 -0.022020000000 -2.356399000000

N 3.462512000000 2.842905000000 0.509944000000

C 1.097609000000 2.980979000000 0.015021000000

C 4.277401000000 1.861823000000 0.738365000000

C 4.587561000000 -0.500062000000 0.718217000000

C 1.090849000000 4.058365000000 -0.862730000000

C -0.026259000000 2.644909000000 0.766169000000

C 5.710916000000 1.682818000000 1.125947000000

C 5.735829000000 0.178841000000 1.512897000000

H 6.351402000000 1.894159000000 0.263913000000

H 6.011615000000 2.342122000000 1.940536000000

C 4.994759000000 -1.087103000000 -0.640846000000

H 4.051757000000 -1.253539000000 1.293871000000

H 5.541418000000 0.080120000000 2.584154000000

H 6.698178000000 -0.288651000000 1.298555000000

C 5.149055000000 -2.609439000000 -0.592542000000

H 4.228307000000 -0.836563000000 -1.383138000000

H 5.928504000000 -0.619366000000 -0.987650000000

C -0.077632000000 4.799275000000 -1.007496000000

H 1.989799000000 4.299468000000 -1.419426000000

C	-1.193368000000	3.377713000000	0.593071000000
H	0.011816000000	1.802256000000	1.448734000000
C	5.464522000000	-3.156538000000	-1.982540000000
C	6.218547000000	-3.039976000000	0.412047000000
H	4.174132000000	-2.999739000000	-0.271349000000
H	5.536491000000	-4.247615000000	-1.966155000000
H	4.688720000000	-2.880779000000	-2.702863000000
H	6.420975000000	-2.763898000000	-2.347205000000
H	6.313037000000	-4.129251000000	0.435030000000
H	5.987872000000	-2.710417000000	1.430671000000
H	7.196927000000	-2.627213000000	0.134965000000
C	-1.220879000000	4.452689000000	-0.292422000000
H	-0.097735000000	5.638957000000	-1.693681000000
H	-2.136415000000	5.018339000000	-0.427463000000
H	-2.081088000000	3.107292000000	1.154407000000
C	-6.575971000000	-1.012213000000	-0.115248000000
C	-5.142737000000	0.896523000000	0.202525000000
C	-7.415336000000	-0.554057000000	0.898909000000
H	-6.809046000000	-1.937388000000	-0.637587000000
C	-5.977479000000	1.354014000000	1.214740000000
H	-4.257255000000	1.456866000000	-0.090194000000
C	-7.115998000000	0.628275000000	1.566243000000
H	-8.298951000000	-1.123760000000	1.168621000000
H	-7.766582000000	0.986191000000	2.357816000000
H	-5.745330000000	2.280497000000	1.731086000000

TSb2''

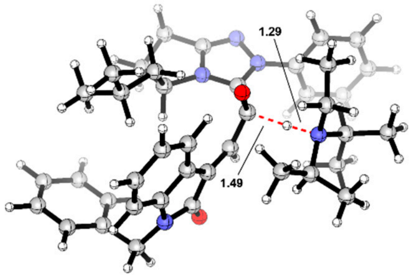

Frequency -1252.0245

Zero-point correction= 0.849895 (Hartree/Particle)

Thermal correction to Energy= 0.893854

Thermal correction to Enthalpy= 0.894799

Thermal correction to Gibbs Free Energy= 0.772913

Sum of electronic and zero-point Energies= -1977.468932

Sum of electronic and thermal Energies= -1977.424973

Sum of electronic and thermal Enthalpies= -1977.424029

Sum of electronic and thermal Free Energies= -1977.545914

E(RM062X) = -1978.78749882

N	3.135958000000	-1.904088000000	0.344030000000
H	2.315397000000	-0.923600000000	0.155406000000
C	1.200715000000	0.042654000000	0.363608000000
C	1.375898000000	1.527103000000	0.087242000000
C	0.271793000000	-0.451492000000	-0.615603000000
O	1.063471000000	-0.086117000000	1.686201000000
N	2.439184000000	2.341043000000	-0.072050000000
N	0.355705000000	2.397694000000	0.254580000000
C	-0.857582000000	-1.229173000000	-0.482836000000
H	0.486469000000	-0.170433000000	-1.652352000000
C	-1.673019000000	-1.676824000000	0.645346000000
C	-1.639313000000	-1.486665000000	-1.699955000000
C	-2.890982000000	-2.149809000000	0.100677000000
C	-1.514776000000	-1.689791000000	2.032199000000
C	-3.934570000000	-2.604322000000	0.892885000000
N	-2.842919000000	-2.065955000000	-1.290790000000

C	-3.749412000000	-2.615796000000	2.276947000000
H	-4.868362000000	-2.938677000000	0.451974000000
C	-2.550088000000	-2.174242000000	2.833958000000
H	-4.549789000000	-2.968386000000	2.919825000000
H	-2.426190000000	-2.182118000000	3.912808000000
H	-0.602965000000	-1.279696000000	2.457929000000
C	-4.004130000000	-2.069393000000	-2.155155000000
O	-1.383058000000	-1.209555000000	-2.870446000000
C	-4.806331000000	-0.793929000000	-1.988329000000
H	-4.619898000000	-2.949102000000	-1.940649000000
H	-3.619580000000	-2.152463000000	-3.175159000000
N	2.125713000000	3.682258000000	0.002499000000
C	3.760820000000	2.008563000000	-0.504434000000
C	0.843592000000	3.665321000000	0.209131000000
C	-1.111474000000	2.324734000000	0.446781000000
C	4.823160000000	2.145020000000	0.381164000000
C	3.960667000000	1.649947000000	-1.833068000000
C	-0.256667000000	4.659777000000	0.371835000000
C	-1.491849000000	3.779804000000	0.049140000000
H	-0.276691000000	5.023742000000	1.403697000000
H	-0.147974000000	5.517440000000	-0.292330000000
C	-1.457261000000	1.928653000000	1.882308000000
H	-1.509283000000	1.583061000000	-0.253703000000
H	-1.697390000000	3.823603000000	-1.024408000000
H	-2.379749000000	4.115586000000	0.581438000000
C	-2.886531000000	1.393059000000	2.059876000000
H	-0.738657000000	1.152485000000	2.181843000000
H	-1.300781000000	2.800577000000	2.537928000000
C	6.111195000000	1.867766000000	-0.065545000000
H	4.628599000000	2.448010000000	1.404342000000
C	5.254528000000	1.388812000000	-2.274538000000
H	3.109059000000	1.581562000000	-2.502949000000
C	-3.121144000000	1.103063000000	3.541577000000

C	-3.986513000000	2.308289000000	1.520395000000
H	-2.948572000000	0.444398000000	1.512285000000
H	-4.075936000000	0.590910000000	3.691899000000
H	-2.329652000000	0.466075000000	3.945481000000
H	-3.138165000000	2.037122000000	4.116972000000
H	-4.967881000000	1.862093000000	1.708114000000
H	-3.912238000000	2.454624000000	0.437913000000
H	-3.966649000000	3.290405000000	2.011532000000
C	6.325775000000	1.485892000000	-1.389245000000
H	6.947644000000	1.954378000000	0.619768000000
H	7.333200000000	1.277541000000	-1.734701000000
H	5.424220000000	1.112017000000	-3.309887000000
C	-6.073389000000	-0.795922000000	-1.411556000000
C	-4.231455000000	0.424683000000	-2.368862000000
C	-6.764839000000	0.398806000000	-1.212363000000
H	-6.523305000000	-1.739165000000	-1.110598000000
C	-4.922172000000	1.615368000000	-2.174819000000
H	-3.242207000000	0.416176000000	-2.821270000000
C	-6.190032000000	1.606262000000	-1.592138000000
H	-7.750211000000	0.383397000000	-0.757031000000
H	-6.725039000000	2.537980000000	-1.436623000000
H	-4.473859000000	2.556361000000	-2.482012000000
C	3.533150000000	-1.872597000000	1.776229000000
C	4.256028000000	-1.560502000000	-0.587752000000
C	2.333191000000	-3.123092000000	-0.017817000000
C	4.135964000000	-0.545399000000	2.205373000000
H	4.214752000000	-2.708204000000	1.984565000000
H	2.619329000000	-2.004882000000	2.350199000000
H	4.335491000000	-0.591639000000	3.279238000000
H	5.074634000000	-0.306160000000	1.698755000000
H	3.391240000000	0.238437000000	2.037573000000
C	3.802535000000	-1.661026000000	-2.041412000000
C	5.579665000000	-2.289234000000	-0.349260000000

H 4.443662000000 -0.501526000000 -0.384675000000

H 2.863063000000 -1.121570000000 -2.198027000000

H 4.565903000000 -1.210663000000 -2.679897000000

H 3.665610000000 -2.694911000000 -2.366980000000

H 5.927440000000 -2.184805000000 0.681080000000

H 5.535540000000 -3.351527000000 -0.592918000000

H 6.333442000000 -1.828322000000 -0.995354000000

C 1.220015000000 -3.349701000000 1.001562000000

C 3.151144000000 -4.403269000000 -0.190509000000

H 1.855704000000 -2.864072000000 -0.968876000000

H 0.812118000000 -2.400412000000 1.349041000000

H 0.413547000000 -3.917223000000 0.531136000000

H 1.581217000000 -3.920826000000 1.863368000000

H 3.791972000000 -4.396589000000 -1.073206000000

H 3.770335000000 -4.600824000000 0.690536000000

H 2.454813000000 -5.239854000000 -0.295197000000

TSb2

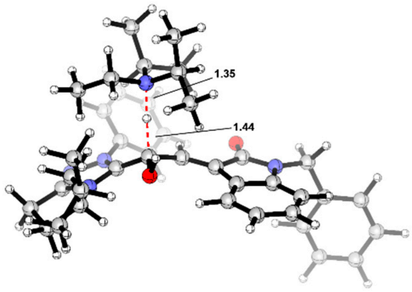

Frequency -1269.4558

Zero-point correction= 0.862683 (Hartree/Particle)

Thermal correction to Energy= 0.907941

Thermal correction to Enthalpy= 0.908885

Thermal correction to Gibbs Free Energy= 0.782087

Sum of electronic and zero-point Energies= -1977.921555

Sum of electronic and thermal Energies= -1977.876297

Sum of electronic and thermal Enthalpies= -1977.875353

Sum of electronic and thermal Free Energies= -1978.002151

E(RM062X) = -1979.24367816

N 1.931386000000 -0.820168000000 2.248036000000

H 1.511613000000 -0.347874000000 1.053256000000

C 1.034533000000 0.217901000000 -0.183617000000

C 3.341066000000 -0.351122000000 2.307966000000

C 1.826973000000 -2.314610000000 2.092805000000

C 1.096643000000 -0.236058000000 3.356719000000

C 1.905208000000 -0.232117000000 -1.280300000000

C -0.327227000000 -0.318736000000 -0.171304000000

O 1.130815000000 1.630153000000 -0.120528000000

C 4.236316000000 -0.884840000000 1.194287000000

H 3.780803000000 -0.612131000000 3.274738000000

H 3.310340000000 0.743523000000 2.260985000000

C 0.381580000000 -2.812326000000 2.066527000000

C 2.633476000000 -3.138710000000 3.097842000000

H 2.247069000000 -2.489999000000 1.097503000000

C 0.653101000000 1.186448000000 3.019959000000

C 1.766461000000 -0.263194000000 4.732482000000

H 0.196584000000 -0.851961000000 3.392808000000

N 2.116241000000 -1.458409000000 -1.819107000000

N 2.819048000000 0.551603000000 -1.892163000000

C -1.521778000000 0.225423000000 0.125523000000

H -0.388859000000 -1.392513000000 -0.313642000000

H 1.243576000000 1.884950000000 0.802208000000

N 3.159798000000 -1.463636000000 -2.719614000000

C 1.474007000000 -2.720552000000 -1.567829000000

C 3.557403000000 -0.232958000000 -2.736498000000

C 3.260289000000 1.967822000000 -1.876595000000

C -2.107685000000 1.540203000000 0.419844000000

C -2.649288000000 -0.769266000000 0.246159000000

C 2.255470000000 -3.750242000000 -1.047941000000

C	0.140222000000	-2.920115000000	-1.905973000000	H	4.914642000000	2.728739000000	-3.099725000000
C	4.611219000000	0.568044000000	-3.425094000000	C	3.912424000000	3.735496000000	-0.145256000000
C	4.099338000000	2.009659000000	-3.181550000000	H	3.668033000000	1.633751000000	0.203998000000
C	4.045810000000	2.277334000000	-0.597011000000	H	5.106097000000	2.020893000000	-0.739422000000
H	2.375681000000	2.600847000000	-1.937187000000	C	0.316862000000	-5.185772000000	-1.096091000000
C	-3.467628000000	1.308564000000	0.720607000000	H	2.267992000000	-5.801181000000	-0.415565000000
C	-1.633982000000	2.848855000000	0.420502000000	H	-1.494866000000	-4.303198000000	-1.862813000000
N	-3.761883000000	-0.058033000000	0.623612000000	C	4.639418000000	3.935023000000	1.183475000000
O	-2.590698000000	-1.975350000000	0.069816000000	C	4.418722000000	4.725250000000	-1.194924000000
C	-4.342247000000	2.332162000000	1.046713000000	H	2.839262000000	3.920250000000	0.011849000000
C	-2.501678000000	3.889222000000	0.755111000000	H	4.507606000000	4.954002000000	1.555485000000
H	-0.610404000000	3.056626000000	0.135410000000	H	4.267993000000	3.246358000000	1.950712000000
C	-5.067932000000	-0.651733000000	0.825292000000	H	5.714044000000	3.758080000000	1.066410000000
C	-3.834621000000	3.632278000000	1.068096000000	H	4.326880000000	5.751471000000	-0.830477000000
H	-5.387819000000	2.130348000000	1.253191000000	H	3.857039000000	4.661106000000	-2.132396000000
H	-2.136832000000	4.910436000000	0.756169000000	H	5.477347000000	4.545380000000	-1.416402000000
H	-4.496728000000	4.454880000000	1.316742000000	H	-0.140651000000	-6.150184000000	-0.903248000000
C	-6.096088000000	-0.174661000000	-0.179994000000	C	-8.015131000000	0.652708000000	-2.035532000000
H	-5.410034000000	-0.435811000000	1.844388000000	H	-9.394718000000	0.620547000000	-0.384166000000
H	-4.912846000000	-1.732264000000	0.743713000000	H	-6.414767000000	0.599877000000	-3.472985000000
C	-7.413819000000	0.044101000000	0.218798000000	H	-8.758760000000	0.977524000000	-2.755729000000
C	-5.743606000000	0.028807000000	-1.514357000000	H	5.191369000000	-0.355143000000	1.233225000000
C	-8.371985000000	0.452576000000	-0.705522000000	H	4.445327000000	-1.950400000000	1.308653000000
H	-7.692807000000	-0.106187000000	1.259083000000	H	3.811381000000	-0.727009000000	0.200119000000
C	-6.698212000000	0.441214000000	-2.437518000000	H	-0.306286000000	-2.147515000000	1.541137000000
H	-4.714937000000	-0.131648000000	-1.827224000000	H	0.360402000000	-3.775939000000	1.549695000000
C	1.668779000000	-4.989281000000	-0.813654000000	H	-0.006400000000	-2.974517000000	3.076446000000
H	3.308446000000	-3.573501000000	-0.848812000000	H	3.668951000000	-2.809156000000	3.209364000000
C	-0.444779000000	-4.154997000000	-1.639815000000	H	2.161642000000	-3.145468000000	4.083265000000
H	-0.437834000000	-2.120494000000	-2.357153000000	H	2.653396000000	-4.173971000000	2.745203000000
H	5.580228000000	0.387674000000	-2.949301000000	H	-0.014810000000	1.208426000000	2.154991000000
H	4.701004000000	0.315187000000	-4.481495000000	H	0.091782000000	1.598993000000	3.861605000000
H	3.460803000000	2.313191000000	-4.014690000000	H	1.512576000000	1.852889000000	2.866008000000

H	2.199578000000	-1.234146000000	4.976674000000
H	2.549343000000	0.496490000000	4.814177000000
H	1.012669000000	-0.034949000000	5.489585000000

INTb4

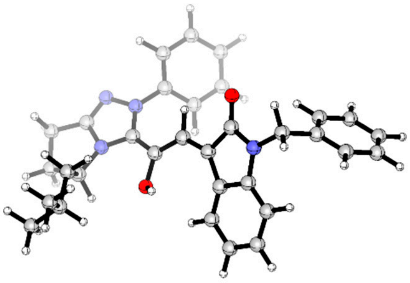

Zero-point correction= 0.586868 (Hartree/Particle)

Thermal correction to Energy= 0.619334

Thermal correction to Enthalpy= 0.620279

Thermal correction to Gibbs Free Energy= 0.520895

Sum of electronic and zero-point Energies= -1606.914849

Sum of electronic and thermal Energies= -1606.882383

Sum of electronic and thermal Enthalpies= -1606.881439

Sum of electronic and thermal Free Energies= -1606.980822

E(RM062X) = -1607.88431861

C	1.769644000000	-3.585772000000	-1.875739000000
C	3.037593000000	-3.821536000000	-1.352142000000
C	3.564486000000	-2.990843000000	-0.360332000000
C	2.776792000000	-1.948190000000	0.097410000000
C	1.471454000000	-1.700744000000	-0.398865000000
C	0.987638000000	-2.522325000000	-1.417056000000
N	3.103625000000	-0.988692000000	1.050347000000
C	2.088766000000	-0.044827000000	1.165394000000
C	0.989003000000	-0.502359000000	0.287379000000
O	2.150856000000	0.948824000000	1.876170000000
C	-0.160846000000	0.244057000000	0.288755000000
C	-1.421487000000	-0.062437000000	-0.239329000000
C	-2.498014000000	0.814343000000	-0.227492000000
O	-1.727031000000	-1.335281000000	-0.717839000000
N	-2.555111000000	2.176622000000	-0.017955000000
N	-3.868372000000	2.638621000000	0.052936000000
C	-4.573614000000	1.583118000000	-0.170922000000
N	-3.812508000000	0.469664000000	-0.395237000000
C	-6.024363000000	1.221391000000	-0.181238000000
C	-5.986326000000	-0.197637000000	-0.805367000000
C	-4.576078000000	-0.774131000000	-0.497374000000
C	-1.514903000000	3.140745000000	-0.133129000000
C	-4.477702000000	-1.598984000000	0.793309000000
C	-4.593615000000	-3.107320000000	0.554150000000
C	-1.529363000000	4.246702000000	0.712216000000
C	-0.533919000000	5.210678000000	0.586255000000
C	0.469796000000	5.067086000000	-0.366835000000
C	0.467870000000	3.959271000000	-1.212090000000
C	-0.528225000000	2.997673000000	-1.109022000000
C	-4.421987000000	-3.865284000000	1.869357000000
C	-5.909385000000	-3.495764000000	-0.121169000000
C	5.453262000000	-0.285477000000	0.821456000000
C	6.625165000000	-0.987803000000	0.553780000000
C	7.599250000000	-0.445489000000	-0.283273000000
C	7.401656000000	0.805073000000	-0.858197000000
C	6.230336000000	1.513918000000	-0.591337000000
C	5.260974000000	0.973084000000	0.244197000000
C	4.377057000000	-0.854651000000	1.721403000000
H	1.386451000000	-4.225029000000	-2.664367000000
H	3.634791000000	-4.645390000000	-1.729342000000
H	4.568808000000	-3.139248000000	0.022689000000
H	0.021322000000	-2.329375000000	-1.869799000000
H	-0.081902000000	1.166550000000	0.862925000000
H	-1.227554000000	-1.964114000000	-0.178378000000

H -6.400520000000 1.203237000000 0.846319000000

H -6.631451000000 1.925420000000 -0.750978000000

H -6.116368000000 -0.121033000000 -1.888117000000

H -6.774407000000 -0.842038000000 -0.413767000000

H -4.183093000000 -1.357229000000 -1.333513000000

H -3.507842000000 -1.393240000000 1.259421000000

H -5.250134000000 -1.268718000000 1.503826000000

H -3.767545000000 -3.382833000000 -0.117077000000

H -2.318757000000 4.341246000000 1.449229000000

H -0.539967000000 6.072380000000 1.245287000000

H 1.251286000000 5.814433000000 -0.451816000000

H 1.242693000000 3.842631000000 -1.962217000000

H -0.532990000000 2.131091000000 -1.761073000000

H -4.443590000000 -4.946295000000 1.705577000000

H -3.474111000000 -3.613979000000 2.354232000000

H -5.231775000000 -3.616009000000 2.564378000000

H -5.966810000000 -4.579069000000 -0.260377000000

H -6.023787000000 -3.032800000000 -1.106737000000

H -6.763804000000 -3.195078000000 0.497540000000

H 6.778382000000 -1.966497000000 1.003577000000

H 8.507864000000 -1.002560000000 -0.489068000000

H 8.156543000000 1.228345000000 -1.513256000000

H 6.076107000000 2.492582000000 -1.035167000000

H 4.347100000000 1.519123000000 0.467433000000

H 4.681815000000 -1.830575000000 2.116263000000

H 4.193692000000 -0.181195000000 2.564154000000

----

^{rr}INTb5

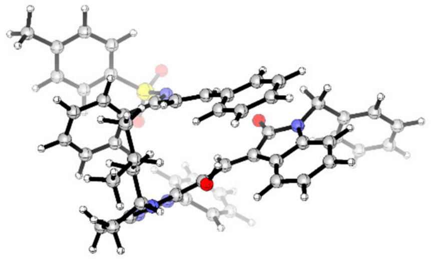

Zero-point correction= 0.946534 (Hartree/Particle)

Thermal correction to Energy= 1.003447

Thermal correction to Enthalpy= 1.004391

Thermal correction to Gibbs Free Energy= 0.849249

Sum of electronic and zero-point Energies= -3059.226823

Sum of electronic and thermal Energies= -3059.169910

Sum of electronic and thermal Enthalpies= -3059.168966

Sum of electronic and thermal Free Energies= -3059.324109

E(RM062X) = -3060.83875353

C 2.647938000000 -0.448222000000 -0.347277000000

C 0.328113000000 0.133854000000 1.959238000000

C 1.441426000000 -0.389847000000 -1.007397000000

C 0.597772000000 -1.446229000000 -1.364127000000

O 0.787391000000 -2.732459000000 -0.861731000000

H 1.190942000000 -2.632227000000 0.013738000000

C 3.613286000000 -1.496403000000 -0.024403000000

C 4.668482000000 -0.868750000000 0.687341000000

C 5.779467000000 -1.556921000000 1.144986000000

C 5.845272000000 -2.930036000000 0.894079000000

C 3.733215000000 -2.857894000000 -0.304686000000

C 4.837705000000 -3.567552000000 0.175194000000

N 4.385585000000 0.482643000000 0.832910000000

C 3.178115000000 0.803749000000 0.219845000000

O 2.687138000000 1.927524000000 0.231274000000

C -0.956551000000 -0.119982000000 1.658587000000

C -1.858163000000 0.853946000000 1.035570000000

N	-1.479223000000	2.082945000000	0.947973000000	H	4.918015000000	-4.628963000000	-0.036321000000
S	-2.480749000000	3.332420000000	0.407508000000	H	0.730686000000	1.126288000000	1.749086000000
O	-1.906473000000	4.531858000000	0.982934000000	H	-3.219152000000	3.285438000000	3.170348000000
O	-2.682228000000	3.235893000000	-1.036948000000	H	-5.374048000000	2.753087000000	4.297850000000
C	-4.049576000000	3.042697000000	1.201659000000	H	-7.259485000000	2.281221000000	0.474138000000
C	-4.107648000000	3.052980000000	2.590935000000	H	-5.089773000000	2.787679000000	-0.653300000000
C	-5.315312000000	2.755894000000	3.213247000000	H	-8.580029000000	2.720312000000	2.745713000000
C	-6.457938000000	2.465030000000	2.462804000000	H	-8.015576000000	1.062774000000	2.951105000000
C	-6.374310000000	2.496276000000	1.066967000000	H	-7.704517000000	2.261129000000	4.214230000000
C	-5.174879000000	2.782852000000	0.428063000000	H	7.831217000000	1.307062000000	2.237661000000
C	-7.759267000000	2.111360000000	3.134256000000	H	9.767353000000	2.120408000000	0.918999000000
C	5.174259000000	1.443090000000	1.566579000000	H	9.399505000000	3.209868000000	-1.278196000000
C	6.375497000000	1.932619000000	0.787042000000	H	7.092389000000	3.485632000000	-2.149234000000
C	7.671370000000	1.784156000000	1.273156000000	H	5.155108000000	2.667631000000	-0.817250000000
C	8.760303000000	2.243369000000	0.533188000000	H	5.489513000000	0.997196000000	2.518778000000
C	8.553729000000	2.853097000000	-0.699149000000	H	4.498741000000	2.278615000000	1.778661000000
C	7.256472000000	3.005808000000	-1.189546000000	H	0.096168000000	-2.594961000000	1.930606000000
C	6.172351000000	2.548611000000	-0.450861000000	H	1.744566000000	-4.224897000000	2.770726000000
C	1.250347000000	-0.862313000000	2.513059000000	H	3.859116000000	-3.439258000000	3.809918000000
C	1.005098000000	-2.241870000000	2.414358000000	H	4.288865000000	-1.010269000000	4.041637000000
C	1.935145000000	-3.161883000000	2.880483000000	H	2.641489000000	0.632733000000	3.176698000000
C	3.120605000000	-2.721544000000	3.469526000000	H	-3.788570000000	-0.381075000000	2.491640000000
C	3.363010000000	-1.356868000000	3.593032000000	H	-5.925371000000	-1.325201000000	1.664627000000
C	2.439550000000	-0.433607000000	3.112302000000	H	-6.480947000000	-1.212973000000	-0.753345000000
C	-3.146459000000	0.274257000000	0.541865000000	H	-4.867468000000	-0.169688000000	-2.345192000000
C	-4.028749000000	-0.344847000000	1.432307000000	H	-2.748979000000	0.780031000000	-1.509024000000
C	-5.229178000000	-0.871229000000	0.966227000000	C	-0.508963000000	-1.337640000000	-2.202735000000
C	-5.539776000000	-0.809100000000	-0.392936000000	N	-0.911930000000	-0.335167000000	-3.052432000000
C	-4.641113000000	-0.227264000000	-1.284515000000	N	-2.111491000000	-0.633390000000	-3.689655000000
C	-3.442928000000	0.310666000000	-0.821463000000	C	-2.397028000000	-1.816070000000	-3.256003000000
H	6.703206000000	-3.495824000000	1.243012000000	N	-1.457865000000	-2.305227000000	-2.391355000000
H	6.579389000000	-1.039508000000	1.665433000000	C	-3.530831000000	-2.782974000000	-3.384896000000
H	2.981072000000	-3.366959000000	-0.895968000000	C	-2.957889000000	-4.038281000000	-2.677520000000

C	-1.866071000000	-3.528840000000	-1.697218000000
C	-2.354535000000	-3.187097000000	-0.284982000000
C	-2.248196000000	-4.341494000000	0.716314000000
C	-0.177231000000	0.827020000000	-3.436180000000
C	1.102866000000	0.680253000000	-3.968449000000
C	1.831465000000	1.816809000000	-4.294259000000
C	1.278435000000	3.082916000000	-4.104119000000
C	-0.008208000000	3.212819000000	-3.590932000000
C	-0.745480000000	2.081503000000	-3.252548000000
C	-2.761237000000	-3.892133000000	2.084632000000
C	-2.989310000000	-5.599856000000	0.262446000000
H	-4.404148000000	-2.392782000000	-2.854439000000
H	-3.812488000000	-2.965464000000	-4.422510000000
H	-2.496879000000	-4.697874000000	-3.417253000000
H	-3.733503000000	-4.600416000000	-2.157294000000
H	-1.013321000000	-4.208075000000	-1.642561000000
H	-3.389172000000	-2.812075000000	-0.332234000000
H	-1.738600000000	-2.355397000000	0.078190000000
H	-1.179653000000	-4.590007000000	0.805712000000
H	1.524371000000	-0.312266000000	-4.090538000000
H	2.834384000000	1.713213000000	-4.694225000000
H	1.856561000000	3.967592000000	-4.348858000000
H	-0.440944000000	4.193020000000	-3.423513000000
H	-1.736611000000	2.183694000000	-2.826494000000
H	-2.671470000000	-4.693364000000	2.823727000000
H	-2.207175000000	-3.026434000000	2.461977000000
H	-3.817285000000	-3.605941000000	2.018516000000
H	-2.909415000000	-6.387198000000	1.017374000000
H	-2.589072000000	-5.999996000000	-0.674157000000
H	-4.055242000000	-5.387176000000	0.115442000000
H	-1.389035000000	-1.097649000000	1.846587000000
H	1.078278000000	0.622123000000	-1.185158000000

----

^{rs}INTb5

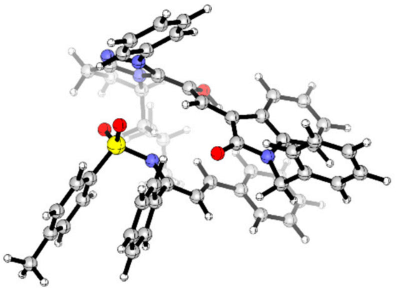

Zero-point correction=	0.946675 (Hartree/Particle)
Thermal correction to Energy=	1.003226
Thermal correction to Enthalpy=	1.004170
Thermal correction to Gibbs Free Energy=	0.852330
Sum of electronic and zero-point Energies=	-3059.240368
Sum of electronic and thermal Energies=	-3059.183817
Sum of electronic and thermal Enthalpies=	-3059.182873
Sum of electronic and thermal Free Energies=	-3059.334713

E(RM062X) = -3060.85268824

C	-1.834284000000	-0.816150000000	0.868292000000
C	-0.891083000000	-0.323185000000	-2.102671000000
C	-0.563595000000	-1.067475000000	1.387892000000
C	0.152627000000	-2.237489000000	1.270009000000
O	-0.256135000000	-3.289329000000	0.460615000000
H	-0.932333000000	-2.933266000000	-0.134891000000
C	-2.946229000000	-1.660156000000	0.460760000000
C	-3.931385000000	-0.795466000000	-0.089053000000
C	-5.146827000000	-1.254811000000	-0.566201000000
C	-5.402262000000	-2.626766000000	-0.493773000000
C	-3.247769000000	-3.022255000000	0.559432000000
C	-4.467195000000	-3.494186000000	0.067243000000
N	-3.457757000000	0.507665000000	-0.043310000000
C	-2.213262000000	0.558128000000	0.583240000000
O	-1.588368000000	1.608990000000	0.761494000000

C	-0.530909000000	0.963030000000	-1.935448000000
C	0.633387000000	1.334933000000	-1.132844000000
N	1.379080000000	0.379744000000	-0.672360000000
S	2.784712000000	0.628584000000	0.194916000000
O	2.476998000000	1.121811000000	1.535066000000
O	3.518870000000	-0.626918000000	0.054501000000
C	3.718280000000	1.867096000000	-0.674717000000
C	3.975183000000	3.088557000000	-0.064760000000
C	4.652311000000	4.064014000000	-0.785292000000
C	5.066951000000	3.831949000000	-2.100172000000
C	4.814006000000	2.584087000000	-2.677620000000
C	4.143815000000	1.593032000000	-1.970184000000
C	5.748572000000	4.923511000000	-2.882737000000
C	-4.147680000000	1.682569000000	-0.519995000000
C	-5.184828000000	2.194695000000	0.456001000000
C	-6.529294000000	2.286520000000	0.106614000000
C	-7.467520000000	2.757204000000	1.024274000000
C	-7.060918000000	3.136791000000	2.298613000000
C	-5.714463000000	3.047150000000	2.653182000000
C	-4.780099000000	2.579355000000	1.737459000000
C	-2.082182000000	-0.822113000000	-2.784617000000
C	-3.024914000000	0.023098000000	-3.388691000000
C	-4.172081000000	-0.501223000000	-3.967357000000
C	-4.401357000000	-1.877756000000	-3.952294000000
C	-3.471954000000	-2.728946000000	-3.363123000000
C	-2.321082000000	-2.202272000000	-2.789407000000
C	0.867162000000	2.799053000000	-0.954329000000
C	0.737883000000	3.394835000000	0.302085000000
C	0.972553000000	4.760706000000	0.433715000000
C	1.347368000000	5.523659000000	-0.669621000000
C	1.468014000000	4.926269000000	-1.922822000000
C	1.210519000000	3.567564000000	-2.069736000000
H	-6.347000000000	-3.013417000000	-0.862419000000
H	-5.882088000000	-0.566254000000	-0.970908000000
H	-2.548156000000	-3.710794000000	1.021853000000
H	-4.693825000000	-4.553161000000	0.141147000000
H	-0.245830000000	-1.076936000000	-1.650622000000
H	3.622535000000	3.265020000000	0.945548000000
H	4.847685000000	5.029327000000	-0.326210000000
H	5.146282000000	2.388764000000	-3.693175000000
H	3.944626000000	0.621818000000	-2.413652000000
H	6.510007000000	5.425086000000	-2.280060000000
H	6.226107000000	4.531223000000	-3.782837000000
H	5.022319000000	5.682877000000	-3.191203000000
H	-6.845469000000	1.987143000000	-0.890278000000
H	-8.514165000000	2.821785000000	0.743812000000
H	-7.789409000000	3.500940000000	3.016084000000
H	-5.394409000000	3.345030000000	3.646737000000
H	-3.726114000000	2.508373000000	1.996580000000
H	-4.609587000000	1.451362000000	-1.487350000000
H	-3.370903000000	2.439944000000	-0.672946000000
H	-2.862201000000	1.096711000000	-3.396985000000
H	-4.894933000000	0.164559000000	-4.428577000000
H	-5.306534000000	-2.282451000000	-4.393339000000
H	-3.649571000000	-3.798539000000	-3.334089000000
H	-1.590581000000	-2.865953000000	-2.330773000000
H	0.431694000000	2.786321000000	1.144531000000
H	0.859755000000	5.229931000000	1.405704000000
H	1.536709000000	6.586555000000	-0.555871000000
H	1.756361000000	5.517933000000	-2.786039000000
H	1.302905000000	3.092624000000	-3.042933000000
C	1.481046000000	-2.372568000000	1.722967000000
N	2.140459000000	-1.759507000000	2.745364000000
N	3.489366000000	-2.063310000000	2.746467000000
C	3.613824000000	-2.889867000000	1.760512000000
N	2.423970000000	-3.157112000000	1.149231000000

C 4.730441000000 -3.524083000000 1.000341000000

C 3.962781000000 -4.516479000000 0.089244000000

C 2.536738000000 -3.926423000000 -0.094540000000

C 2.345908000000 -3.037804000000 -1.331898000000

C 1.692700000000 -3.779312000000 -2.500776000000

C 1.616348000000 -1.006527000000 3.837436000000

C 2.365307000000 0.061708000000 4.320351000000

C 1.894917000000 0.771833000000 5.419293000000

C 0.689190000000 0.423946000000 6.021758000000

C -0.046955000000 -0.651192000000 5.528414000000

C 0.416285000000 -1.379925000000 4.439952000000

C 1.507094000000 -2.831053000000 -3.684928000000

C 2.484802000000 -5.016145000000 -2.928148000000

H 5.217814000000 -2.734945000000 0.418930000000

H 5.473872000000 -4.005164000000 1.635977000000

H 3.893556000000 -5.488388000000 0.585632000000

H 4.456706000000 -4.660512000000 -0.872550000000

H 1.770630000000 -4.705354000000 -0.082283000000

H 3.314716000000 -2.620104000000 -1.637485000000

H 1.730395000000 -2.174533000000 -1.056136000000

H 0.700941000000 -4.109154000000 -2.155276000000

H 3.287405000000 0.325348000000 3.818251000000

H 2.471811000000 1.608839000000 5.798184000000

H 0.322259000000 0.988294000000 6.872542000000

H -0.984722000000 -0.932162000000 5.995565000000

H -0.150960000000 -2.217298000000 4.049724000000

H 0.959429000000 -3.314607000000 -4.499239000000

H 0.960400000000 -1.928244000000 -3.395621000000

H 2.482408000000 -2.517490000000 -4.074927000000

H 2.001456000000 -5.517319000000 -3.771825000000

H 2.579051000000 -5.748247000000 -2.119346000000

H 3.495377000000 -4.730488000000 -3.244953000000

H -1.132481000000 1.786949000000 -2.305612000000

H -0.041384000000 -0.206088000000 1.805878000000

----

^{sr}INTb5

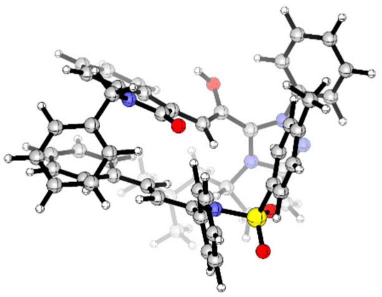

Zero-point correction= 0.948023 (Hartree/Particle)

Thermal correction to Energy= 1.004129

Thermal correction to Enthalpy= 1.005073

Thermal correction to Gibbs Free Energy= 0.855381

Sum of electronic and zero-point Energies= -3059.232759

Sum of electronic and thermal Energies= -3059.176653

Sum of electronic and thermal Enthalpies= -3059.175709

Sum of electronic and thermal Free Energies= -3059.325401

E(RM062X) = -3060.84736645

C 0.822333000000 -1.097845000000 1.428454000000

C 1.883918000000 -0.199875000000 -1.375747000000

C -0.445624000000 -1.134800000000 0.804208000000

C -1.386739000000 -2.109779000000 0.948430000000

O -1.259221000000 -3.179621000000 1.817441000000

H -0.571600000000 -2.930567000000 2.456079000000

C 1.741394000000 -2.133091000000 1.841942000000

C 2.913988000000 -1.472635000000 2.313115000000

C 4.040291000000 -2.166555000000 2.722584000000

C 4.012402000000 -3.563141000000 2.673476000000

C 1.757419000000 -3.532268000000 1.768667000000

C 2.885061000000 -4.233233000000 2.200851000000

N 2.709364000000 -0.103138000000 2.237333000000

C	1.448842000000	0.173977000000	1.698609000000
O	1.017597000000	1.325161000000	1.548671000000
C	3.548227000000	0.935505000000	2.797910000000
C	4.438596000000	1.624661000000	1.785245000000
C	5.779836000000	1.276934000000	1.636110000000
C	6.576497000000	1.917960000000	0.688520000000
C	6.035454000000	2.913987000000	-0.120843000000
C	4.695759000000	3.273747000000	0.028702000000
C	3.905485000000	2.633288000000	0.978280000000
H	4.883563000000	-4.125817000000	2.992725000000
H	4.930875000000	-1.640253000000	3.052412000000
H	0.903802000000	-4.075559000000	1.373026000000
H	2.885619000000	-5.318023000000	2.153968000000
H	6.204479000000	0.494576000000	2.260780000000
H	7.620971000000	1.639748000000	0.583481000000
H	6.656015000000	3.413172000000	-0.858301000000
H	4.262993000000	4.054153000000	-0.591305000000
H	2.857447000000	2.889846000000	1.096183000000
H	4.149890000000	0.492814000000	3.597808000000
H	2.873998000000	1.674822000000	3.242003000000
C	-2.611319000000	-2.087702000000	0.179394000000
N	-3.906029000000	-1.964813000000	0.578808000000
N	-4.769620000000	-1.933144000000	-0.482961000000
C	-3.994206000000	-2.018390000000	-1.521754000000
N	-2.688894000000	-2.129206000000	-1.163254000000
C	-4.168922000000	-2.028977000000	-2.998089000000
C	-2.720091000000	-2.269851000000	-3.507758000000
C	-1.741684000000	-2.261283000000	-2.290389000000
C	-0.882890000000	-3.516524000000	-2.124250000000
C	0.341596000000	-3.586910000000	-3.046911000000
C	-4.399430000000	-1.561453000000	1.859160000000
C	-3.775839000000	-1.976829000000	3.031198000000
C	-4.248658000000	-1.490398000000	4.247627000000
C	-5.333664000000	-0.620640000000	4.292589000000
C	-5.958098000000	-0.231787000000	3.108546000000
C	-5.492488000000	-0.693180000000	1.883877000000
C	1.175263000000	-4.816527000000	-2.680147000000
C	-0.000475000000	-3.608577000000	-4.537511000000
H	-4.864607000000	-2.810427000000	-3.308898000000
H	-4.550141000000	-1.059108000000	-3.319791000000
H	-2.437405000000	-1.486999000000	-4.210683000000
H	-2.655559000000	-3.233745000000	-4.016323000000
H	-1.128673000000	-1.353819000000	-2.299124000000
H	-1.513120000000	-4.405411000000	-2.272155000000
H	-0.524425000000	-3.548634000000	-1.087472000000
H	0.951110000000	-2.692448000000	-2.860534000000
H	-2.950289000000	-2.675533000000	2.988340000000
H	-3.764890000000	-1.806095000000	5.165660000000
H	-5.695465000000	-0.250475000000	5.245949000000
H	-6.804703000000	0.445960000000	3.133135000000
H	-5.950119000000	-0.383280000000	0.952705000000
H	2.120396000000	-4.825060000000	-3.230235000000
H	1.406667000000	-4.844772000000	-1.610025000000
H	0.629448000000	-5.734214000000	-2.927416000000
H	0.910197000000	-3.737085000000	-5.129298000000
H	-0.477802000000	-2.682554000000	-4.867880000000
H	-0.670166000000	-4.444559000000	-4.772510000000
H	-0.718886000000	-0.248675000000	0.231172000000
C	1.735269000000	1.140193000000	-1.412580000000
C	0.451771000000	1.785011000000	-1.661070000000
N	-0.566025000000	1.049218000000	-2.005475000000
C	0.448311000000	3.273388000000	-1.505811000000
C	0.285428000000	4.107304000000	-2.614862000000
C	0.324879000000	5.488917000000	-2.449045000000
C	0.518881000000	6.037816000000	-1.183130000000
C	0.683962000000	5.202975000000	-0.079927000000

C	0.659805000000	3.820046000000	-0.235727000000
S	-2.112815000000	1.663661000000	-2.206164000000
C	-2.566662000000	2.366107000000	-0.638741000000
C	-2.930623000000	1.505566000000	0.394195000000
C	-3.227495000000	2.030633000000	1.642907000000
C	-3.181853000000	3.411589000000	1.863055000000
C	-2.845316000000	4.254686000000	0.801126000000
C	-2.529172000000	3.742898000000	-0.453328000000
C	-3.468099000000	3.968977000000	3.232291000000
O	-2.931316000000	0.461739000000	-2.383406000000
O	-2.168929000000	2.699010000000	-3.230433000000
C	3.117791000000	-0.939604000000	-1.130266000000
C	3.032232000000	-2.330468000000	-0.975898000000
C	4.164887000000	-3.103068000000	-0.759203000000
C	5.410895000000	-2.487905000000	-0.684282000000
C	5.511314000000	-1.103057000000	-0.819938000000
C	4.378588000000	-0.330416000000	-1.038688000000
H	2.567482000000	1.804856000000	-1.206257000000
H	0.994142000000	-0.806935000000	-1.535073000000
H	0.110361000000	3.675068000000	-3.593204000000
H	0.199314000000	6.135752000000	-3.311054000000
H	0.542894000000	7.115935000000	-1.058309000000
H	0.827906000000	5.627181000000	0.908797000000
H	0.754675000000	3.149726000000	0.614263000000
H	-2.991499000000	0.438315000000	0.210392000000
H	-3.499907000000	1.360675000000	2.454506000000
H	-2.816736000000	5.329092000000	0.959167000000
H	-2.247216000000	4.392950000000	-1.276611000000
H	-4.221075000000	3.370016000000	3.750657000000
H	-2.560516000000	3.958708000000	3.844388000000
H	-3.822356000000	5.000639000000	3.176994000000
H	2.051079000000	-2.795219000000	-0.990170000000
H	4.068565000000	-4.174685000000	-0.619215000000
H	6.301788000000	-3.082695000000	-0.508697000000
H	6.478580000000	-0.616095000000	-0.749172000000
H	4.479127000000	0.747043000000	-1.136641000000

----

^{SS}INTb5

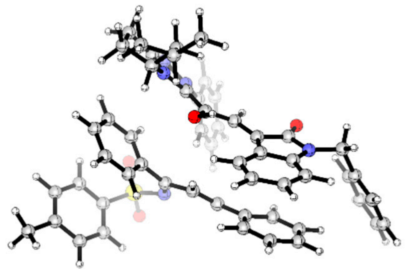

Zero-point correction= 0.947626 (Hartree/Particle)

Thermal correction to Energy= 1.004062

Thermal correction to Enthalpy= 1.005006

Thermal correction to Gibbs Free Energy= 0.852607

Sum of electronic and zero-point Energies= -3059.219513

Sum of electronic and thermal Energies= -3059.163078

Sum of electronic and thermal Enthalpies= -3059.162133

Sum of electronic and thermal Free Energies= -3059.314533

E(RM062X) = -3060.83236744

C	0.068953000000	-1.476660000000	-0.905270000000
C	1.382883000000	-1.774338000000	-0.315078000000
C	-1.038535000000	-2.143867000000	-0.528181000000
N	1.472226000000	-2.820112000000	0.439250000000
C	2.488159000000	-0.828040000000	-0.656470000000
C	3.224152000000	-0.199281000000	0.352935000000
C	4.260612000000	0.665195000000	0.012964000000
C	4.571930000000	0.902167000000	-1.324134000000
C	3.828971000000	0.286180000000	-2.330222000000
C	2.781785000000	-0.565475000000	-1.998130000000
C	4.012701000000	-3.439644000000	-0.359121000000

C	3.657176000000	-4.161146000000	-1.496168000000
C	4.476189000000	-4.095180000000	-2.614875000000
C	5.649041000000	-3.330986000000	-2.604204000000
C	5.990342000000	-2.638139000000	-1.441016000000
C	5.178620000000	-2.688167000000	-0.312070000000
C	6.538959000000	-3.284965000000	-3.818861000000
S	2.910677000000	-3.459067000000	1.040602000000
O	2.585031000000	-4.842767000000	1.331103000000
O	3.467566000000	-2.607441000000	2.086242000000
C	-2.357107000000	-2.049116000000	-1.151180000000
C	-3.502378000000	-2.327966000000	-0.394594000000
C	-4.761456000000	-2.292515000000	-0.981283000000
C	-4.887716000000	-1.993615000000	-2.335368000000
C	-3.754229000000	-1.732707000000	-3.103454000000
C	-2.497862000000	-1.756288000000	-2.515336000000
H	0.024205000000	-0.679025000000	-1.642956000000
H	-0.938381000000	-2.844749000000	0.300537000000
H	3.006189000000	-0.411483000000	1.395467000000
H	4.836849000000	1.137253000000	0.804867000000
H	5.396652000000	1.559703000000	-1.585004000000
H	4.069315000000	0.464867000000	-3.373187000000
H	2.208165000000	-1.056610000000	-2.779029000000
H	2.748989000000	-4.756356000000	-1.498701000000
H	4.206208000000	-4.645985000000	-3.511755000000
H	6.898659000000	-2.042193000000	-1.421730000000
H	5.420684000000	-2.138417000000	0.591244000000
H	5.950918000000	-3.283651000000	-4.740005000000
H	7.172089000000	-2.394910000000	-3.812848000000
H	7.195801000000	-4.160325000000	-3.849443000000
H	-3.400854000000	-2.557995000000	0.662986000000
H	-5.643214000000	-2.505342000000	-0.387256000000
H	-5.873953000000	-1.968516000000	-2.789920000000
H	-3.850367000000	-1.500078000000	-4.158945000000
H	-1.612034000000	-1.567334000000	-3.114395000000
C	-2.633758000000	1.533223000000	-3.688211000000
C	-4.024715000000	1.562375000000	-3.701593000000
C	-4.746835000000	1.438350000000	-2.512688000000
C	-4.041839000000	1.318188000000	-1.326219000000
C	-2.620489000000	1.328367000000	-1.276418000000
C	-1.929430000000	1.404276000000	-2.488085000000
N	-4.549731000000	1.176828000000	-0.041092000000
C	-3.518553000000	1.076681000000	0.886579000000
C	-2.255018000000	1.199903000000	0.134756000000
O	-3.698039000000	0.966908000000	2.093668000000
C	-1.108603000000	1.308764000000	0.889727000000
C	0.166416000000	1.725114000000	0.509768000000
C	1.208758000000	1.999298000000	1.400416000000
O	0.430578000000	2.142379000000	-0.793967000000
N	1.458309000000	1.592494000000	2.690085000000
N	2.586191000000	2.209880000000	3.220466000000
C	3.018671000000	2.943341000000	2.251603000000
N	2.255210000000	2.835242000000	1.127745000000
C	4.110267000000	3.942987000000	2.045815000000
C	4.071608000000	4.140237000000	0.507384000000
C	2.650196000000	3.723125000000	0.033442000000
C	0.907919000000	0.503377000000	3.429225000000
C	1.654508000000	4.879960000000	-0.129463000000
C	1.540726000000	5.372306000000	-1.575099000000
C	0.877793000000	0.594520000000	4.818584000000
C	0.431577000000	-0.495566000000	5.556917000000
C	0.027407000000	-1.665577000000	4.918870000000
C	0.063415000000	-1.742509000000	3.529306000000
C	0.497597000000	-0.657429000000	2.779217000000
C	0.474858000000	6.461300000000	-1.681468000000
C	2.878067000000	5.869971000000	-2.125888000000
C	-6.654299000000	-0.006428000000	0.556889000000

C	-7.610310000000	-0.449924000000	-0.354804000000
C	-8.263909000000	-1.666448000000	-0.159482000000
C	-7.965127000000	-2.444735000000	0.955680000000
C	-7.008353000000	-2.005195000000	1.871053000000
C	-6.354488000000	-0.793634000000	1.673317000000
C	-5.933501000000	1.310925000000	0.369733000000
H	-2.083535000000	1.602028000000	-4.621358000000
H	-4.556832000000	1.659615000000	-4.642594000000
H	-5.831969000000	1.412775000000	-2.520237000000
H	-0.846111000000	1.347024000000	-2.514360000000
H	-1.275300000000	1.144258000000	1.953934000000
H	-0.343164000000	2.642853000000	-1.090216000000
H	3.865176000000	4.864640000000	2.582126000000
H	5.075828000000	3.589667000000	2.410286000000
H	4.811665000000	3.488600000000	0.035797000000
H	4.297720000000	5.168373000000	0.221172000000
H	2.691394000000	3.134542000000	-0.886687000000
H	0.669263000000	4.538332000000	0.206105000000
H	1.940665000000	5.713055000000	0.529428000000
H	1.221865000000	4.509826000000	-2.178360000000
H	1.222319000000	1.502085000000	5.300242000000
H	0.407042000000	-0.429646000000	6.639550000000
H	-0.307041000000	-2.516448000000	5.502334000000
H	-0.223035000000	-2.654579000000	3.015219000000
H	0.506696000000	-0.707488000000	1.698597000000
H	0.349227000000	6.789601000000	-2.717037000000
H	-0.494204000000	6.107189000000	-1.317754000000
H	0.758583000000	7.335789000000	-1.085173000000
H	2.763491000000	6.231689000000	-3.151622000000
H	3.639577000000	5.082942000000	-2.137505000000
H	3.257561000000	6.702269000000	-1.520505000000
H	-7.850520000000	0.160431000000	-1.222626000000
H	-9.007601000000	-2.003557000000	-0.874991000000
H	-8.474478000000	-3.390504000000	1.111340000000
H	-6.772288000000	-2.609034000000	2.741788000000
H	-5.597066000000	-0.442298000000	2.369105000000
H	-6.450556000000	1.932068000000	-0.368366000000
H	-5.918113000000	1.849628000000	1.322785000000

----

^{SS}TSb3

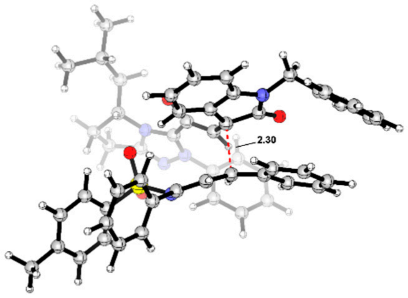

Frequency -247.1961

Zero-point correction= 0.947054 (Hartree/Particle)

Thermal correction to Energy= 1.002478

Thermal correction to Enthalpy= 1.003423

Thermal correction to Gibbs Free Energy= 0.854966

Sum of electronic and zero-point Energies= -3059.210684

Sum of electronic and thermal Energies= -3059.155259

Sum of electronic and thermal Enthalpies= -3059.154315

Sum of electronic and thermal Free Energies= -3059.302772

E(RM062X) = -3060.82528433

C	-1.729508000000	-0.799945000000	0.501481000000
C	-1.698682000000	1.024516000000	-0.892221000000
C	-0.780431000000	-1.584754000000	-0.243970000000
C	0.372443000000	-2.117889000000	0.221173000000
O	0.759923000000	-2.170438000000	1.531439000000
H	0.113431000000	-1.691766000000	2.070421000000
C	-1.563487000000	-0.152013000000	1.786824000000
C	-2.851597000000	-0.042241000000	2.374717000000

C	-3.047706000000	0.613121000000	3.576918000000
C	-1.928417000000	1.167668000000	4.213736000000
C	-0.467600000000	0.446451000000	2.410586000000
C	-0.663056000000	1.084980000000	3.644488000000
N	-3.778615000000	-0.672240000000	1.548608000000
C	-3.148677000000	-1.194381000000	0.417179000000
O	-3.717532000000	-1.871938000000	-0.427540000000
C	-5.106592000000	-1.113504000000	1.931274000000
C	-6.254538000000	-0.383129000000	1.270730000000
C	-6.825053000000	0.745626000000	1.859042000000
C	-7.936765000000	1.356844000000	1.285113000000
C	-8.492196000000	0.834479000000	0.119584000000
C	-7.916998000000	-0.283078000000	-0.480652000000
C	-6.797528000000	-0.883495000000	0.086611000000
H	-2.059461000000	1.673367000000	5.164913000000
H	-4.035295000000	0.700565000000	4.018846000000
H	0.521337000000	0.402686000000	1.953225000000
H	0.186780000000	1.527364000000	4.155739000000
H	-6.403538000000	1.141278000000	2.780642000000
H	-8.378523000000	2.230126000000	1.755421000000
H	-9.369234000000	1.299341000000	-0.320672000000
H	-8.337787000000	-0.684660000000	-1.397699000000
H	-6.321998000000	-1.738684000000	-0.384958000000
H	-5.181188000000	-1.024193000000	3.019403000000
H	-5.173955000000	-2.175862000000	1.673431000000
C	1.407737000000	-2.656538000000	-0.633957000000
N	1.414510000000	-2.922718000000	-1.956082000000
N	2.643400000000	-3.332033000000	-2.401097000000
C	3.366874000000	-3.330839000000	-1.325731000000
N	2.651853000000	-2.955129000000	-0.227091000000
C	4.795868000000	-3.560753000000	-0.964237000000
C	4.874552000000	-2.789216000000	0.377655000000
C	3.458361000000	-2.855059000000	1.006187000000
C	3.192037000000	-4.053997000000	1.922156000000
C	3.421121000000	-3.739287000000	3.404052000000
C	0.386832000000	-2.618371000000	-2.907754000000
C	-0.598340000000	-3.561096000000	-3.170340000000
C	-1.635773000000	-3.218998000000	-4.032196000000
C	-1.665549000000	-1.953569000000	-4.614293000000
C	-0.658963000000	-1.026216000000	-4.345371000000
C	0.385149000000	-1.352159000000	-3.484160000000
C	3.095758000000	-4.966921000000	4.253386000000
C	4.839190000000	-3.247024000000	3.694271000000
H	4.991838000000	-4.630494000000	-0.838840000000
H	5.472944000000	-3.169387000000	-1.723138000000
H	5.106292000000	-1.740861000000	0.168592000000
H	5.632504000000	-3.195440000000	1.048314000000
H	3.190957000000	-1.917630000000	1.492486000000
H	3.817409000000	-4.906786000000	1.616837000000
H	2.146723000000	-4.353940000000	1.797578000000
H	2.717214000000	-2.937089000000	3.665797000000
H	-0.560178000000	-4.530521000000	-2.684972000000
H	-2.424676000000	-3.934154000000	-4.236692000000
H	-2.483348000000	-1.685139000000	-5.275551000000
H	-0.694022000000	-0.038807000000	-4.794026000000
H	1.165267000000	-0.646961000000	-3.210449000000
H	3.196705000000	-4.745823000000	5.319753000000
H	2.074665000000	-5.315697000000	4.073566000000
H	3.780066000000	-5.789805000000	4.016471000000
H	4.971373000000	-3.063623000000	4.764550000000
H	5.068546000000	-2.313845000000	3.170436000000
H	5.578865000000	-3.999487000000	3.393191000000
H	-1.053661000000	-1.770975000000	-1.275025000000
C	-0.890984000000	2.009621000000	-0.295959000000
C	0.492829000000	2.039628000000	-0.424563000000
N	1.148438000000	1.105428000000	-1.145612000000

C	1.197905000000	3.240038000000	0.123519000000
C	1.143511000000	3.564996000000	1.479008000000
C	1.815641000000	4.689085000000	1.955264000000
C	2.520993000000	5.507005000000	1.078139000000
C	2.551325000000	5.203027000000	-0.282884000000
C	1.897103000000	4.073962000000	-0.756721000000
S	2.584886000000	0.536556000000	-0.716598000000
C	3.873371000000	1.776966000000	-0.705214000000
C	4.391208000000	2.225832000000	-1.914519000000
C	5.340894000000	3.241955000000	-1.906836000000
C	5.781395000000	3.804254000000	-0.705756000000
C	5.268419000000	3.308562000000	0.497040000000
C	4.315394000000	2.298913000000	0.506089000000
C	6.768053000000	4.942584000000	-0.696218000000
O	2.985271000000	-0.416748000000	-1.763925000000
O	2.544238000000	0.021783000000	0.668011000000
C	-3.128471000000	1.260424000000	-1.120828000000
C	-3.753355000000	0.627134000000	-2.204130000000
C	-5.072769000000	0.916004000000	-2.527235000000
C	-5.794160000000	1.825792000000	-1.759389000000
C	-5.202103000000	2.417152000000	-0.644464000000
C	-3.881119000000	2.136371000000	-0.323824000000
H	-1.350081000000	2.840837000000	0.226966000000
H	-1.205589000000	0.394163000000	-1.628611000000
H	0.578345000000	2.928596000000	2.152739000000
H	1.782695000000	4.926997000000	3.014021000000
H	3.041835000000	6.383745000000	1.451121000000
H	3.094772000000	5.840928000000	-0.973491000000
H	1.927691000000	3.818034000000	-1.811500000000
H	4.048902000000	1.779583000000	-2.843333000000
H	5.748024000000	3.605416000000	-2.846538000000
H	5.610054000000	3.733191000000	1.437638000000
H	3.894814000000	1.920524000000	1.431572000000
H	7.247088000000	5.064914000000	-1.669979000000
H	6.264317000000	5.883597000000	-0.449410000000
H	7.548330000000	4.784670000000	0.053131000000
H	-3.184263000000	-0.086328000000	-2.794454000000
H	-5.540424000000	0.429292000000	-3.377411000000
H	-6.825349000000	2.053848000000	-2.007896000000
H	-5.778508000000	3.093928000000	-0.022182000000
H	-3.430445000000	2.590516000000	0.553817000000

STTSb3

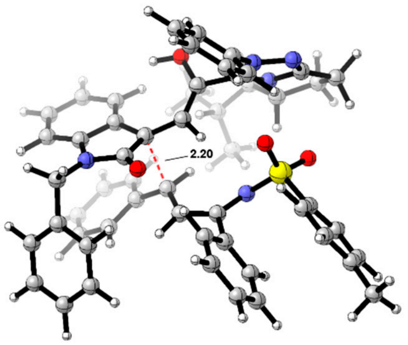

Zero-point correction= 0.948204 (Hartree/Particle)

Thermal correction to Energy= 1.002780

Thermal correction to Enthalpy= 1.003724

Thermal correction to Gibbs Free Energy= 0.859653

Sum of electronic and zero-point Energies= -3059.219780

Sum of electronic and thermal Energies= -3059.165204

Sum of electronic and thermal Enthalpies= -3059.164260

Sum of electronic and thermal Free Energies= -3059.308331

E(RM062X) = -3060.83393468

C	1.793958000000	-0.818569000000	0.714336000000
C	1.553683000000	0.254769000000	-1.193481000000
C	0.448706000000	-1.357455000000	0.781835000000
C	0.055350000000	-2.635163000000	0.840221000000
O	0.820411000000	-3.771099000000	0.959639000000

H	1.716108000000	-3.502615000000	1.213055000000
C	3.085541000000	-1.489731000000	0.611134000000
C	4.061546000000	-0.580738000000	1.103871000000
C	5.418961000000	-0.839122000000	1.010876000000
C	5.823695000000	-2.052565000000	0.446854000000
C	3.522481000000	-2.680567000000	0.028595000000
C	4.888643000000	-2.967343000000	-0.027527000000
N	3.416926000000	0.532704000000	1.630559000000
C	2.037691000000	0.391038000000	1.517979000000
O	1.226518000000	1.170268000000	2.002598000000
C	4.000156000000	1.635753000000	2.375601000000
C	4.091434000000	2.902467000000	1.553091000000
C	5.289698000000	3.288260000000	0.956479000000
C	5.339480000000	4.411988000000	0.132552000000
C	4.185459000000	5.152582000000	-0.106681000000
C	2.985440000000	4.779412000000	0.498008000000
C	2.940158000000	3.665887000000	1.331034000000
H	6.883136000000	-2.274705000000	0.371987000000
H	6.151097000000	-0.116949000000	1.357882000000
H	2.813386000000	-3.373520000000	-0.416634000000
H	5.220356000000	-3.899618000000	-0.473270000000
H	6.187984000000	2.697728000000	1.122703000000
H	6.278637000000	4.703216000000	-0.328675000000
H	4.220711000000	6.020205000000	-0.757770000000
H	2.079129000000	5.349437000000	0.316598000000
H	2.004999000000	3.350772000000	1.784413000000
H	4.986849000000	1.325486000000	2.730700000000
H	3.356275000000	1.803741000000	3.244892000000
C	-1.376364000000	-2.950279000000	0.743748000000
N	-2.294346000000	-2.874978000000	1.712468000000
N	-3.546713000000	-3.160382000000	1.267114000000
C	-3.365480000000	-3.439039000000	0.008214000000
N	-2.050364000000	-3.346184000000	-0.338707000000

C	-4.207516000000	-3.791747000000	-1.171205000000
C	-3.124307000000	-4.245817000000	-2.179741000000
C	-1.806864000000	-3.533278000000	-1.786900000000
C	-0.537810000000	-4.315102000000	-2.115909000000
C	0.698624000000	-3.465535000000	-2.443536000000
C	-2.095570000000	-2.382574000000	3.038261000000
C	-1.046086000000	-2.874583000000	3.804202000000
C	-0.864314000000	-2.360674000000	5.083869000000
C	-1.729708000000	-1.386722000000	5.576273000000
C	-2.775506000000	-0.910998000000	4.788148000000
C	-2.963592000000	-1.401561000000	3.501495000000
C	1.874131000000	-4.397723000000	-2.739190000000
C	0.434514000000	-2.522387000000	-3.621211000000
H	-4.944483000000	-4.563722000000	-0.947497000000
H	-4.707074000000	-2.881714000000	-1.511398000000
H	-3.389746000000	-4.009176000000	-3.210732000000
H	-2.974335000000	-5.328414000000	-2.100160000000
H	-1.796321000000	-2.525931000000	-2.210663000000
H	-0.757074000000	-4.932940000000	-2.998190000000
H	-0.315595000000	-5.007361000000	-1.294020000000
H	0.948386000000	-2.859700000000	-1.561182000000
H	-0.389490000000	-3.637913000000	3.397503000000
H	-0.048082000000	-2.726151000000	5.697368000000
H	-1.584115000000	-0.991357000000	6.576046000000
H	-3.438981000000	-0.142519000000	5.169612000000
H	-3.735539000000	-1.028275000000	2.838154000000
H	2.798320000000	-3.836815000000	-2.914313000000
H	2.050459000000	-5.089934000000	-1.908513000000
H	1.671765000000	-4.993335000000	-3.637051000000
H	1.359452000000	-2.038770000000	-3.948997000000
H	-0.271250000000	-1.724776000000	-3.366980000000
H	0.035615000000	-3.081839000000	-4.476495000000
H	-0.345412000000	-0.611383000000	0.783720000000

C	0.780386000000	1.408248000000	-0.907324000000
C	-0.597979000000	1.451041000000	-0.961707000000
N	-1.337049000000	0.430155000000	-1.481620000000
C	-1.217584000000	2.748186000000	-0.548948000000
C	-1.919898000000	3.518245000000	-1.480510000000
C	-2.471299000000	4.736907000000	-1.106173000000
C	-2.328616000000	5.193106000000	0.204534000000
C	-1.623227000000	4.431797000000	1.132361000000
C	-1.057370000000	3.213461000000	0.758322000000
S	-2.762750000000	0.013219000000	-0.888614000000
C	-3.966638000000	1.335191000000	-0.907558000000
C	-4.252526000000	2.024812000000	0.266576000000
C	-5.138499000000	3.092046000000	0.220844000000
C	-5.741262000000	3.477786000000	-0.980871000000
C	-5.458943000000	2.749764000000	-2.138799000000
C	-4.574062000000	1.676292000000	-2.109417000000
C	-6.657011000000	4.673621000000	-1.014409000000
O	-2.655026000000	-0.378670000000	0.535182000000
O	-3.315665000000	-1.007771000000	-1.799192000000
C	2.937852000000	0.351046000000	-1.684929000000
C	3.500483000000	-0.759226000000	-2.329642000000
C	4.811229000000	-0.745474000000	-2.786441000000
C	5.590250000000	0.394413000000	-2.610851000000
C	5.041953000000	1.514157000000	-1.989084000000
C	3.730663000000	1.497594000000	-1.527654000000
H	1.281791000000	2.319370000000	-0.598246000000
H	0.988172000000	-0.587145000000	-1.590217000000
H	-2.034089000000	3.144681000000	-2.493881000000
H	-3.018912000000	5.328120000000	-1.834223000000
H	-2.766048000000	6.142383000000	0.499913000000
H	-1.510695000000	4.786705000000	2.152471000000
H	-0.502557000000	2.601314000000	1.465400000000
H	-3.762796000000	1.729938000000	1.188593000000
H	-5.354864000000	3.649944000000	1.128464000000
H	-5.935633000000	3.029837000000	-3.074376000000
H	-4.348596000000	1.103685000000	-3.003770000000
H	-7.441348000000	4.592615000000	-0.256420000000
H	-6.095902000000	5.591040000000	-0.806404000000
H	-7.134878000000	4.784548000000	-1.990085000000
H	2.892777000000	-1.650727000000	-2.449650000000
H	5.225539000000	-1.626558000000	-3.266852000000
H	6.617048000000	0.414112000000	-2.962736000000
H	5.636309000000	2.412533000000	-1.857225000000
H	3.329665000000	2.384544000000	-1.047242000000

----

^{rr}TSb3

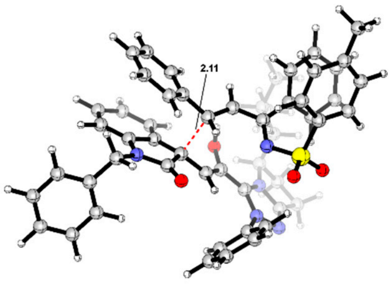

Frequency -359.7522

Zero-point correction= 0.947196 (Hartree/Particle)

Thermal correction to Energy= 1.002449

Thermal correction to Enthalpy= 1.003393

Thermal correction to Gibbs Free Energy= 0.854563

Sum of electronic and zero-point Energies= -3059.203562

Sum of electronic and thermal Energies= -3059.148310

Sum of electronic and thermal Enthalpies= -3059.147365

Sum of electronic and thermal Free Energies= -3059.296196

E(RM062X) = -3060.81557435

C	1.946138000000	-0.264461000000	0.343041000000
C	0.693332000000	1.427351000000	0.494310000000

C	1.020701000000	-1.177693000000	-0.260943000000	C	1.560637000000	2.334728000000	1.275263000000
C	0.091584000000	-1.941740000000	0.362942000000	C	1.581795000000	2.335853000000	2.675918000000
O	-0.115569000000	-1.923638000000	1.733450000000	C	2.441422000000	3.176235000000	3.371530000000
H	-0.280194000000	-0.990275000000	1.948647000000	C	3.290053000000	4.037743000000	2.678710000000
C	2.702243000000	-0.357171000000	1.611858000000	C	3.269465000000	4.056585000000	1.286199000000
C	3.931485000000	0.309950000000	1.397439000000	C	2.414439000000	3.207375000000	0.590492000000
C	4.863768000000	0.520966000000	2.398905000000	C	-3.049056000000	0.975613000000	0.585188000000
C	4.563515000000	0.043532000000	3.675526000000	C	-3.395221000000	2.070774000000	1.385532000000
C	2.446546000000	-0.851722000000	2.888097000000	C	-4.689628000000	2.206442000000	1.877718000000
C	3.376333000000	-0.641329000000	3.911849000000	C	-5.651283000000	1.242495000000	1.585117000000
N	4.029765000000	0.703122000000	0.065634000000	C	-5.313329000000	0.146923000000	0.793344000000
C	2.896327000000	0.345777000000	-0.636343000000	C	-4.023556000000	0.016973000000	0.287594000000
O	2.715919000000	0.559883000000	-1.825222000000	H	5.273927000000	0.193523000000	4.482024000000
C	-0.610921000000	1.102317000000	0.961533000000	H	5.800524000000	1.026898000000	2.189447000000
C	-1.652056000000	0.871609000000	0.046299000000	H	1.545929000000	-1.413400000000	3.095869000000
N	-1.352799000000	0.647475000000	-1.237861000000	H	3.167699000000	-1.029871000000	4.903460000000
S	-2.397126000000	0.885817000000	-2.466356000000	H	0.735077000000	1.568471000000	-0.586877000000
O	-1.587892000000	1.155841000000	-3.652568000000	H	-1.481300000000	3.532171000000	-1.901164000000
O	-3.388310000000	-0.198157000000	-2.584634000000	H	-2.681798000000	5.586926000000	-1.167333000000
C	-3.289660000000	2.381356000000	-2.064024000000	H	-6.415254000000	3.491746000000	-1.414174000000
C	-2.563047000000	3.540289000000	-1.800930000000	H	-5.201932000000	1.429903000000	-2.142939000000
C	-3.239098000000	4.680235000000	-1.387999000000	H	-5.430309000000	6.643856000000	-1.613591000000
C	-4.631336000000	4.677194000000	-1.240511000000	H	-6.363278000000	5.694776000000	-0.451715000000
C	-5.334841000000	3.507244000000	-1.530111000000	H	-4.817009000000	6.415942000000	0.023793000000
C	-4.671556000000	2.355193000000	-1.943511000000	H	7.651282000000	2.059131000000	0.299749000000
C	-5.351126000000	5.923167000000	-0.793175000000	H	9.708064000000	0.719354000000	-0.042090000000
C	5.111757000000	1.466503000000	-0.508775000000	H	9.587731000000	-1.454518000000	-1.231370000000
C	6.372293000000	0.650853000000	-0.697451000000	H	7.405644000000	-2.277115000000	-2.079485000000
C	7.600279000000	1.107686000000	-0.225076000000	H	5.349647000000	-0.923388000000	-1.738330000000
C	8.757010000000	0.354875000000	-0.417369000000	H	5.309448000000	2.338550000000	0.127733000000
C	8.689019000000	-0.864405000000	-1.082697000000	H	4.738342000000	1.822478000000	-1.474920000000
C	7.461887000000	-1.326813000000	-1.557718000000	H	0.945525000000	1.648200000000	3.224476000000
C	6.310136000000	-0.573317000000	-1.367093000000	H	2.459733000000	3.148745000000	4.456195000000

H	3.963095000000	4.692571000000	3.223192000000
H	3.920328000000	4.732076000000	0.738890000000
H	2.410081000000	3.198817000000	-0.497589000000
H	-2.648068000000	2.833109000000	1.584574000000
H	-4.949976000000	3.072073000000	2.478635000000
H	-6.661932000000	1.348121000000	1.967298000000
H	-6.060663000000	-0.605444000000	0.558058000000
H	-3.768505000000	-0.813366000000	-0.362030000000
C	-0.794673000000	-2.861602000000	-0.301896000000
N	-0.876020000000	-3.245382000000	-1.593894000000
N	-1.870093000000	-4.164200000000	-1.810727000000
C	-2.376861000000	-4.346023000000	-0.631409000000
N	-1.746579000000	-3.594364000000	0.318151000000
C	-3.486775000000	-5.136274000000	-0.021488000000
C	-3.122731000000	-5.060726000000	1.484108000000
C	-2.288751000000	-3.763848000000	1.681089000000
C	-3.084720000000	-2.527975000000	2.103210000000
C	-3.101703000000	-2.289698000000	3.614723000000
C	-0.040848000000	-2.794713000000	-2.671261000000
C	1.249859000000	-3.304425000000	-2.775116000000
C	2.104078000000	-2.748868000000	-3.719530000000
C	1.662988000000	-1.701065000000	-4.528779000000
C	0.359106000000	-1.225518000000	-4.425328000000
C	-0.512541000000	-1.784381000000	-3.495054000000
C	-3.847770000000	-0.990479000000	3.916511000000
C	-3.702370000000	-3.459655000000	4.393672000000
H	-4.441957000000	-4.647442000000	-0.237068000000
H	-3.534051000000	-6.156516000000	-0.402253000000
H	-2.514571000000	-5.928254000000	1.753044000000
H	-4.008294000000	-5.057277000000	2.120322000000
H	-1.451415000000	-3.917417000000	2.363888000000
H	-4.113029000000	-2.596535000000	1.717439000000
H	-2.634474000000	-1.651940000000	1.629992000000

H	-2.053358000000	-2.170581000000	3.926502000000
H	1.580895000000	-4.087948000000	-2.100971000000
H	3.120264000000	-3.117710000000	-3.808995000000
H	2.348760000000	-1.245700000000	-5.235285000000
H	0.011504000000	-0.390138000000	-5.022114000000
H	-1.530352000000	-1.426873000000	-3.386695000000
H	-3.815654000000	-0.758869000000	4.985138000000
H	-3.419980000000	-0.145835000000	3.365074000000
H	-4.899084000000	-1.073396000000	3.618874000000
H	-3.716889000000	-3.243420000000	5.465609000000
H	-3.135645000000	-4.386535000000	4.253568000000
H	-4.737015000000	-3.641707000000	4.077728000000
H	-0.874688000000	1.261252000000	2.003666000000
H	1.021649000000	-1.137896000000	-1.346322000000

----

^{rr}**TSb3**

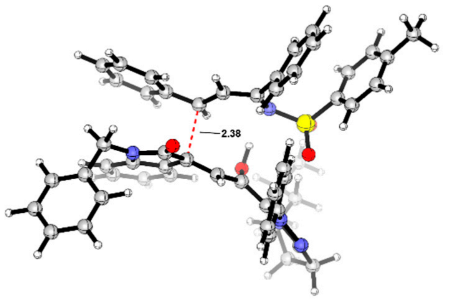

Frequency -252.3950

Zero-point correction= 0.947485 (Hartree/Particle)

Thermal correction to Energy= 1.002422

Thermal correction to Enthalpy= 1.003366

Thermal correction to Gibbs Free Energy= 0.855464

Sum of electronic and zero-point Energies= -3059.210347

Sum of electronic and thermal Energies= -3059.155410

Sum of electronic and thermal Enthalpies= -3059.154466

Sum of electronic and thermal Free Energies= -3059.302368

E(RM062X) = -3060.82310659

C	2.025249000000	0.126585000000	-0.151814000000
C	0.993313000000	-1.214139000000	-1.827233000000
C	0.973192000000	0.544182000000	0.713604000000
C	-0.023837000000	1.414713000000	0.402254000000
O	-0.298169000000	1.810446000000	-0.883906000000
H	-0.784698000000	1.056456000000	-1.305737000000
C	2.831813000000	0.946906000000	-1.055003000000
C	4.125216000000	0.371308000000	-1.080677000000
C	5.157695000000	0.892330000000	-1.843070000000
C	4.886068000000	2.017188000000	-2.625641000000
C	2.587439000000	2.073469000000	-1.837237000000
C	3.619244000000	2.596800000000	-2.622807000000
N	4.149269000000	-0.741328000000	-0.246483000000
C	2.921645000000	-0.910862000000	0.377983000000
O	2.671767000000	-1.794798000000	1.194012000000
C	0.029725000000	-2.015561000000	-1.222716000000
C	-1.311643000000	-1.668951000000	-1.014225000000
N	-1.826414000000	-0.508894000000	-1.447859000000
S	-3.186762000000	0.214932000000	-0.892253000000
O	-2.920520000000	0.758127000000	0.453200000000
O	-3.571254000000	1.158961000000	-1.939518000000
C	-4.574305000000	-0.883135000000	-0.682878000000
C	-4.870710000000	-1.376583000000	0.584052000000
C	-5.937046000000	-2.251673000000	0.729694000000
C	-6.708970000000	-2.635696000000	-0.372845000000
C	-6.409536000000	-2.095681000000	-1.625801000000
C	-5.347764000000	-1.211635000000	-1.789239000000
C	-7.825828000000	-3.631668000000	-0.202442000000
C	5.263707000000	-1.641973000000	-0.064143000000
C	6.286152000000	-1.114670000000	0.918545000000
C	7.610610000000	-0.901870000000	0.546342000000
C	8.535285000000	-0.415353000000	1.469648000000
C	8.135165000000	-0.138869000000	2.772129000000
C	6.809352000000	-0.351681000000	3.150690000000
C	5.889269000000	-0.837109000000	2.229808000000
C	2.210840000000	-1.796927000000	-2.403216000000
C	2.744568000000	-3.012026000000	-1.938359000000
C	3.871640000000	-3.558433000000	-2.532184000000
C	4.493829000000	-2.903251000000	-3.598163000000
C	3.988570000000	-1.689671000000	-4.051544000000
C	2.858346000000	-1.137111000000	-3.455030000000
C	-2.121006000000	-2.774438000000	-0.410600000000
C	-1.798765000000	-3.231391000000	0.867536000000
C	-2.502088000000	-4.294786000000	1.428703000000
C	-3.504498000000	-4.928633000000	0.699797000000
C	-3.790280000000	-4.508815000000	-0.599116000000
C	-3.099911000000	-3.438662000000	-1.154508000000
H	5.675291000000	2.445260000000	-3.235502000000
H	6.145614000000	0.442756000000	-1.823631000000
H	1.603049000000	2.525800000000	-1.841822000000
H	3.427806000000	3.472252000000	-3.235378000000
H	0.683118000000	-0.262099000000	-2.251838000000
H	-4.264209000000	-1.076537000000	1.432161000000
H	-6.170950000000	-2.654328000000	1.711645000000
H	-7.019200000000	-2.366702000000	-2.483226000000
H	-5.115386000000	-0.775725000000	-2.755970000000
H	-8.447084000000	-3.384121000000	0.662299000000
H	-8.466095000000	-3.669364000000	-1.086117000000
H	-7.419626000000	-4.635474000000	-0.036861000000
H	7.921832000000	-1.119345000000	-0.473116000000
H	9.565078000000	-0.249487000000	1.168775000000
H	8.852264000000	0.242415000000	3.492310000000
H	6.495759000000	-0.139697000000	4.168283000000
H	4.853392000000	-1.015168000000	2.510605000000
H	5.721264000000	-1.839775000000	-1.040483000000

H	4.831235000000	-2.577895000000	0.305200000000
H	2.300984000000	-3.502300000000	-1.077581000000
H	4.275675000000	-4.494255000000	-2.158461000000
H	5.374369000000	-3.336051000000	-4.062823000000
H	4.478613000000	-1.161570000000	-4.863072000000
H	2.476822000000	-0.178117000000	-3.794993000000
H	-0.977857000000	-2.757084000000	1.399336000000
H	-2.250276000000	-4.645633000000	2.425419000000
H	-4.047310000000	-5.762549000000	1.133848000000
H	-4.553208000000	-5.016836000000	-1.181239000000
H	-3.315170000000	-3.108430000000	-2.165656000000
C	-0.794053000000	2.144675000000	1.381210000000
N	-1.071819000000	1.919964000000	2.682891000000
N	-1.701202000000	2.990388000000	3.281198000000
C	-1.814199000000	3.847648000000	2.316684000000
N	-1.287574000000	3.377478000000	1.151028000000
C	-2.301331000000	5.247814000000	2.134776000000
C	-1.496169000000	5.666139000000	0.877178000000
C	-1.294292000000	4.365494000000	0.053621000000
C	-2.407612000000	4.083834000000	-0.959549000000
C	-2.021621000000	4.495377000000	-2.383892000000
C	-0.864068000000	0.741905000000	3.463520000000
C	-1.374942000000	-0.474267000000	3.022292000000
C	-1.221363000000	-1.588029000000	3.841744000000
C	-0.567735000000	-1.482849000000	5.067208000000
C	-0.060732000000	-0.256099000000	5.486717000000
C	-0.209097000000	0.868873000000	4.683662000000
C	-3.160769000000	4.160674000000	-3.344686000000
C	-1.640924000000	5.973234000000	-2.488456000000
H	-3.378954000000	5.242899000000	1.943597000000
H	-2.106266000000	5.873950000000	3.005182000000
H	-0.525083000000	6.061827000000	1.188629000000
H	-2.006987000000	6.429641000000	0.289343000000
H	-0.316930000000	4.329457000000	-0.434194000000
H	-3.321158000000	4.616060000000	-0.654216000000
H	-2.642399000000	3.018625000000	-0.957014000000
H	-1.146437000000	3.888012000000	-2.656153000000
H	-1.888085000000	-0.519591000000	2.064398000000
H	-1.633675000000	-2.540128000000	3.525900000000
H	-0.456232000000	-2.359133000000	5.697005000000
H	0.449351000000	-0.171992000000	6.440189000000
H	0.160782000000	1.840291000000	4.992530000000
H	-2.878332000000	4.384963000000	-4.377815000000
H	-3.430481000000	3.104886000000	-3.270851000000
H	-4.047855000000	4.758815000000	-3.103946000000
H	-1.376414000000	6.232100000000	-3.517838000000
H	-0.785183000000	6.234632000000	-1.855630000000
H	-2.486322000000	6.608535000000	-2.195198000000
H	0.297044000000	-3.024782000000	-0.935743000000
H	1.009257000000	0.130581000000	1.721825000000

----

^{rr}INTb6

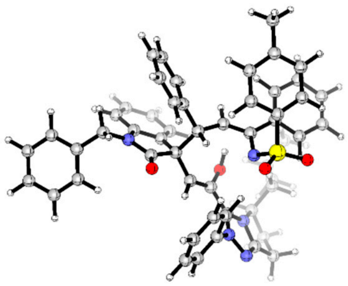

Zero-point correction= 0.949206 (Hartree/Particle)

Thermal correction to Energy= 1.004475

Thermal correction to Enthalpy= 1.005419

Thermal correction to Gibbs Free Energy= 0.853120

Sum of electronic and zero-point Energies= -3059.217983

Sum of electronic and thermal Energies= -3059.162715

Sum of electronic and thermal Enthalpies= -3059.161770

Sum of electronic and thermal Free Energies= -3059.314069

E(RM062X) = -3060.83111967

C	-1.619116000000	0.256335000000	0.357249000000
C	-0.713143000000	-1.026605000000	0.040243000000
C	-0.914313000000	1.449991000000	-0.224480000000
C	0.185831000000	2.028636000000	0.263331000000
O	0.782256000000	1.716559000000	1.451755000000
H	0.925267000000	0.733240000000	1.414750000000
C	-2.134839000000	0.244026000000	1.782958000000
C	-3.469961000000	-0.193654000000	1.744723000000
C	-4.214428000000	-0.431916000000	2.888506000000
C	-3.588985000000	-0.225097000000	4.119388000000
C	-1.535554000000	0.449271000000	3.016653000000
C	-2.271042000000	0.213206000000	4.184591000000
N	-3.891185000000	-0.347335000000	0.420541000000
C	-2.903413000000	0.018300000000	-0.454439000000
O	-3.010127000000	0.084976000000	-1.663134000000
C	0.604616000000	-1.053534000000	0.770052000000
C	1.795293000000	-0.911627000000	0.103336000000
N	1.811441000000	-0.397367000000	-1.170509000000
S	2.637310000000	-1.142648000000	-2.342541000000
O	1.868015000000	-0.939345000000	-3.578846000000
O	4.068032000000	-0.810715000000	-2.416038000000
C	2.541626000000	-2.875776000000	-1.898732000000
C	1.283219000000	-3.458195000000	-1.768452000000
C	1.173589000000	-4.746897000000	-1.266583000000
C	2.316553000000	-5.471318000000	-0.906119000000
C	3.567887000000	-4.870974000000	-1.056180000000
C	3.688883000000	-3.570851000000	-1.542542000000
C	2.190187000000	-6.877735000000	-0.379146000000
C	-5.154012000000	-0.917981000000	-0.001180000000
C	-6.312009000000	0.042436000000	0.153683000000
C	-7.444160000000	-0.301589000000	0.887863000000
C	-8.504398000000	0.595028000000	1.011864000000
C	-8.433493000000	1.842490000000	0.401537000000
C	-7.302209000000	2.191083000000	-0.336358000000
C	-6.247188000000	1.295541000000	-0.460533000000
C	-1.505382000000	-2.310451000000	0.230374000000
C	-1.693467000000	-2.892080000000	1.488080000000
C	-2.450125000000	-4.052404000000	1.628514000000
C	-3.030259000000	-4.653153000000	0.514020000000
C	-2.855410000000	-4.080354000000	-0.743788000000
C	-2.101709000000	-2.917595000000	-0.881288000000
C	3.070457000000	-1.273655000000	0.801416000000
C	3.114291000000	-2.322101000000	1.728589000000
C	4.309073000000	-2.663836000000	2.352956000000
C	5.481612000000	-1.975548000000	2.044387000000
C	5.451768000000	-0.946185000000	1.107011000000
C	4.254638000000	-0.593330000000	0.491958000000
H	-4.145411000000	-0.401807000000	5.034089000000
H	-5.248543000000	-0.753028000000	2.825384000000
H	-0.514797000000	0.801713000000	3.085379000000
H	-1.804402000000	0.378344000000	5.149711000000
H	-0.478712000000	-0.919542000000	-1.025860000000
H	0.399459000000	-2.890628000000	-2.043802000000
H	0.188180000000	-5.189350000000	-1.138085000000
H	4.460085000000	-5.420976000000	-0.768578000000
H	4.651502000000	-3.076059000000	-1.624096000000
H	2.060629000000	-7.591530000000	-1.199403000000
H	3.080880000000	-7.173404000000	0.180124000000
H	1.322585000000	-6.974627000000	0.278914000000
H	-7.497818000000	-1.277803000000	1.364547000000
H	-9.381802000000	0.318652000000	1.587700000000
H	-9.256536000000	2.543082000000	0.498453000000
H	-7.246434000000	3.162181000000	-0.817840000000

H	-5.364223000000	1.552848000000	-1.041072000000
H	-5.324902000000	-1.838339000000	0.569579000000
H	-5.016847000000	-1.189743000000	-1.052477000000
H	-1.260135000000	-2.428075000000	2.369749000000
H	-2.586335000000	-4.487505000000	2.613637000000
H	-3.614770000000	-5.560998000000	0.624911000000
H	-3.304486000000	-4.537464000000	-1.620092000000
H	-1.985325000000	-2.456713000000	-1.859192000000
H	2.214319000000	-2.900645000000	1.915180000000
H	4.331204000000	-3.485824000000	3.061611000000
H	6.416742000000	-2.253564000000	2.520661000000
H	6.364175000000	-0.417595000000	0.848687000000
H	4.229652000000	0.180552000000	-0.266594000000
C	0.949839000000	3.063042000000	-0.437792000000
N	0.894506000000	3.520863000000	-1.698301000000
N	1.790441000000	4.529348000000	-1.923876000000
C	2.395493000000	4.664891000000	-0.781697000000
N	1.915032000000	3.793257000000	0.148755000000
C	3.484878000000	5.504349000000	-0.202152000000
C	3.237744000000	5.306319000000	1.315891000000
C	2.548639000000	3.922342000000	1.480852000000
C	3.492363000000	2.753770000000	1.801496000000
C	3.481821000000	2.432407000000	3.302567000000
C	0.112599000000	3.012938000000	-2.795988000000
C	-0.823547000000	3.844677000000	-3.398629000000
C	-1.569499000000	3.330473000000	-4.454181000000
C	-1.369718000000	2.016931000000	-4.879882000000
C	-0.416585000000	1.209401000000	-4.263798000000
C	0.341446000000	1.708489000000	-3.210411000000
C	4.276872000000	1.161933000000	3.593783000000
C	3.994057000000	3.604013000000	4.142630000000
H	4.454983000000	5.106709000000	-0.515325000000
H	3.424231000000	6.545751000000	-0.517429000000
H	2.569791000000	6.092528000000	1.677713000000
H	4.161204000000	5.352556000000	1.893477000000
H	1.747623000000	3.954825000000	2.222112000000
H	4.513504000000	2.995533000000	1.473351000000
H	3.174630000000	1.868726000000	1.245614000000
H	2.432365000000	2.245492000000	3.572007000000
H	-0.957315000000	4.862397000000	-3.047610000000
H	-2.309259000000	3.955906000000	-4.941961000000
H	-1.962363000000	1.624518000000	-5.699623000000
H	-0.229735000000	0.189767000000	-4.582540000000
H	1.067066000000	1.075691000000	-2.708710000000
H	4.289827000000	0.954675000000	4.667991000000
H	3.848432000000	0.296123000000	3.081051000000
H	5.315146000000	1.264178000000	3.258524000000
H	3.983001000000	3.350749000000	5.206241000000
H	3.387065000000	4.507498000000	4.016557000000
H	5.027957000000	3.849125000000	3.870381000000
H	0.618666000000	-1.393286000000	1.802596000000
H	-1.277877000000	1.747101000000	-1.202803000000

----

*rs*INTb6

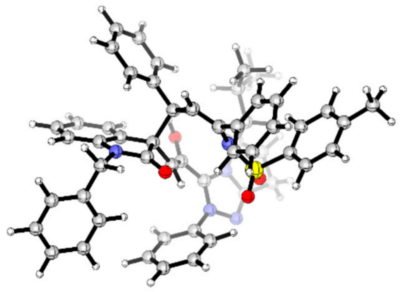

Zero-point correction=	0.948375 (Hartree/Particle)
Thermal correction to Energy=	1.004029
Thermal correction to Enthalpy=	1.004973
Thermal correction to Gibbs Free Energy=	0.854823

Sum of electronic and zero-point Energies= -3059.220473

Sum of electronic and thermal Energies= -3059.164819

Sum of electronic and thermal Enthalpies= -3059.163875

Sum of electronic and thermal Free Energies= -3059.314025

E(RM062X) = -3060.83549864

C	1.673693000000	-0.668232000000	0.473448000000
C	0.532841000000	-1.184900000000	1.553029000000
C	1.034104000000	-0.574292000000	-0.879630000000
C	0.523915000000	-1.600002000000	-1.567949000000
O	0.497037000000	-2.922994000000	-1.192702000000
H	0.656560000000	-2.983422000000	-0.239425000000
C	2.980820000000	-1.440966000000	0.554737000000
C	3.940559000000	-0.615056000000	1.159999000000
C	5.227850000000	-1.044214000000	1.439030000000
C	5.559444000000	-2.357221000000	1.100604000000
C	3.336209000000	-2.736991000000	0.214167000000
C	4.629008000000	-3.192280000000	0.492712000000
N	3.397790000000	0.651500000000	1.411388000000
C	2.139151000000	0.751315000000	0.850729000000
O	1.553062000000	1.791040000000	0.650800000000
C	-0.199263000000	-0.062392000000	2.215913000000
C	-1.120744000000	0.664784000000	1.540675000000
N	-1.455883000000	0.289339000000	0.255351000000
S	-2.295173000000	1.197489000000	-0.740577000000
O	-1.701665000000	2.511478000000	-1.043912000000
O	-2.578131000000	0.350451000000	-1.923226000000
C	-3.878726000000	1.546298000000	0.023008000000
C	-4.278570000000	2.860190000000	0.229123000000
C	-5.464858000000	3.107559000000	0.911968000000
C	-6.253724000000	2.059608000000	1.391731000000
C	-5.836707000000	0.745551000000	1.158868000000
C	-4.652548000000	0.485160000000	0.480599000000
C	-7.504329000000	2.345935000000	2.182319000000
C	4.173629000000	1.827286000000	1.756618000000
C	5.153414000000	2.221044000000	0.670190000000
C	6.500479000000	2.419080000000	0.964626000000
C	7.399126000000	2.786079000000	-0.035315000000
C	6.952213000000	2.951961000000	-1.341377000000
C	5.604945000000	2.753617000000	-1.641431000000
C	4.707111000000	2.391935000000	-0.643892000000
C	1.092921000000	-2.178062000000	2.541378000000
C	2.028744000000	-1.795043000000	3.507975000000
C	2.561116000000	-2.729361000000	4.387150000000
C	2.159361000000	-4.063648000000	4.324789000000
C	1.215960000000	-4.452890000000	3.381325000000
C	0.689522000000	-3.512697000000	2.496952000000
C	-1.758377000000	1.832831000000	2.227919000000
C	-1.318838000000	3.130234000000	1.952294000000
C	-1.930747000000	4.216055000000	2.573002000000
C	-2.982876000000	4.016466000000	3.464349000000
C	-3.418185000000	2.723273000000	3.745062000000
C	-2.802467000000	1.635018000000	3.133845000000
H	6.560075000000	-2.722760000000	1.306424000000
H	5.955968000000	-0.376436000000	1.886680000000
H	2.641229000000	-3.403851000000	-0.282996000000
H	4.905710000000	-4.205969000000	0.224771000000
H	-0.201112000000	-1.701848000000	0.920884000000
H	-3.640149000000	3.664889000000	-0.119245000000
H	-5.774917000000	4.133570000000	1.093402000000
H	-6.442170000000	-0.079300000000	1.526268000000
H	-4.305082000000	-0.533050000000	0.332386000000
H	-8.055931000000	3.189999000000	1.760320000000
H	-8.169034000000	1.479255000000	2.205372000000
H	-7.255049000000	2.602312000000	3.217676000000
H	6.850427000000	2.284266000000	1.985653000000
H	8.446432000000	2.935545000000	0.206674000000

H	7.648929000000	3.234085000000	-2.124074000000
H	5.252469000000	2.885969000000	-2.659862000000
H	3.654198000000	2.250350000000	-0.878371000000
H	4.701825000000	1.643448000000	2.698430000000
H	3.442325000000	2.623280000000	1.922699000000
H	2.347289000000	-0.756354000000	3.556758000000
H	3.294118000000	-2.417934000000	5.124649000000
H	2.577256000000	-4.791637000000	5.012404000000
H	0.886030000000	-5.485810000000	3.331839000000
H	-0.067383000000	-3.818260000000	1.774958000000
H	-0.510951000000	3.266878000000	1.240949000000
H	-1.586342000000	5.222962000000	2.357154000000
H	-3.462489000000	4.866931000000	3.939644000000
H	-4.238996000000	2.561523000000	4.437707000000
H	-3.140866000000	0.622542000000	3.335744000000
C	-0.327106000000	-1.376509000000	-2.727154000000
N	-0.291537000000	-0.417889000000	-3.662347000000
N	-1.389865000000	-0.446314000000	-4.473586000000
C	-2.067524000000	-1.461923000000	-4.028083000000
N	-1.424617000000	-2.087381000000	-3.003193000000
C	-3.434115000000	-2.034795000000	-4.202314000000
C	-3.327405000000	-3.349065000000	-3.384716000000
C	-2.227296000000	-3.114311000000	-2.314007000000
C	-2.706549000000	-2.590759000000	-0.952886000000
C	-2.677279000000	-3.652671000000	0.146546000000
C	0.634006000000	0.674860000000	-3.733053000000
C	0.149631000000	1.941961000000	-3.434098000000
C	1.056294000000	2.996875000000	-3.394006000000
C	2.406147000000	2.773481000000	-3.656166000000
C	2.866119000000	1.491632000000	-3.959902000000
C	1.974762000000	0.425032000000	-4.000446000000
C	-2.987767000000	-3.006535000000	1.497663000000
C	-3.624755000000	-4.816482000000	-0.143946000000

H	-4.133360000000	-1.326970000000	-3.745190000000
H	-3.716542000000	-2.192345000000	-5.242946000000
H	-3.026104000000	-4.165456000000	-4.046708000000
H	-4.272907000000	-3.622682000000	-2.914340000000
H	-1.599435000000	-3.998123000000	-2.179736000000
H	-3.721326000000	-2.183618000000	-1.061562000000
H	-2.085580000000	-1.741122000000	-0.646390000000
H	-1.649050000000	-4.047835000000	0.176982000000
H	-0.895067000000	2.073883000000	-3.172575000000
H	0.702877000000	3.987024000000	-3.129830000000
H	3.106068000000	3.602485000000	-3.613760000000
H	3.918600000000	1.318463000000	-4.158679000000
H	2.309036000000	-0.584648000000	-4.214861000000
H	-2.855564000000	-3.719336000000	2.317534000000
H	-2.347510000000	-2.135771000000	1.685674000000
H	-4.029112000000	-2.663017000000	1.522176000000
H	-3.587083000000	-5.567097000000	0.650790000000
H	-3.381381000000	-5.319954000000	-1.086610000000
H	-4.657370000000	-4.454035000000	-0.213384000000
H	0.065836000000	0.210011000000	3.231467000000
H	0.859684000000	0.431632000000	-1.249069000000

----

^{sr}INTb6

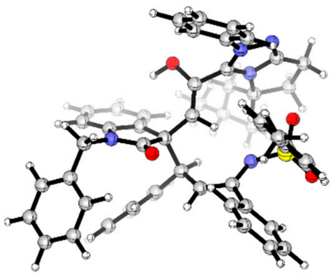

Zero-point correction= 0.949895 (Hartree/Particle)

Thermal correction to Energy= 1.005172

Thermal correction to Enthalpy= 1.006116

Thermal correction to Gibbs Free Energy= 0.857773

Sum of electronic and zero-point Energies= -3059.225630

Sum of electronic and thermal Energies= -3059.170352

Sum of electronic and thermal Enthalpies= -3059.169408

Sum of electronic and thermal Free Energies= -3059.317751

E(RM062X) = -3060.84294923

C	1.463459000000	0.541461000000	-0.302698000000
C	0.030398000000	0.847632000000	-0.625039000000
C	-0.455829000000	1.829188000000	-1.387903000000
O	0.179457000000	2.742188000000	-2.179834000000
H	1.137896000000	2.653287000000	-2.051159000000
C	2.466707000000	1.619501000000	-0.628474000000
C	3.493724000000	1.072651000000	-1.411423000000
C	4.602192000000	1.822139000000	-1.779054000000
C	4.661275000000	3.150634000000	-1.348424000000
C	2.545848000000	2.934141000000	-0.199809000000
C	3.649130000000	3.709168000000	-0.572714000000
N	3.186353000000	-0.252004000000	-1.717304000000
C	1.998595000000	-0.649530000000	-1.124244000000
O	1.507220000000	-1.749429000000	-1.257472000000
C	3.967229000000	-1.148039000000	-2.554275000000
C	4.980292000000	-1.961890000000	-1.779191000000
C	6.323318000000	-1.592444000000	-1.735340000000
C	7.242761000000	-2.352101000000	-1.014923000000
C	6.820936000000	-3.488261000000	-0.330615000000
C	5.477033000000	-3.860522000000	-0.365070000000
C	4.561621000000	-3.103819000000	-1.089467000000
H	5.519780000000	3.754392000000	-1.623395000000
H	5.404232000000	1.394439000000	-2.369996000000
H	1.757935000000	3.345711000000	0.425883000000
H	3.722897000000	4.740175000000	-0.244106000000
H	6.658514000000	-0.712040000000	-2.277991000000
H	8.287105000000	-2.058019000000	-0.991798000000
H	7.535814000000	-4.083294000000	0.228610000000
H	5.143413000000	-4.744766000000	0.168288000000
H	3.510970000000	-3.382382000000	-1.121722000000
H	4.458445000000	-0.548026000000	-3.326758000000
H	3.247565000000	-1.813497000000	-3.039070000000
C	-1.903921000000	2.112881000000	-1.294112000000
N	-2.913277000000	1.773586000000	-2.106790000000
N	-4.120363000000	2.184156000000	-1.622367000000
C	-3.816711000000	2.782316000000	-0.503119000000
N	-2.476663000000	2.780286000000	-0.293873000000
C	-4.545095000000	3.405902000000	0.635160000000
C	-3.397630000000	3.736676000000	1.636763000000
C	-2.038154000000	3.275952000000	1.022364000000
C	-0.969735000000	4.362319000000	0.871768000000
C	-0.067679000000	4.488677000000	2.105084000000
C	-2.850909000000	0.974424000000	-3.290864000000
C	-1.789254000000	1.127334000000	-4.176379000000
C	-1.744467000000	0.309039000000	-5.300865000000
C	-2.750234000000	-0.625257000000	-5.533944000000
C	-3.810063000000	-0.751120000000	-4.638066000000
C	-3.866953000000	0.047430000000	-3.501640000000
C	0.949148000000	5.610969000000	1.895184000000
C	-0.857655000000	4.715292000000	3.394613000000
H	-5.101076000000	4.289430000000	0.314509000000
H	-5.232217000000	2.670944000000	1.054711000000
H	-3.569161000000	3.198557000000	2.567217000000
H	-3.363128000000	4.808732000000	1.843916000000
H	-1.650888000000	2.396362000000	1.553080000000
H	-1.451744000000	5.327484000000	0.662074000000
H	-0.343414000000	4.134333000000	-0.000921000000
H	0.472188000000	3.535048000000	2.210873000000
H	-1.024000000000	1.874037000000	-3.991845000000

H	-0.922981000000	0.412165000000	-6.001198000000
H	-2.708767000000	-1.255900000000	-6.415498000000
H	-4.591870000000	-1.482230000000	-4.813940000000
H	-4.665728000000	-0.046186000000	-2.774682000000
H	1.675683000000	5.641051000000	2.711841000000
H	1.496896000000	5.494254000000	0.953978000000
H	0.441010000000	6.581104000000	1.861559000000
H	-0.177960000000	4.837815000000	4.242308000000
H	-1.519080000000	3.874979000000	3.622532000000
H	-1.466568000000	5.624101000000	3.320214000000
H	-0.696288000000	0.274273000000	-0.055929000000
C	0.757737000000	-1.074125000000	1.591783000000
C	-0.553583000000	-1.204729000000	1.913421000000
C	1.485803000000	0.194845000000	1.267949000000
N	-1.423434000000	-0.121458000000	1.912781000000
C	-1.000792000000	-2.603694000000	2.221473000000
C	-1.576632000000	-2.935601000000	3.451178000000
C	-1.984285000000	-4.243051000000	3.700023000000
C	-1.830517000000	-5.226843000000	2.725641000000
C	-1.248221000000	-4.902441000000	1.502165000000
C	-0.828105000000	-3.599737000000	1.253904000000
C	-3.475758000000	-1.621708000000	0.881073000000
C	-3.090159000000	-1.550599000000	-0.453521000000
C	-3.376082000000	-2.609165000000	-1.308300000000
C	-4.053101000000	-3.739832000000	-0.843697000000
C	-4.455774000000	-3.776573000000	0.493512000000
C	-4.170312000000	-2.726006000000	1.358556000000
C	-4.308897000000	-4.914612000000	-1.752135000000
S	-3.004903000000	-0.304818000000	2.009530000000
O	-3.598854000000	0.923762000000	1.431409000000
O	-3.533809000000	-0.684099000000	3.325153000000
C	2.918758000000	0.180362000000	1.762352000000
C	3.431190000000	1.247333000000	2.502056000000

C	4.769593000000	1.275007000000	2.883291000000
C	5.620415000000	0.235230000000	2.518777000000
C	5.119991000000	-0.839790000000	1.788146000000
C	3.777385000000	-0.869689000000	1.425548000000
H	1.353266000000	-1.979008000000	1.614052000000
H	0.956216000000	1.040613000000	1.727993000000
H	-1.725788000000	-2.161145000000	4.194908000000
H	-2.429148000000	-4.492500000000	4.658536000000
H	-2.161771000000	-6.242532000000	2.920089000000
H	-1.126846000000	-5.663575000000	0.736747000000
H	-0.382698000000	-3.326236000000	0.300898000000
H	-2.541910000000	-0.685564000000	-0.819770000000
H	-3.055718000000	-2.562046000000	-2.346183000000
H	-4.981828000000	-4.651084000000	0.867927000000
H	-4.444057000000	-2.758670000000	2.407729000000
H	-4.238948000000	-4.627136000000	-2.804098000000
H	-3.572741000000	-5.705785000000	-1.574490000000
H	-5.299141000000	-5.343676000000	-1.578044000000
H	2.772514000000	2.069066000000	2.774314000000
H	5.148680000000	2.112993000000	3.460027000000
H	6.666851000000	0.260547000000	2.805830000000
H	5.769424000000	-1.659131000000	1.494090000000
H	3.397984000000	-1.719652000000	0.865326000000

----

^{SS}INTb6

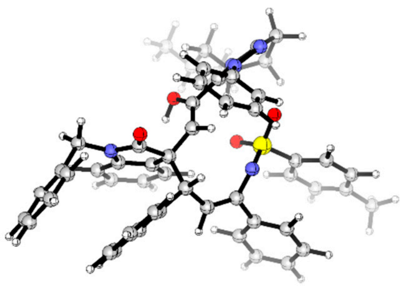

Zero-point correction= 0.950549 (Hartree/Particle)

Thermal correction to Energy= 1.005593

Thermal correction to Enthalpy= 1.006537

Thermal correction to Gibbs Free Energy= 0.858502

Sum of electronic and zero-point Energies= -3059.236643

Sum of electronic and thermal Energies= -3059.181599

Sum of electronic and thermal Enthalpies= -3059.180655

Sum of electronic and thermal Free Energies= -3059.328689

E(RM062X) = -3060.85546216

O	-0.057198000000	-2.438811000000	-1.199471000000
H	0.269217000000	-1.717737000000	-1.761022000000
C	-0.114478000000	-1.996526000000	0.089223000000
C	0.557792000000	-0.995439000000	0.666668000000
C	1.633594000000	-0.146956000000	0.053975000000
C	1.649437000000	-0.035928000000	-1.449608000000
C	2.885715000000	-0.499700000000	-1.926859000000
C	3.207740000000	-0.446534000000	-3.274578000000
C	2.245118000000	0.065900000000	-4.151091000000
C	0.692162000000	0.453083000000	-2.327936000000
C	1.003208000000	0.497061000000	-3.694899000000
N	3.641552000000	-0.999244000000	-0.863285000000
C	2.975192000000	-0.866077000000	0.335964000000
O	3.381080000000	-1.265129000000	1.407600000000
C	4.940323000000	-1.642268000000	-0.950590000000
C	6.107582000000	-0.709344000000	-0.715333000000
C	6.742576000000	-0.067924000000	-1.779437000000
C	7.850920000000	0.743327000000	-1.555100000000
C	8.338458000000	0.910226000000	-0.261402000000
C	7.700060000000	0.284541000000	0.806145000000
C	6.584025000000	-0.515520000000	0.582092000000
H	2.473911000000	0.115175000000	-5.210939000000
H	4.167146000000	-0.791534000000	-3.643962000000
H	-0.280101000000	0.760132000000	-1.949212000000
H	0.269370000000	0.873053000000	-4.399586000000
H	6.378103000000	-0.216174000000	-2.793061000000
H	8.341965000000	1.232598000000	-2.390399000000
H	9.212170000000	1.530248000000	-0.085003000000
H	8.070825000000	0.421971000000	1.817352000000
H	6.066728000000	-0.994954000000	1.409033000000
H	5.014625000000	-2.115363000000	-1.934766000000
H	4.942960000000	-2.430425000000	-0.192204000000
C	-1.179757000000	-2.684356000000	0.815407000000
N	-1.305041000000	-2.943044000000	2.123871000000
N	-2.539204000000	-3.442422000000	2.433204000000
C	-3.130215000000	-3.519867000000	1.278803000000
N	-2.318228000000	-3.109014000000	0.265232000000
C	-4.496597000000	-3.812326000000	0.755967000000
C	-4.470739000000	-2.984940000000	-0.557204000000
C	-2.995273000000	-2.978307000000	-1.039423000000
C	-2.602147000000	-4.116409000000	-1.987344000000
C	-2.634930000000	-3.688697000000	-3.458750000000
C	-0.425124000000	-2.502265000000	3.165898000000
C	0.842418000000	-3.057590000000	3.275643000000
C	1.716943000000	-2.530991000000	4.219566000000
C	1.308173000000	-1.479836000000	5.037025000000
C	0.028238000000	-0.941983000000	4.911919000000
C	-0.853757000000	-1.444613000000	3.960617000000
C	-2.197735000000	-4.847795000000	-4.352429000000
C	-4.005185000000	-3.162953000000	-3.887636000000
H	-4.618624000000	-4.884047000000	0.568962000000
H	-5.271308000000	-3.487480000000	1.449926000000
H	-4.760397000000	-1.955270000000	-0.328070000000
H	-5.142428000000	-3.387477000000	-1.316460000000
H	-2.712408000000	-2.003633000000	-1.443805000000
H	-3.265061000000	-4.981299000000	-1.834133000000
H	-1.584403000000	-4.436712000000	-1.745868000000

H	-1.905551000000	-2.872986000000	-3.564157000000
H	1.144978000000	-3.859608000000	2.610755000000
H	2.721256000000	-2.930391000000	4.301637000000
H	1.997712000000	-1.067509000000	5.766258000000
H	-0.276288000000	-0.108468000000	5.535275000000
H	-1.829308000000	-1.007076000000	3.773095000000
H	-2.158322000000	-4.541795000000	-5.401744000000
H	-1.208282000000	-5.217413000000	-4.067802000000
H	-2.905125000000	-5.681409000000	-4.274014000000
H	-3.997903000000	-2.885883000000	-4.945760000000
H	-4.303501000000	-2.277085000000	-3.318238000000
H	-4.774094000000	-3.933424000000	-3.751365000000
H	0.315963000000	-0.758908000000	1.694298000000
C	0.657301000000	2.263344000000	0.245647000000
C	-0.672857000000	2.237529000000	0.458994000000
C	1.678864000000	1.279148000000	0.760167000000
N	-1.298278000000	1.264091000000	1.246609000000
C	-1.494032000000	3.373909000000	-0.063278000000
C	-1.401959000000	3.793689000000	-1.391540000000
C	-2.199179000000	4.836820000000	-1.860610000000
C	-3.092712000000	5.472863000000	-1.004846000000
C	-3.184061000000	5.064637000000	0.326333000000
C	-2.391730000000	4.024100000000	0.791368000000
C	-3.965198000000	1.414831000000	0.400681000000
C	-4.708549000000	1.742027000000	1.529638000000
C	-5.793114000000	2.603626000000	1.404046000000
C	-6.142178000000	3.142530000000	0.163141000000
C	-5.397746000000	2.774385000000	-0.961211000000
C	-4.312777000000	1.914392000000	-0.850047000000
C	-7.272902000000	4.129990000000	0.033813000000
S	-2.471342000000	0.438649000000	0.593668000000
O	-2.860262000000	-0.630763000000	1.540867000000
O	-2.172681000000	-0.018506000000	-0.793623000000
C	3.072981000000	1.872933000000	0.659220000000
C	3.914399000000	1.885821000000	1.773467000000
C	5.193008000000	2.427764000000	1.693661000000
C	5.659395000000	2.944356000000	0.488386000000
C	4.838303000000	2.916371000000	-0.634753000000
C	3.550647000000	2.395824000000	-0.547587000000
H	1.053992000000	3.130768000000	-0.273338000000
H	1.474841000000	1.062155000000	1.816870000000
H	-0.712717000000	3.282396000000	-2.059202000000
H	-2.126151000000	5.146973000000	-2.898794000000
H	-3.719125000000	6.280406000000	-1.371912000000
H	-3.882922000000	5.552174000000	1.000007000000
H	-2.469821000000	3.680702000000	1.818472000000
H	-4.426264000000	1.326073000000	2.492224000000
H	-6.375302000000	2.869347000000	2.282691000000
H	-5.660858000000	3.185308000000	-1.932873000000
H	-3.713486000000	1.641395000000	-1.711739000000
H	-7.900117000000	4.139371000000	0.928062000000
H	-6.880852000000	5.142597000000	-0.111878000000
H	-7.906066000000	3.898378000000	-0.827050000000
H	3.558580000000	1.460571000000	2.707907000000
H	5.831125000000	2.435537000000	2.572318000000
H	6.662932000000	3.351871000000	0.419888000000
H	5.199267000000	3.305860000000	-1.581730000000
H	2.909629000000	2.394407000000	-1.425909000000

INTb7

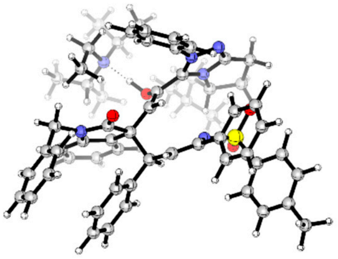

Zero-point correction= 1.217483 (Hartree/Particle)

Thermal correction to Energy= 1.285058

Thermal correction to Enthalpy= 1.286003

Thermal correction to Gibbs Free Energy= 1.112479

Sum of electronic and zero-point Energies= -3429.864618

Sum of electronic and thermal Energies= -3429.797043

Sum of electronic and thermal Enthalpies= -3429.796098

Sum of electronic and thermal Free Energies= -3429.969623

E(RM062X) = -3431.83224430

O	-1.061961000000	2.311329000000	-0.463813000000
H	-1.947545000000	2.617369000000	-0.039816000000
C	-0.383150000000	1.525951000000	0.408020000000
C	-0.459459000000	0.202102000000	0.569755000000
C	-1.221989000000	-0.826834000000	-0.199127000000
C	-2.255570000000	-0.403618000000	-1.208265000000
C	-3.474871000000	-1.017179000000	-0.888198000000
C	-4.595065000000	-0.885355000000	-1.697505000000
C	-4.457711000000	-0.145582000000	-2.876363000000
C	-2.129683000000	0.313307000000	-2.385487000000
C	-3.241969000000	0.438330000000	-3.224628000000
N	-3.344115000000	-1.718423000000	0.314965000000
C	-2.057276000000	-1.642851000000	0.801996000000
O	-1.687974000000	-2.122075000000	1.856132000000
C	-0.133518000000	-1.768066000000	-0.927064000000
C	1.014689000000	-2.147899000000	-0.042295000000
C	2.133142000000	-1.380858000000	0.034444000000
N	2.188388000000	-0.179774000000	-0.655612000000
S	3.534091000000	0.555481000000	-1.053029000000
O	3.293582000000	1.303446000000	-2.302511000000
O	4.102375000000	1.398094000000	0.032351000000
C	4.768266000000	-0.696513000000	-1.389344000000
C	4.483918000000	-1.692867000000	-2.317889000000
C	5.387674000000	-2.735448000000	-2.489087000000
C	6.566427000000	-2.795725000000	-1.739954000000
C	6.836190000000	-1.772032000000	-0.828416000000
C	5.942078000000	-0.723606000000	-0.646474000000
C	7.510087000000	-3.962238000000	-1.878997000000
C	-4.399081000000	-2.381257000000	1.059585000000
C	-4.445544000000	-3.877855000000	0.844840000000
C	-5.216653000000	-4.431748000000	-0.176154000000
C	-5.265078000000	-5.811387000000	-0.354986000000
C	-4.546271000000	-6.648429000000	0.494357000000
C	-3.761163000000	-6.100253000000	1.507096000000
C	-3.706433000000	-4.719968000000	1.678635000000
C	-0.822896000000	-2.948336000000	-1.580817000000
C	-1.237881000000	-4.056394000000	-0.835278000000
C	-1.913640000000	-5.109682000000	-1.443276000000
C	-2.177461000000	-5.077955000000	-2.809839000000
C	-1.758615000000	-3.986465000000	-3.565236000000
C	-1.089443000000	-2.930997000000	-2.952475000000
C	3.231061000000	-1.829029000000	0.945799000000
C	3.733889000000	-3.130562000000	0.871166000000
C	4.760950000000	-3.541795000000	1.715762000000
C	5.291675000000	-2.659692000000	2.653835000000
C	4.790956000000	-1.361953000000	2.738920000000
C	3.773264000000	-0.946545000000	1.886936000000
H	-5.313438000000	-0.042019000000	-3.536449000000
H	-5.537776000000	-1.360278000000	-1.446487000000
H	-1.181330000000	0.780259000000	-2.636476000000

H	-3.156570000000	0.994329000000	-4.152517000000
H	0.270320000000	-1.109601000000	-1.704699000000
H	3.554418000000	-1.657390000000	-2.878837000000
H	5.171735000000	-3.522886000000	-3.206481000000
H	7.749061000000	-1.808223000000	-0.239254000000
H	6.118693000000	0.058319000000	0.084161000000
H	7.389141000000	-4.459741000000	-2.844164000000
H	8.551437000000	-3.643482000000	-1.784980000000
H	7.321797000000	-4.704840000000	-1.095888000000
H	-5.787579000000	-3.780705000000	-0.833791000000
H	-5.870811000000	-6.232628000000	-1.151084000000
H	-4.592110000000	-7.725063000000	0.363350000000
H	-3.190002000000	-6.748473000000	2.164324000000
H	-3.083188000000	-4.278644000000	2.451689000000
H	-5.349625000000	-1.918017000000	0.775760000000
H	-4.214872000000	-2.166619000000	2.117097000000
H	-1.048405000000	-4.089052000000	0.234593000000
H	-2.244016000000	-5.949953000000	-0.841240000000
H	-2.707041000000	-5.899798000000	-3.282205000000
H	-1.956040000000	-3.952216000000	-4.632322000000
H	-0.773190000000	-2.073721000000	-3.540901000000
H	3.332057000000	-3.803791000000	0.119799000000
H	5.155852000000	-4.550081000000	1.632545000000
H	6.097506000000	-2.978421000000	3.307932000000
H	5.206564000000	-0.664230000000	3.460922000000
H	3.423239000000	0.080831000000	1.907275000000
C	0.733067000000	2.207368000000	1.090865000000
N	1.222032000000	2.040987000000	2.330449000000
N	2.394629000000	2.726194000000	2.521661000000
C	2.585556000000	3.322401000000	1.380312000000
N	1.573629000000	3.065992000000	0.505488000000
C	3.639549000000	4.164322000000	0.748257000000
C	3.240638000000	4.121408000000	-0.754521000000
C	1.771104000000	3.613783000000	-0.849712000000
C	0.741999000000	4.698846000000	-1.190939000000
C	0.396189000000	4.695512000000	-2.683986000000
C	0.650610000000	1.289172000000	3.405053000000
C	-0.719553000000	1.366480000000	3.638195000000
C	-1.271108000000	0.608316000000	4.661875000000
C	-0.455098000000	-0.198000000000	5.453156000000
C	0.916154000000	-0.242982000000	5.221870000000
C	1.481765000000	0.500568000000	4.190599000000
C	-0.583112000000	5.822846000000	-3.003317000000
C	1.639655000000	4.787048000000	-3.570655000000
H	3.626359000000	5.174771000000	1.167135000000
H	4.617901000000	3.713244000000	0.912977000000
H	3.883181000000	3.414164000000	-1.277779000000
H	3.327599000000	5.105388000000	-1.218592000000
H	1.703393000000	2.769450000000	-1.543570000000
H	1.130776000000	5.684084000000	-0.894753000000
H	-0.175167000000	4.518751000000	-0.624531000000
H	-0.097342000000	3.732978000000	-2.883726000000
H	-1.339075000000	2.003949000000	3.015189000000
H	-2.339584000000	0.651843000000	4.844296000000
H	-0.890783000000	-0.792039000000	6.249265000000
H	1.553194000000	-0.871475000000	5.834246000000
H	2.544706000000	0.465536000000	3.984033000000
H	-0.899375000000	5.786628000000	-4.049960000000
H	-1.481391000000	5.765381000000	-2.378628000000
H	-0.115189000000	6.798618000000	-2.828921000000
H	1.357197000000	4.786828000000	-4.627453000000
H	2.322169000000	3.946019000000	-3.410134000000
H	2.185399000000	5.718074000000	-3.373307000000
H	0.935246000000	-3.028053000000	0.585906000000
N	-3.340825000000	3.276355000000	0.423666000000
C	-3.897508000000	2.714327000000	1.663527000000

C	-4.122047000000	2.887120000000	-0.790861000000
C	-2.948952000000	4.702808000000	0.542044000000
C	-3.923254000000	1.191364000000	1.659166000000
H	-4.903674000000	3.108158000000	1.866579000000
H	-3.265983000000	3.047443000000	2.491341000000
H	-4.237371000000	0.821861000000	2.639625000000
H	-4.610049000000	0.787405000000	0.909784000000
H	-2.923275000000	0.796832000000	1.449621000000
C	-3.592106000000	3.578906000000	-2.043591000000
C	-5.643640000000	3.033278000000	-0.691220000000
H	-3.913015000000	1.817771000000	-0.919659000000
H	-2.508435000000	3.451414000000	-2.140048000000
H	-4.059711000000	3.118376000000	-2.916544000000
H	-3.833042000000	4.647363000000	-2.060638000000
H	-6.062703000000	2.472020000000	0.147934000000
H	-5.965868000000	4.073751000000	-0.602563000000
H	-6.083616000000	2.622109000000	-1.604491000000
C	-1.882607000000	4.883481000000	1.626926000000
C	-4.100741000000	5.688472000000	0.753084000000
H	-2.460653000000	4.945759000000	-0.407122000000
H	-1.130278000000	4.088961000000	1.570557000000
H	-1.371913000000	5.838288000000	1.475628000000
H	-2.306119000000	4.896799000000	2.635324000000
H	-4.760564000000	5.735993000000	-0.115312000000
H	-4.699053000000	5.419243000000	1.629364000000
H	-3.699819000000	6.692339000000	0.921047000000
H	0.205393000000	-0.247996000000	1.302219000000

INTb7''

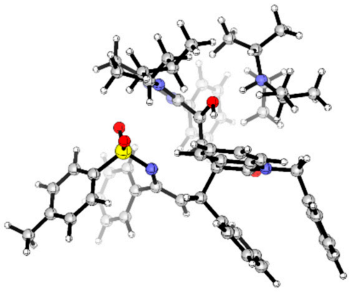

Zero-point correction= 1.231915 (Hartree/Particle)

Thermal correction to Energy= 1.300181

Thermal correction to Enthalpy= 1.301125

Thermal correction to Gibbs Free Energy= 1.123910

Sum of electronic and zero-point Energies= -3430.249282

Sum of electronic and thermal Energies= -3430.181016

Sum of electronic and thermal Enthalpies= -3430.180071

Sum of electronic and thermal Free Energies= -3430.357287

E(RM062X) = -3432.22739953

N	3.016139000000	-3.161983000000	0.782871000000
H	2.175947000000	-2.678976000000	0.428416000000
C	2.967827000000	-3.106462000000	2.286909000000
C	4.141894000000	-2.306299000000	0.194531000000
C	2.842049000000	-4.561946000000	0.211441000000
C	2.820161000000	-1.692951000000	2.819869000000
H	3.865891000000	-3.602002000000	2.659264000000
H	2.099965000000	-3.692531000000	2.591675000000
H	2.703066000000	-1.749981000000	3.904203000000
H	3.672329000000	-1.046305000000	2.604570000000
H	1.925140000000	-1.206906000000	2.420006000000
C	4.234102000000	-2.479187000000	-1.316412000000
C	5.487439000000	-2.495605000000	0.883187000000
H	3.788300000000	-1.289727000000	0.396120000000
H	3.264387000000	-2.366994000000	-1.808763000000
H	4.882343000000	-1.691672000000	-1.707709000000
H	4.673917000000	-3.440102000000	-1.598185000000

H	5.455409000000	-2.248825000000	1.945664000000
H	5.887780000000	-3.503684000000	0.766002000000
H	6.193327000000	-1.805173000000	0.414070000000
C	1.518107000000	-5.151211000000	0.695058000000
C	4.014336000000	-5.486955000000	0.499636000000
H	2.757165000000	-4.393642000000	-0.864698000000
H	0.707570000000	-4.413670000000	0.658962000000
H	1.241741000000	-5.972149000000	0.029662000000
H	1.590088000000	-5.556222000000	1.707586000000
H	4.925658000000	-5.184033000000	-0.016626000000
H	4.218883000000	-5.566175000000	1.570824000000
H	3.753544000000	-6.486334000000	0.143273000000
C	0.185655000000	-0.121354000000	0.412427000000
C	0.149746000000	-1.428727000000	0.133763000000
O	0.921863000000	-2.095792000000	-0.808157000000
C	1.008479000000	0.938744000000	-0.247263000000
C	1.426022000000	0.635509000000	-1.659493000000
C	2.817839000000	0.765906000000	-1.767037000000
C	3.460579000000	0.611488000000	-2.986952000000
C	2.670785000000	0.292533000000	-4.098797000000
C	0.641822000000	0.339251000000	-2.767033000000
C	1.288672000000	0.149255000000	-3.997834000000
N	3.354546000000	1.019397000000	-0.497583000000
C	2.363900000000	1.069497000000	0.466542000000
O	2.569833000000	1.152012000000	1.661286000000
C	0.222583000000	2.372942000000	-0.258578000000
C	-0.872295000000	2.496264000000	0.752084000000
C	-2.039676000000	1.836157000000	0.507821000000
N	-2.050680000000	0.999393000000	-0.590170000000
S	-3.292772000000	0.146748000000	-1.090257000000
O	-2.963492000000	-0.301943000000	-2.459550000000
O	-3.663401000000	-0.965796000000	-0.175387000000
C	-4.707069000000	1.226198000000	-1.168414000000

C	-4.625940000000	2.376778000000	-1.946424000000
C	-5.676463000000	3.286341000000	-1.914679000000
C	-6.797943000000	3.063097000000	-1.110805000000
C	-6.857818000000	1.890967000000	-0.351049000000
C	-5.819368000000	0.969025000000	-0.375050000000
C	-7.912471000000	4.073250000000	-1.035210000000
C	4.764405000000	1.129057000000	-0.154377000000
C	5.290063000000	2.540036000000	-0.028571000000
C	5.775958000000	3.224997000000	-1.142704000000
C	6.323958000000	4.495781000000	-1.000938000000
C	6.396714000000	5.084969000000	0.259350000000
C	5.899039000000	4.411908000000	1.371265000000
C	5.341026000000	3.144791000000	1.226844000000
C	1.220941000000	3.506442000000	-0.287709000000
C	1.714863000000	4.088215000000	0.882853000000
C	2.633263000000	5.130906000000	0.818471000000
C	3.083078000000	5.595683000000	-0.414540000000
C	2.612939000000	5.009232000000	-1.585547000000
C	1.684373000000	3.974826000000	-1.520894000000
C	-3.205532000000	2.025616000000	1.426604000000
C	-3.768284000000	3.296950000000	1.567736000000
C	-4.857021000000	3.496833000000	2.411459000000
C	-5.383407000000	2.431882000000	3.137873000000
C	-4.821290000000	1.163709000000	3.011192000000
C	-3.745946000000	0.957666000000	2.152912000000
H	3.151600000000	0.163534000000	-5.062799000000
H	4.533761000000	0.736884000000	-3.089230000000
H	-0.444323000000	0.268427000000	-2.664822000000
H	0.703601000000	-0.091016000000	-4.878927000000
H	-0.274911000000	2.331463000000	-1.232192000000
H	-3.744383000000	2.559930000000	-2.553515000000
H	-5.623599000000	4.189620000000	-2.515673000000
H	-7.724021000000	1.706263000000	0.278889000000

H	-5.840185000000	0.071960000000	0.233707000000
H	-7.816584000000	4.832196000000	-1.814196000000
H	-8.888610000000	3.593052000000	-1.142698000000
H	-7.903010000000	4.582800000000	-0.066069000000
H	5.736292000000	2.759040000000	-2.124674000000
H	6.706108000000	5.019966000000	-1.870931000000
H	6.840075000000	6.069095000000	0.373105000000
H	5.948346000000	4.871766000000	2.353191000000
H	4.936801000000	2.616681000000	2.086546000000
H	5.329930000000	0.577374000000	-0.914541000000
H	4.892695000000	0.617947000000	0.807378000000
H	1.383758000000	3.716647000000	1.848041000000
H	3.003787000000	5.579475000000	1.734952000000
H	3.803463000000	6.405788000000	-0.461187000000
H	2.959905000000	5.365396000000	-2.550535000000
H	1.300702000000	3.530112000000	-2.436658000000
H	-3.355438000000	4.120284000000	0.992741000000
H	-5.294804000000	4.486073000000	2.501347000000
H	-6.229864000000	2.589044000000	3.798818000000
H	-5.228963000000	0.328408000000	3.572224000000
H	-3.353648000000	-0.044883000000	2.007240000000
C	-0.953002000000	-2.256930000000	0.614981000000
N	-1.464979000000	-2.402855000000	1.847062000000
N	-2.605056000000	-3.163445000000	1.837103000000
C	-2.762418000000	-3.462617000000	0.578837000000
N	-1.743581000000	-2.976236000000	-0.181504000000
C	-3.807176000000	-4.088611000000	-0.282501000000
C	-3.424524000000	-3.552638000000	-1.692394000000
C	-1.935075000000	-3.110687000000	-1.637145000000
C	-0.931998000000	-4.087470000000	-2.268426000000
C	-0.479809000000	-3.604866000000	-3.651317000000
C	-0.857705000000	-2.058493000000	3.105176000000
C	-0.331304000000	-3.107499000000	3.856694000000
C	0.252200000000	-2.831410000000	5.087940000000
C	0.305805000000	-1.516852000000	5.552245000000
C	-0.236968000000	-0.482938000000	4.795299000000
C	-0.839319000000	-0.747344000000	3.566581000000
C	0.521946000000	-4.585263000000	-4.255929000000
C	-1.665482000000	-3.371467000000	-4.589054000000
H	-3.760609000000	-5.179371000000	-0.214499000000
H	-4.799243000000	-3.759876000000	0.027169000000
H	-4.027645000000	-2.674833000000	-1.923205000000
H	-3.573164000000	-4.306504000000	-2.466836000000
H	-1.831013000000	-2.106961000000	-2.063055000000
H	-1.381352000000	-5.086698000000	-2.350097000000
H	-0.050669000000	-4.178844000000	-1.625688000000
H	0.027390000000	-2.638253000000	-3.501263000000
H	-0.413651000000	-4.124232000000	3.482643000000
H	0.651504000000	-3.640938000000	5.689922000000
H	0.758173000000	-1.302609000000	6.514825000000
H	-0.212487000000	0.536402000000	5.163527000000
H	-1.309279000000	0.053668000000	3.004910000000
H	0.899995000000	-4.221436000000	-5.214873000000
H	1.379727000000	-4.740748000000	-3.591252000000
H	0.052334000000	-5.559486000000	-4.429979000000
H	-1.319775000000	-3.032745000000	-5.569508000000
H	-2.352525000000	-2.611552000000	-4.201465000000
H	-2.225810000000	-4.302144000000	-4.738607000000
H	-0.783938000000	3.162696000000	1.601237000000
H	-0.491105000000	0.243861000000	1.172554000000
H	1.089817000000	-1.484845000000	-1.553201000000

TSb4

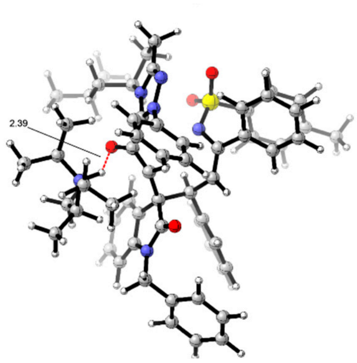

Frequency -42.5761

Zero-point correction= 1.218449 (Hartree/Particle)

Thermal correction to Energy= 1.285339

Thermal correction to Enthalpy= 1.286283

Thermal correction to Gibbs Free Energy= 1.114529

Sum of electronic and zero-point Energies= -3429.840548

Sum of electronic and thermal Energies= -3429.773657

Sum of electronic and thermal Enthalpies= -3429.772713

Sum of electronic and thermal Free Energies= -3429.944468

E(RM062X) = -3431.81139464

N	-2.449860000000	3.453271000000	0.835727000000
H	-1.693056000000	2.745380000000	0.683660000000
C	-2.705429000000	3.420626000000	2.314657000000
C	-3.611066000000	2.879861000000	0.017160000000
C	-1.885753000000	4.773153000000	0.336638000000
C	-2.925077000000	2.009857000000	2.838039000000
H	-3.551039000000	4.079397000000	2.521574000000
H	-1.818206000000	3.836334000000	2.794392000000
H	-3.060977000000	2.061199000000	3.920783000000
H	-3.806029000000	1.526177000000	2.408624000000
H	-2.059751000000	1.372726000000	2.635642000000
C	-3.416156000000	3.142534000000	-1.469476000000
C	-4.989307000000	3.311509000000	0.506068000000
H	-3.508568000000	1.803035000000	0.184363000000
H	-2.392452000000	2.909875000000	-1.773492000000
H	-4.087862000000	2.477877000000	-2.018980000000
H	-3.665608000000	4.174434000000	-1.736364000000
H	-5.202689000000	2.996809000000	1.529448000000
H	-5.147663000000	4.389072000000	0.430310000000
H	-5.721149000000	2.825061000000	-0.144399000000
C	-0.590261000000	5.113231000000	1.064881000000
C	-2.883080000000	5.919965000000	0.428224000000
H	-1.624259000000	4.562022000000	-0.700106000000
H	0.063513000000	4.242310000000	1.147090000000
H	-0.058816000000	5.866519000000	0.478335000000
H	-0.765701000000	5.528301000000	2.061830000000
H	-3.736303000000	5.791568000000	-0.238142000000
H	-3.246841000000	6.067032000000	1.449602000000
H	-2.369918000000	6.837915000000	0.130409000000
C	-0.637730000000	0.818867000000	0.383720000000
C	0.026299000000	1.879818000000	-0.179665000000
O	-0.232514000000	2.594076000000	-1.199489000000
C	-1.464533000000	-0.233686000000	-0.303364000000
C	-2.272783000000	0.079850000000	-1.533996000000
C	-3.598020000000	-0.325833000000	-1.322263000000
C	-4.552934000000	-0.276085000000	-2.327192000000
C	-4.142717000000	0.186196000000	-3.582415000000
C	-1.878855000000	0.526041000000	-2.783353000000
C	-2.827780000000	0.584212000000	-3.809741000000
N	-3.747126000000	-0.745619000000	0.009599000000
C	-2.536390000000	-0.728577000000	0.671820000000
O	-2.394383000000	-1.030875000000	1.845360000000
C	-0.459947000000	-1.429666000000	-0.675766000000
C	0.444853000000	-1.856346000000	0.441362000000
C	1.735648000000	-1.439512000000	0.517558000000
N	2.190204000000	-0.425832000000	-0.315240000000
S	3.643282000000	-0.452401000000	-0.963628000000
O	3.541516000000	0.083347000000	-2.335898000000

O	4.685653000000	0.211521000000	-0.143632000000
C	4.121113000000	-2.172665000000	-1.084889000000
C	3.272916000000	-3.050544000000	-1.753248000000
C	3.565328000000	-4.409289000000	-1.751169000000
C	4.693353000000	-4.898908000000	-1.086202000000
C	5.535147000000	-3.994538000000	-0.434208000000
C	5.252261000000	-2.632875000000	-0.423115000000
C	4.975587000000	-6.378572000000	-1.034082000000
C	-4.980660000000	-1.135592000000	0.667996000000
C	-5.234730000000	-2.627014000000	0.676595000000
C	-6.010376000000	-3.230508000000	-0.311694000000
C	-6.252202000000	-4.601482000000	-0.281478000000
C	-5.722567000000	-5.378455000000	0.744641000000
C	-4.935673000000	-4.783297000000	1.729674000000
C	-4.690724000000	-3.413870000000	1.694926000000
C	-1.196583000000	-2.600848000000	-1.295552000000
C	-1.965374000000	-3.474786000000	-0.520358000000
C	-2.662027000000	-4.525715000000	-1.109284000000
C	-2.593725000000	-4.727113000000	-2.485279000000
C	-1.823937000000	-3.870445000000	-3.267290000000
C	-1.134978000000	-2.816095000000	-2.675227000000
C	2.631693000000	-2.054873000000	1.545370000000
C	2.605118000000	-3.435534000000	1.771209000000
C	3.461128000000	-4.017435000000	2.699597000000
C	4.354116000000	-3.225907000000	3.419067000000
C	4.385767000000	-1.850205000000	3.201989000000
C	3.537182000000	-1.266809000000	2.266453000000
H	-4.865092000000	0.226989000000	-4.391603000000
H	-5.573749000000	-0.604194000000	-2.161541000000
H	-0.856138000000	0.858637000000	-2.936792000000
H	-2.535401000000	0.938161000000	-4.792577000000
H	0.185148000000	-0.971851000000	-1.435439000000
H	2.381753000000	-2.670262000000	-2.245751000000

H	2.902306000000	-5.102610000000	-2.262184000000
H	6.413637000000	-4.365564000000	0.087953000000
H	5.873690000000	-1.922041000000	0.111760000000
H	6.048579000000	-6.580284000000	-1.088590000000
H	4.607898000000	-6.808227000000	-0.095790000000
H	4.484781000000	-6.906559000000	-1.855050000000
H	-6.435852000000	-2.624994000000	-1.108020000000
H	-6.859406000000	-5.060562000000	-1.055098000000
H	-5.917621000000	-6.445816000000	0.774930000000
H	-4.514250000000	-5.386775000000	2.527534000000
H	-4.067702000000	-2.939804000000	2.449051000000
H	-5.804703000000	-0.606349000000	0.178024000000
H	-4.904946000000	-0.777421000000	1.699987000000
H	-2.028368000000	-3.327624000000	0.555383000000
H	-3.264444000000	-5.181390000000	-0.488362000000
H	-3.136493000000	-5.547458000000	-2.944989000000
H	-1.761143000000	-4.019501000000	-4.340999000000
H	-0.542999000000	-2.141269000000	-3.288346000000
H	1.938019000000	-4.049465000000	1.173040000000
H	3.444236000000	-5.093050000000	2.849450000000
H	5.029748000000	-3.679854000000	4.137800000000
H	5.082134000000	-1.227624000000	3.755515000000
H	3.591157000000	-0.201242000000	2.062095000000
C	1.322695000000	2.226719000000	0.526784000000
N	1.744480000000	2.136357000000	1.802729000000
N	3.069294000000	2.479512000000	1.942918000000
C	3.432357000000	2.773538000000	0.728548000000
N	2.390335000000	2.671159000000	-0.141243000000
C	4.681837000000	3.183736000000	0.026751000000
C	4.283596000000	3.011412000000	-1.465681000000
C	2.729786000000	2.975086000000	-1.543714000000
C	2.075118000000	4.286406000000	-1.998376000000
C	1.677893000000	4.238561000000	-3.475382000000

C	0.994363000000	1.866591000000	2.998359000000
C	0.595754000000	2.950853000000	3.775676000000
C	-0.105427000000	2.720606000000	4.956066000000
C	-0.391632000000	1.414750000000	5.349984000000
C	0.035557000000	0.339559000000	4.574584000000
C	0.746348000000	0.557733000000	3.397964000000
C	0.989418000000	5.540235000000	-3.879148000000
C	2.872165000000	3.945236000000	-4.384196000000
H	4.940148000000	4.215183000000	0.285461000000
H	5.500173000000	2.517795000000	0.300132000000
H	4.669979000000	2.059459000000	-1.829870000000
H	4.674325000000	3.822562000000	-2.083143000000
H	2.392981000000	2.124654000000	-2.143153000000
H	2.762530000000	5.126835000000	-1.818404000000
H	1.168392000000	4.447773000000	-1.410270000000
H	0.953085000000	3.418586000000	-3.567123000000
H	0.860225000000	3.956628000000	3.463203000000
H	-0.413507000000	3.559505000000	5.572110000000
H	-0.938596000000	1.234772000000	6.269974000000
H	-0.179507000000	-0.678242000000	4.879986000000
H	1.088754000000	-0.273667000000	2.789157000000
H	0.653893000000	5.504661000000	-4.919876000000
H	0.115786000000	5.736033000000	-3.248109000000
H	1.676184000000	6.389164000000	-3.779006000000
H	2.563944000000	3.914883000000	-5.433630000000
H	3.339478000000	2.983225000000	-4.148698000000
H	3.633733000000	4.728734000000	-4.283353000000
H	0.067236000000	-2.550773000000	1.185986000000
H	-0.202420000000	0.397141000000	1.275180000000

TSb4'

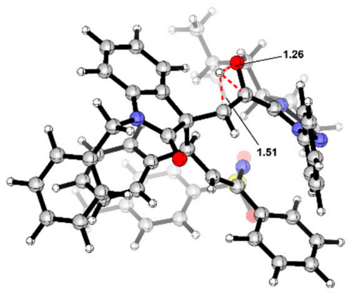

Frequency -2301.8783

Zero-point correction= 0.945192 (Hartree/Particle)

Thermal correction to Energy= 0.999248

Thermal correction to Enthalpy= 1.000192

Thermal correction to Gibbs Free Energy= 0.857287

Sum of electronic and zero-point Energies= -3059.135560

Sum of electronic and thermal Energies= -3059.081504

Sum of electronic and thermal Enthalpies= -3059.080560

Sum of electronic and thermal Free Energies= -3059.223465

E(RM062X) = -3060.74448107

O	1.213390000000	0.296399000000	-3.534242000000
H	0.247748000000	1.095023000000	-3.373756000000
C	1.421541000000	0.804969000000	-2.372595000000
C	0.311052000000	1.555875000000	-1.937757000000
C	-0.942000000000	0.794267000000	-1.445073000000
C	-1.669728000000	-0.117261000000	-2.407307000000
C	-2.990769000000	0.331609000000	-2.543450000000
C	-3.929721000000	-0.367485000000	-3.285847000000
C	-3.515868000000	-1.546298000000	-3.910763000000
C	-1.278511000000	-1.289544000000	-3.029753000000
C	-2.207815000000	-2.004610000000	-3.792040000000
N	-3.156489000000	1.525787000000	-1.833018000000
C	-1.999922000000	1.888547000000	-1.192390000000
O	-1.851525000000	2.911098000000	-0.550939000000
C	-0.626204000000	0.028411000000	-0.091911000000
C	0.184572000000	0.875248000000	0.845589000000

C	1.349480000000	0.470677000000	1.404766000000
N	1.968065000000	-0.718512000000	0.989144000000
S	2.152674000000	-1.916454000000	2.022778000000
O	2.808781000000	-3.014872000000	1.276142000000
O	2.769444000000	-1.544646000000	3.301964000000
C	0.519230000000	-2.512382000000	2.441589000000
C	0.048025000000	-3.707353000000	1.908937000000
C	-1.221745000000	-4.153313000000	2.260720000000
C	-2.031372000000	-3.412516000000	3.125178000000
C	-1.542946000000	-2.205334000000	3.631788000000
C	-0.270135000000	-1.755160000000	3.303706000000
C	-3.395487000000	-3.913443000000	3.522328000000
C	-4.336020000000	2.375582000000	-1.809344000000
C	-5.173693000000	2.227995000000	-0.558537000000
C	-6.200843000000	1.287976000000	-0.488642000000
C	-6.987656000000	1.186636000000	0.655392000000
C	-6.752488000000	2.029602000000	1.738531000000
C	-5.714250000000	2.957456000000	1.682204000000
C	-4.925612000000	3.053889000000	0.539834000000
C	-1.926909000000	-0.492173000000	0.503922000000
C	-2.714275000000	0.298719000000	1.347289000000
C	-3.953507000000	-0.153071000000	1.789980000000
C	-4.432117000000	-1.396982000000	1.386479000000
C	-3.648965000000	-2.199641000000	0.562235000000
C	-2.402786000000	-1.752497000000	0.135374000000
C	2.077119000000	1.389738000000	2.341131000000
C	3.445212000000	1.212567000000	2.582519000000
C	4.163516000000	2.125393000000	3.348104000000
C	3.529423000000	3.237095000000	3.897593000000
C	2.159825000000	3.404988000000	3.700945000000
C	1.442223000000	2.485480000000	2.943685000000
H	-4.233007000000	-2.113256000000	-4.495461000000
H	-4.954604000000	-0.023568000000	-3.373353000000
H	-0.261224000000	-1.655236000000	-2.927438000000
H	-1.907381000000	-2.924277000000	-4.282785000000
H	-0.008047000000	-0.834391000000	-0.376079000000
H	0.485842000000	2.419228000000	-1.297599000000
H	0.691190000000	-4.281787000000	1.249642000000
H	-1.593978000000	-5.092472000000	1.857957000000
H	-2.170373000000	-1.611413000000	4.291051000000
H	0.121485000000	-0.832116000000	3.721506000000
H	-3.855542000000	-4.491104000000	2.715531000000
H	-3.332451000000	-4.563356000000	4.401846000000
H	-4.060735000000	-3.081115000000	3.765076000000
H	-6.389346000000	0.632557000000	-1.335613000000
H	-7.788808000000	0.455393000000	0.698096000000
H	-7.373083000000	1.960904000000	2.626597000000
H	-5.520745000000	3.609979000000	2.527885000000
H	-4.104757000000	3.763718000000	0.488904000000
H	-4.925720000000	2.147470000000	-2.701487000000
H	-3.979787000000	3.406845000000	-1.890849000000
H	-2.363302000000	1.282532000000	1.646916000000
H	-4.557072000000	0.480776000000	2.431267000000
H	-5.408401000000	-1.738991000000	1.718100000000
H	-4.001025000000	-3.180706000000	0.256326000000
H	-1.789752000000	-2.388655000000	-0.497543000000
H	3.938970000000	0.347406000000	2.161031000000
H	5.224324000000	1.965764000000	3.513818000000
H	4.090003000000	3.950812000000	4.493288000000
H	1.643757000000	4.246914000000	4.152693000000
H	0.371438000000	2.618603000000	2.828906000000
C	2.722767000000	0.599825000000	-1.703767000000
N	3.429355000000	1.557916000000	-1.091365000000
N	4.519746000000	1.054429000000	-0.457729000000
C	4.448939000000	-0.225521000000	-0.696789000000
N	3.398241000000	-0.537200000000	-1.515580000000

C	5.135934000000	-1.450541000000	-0.203625000000
C	4.524202000000	-2.532644000000	-1.117006000000
C	3.135342000000	-2.008437000000	-1.539586000000
C	2.572220000000	-2.572563000000	-2.840151000000
C	1.559801000000	-3.694888000000	-2.561092000000
C	3.164899000000	2.965715000000	-1.099095000000
C	2.848799000000	3.587200000000	-2.303712000000
C	2.539471000000	4.942291000000	-2.293441000000
C	2.569245000000	5.654842000000	-1.096649000000
C	2.918085000000	5.018091000000	0.092037000000
C	3.222048000000	3.661213000000	0.103408000000
C	0.994290000000	-4.221700000000	-3.878034000000
C	2.159228000000	-4.819410000000	-1.715994000000
H	6.222578000000	-1.379599000000	-0.259801000000
H	4.827975000000	-1.621410000000	0.834397000000
H	4.421789000000	-3.480028000000	-0.590194000000
H	5.143185000000	-2.672126000000	-2.010163000000
H	2.432754000000	-2.147816000000	-0.711360000000
H	3.386289000000	-2.961564000000	-3.466911000000
H	2.081643000000	-1.784092000000	-3.418879000000
H	0.736284000000	-3.255646000000	-1.977321000000
H	2.848093000000	3.020485000000	-3.230272000000
H	2.283455000000	5.440597000000	-3.221780000000
H	2.323933000000	6.711565000000	-1.092911000000
H	2.942887000000	5.568119000000	1.026385000000
H	3.489803000000	3.146448000000	1.019320000000
H	0.215344000000	-4.969135000000	-3.701643000000
H	0.565504000000	-3.413044000000	-4.480986000000
H	1.784330000000	-4.695794000000	-4.471633000000
H	1.436064000000	-5.631713000000	-1.595174000000
H	2.433930000000	-4.466974000000	-0.715790000000
H	3.050578000000	-5.236139000000	-2.201555000000
H	-0.188819000000	1.876066000000	1.038303000000

TSb4''

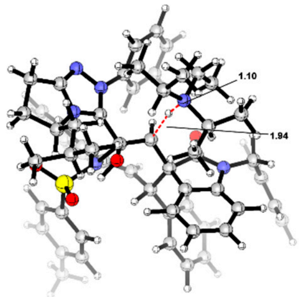

Frequency -294.4606

Zero-point correction= 1.231836 (Hartree/Particle)

Thermal correction to Energy= 1.297975

Thermal correction to Enthalpy= 1.298919

Thermal correction to Gibbs Free Energy= 1.130625

Sum of electronic and zero-point Energies= -3430.241300

Sum of electronic and thermal Energies= -3430.175161

Sum of electronic and thermal Enthalpies= -3430.174217

Sum of electronic and thermal Free Energies= -3430.342511

E(RM062X) = -3432.21300784

H	-0.536449000000	-2.793771000000	0.223748000000
C	-0.064632000000	-0.913119000000	0.260977000000
N	-0.557208000000	-3.890045000000	0.172787000000
C	-0.234768000000	-4.397871000000	1.544991000000
C	0.565440000000	-4.143259000000	-0.827124000000
C	-1.938362000000	-4.291061000000	-0.298466000000
C	1.005423000000	-3.759571000000	2.149531000000
H	-0.144774000000	-5.484916000000	1.484259000000
H	-1.089520000000	-4.161903000000	2.178767000000
H	1.167290000000	-4.197450000000	3.137462000000
H	1.905500000000	-3.935334000000	1.554280000000
H	0.884411000000	-2.681719000000	2.279090000000
C	0.119454000000	-3.794607000000	-2.240417000000

C	1.212536000000	-5.521644000000	-0.739364000000
H	1.312696000000	-3.403904000000	-0.527508000000
H	-0.390095000000	-2.826962000000	-2.269010000000
H	1.012493000000	-3.709067000000	-2.864678000000
H	-0.523784000000	-4.565477000000	-2.674536000000
H	1.659677000000	-5.719022000000	0.237071000000
H	0.530120000000	-6.337586000000	-0.981869000000
H	2.021624000000	-5.539346000000	-1.474799000000
C	-3.008250000000	-3.871551000000	0.705769000000
C	-2.075505000000	-5.776021000000	-0.617096000000
H	-2.084494000000	-3.707354000000	-1.210964000000
H	-2.818639000000	-2.874983000000	1.107582000000
H	-3.974980000000	-3.855588000000	0.195832000000
H	-3.087174000000	-4.574492000000	1.539835000000
H	-1.487181000000	-6.083783000000	-1.480607000000
H	-1.804442000000	-6.400359000000	0.239205000000
H	-3.124823000000	-5.978019000000	-0.847279000000
C	-1.259600000000	-0.300192000000	-0.220382000000
O	-1.578861000000	-0.622766000000	-1.529314000000
C	1.282227000000	-0.499636000000	-0.358902000000
C	1.735007000000	-1.059266000000	-1.690762000000
C	2.932368000000	-1.764639000000	-1.505060000000
C	3.633027000000	-2.319922000000	-2.565730000000
C	3.125016000000	-2.117734000000	-3.853287000000
C	1.262221000000	-0.845464000000	-2.974039000000
C	1.960194000000	-1.384148000000	-4.060311000000
N	3.248308000000	-1.795038000000	-0.136110000000
C	2.356973000000	-1.046841000000	0.591338000000
O	2.412733000000	-0.879005000000	1.798903000000
C	1.447736000000	1.071913000000	-0.456357000000
C	0.874011000000	1.772607000000	0.740433000000
C	-0.440394000000	2.045567000000	0.808914000000
N	-1.265060000000	1.446659000000	-0.185650000000
S	-2.170344000000	2.418065000000	-1.166824000000
O	-2.182959000000	1.724323000000	-2.481108000000
O	-3.488647000000	2.725096000000	-0.610827000000
C	-1.229267000000	3.902845000000	-1.317848000000
C	-0.101257000000	3.895762000000	-2.137300000000
C	0.707474000000	5.020643000000	-2.162985000000
C	0.396780000000	6.148386000000	-1.390143000000
C	-0.747545000000	6.125411000000	-0.589196000000
C	-1.567804000000	5.002765000000	-0.538942000000
C	1.277565000000	7.367438000000	-1.447113000000
C	4.398979000000	-2.437934000000	0.482810000000
C	5.572827000000	-1.514452000000	0.720261000000
C	6.527632000000	-1.294145000000	-0.272410000000
C	7.626339000000	-0.476675000000	-0.023367000000
C	7.780277000000	0.121807000000	1.224874000000
C	6.819419000000	-0.078597000000	2.214228000000
C	5.717038000000	-0.889599000000	1.960702000000
C	2.856446000000	1.518297000000	-0.792013000000
C	3.863329000000	1.581848000000	0.175701000000
C	5.149245000000	1.988055000000	-0.167807000000
C	5.451462000000	2.327463000000	-1.483093000000
C	4.458195000000	2.264279000000	-2.456411000000
C	3.171465000000	1.866689000000	-2.108878000000
C	-1.060074000000	2.866809000000	1.878683000000
C	-0.309848000000	3.856347000000	2.529034000000
C	-0.866886000000	4.604568000000	3.558205000000
C	-2.187984000000	4.388413000000	3.947379000000
C	-2.949291000000	3.425236000000	3.292026000000
C	-2.392053000000	2.672715000000	2.263417000000
H	3.660081000000	-2.530259000000	-4.702362000000
H	4.557812000000	-2.866719000000	-2.414960000000
H	0.349265000000	-0.277488000000	-3.130901000000
H	1.592501000000	-1.228493000000	-5.068643000000

H	0.809457000000	1.341631000000	-1.305190000000
H	0.124035000000	3.025691000000	-2.746509000000
H	1.596651000000	5.026819000000	-2.786674000000
H	-0.996989000000	6.994197000000	0.012286000000
H	-2.446624000000	4.968220000000	0.097522000000
H	2.333982000000	7.088306000000	-1.432827000000
H	1.097274000000	7.925556000000	-2.371298000000
H	1.084543000000	8.038168000000	-0.608005000000
H	6.419190000000	-1.771432000000	-1.243021000000
H	8.369276000000	-0.319140000000	-0.798433000000
H	8.646292000000	0.744274000000	1.426534000000
H	6.934125000000	0.389935000000	3.186572000000
H	4.957126000000	-1.045791000000	2.721274000000
H	4.687186000000	-3.281865000000	-0.151298000000
H	4.053296000000	-2.834184000000	1.442445000000
H	3.651027000000	1.294796000000	1.202638000000
H	5.919163000000	2.024696000000	0.594995000000
H	6.457568000000	2.636897000000	-1.747720000000
H	4.683766000000	2.523144000000	-3.486125000000
H	2.399352000000	1.806581000000	-2.872365000000
H	0.703713000000	4.059181000000	2.196413000000
H	-0.274623000000	5.370608000000	4.048022000000
H	-2.624593000000	4.977817000000	4.746791000000
H	-3.982343000000	3.258258000000	3.578880000000
H	-3.004358000000	1.950243000000	1.733820000000
C	-2.475723000000	-0.468980000000	0.681327000000
N	-2.617715000000	-0.654201000000	2.014525000000
N	-3.938488000000	-0.641282000000	2.391757000000
C	-4.582354000000	-0.436183000000	1.283610000000
N	-3.730134000000	-0.332489000000	0.224279000000
C	-6.003369000000	-0.297442000000	0.850241000000
C	-5.816683000000	0.297166000000	-0.567557000000
C	-4.449708000000	-0.218141000000	-1.075293000000
C	-4.471242000000	-1.579532000000	-1.779162000000
C	-4.493252000000	-1.463356000000	-3.306822000000
C	-1.694446000000	-1.031969000000	3.063795000000
C	-1.994940000000	-2.205055000000	3.754489000000
C	-1.167662000000	-2.610449000000	4.796171000000
C	-0.054032000000	-1.846402000000	5.139976000000
C	0.215176000000	-0.663505000000	4.458267000000
C	-0.617683000000	-0.233907000000	3.428981000000
C	-4.413540000000	-2.857230000000	-3.926368000000
C	-5.715661000000	-0.703267000000	-3.820092000000
H	-6.481544000000	-1.282068000000	0.831178000000
H	-6.577026000000	0.346330000000	1.517007000000
H	-5.765446000000	1.387234000000	-0.503866000000
H	-6.623054000000	0.015777000000	-1.245100000000
H	-3.964242000000	0.531216000000	-1.697657000000
H	-5.335146000000	-2.167468000000	-1.433192000000
H	-3.563112000000	-2.120525000000	-1.498091000000
H	-3.592543000000	-0.902944000000	-3.594782000000
H	-2.884028000000	-2.769744000000	3.493820000000
H	-1.401041000000	-3.517672000000	5.343271000000
H	0.594391000000	-2.166229000000	5.948607000000
H	1.075698000000	-0.060450000000	4.723513000000
H	-0.418001000000	0.694207000000	2.911143000000
H	-4.376311000000	-2.800298000000	-5.017118000000
H	-3.519457000000	-3.391943000000	-3.586628000000
H	-5.291415000000	-3.453301000000	-3.652626000000
H	-5.714459000000	-0.666398000000	-4.912444000000
H	-5.738698000000	0.330439000000	-3.461022000000
H	-6.641647000000	-1.199669000000	-3.505224000000
H	1.533340000000	2.074476000000	1.548619000000
H	0.040269000000	-0.851270000000	1.335762000000
H	-1.796479000000	0.188279000000	-2.052298000000

INTb8

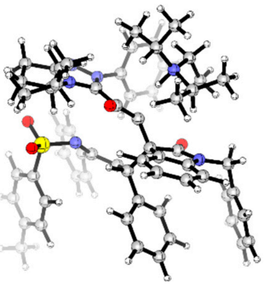

Zero-point correction= 1.218797 (Hartree/Particle)

Thermal correction to Energy= 1.286078

Thermal correction to Enthalpy= 1.287022

Thermal correction to Gibbs Free Energy= 1.115728

Sum of electronic and zero-point Energies= -3429.840554

Sum of electronic and thermal Energies= -3429.773273

Sum of electronic and thermal Enthalpies= -3429.772328

Sum of electronic and thermal Free Energies= -3429.943623

E(RM062X) = -3431.81059351

N	2.244685000000	-3.520636000000	0.887951000000
H	1.566904000000	-2.704302000000	0.746668000000
C	2.563462000000	-3.502231000000	2.352289000000
C	3.419780000000	-3.096952000000	0.009738000000
C	1.511615000000	-4.769950000000	0.433518000000
C	2.972340000000	-2.121912000000	2.845292000000
H	3.332248000000	-4.255029000000	2.540434000000
H	1.653700000000	-3.800673000000	2.875182000000
H	3.145296000000	-2.179336000000	3.922642000000
H	3.888302000000	-1.754842000000	2.374413000000
H	2.183715000000	-1.385451000000	2.670469000000
C	3.116739000000	-3.340438000000	-1.462284000000
C	4.765165000000	-3.676752000000	0.432277000000
H	3.452055000000	-2.014199000000	0.166803000000
H	2.108504000000	-3.003810000000	-1.722585000000
H	3.823282000000	-2.751383000000	-2.053085000000
H	3.240334000000	-4.393289000000	-1.734704000000
H	5.054323000000	-3.390271000000	1.445552000000
H	4.807140000000	-4.764629000000	0.351085000000
H	5.514316000000	-3.265547000000	-0.249734000000
C	0.244820000000	-4.987270000000	1.253878000000
C	2.385435000000	-6.017890000000	0.458135000000
H	1.204344000000	-4.533082000000	-0.586959000000
H	-0.291966000000	-4.052748000000	1.422896000000
H	-0.415616000000	-5.654503000000	0.694609000000
H	0.451913000000	-5.453683000000	2.221729000000
H	3.197747000000	-5.981648000000	-0.267574000000
H	2.804053000000	-6.197399000000	1.453249000000
H	1.758209000000	-6.877262000000	0.207822000000
C	0.705733000000	-0.919620000000	0.364340000000
C	-0.170629000000	-1.785314000000	-0.263117000000
O	-0.094038000000	-2.352435000000	-1.384553000000
C	1.539575000000	0.129440000000	-0.338660000000
C	2.366355000000	-0.207275000000	-1.555393000000
C	3.707034000000	0.112061000000	-1.299065000000
C	4.692763000000	-0.003544000000	-2.268718000000
C	4.297059000000	-0.428942000000	-3.541115000000
C	1.987283000000	-0.608518000000	-2.824738000000
C	2.964701000000	-0.723642000000	-3.819067000000
N	3.838708000000	0.525868000000	0.036392000000
C	2.609940000000	0.586556000000	0.657952000000
O	2.448967000000	0.911134000000	1.824101000000
C	0.567898000000	1.336140000000	-0.725134000000
C	-0.314261000000	1.814373000000	0.389514000000
C	-1.630358000000	1.487805000000	0.465469000000
N	-2.156745000000	0.512499000000	-0.370168000000
S	-3.633299000000	0.598747000000	-0.957969000000
O	-3.612016000000	0.058487000000	-2.331137000000

O	-4.666478000000	-0.024429000000	-0.094262000000
C	-4.047488000000	2.336508000000	-1.058554000000
C	-3.185482000000	3.187326000000	-1.743734000000
C	-3.431088000000	4.555388000000	-1.729138000000
C	-4.525040000000	5.080506000000	-1.035126000000
C	-5.382199000000	4.202539000000	-0.367134000000
C	-5.146290000000	2.832115000000	-0.368527000000
C	-4.754565000000	6.568564000000	-0.968515000000
C	5.068642000000	0.882549000000	0.720458000000
C	5.374568000000	2.364089000000	0.699436000000
C	6.189135000000	2.917803000000	-0.286823000000
C	6.472284000000	4.281188000000	-0.285987000000
C	5.943407000000	5.100661000000	0.707024000000
C	5.116976000000	4.555785000000	1.688689000000
C	4.832559000000	3.193595000000	1.684519000000
C	1.330983000000	2.478744000000	-1.367916000000
C	2.156281000000	3.320077000000	-0.614390000000
C	2.885486000000	4.336902000000	-1.223327000000
C	2.790121000000	4.539978000000	-2.597587000000
C	1.959168000000	3.720469000000	-3.356608000000
C	1.239709000000	2.698172000000	-2.744982000000
C	-2.472201000000	2.164136000000	1.502061000000
C	-2.367915000000	3.545350000000	1.699253000000
C	-3.165987000000	4.189422000000	2.638039000000
C	-4.077472000000	3.459573000000	3.398083000000
C	-4.185460000000	2.083388000000	3.210899000000
C	-3.395197000000	1.438203000000	2.265073000000
H	5.043928000000	-0.518933000000	-4.323607000000
H	5.728027000000	0.248646000000	-2.063486000000
H	0.949514000000	-0.864171000000	-3.016475000000
H	2.681607000000	-1.044123000000	-4.816022000000
H	-0.097056000000	0.886400000000	-1.473006000000
H	-2.319041000000	2.779008000000	-2.257393000000

H	-2.757221000000	5.228056000000	-2.253440000000
H	-6.234179000000	4.601336000000	0.178037000000
H	-5.777844000000	2.140392000000	0.179278000000
H	-4.249866000000	7.086669000000	-1.787277000000
H	-5.820112000000	6.808486000000	-1.015238000000
H	-4.367556000000	6.976020000000	-0.028102000000
H	6.612243000000	2.278792000000	-1.057715000000
H	7.111056000000	4.701042000000	-1.056397000000
H	6.169814000000	6.162242000000	0.713967000000
H	4.696015000000	5.193044000000	2.460003000000
H	4.177569000000	2.758815000000	2.435074000000
H	5.884403000000	0.311407000000	0.264966000000
H	4.951837000000	0.551782000000	1.757565000000
H	2.234989000000	3.176537000000	0.460832000000
H	3.530726000000	4.966487000000	-0.618381000000
H	3.357419000000	5.334096000000	-3.073380000000
H	1.873101000000	3.873064000000	-4.428247000000
H	0.601780000000	2.050941000000	-3.341585000000
H	-1.685462000000	4.111716000000	1.072105000000
H	-3.088431000000	5.265380000000	2.765155000000
H	-4.707179000000	3.962147000000	4.126093000000
H	-4.896129000000	1.508320000000	3.796719000000
H	-3.510612000000	0.372851000000	2.087407000000
C	-1.446721000000	-2.097054000000	0.511830000000
N	-1.827480000000	-1.989748000000	1.802820000000
N	-3.150993000000	-2.317076000000	1.983880000000
C	-3.556586000000	-2.611864000000	0.782846000000
N	-2.541471000000	-2.529903000000	-0.117652000000
C	-4.835952000000	-2.996484000000	0.121805000000
C	-4.478451000000	-2.840853000000	-1.382624000000
C	-2.928373000000	-2.844025000000	-1.505874000000
C	-2.316765000000	-4.177293000000	-1.955343000000
C	-1.958651000000	-4.162581000000	-3.442867000000

C -1.054129000000 -1.726976000000 2.987762000000

C -0.783486000000 -2.798824000000 3.835394000000

C -0.052439000000 -2.579007000000 4.999391000000

C 0.391211000000 -1.294574000000 5.309153000000

C 0.083852000000 -0.227306000000 4.469289000000

C -0.657639000000 -0.433233000000 3.309727000000

C -1.305060000000 -5.483225000000 -3.843141000000

C -3.173074000000 -3.863280000000 -4.322522000000

H -5.112493000000 -4.019571000000 0.394317000000

H -5.629271000000 -2.309827000000 0.417280000000

H -4.848214000000 -1.879498000000 -1.738905000000

H -4.909567000000 -3.642982000000 -1.984799000000

H -2.581338000000 -2.013743000000 -2.125954000000

H -3.015178000000 -5.000790000000 -1.741109000000

H -1.398372000000 -4.350945000000 -1.387906000000

H -1.224022000000 -3.356360000000 -3.566816000000

H -1.167260000000 -3.784063000000 3.589228000000

H 0.158219000000 -3.408017000000 5.667394000000

H 0.964267000000 -1.123494000000 6.214885000000

H 0.418786000000 0.775912000000 4.707432000000

H -0.901978000000 0.392974000000 2.650003000000

H -0.998218000000 -5.470319000000 -4.893185000000

H -0.417055000000 -5.685110000000 -3.234299000000

H -2.003397000000 -6.318145000000 -3.710173000000

H -2.893649000000 -3.857251000000 -5.380361000000

H -3.614402000000 -2.887958000000 -4.091908000000

H -3.946201000000 -4.630001000000 -4.186568000000

H 0.102663000000 2.488519000000 1.132068000000

H 0.400202000000 -0.537181000000 1.324009000000

TSb5

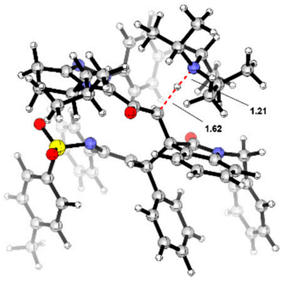

Frequency -758.9717

Zero-point correction= 1.214604 (Hartree/Particle)

Thermal correction to Energy= 1.281484

Thermal correction to Enthalpy= 1.282428

Thermal correction to Gibbs Free Energy= 1.111680

Sum of electronic and zero-point Energies= -3429.843528

Sum of electronic and thermal Energies= -3429.776648

Sum of electronic and thermal Enthalpies= -3429.775704

Sum of electronic and thermal Free Energies= -3429.946452

E(RM062X) = -3431.80656331

N 2.326733000000 -3.409473000000 0.987595000000

H 1.644419000000 -2.452350000000 0.710846000000

C 2.547689000000 -3.327332000000 2.459252000000

C 3.560538000000 -3.119505000000 0.168750000000

C 1.558799000000 -4.637593000000 0.571197000000

C 3.148175000000 -2.006634000000 2.921114000000

H 3.162691000000 -4.177491000000 2.773758000000

H 1.567617000000 -3.428934000000 2.931284000000

H 3.241749000000 -2.040644000000 4.009776000000

H 4.142020000000 -1.817595000000 2.506863000000

H 2.501738000000 -1.162746000000 2.672565000000

C 3.298431000000 -3.371168000000 -1.312544000000

C 4.839628000000 -3.805426000000 0.644816000000

H 3.692775000000 -2.039057000000 0.297775000000

H 2.322494000000 -2.985106000000 -1.617667000000

H 4.053291000000 -2.837922000000 -1.896764000000

H	3.361262000000	-4.434192000000	-1.563784000000
H	5.074685000000	-3.591485000000	1.689422000000
H	4.813662000000	-4.887977000000	0.512557000000
H	5.663253000000	-3.419148000000	0.037567000000
C	0.276331000000	-4.787401000000	1.384687000000
C	2.361754000000	-5.935892000000	0.638976000000
H	1.268220000000	-4.439114000000	-0.463762000000
H	-0.220454000000	-3.829651000000	1.530847000000
H	-0.406757000000	-5.447472000000	0.843718000000
H	0.463730000000	-5.229162000000	2.368142000000
H	3.154575000000	-5.987862000000	-0.106880000000
H	2.800641000000	-6.083895000000	1.630538000000
H	1.681120000000	-6.771452000000	0.454137000000
C	0.837622000000	-1.096243000000	0.353646000000
C	-0.232999000000	-1.720734000000	-0.332812000000
O	-0.253741000000	-2.096772000000	-1.510435000000
C	1.599271000000	0.028410000000	-0.363988000000
C	2.477003000000	-0.287306000000	-1.554867000000
C	3.798524000000	0.072824000000	-1.257818000000
C	4.823258000000	-0.035510000000	-2.186807000000
C	4.488280000000	-0.494635000000	-3.464245000000
C	2.157162000000	-0.710646000000	-2.833712000000
C	3.174126000000	-0.821287000000	-3.787766000000
N	3.869693000000	0.525686000000	0.069672000000
C	2.621349000000	0.562090000000	0.645197000000
O	2.406066000000	0.934415000000	1.789316000000
C	0.591171000000	1.187066000000	-0.776708000000
C	-0.270976000000	1.688089000000	0.344158000000
C	-1.599079000000	1.421365000000	0.421525000000
N	-2.168640000000	0.491303000000	-0.436725000000
S	-3.664187000000	0.608866000000	-0.968739000000
O	-3.697370000000	0.114511000000	-2.358353000000
O	-4.666055000000	-0.040544000000	-0.087147000000
C	-4.069536000000	2.350039000000	-0.996086000000
C	-3.230916000000	3.217000000000	-1.689469000000
C	-3.468697000000	4.584972000000	-1.623014000000
C	-4.530736000000	5.093201000000	-0.869838000000
C	-5.365636000000	4.198829000000	-0.195032000000
C	-5.137842000000	2.828367000000	-0.248073000000
C	-4.750775000000	6.579172000000	-0.749226000000
C	5.061717000000	0.950834000000	0.781767000000
C	5.320228000000	2.439967000000	0.710009000000
C	6.152763000000	2.980967000000	-0.268435000000
C	6.392943000000	4.351818000000	-0.313254000000
C	5.800821000000	5.192517000000	0.624808000000
C	4.955722000000	4.660105000000	1.597510000000
C	4.715796000000	3.290046000000	1.639808000000
C	1.315948000000	2.332246000000	-1.463429000000
C	2.126537000000	3.217176000000	-0.744963000000
C	2.829242000000	4.228787000000	-1.392580000000
C	2.715301000000	4.387636000000	-2.771193000000
C	1.892676000000	3.528883000000	-3.494940000000
C	1.203110000000	2.509616000000	-2.844726000000
C	-2.398061000000	2.101864000000	1.488645000000
C	-2.248192000000	3.475562000000	1.706735000000
C	-3.002248000000	4.125240000000	2.677880000000
C	-3.913071000000	3.407992000000	3.450344000000
C	-4.063454000000	2.038453000000	3.244621000000
C	-3.317344000000	1.388075000000	2.267022000000
H	5.266654000000	-0.582243000000	-4.215656000000
H	5.843560000000	0.244650000000	-1.946531000000
H	1.131217000000	-0.977567000000	-3.066472000000
H	2.935492000000	-1.160024000000	-4.790369000000
H	-0.078407000000	0.711037000000	-1.503625000000
H	-2.388273000000	2.821379000000	-2.250361000000
H	-2.813164000000	5.270500000000	-2.153696000000

H	-6.192476000000	4.584009000000	0.396601000000
H	-5.750742000000	2.122799000000	0.303080000000
H	-4.253229000000	7.122100000000	-1.556125000000
H	-5.815488000000	6.826084000000	-0.774691000000
H	-4.350475000000	6.951898000000	0.199951000000
H	6.624875000000	2.326407000000	-0.996506000000
H	7.047186000000	4.761173000000	-1.076264000000
H	5.992928000000	6.260439000000	0.596335000000
H	4.486134000000	5.313327000000	2.326219000000
H	4.047961000000	2.864363000000	2.384224000000
H	5.911013000000	0.389027000000	0.379091000000
H	4.916413000000	0.656678000000	1.826020000000
H	2.215991000000	3.114401000000	0.333766000000
H	3.466666000000	4.889504000000	-0.812840000000
H	3.260652000000	5.178229000000	-3.277407000000
H	1.790365000000	3.647806000000	-4.569456000000
H	0.572881000000	1.830842000000	-3.414003000000
H	-1.564614000000	4.032559000000	1.072434000000
H	-2.889696000000	5.195992000000	2.821646000000
H	-4.507574000000	3.915156000000	4.204257000000
H	-4.772120000000	1.472525000000	3.841641000000
H	-3.467953000000	0.329388000000	2.078229000000
C	-1.517849000000	-2.041560000000	0.442053000000
N	-1.898402000000	-1.959065000000	1.735294000000
N	-3.211009000000	-2.320456000000	1.908748000000
C	-3.612725000000	-2.607281000000	0.703736000000
N	-2.602486000000	-2.490543000000	-0.194794000000
C	-4.887773000000	-3.002343000000	0.042788000000
C	-4.537173000000	-2.825588000000	-1.460699000000
C	-2.987677000000	-2.787626000000	-1.588347000000
C	-2.339426000000	-4.094257000000	-2.062134000000
C	-2.020909000000	-4.064358000000	-3.558916000000
C	-1.142267000000	-1.721665000000	2.938672000000
C	-1.042570000000	-2.780548000000	3.839603000000
C	-0.324844000000	-2.605058000000	5.017839000000
C	0.281014000000	-1.378234000000	5.286739000000
C	0.141465000000	-0.319690000000	4.393372000000
C	-0.591440000000	-0.476714000000	3.219641000000
C	-1.345636000000	-5.367433000000	-3.980825000000
C	-3.263069000000	-3.791854000000	-4.407832000000
H	-5.151700000000	-4.031154000000	0.305437000000
H	-5.687332000000	-2.327991000000	0.349734000000
H	-4.930637000000	-1.870914000000	-1.810529000000
H	-4.950247000000	-3.633530000000	-2.067447000000
H	-2.664347000000	-1.937534000000	-2.193808000000
H	-3.000711000000	-4.943305000000	-1.831667000000
H	-1.401981000000	-4.243472000000	-1.517482000000
H	-1.311255000000	-3.239017000000	-3.701918000000
H	-1.536017000000	-3.720323000000	3.613366000000
H	-0.243639000000	-3.423614000000	5.725384000000
H	0.847272000000	-1.243748000000	6.202885000000
H	0.600299000000	0.641265000000	4.597941000000
H	-0.709294000000	0.344895000000	2.520665000000
H	-1.063715000000	-5.341706000000	-5.037477000000
H	-0.439889000000	-5.552794000000	-3.393830000000
H	-2.021952000000	-6.218411000000	-3.836890000000
H	-3.008251000000	-3.780469000000	-5.471693000000
H	-3.719211000000	-2.825851000000	-4.167515000000
H	-4.016049000000	-4.575067000000	-4.254515000000
H	0.179449000000	2.326720000000	1.098402000000
H	0.576406000000	-0.736177000000	1.338991000000

INTb9

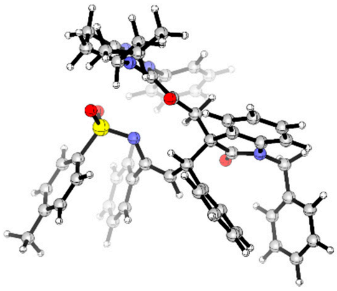

Zero-point correction=	0.950727 (Hartree/Particle)
Thermal correction to Energy=	1.005792
Thermal correction to Enthalpy=	1.006736
Thermal correction to Gibbs Free Energy=	0.858148
Sum of electronic and zero-point Energies=	-3059.244751
Sum of electronic and thermal Energies=	-3059.189686
Sum of electronic and thermal Enthalpies=	-3059.188742
Sum of electronic and thermal Free Energies=	-3059.337330

E(RM062X) = -3060.86021757

C	4.532741000000	-3.922410000000	-0.825027000000
C	4.844945000000	-2.859622000000	0.024139000000
C	3.835631000000	-1.959892000000	0.327670000000
C	2.533089000000	-2.081422000000	-0.182967000000
C	2.252124000000	-3.131767000000	-1.043730000000
C	3.254515000000	-4.056991000000	-1.353816000000
N	3.920295000000	-0.833716000000	1.153040000000
C	2.726154000000	-0.157121000000	1.197944000000
C	1.716236000000	-0.883854000000	0.279916000000
O	2.519336000000	0.825484000000	1.883780000000
C	1.295814000000	0.060971000000	-0.916075000000
C	0.727171000000	1.369125000000	-0.442214000000
C	-0.646960000000	-1.857959000000	0.498005000000
C	0.506418000000	-1.181197000000	1.183087000000
C	-0.576185000000	1.584373000000	-0.156883000000
N	-1.495993000000	0.537038000000	-0.231649000000
O	-0.582212000000	-2.462250000000	-0.548701000000
S	-3.071032000000	0.765955000000	-0.314537000000
O	-3.642471000000	-0.355841000000	-1.088109000000
O	-3.715472000000	0.963410000000	1.004333000000
C	-3.383188000000	2.241694000000	-1.270818000000
C	-2.867954000000	2.322582000000	-2.560152000000
C	-3.042792000000	3.496349000000	-3.283801000000
C	-3.724399000000	4.585058000000	-2.732740000000
C	-4.251519000000	4.466379000000	-1.443826000000
C	-4.084375000000	3.299905000000	-0.707537000000
C	-3.857462000000	5.879460000000	-3.492260000000
C	5.931389000000	0.610256000000	1.166489000000
C	7.062528000000	0.216629000000	0.453417000000
C	7.832598000000	1.157353000000	-0.226693000000
C	7.473512000000	2.501844000000	-0.198244000000
C	6.337761000000	2.901546000000	0.505297000000
C	5.571912000000	1.961120000000	1.187081000000
C	5.080538000000	-0.397012000000	1.909169000000
C	2.468699000000	0.254551000000	-1.858844000000
C	3.550276000000	1.067669000000	-1.509789000000
C	4.662682000000	1.172138000000	-2.339460000000
C	4.698808000000	0.480249000000	-3.547227000000
C	3.617986000000	-0.316100000000	-3.916375000000
C	2.515957000000	-0.429582000000	-3.074401000000
C	-0.949922000000	2.971185000000	0.271968000000
C	-1.584091000000	3.206929000000	1.493960000000
C	-1.917764000000	4.500233000000	1.881640000000
C	-1.629951000000	5.578082000000	1.047312000000
C	-0.994363000000	5.353126000000	-0.171335000000
C	-0.652588000000	4.059618000000	-0.553551000000
H	5.303882000000	-4.642542000000	-1.079612000000
H	5.846223000000	-2.740469000000	0.423009000000
H	1.265272000000	-3.231300000000	-1.476179000000
H	3.031248000000	-4.881173000000	-2.023029000000

H	0.506443000000	-0.485086000000	-1.446087000000
H	0.821193000000	-1.838240000000	2.005781000000
H	0.172108000000	-0.239994000000	1.633345000000
H	-2.321399000000	1.481204000000	-2.976277000000
H	-2.635501000000	3.575875000000	-4.288202000000
H	-4.782656000000	5.307171000000	-1.004916000000
H	-4.458844000000	3.203246000000	0.305917000000
H	-3.680731000000	5.735566000000	-4.560531000000
H	-4.852000000000	6.314855000000	-3.363974000000
H	-3.129560000000	6.612977000000	-3.128995000000
H	7.351061000000	-0.831322000000	0.435634000000
H	8.713767000000	0.840457000000	-0.775459000000
H	8.074696000000	3.237151000000	-0.723747000000
H	6.051834000000	3.948443000000	0.527685000000
H	4.681395000000	2.259113000000	1.735165000000
H	5.665845000000	-1.282531000000	2.174425000000
H	4.695949000000	0.051157000000	2.829855000000
H	3.531677000000	1.617964000000	-0.572843000000
H	5.500368000000	1.791470000000	-2.032004000000
H	5.564851000000	0.559840000000	-4.196984000000
H	3.635659000000	-0.857705000000	-4.857214000000
H	1.680751000000	-1.066526000000	-3.354503000000
H	-1.818987000000	2.361315000000	2.127508000000
H	-2.406366000000	4.667609000000	2.837353000000
H	-1.900221000000	6.586703000000	1.344655000000
H	-0.770158000000	6.186600000000	-0.830692000000
H	-0.177485000000	3.873944000000	-1.512224000000
C	-1.997296000000	-1.944907000000	1.180971000000
N	-2.465840000000	-1.552162000000	2.374850000000
N	-3.797956000000	-1.788456000000	2.509070000000
C	-4.123957000000	-2.351715000000	1.378535000000
N	-3.048000000000	-2.496031000000	0.567345000000
C	-5.359541000000	-2.874879000000	0.729922000000

C	-4.894718000000	-3.028445000000	-0.744603000000
C	-3.344289000000	-3.128287000000	-0.733642000000
C	-2.766754000000	-4.548142000000	-0.783424000000
C	-2.374927000000	-4.970044000000	-2.203040000000
C	-1.779299000000	-0.870876000000	3.431968000000
C	-0.779671000000	-1.529120000000	4.137605000000
C	-0.115963000000	-0.840770000000	5.147121000000
C	-0.475733000000	0.471594000000	5.445726000000
C	-1.506763000000	1.097603000000	4.748720000000
C	-2.177365000000	0.425528000000	3.730791000000
C	-1.734765000000	-6.356695000000	-2.180285000000
C	-3.559216000000	-4.936759000000	-3.169721000000
H	-5.650331000000	-3.826600000000	1.184884000000
H	-6.186760000000	-2.172932000000	0.837415000000
H	-5.169738000000	-2.130674000000	-1.298044000000
H	-5.340103000000	-3.904724000000	-1.217866000000
H	-2.903787000000	-2.489589000000	-1.499162000000
H	-3.491309000000	-5.261416000000	-0.363712000000
H	-1.870178000000	-4.589279000000	-0.157287000000
H	-1.624630000000	-4.245252000000	-2.548027000000
H	-0.533152000000	-2.559393000000	3.899799000000
H	0.676835000000	-1.330895000000	5.701292000000
H	0.048998000000	1.007558000000	6.229318000000
H	-1.782784000000	2.119538000000	4.986435000000
H	-2.974152000000	0.872793000000	3.142069000000
H	-1.410599000000	-6.655405000000	-3.181164000000
H	-0.862469000000	-6.380870000000	-1.520124000000
H	-2.450782000000	-7.106033000000	-1.823582000000
H	-3.250333000000	-5.258688000000	-4.168247000000
H	-3.987175000000	-3.933886000000	-3.265598000000
H	-4.351956000000	-5.615583000000	-2.831673000000
H	1.415329000000	2.191644000000	-0.285831000000---------

TSb6

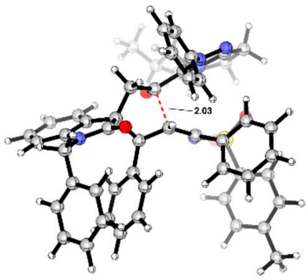

Frequency -208.8011

Zero-point correction= 0.950261 (Hartree/Particle)

Thermal correction to Energy= 1.004532

Thermal correction to Enthalpy= 1.005476

Thermal correction to Gibbs Free Energy= 0.858884

Sum of electronic and zero-point Energies= -3059.233716

Sum of electronic and thermal Energies= -3059.179445

Sum of electronic and thermal Enthalpies= -3059.178501

Sum of electronic and thermal Free Energies= -3059.325093

E(RM062X) = -3060.84609796

C	0.029315000000	-0.100770000000	0.340484000000
C	-0.575658000000	-2.029353000000	0.204805000000
C	1.071631000000	-0.424959000000	-0.698477000000
C	1.823216000000	-1.690815000000	-0.124863000000
C	2.539489000000	-2.413567000000	-1.230337000000
C	3.919955000000	-2.246928000000	-1.064362000000
C	4.827785000000	-2.741149000000	-1.988378000000
C	4.312519000000	-3.423807000000	-3.095300000000
C	2.038659000000	-3.082798000000	-2.330864000000
C	2.942299000000	-3.598374000000	-3.268114000000
N	4.160234000000	-1.553549000000	0.132659000000
C	2.984544000000	-1.240781000000	0.769631000000
O	2.907840000000	-0.704574000000	1.861795000000
C	0.790354000000	-2.474136000000	0.719392000000
C	-1.119226000000	0.710031000000	0.068494000000
N	-1.757959000000	0.555830000000	-1.068230000000
O	-0.867860000000	-2.323390000000	-0.982108000000
S	-3.246944000000	1.247412000000	-1.298873000000
O	-3.677266000000	0.817028000000	-2.623902000000
O	-4.147553000000	0.959187000000	-0.169323000000
C	-2.958769000000	3.002082000000	-1.325718000000
C	-2.108883000000	3.521097000000	-2.300596000000
C	-1.822754000000	4.878427000000	-2.282993000000
C	-2.378777000000	5.719846000000	-1.311104000000
C	-3.232184000000	5.170524000000	-0.353429000000
C	-3.526974000000	3.810104000000	-0.351262000000
C	-2.064367000000	7.193249000000	-1.316298000000
C	5.447959000000	-1.250558000000	0.727689000000
C	5.894298000000	0.177350000000	0.504982000000
C	6.504545000000	0.557090000000	-0.690805000000
C	6.943712000000	1.864631000000	-0.872614000000
C	6.777399000000	2.803369000000	0.143016000000
C	6.151791000000	2.434862000000	1.331014000000
C	5.704486000000	1.128470000000	1.507857000000
C	2.014492000000	0.687068000000	-1.105230000000
C	2.461345000000	1.663687000000	-0.209582000000
C	3.343150000000	2.656649000000	-0.626661000000
C	3.807692000000	2.677101000000	-1.938904000000
C	3.384561000000	1.698217000000	-2.833579000000
C	2.493622000000	0.713520000000	-2.418203000000
C	-1.465665000000	1.711508000000	1.141581000000
C	-2.498294000000	1.576823000000	2.071195000000
C	-2.702621000000	2.563349000000	3.034757000000
C	-1.897876000000	3.698822000000	3.062816000000
C	-0.871378000000	3.841145000000	2.131787000000
C	-0.647021000000	2.846560000000	1.186798000000
H	5.001685000000	-3.822149000000	-3.833201000000
H	5.897830000000	-2.607395000000	-1.867179000000

H	0.961827000000	-3.188932000000	-2.439319000000
H	2.573655000000	-4.132096000000	-4.137602000000
H	0.541565000000	-0.764693000000	-1.593794000000
H	0.891354000000	-3.553329000000	0.565913000000
H	0.946085000000	-2.238979000000	1.775423000000
H	-1.675179000000	2.863170000000	-3.047527000000
H	-1.153482000000	5.295747000000	-3.030403000000
H	-3.661640000000	5.812338000000	0.410859000000
H	-4.164830000000	3.365284000000	0.405655000000
H	-2.573812000000	7.695954000000	-2.144562000000
H	-2.384467000000	7.670339000000	-0.387538000000
H	-0.991725000000	7.365935000000	-1.439432000000
H	6.637348000000	-0.176550000000	-1.482335000000
H	7.421259000000	2.149867000000	-1.804950000000
H	7.131140000000	3.820674000000	0.006065000000
H	6.009918000000	3.166560000000	2.120609000000
H	5.193836000000	0.834690000000	2.420670000000
H	6.178779000000	-1.954857000000	0.319682000000
H	5.352951000000	-1.440152000000	1.801042000000
H	2.127833000000	1.633449000000	0.824006000000
H	3.685609000000	3.404661000000	0.081273000000
H	4.503718000000	3.447154000000	-2.257490000000
H	3.744011000000	1.702107000000	-3.858038000000
H	2.158911000000	-0.050075000000	-3.116092000000
H	-3.150096000000	0.713748000000	2.036690000000
H	-3.500319000000	2.441431000000	3.760510000000
H	-2.068311000000	4.468641000000	3.808841000000
H	-0.242542000000	4.725645000000	2.141087000000
H	0.150491000000	2.952361000000	0.456153000000
C	-1.802256000000	-1.967941000000	1.081068000000
N	-1.999611000000	-1.818655000000	2.400510000000
N	-3.326495000000	-1.680362000000	2.722309000000
C	-3.922066000000	-1.752165000000	1.563003000000
N	-3.034357000000	-1.949652000000	0.551598000000
C	-5.319331000000	-1.643266000000	1.052130000000
C	-5.142478000000	-1.811797000000	-0.485976000000
C	-3.655552000000	-2.176368000000	-0.775121000000
C	-3.441484000000	-3.619413000000	-1.250227000000
C	-3.331150000000	-3.689477000000	-2.776461000000
C	-0.990593000000	-1.847838000000	3.421052000000
C	-0.617366000000	-3.082046000000	3.941933000000
C	0.409873000000	-3.133253000000	4.877240000000
C	1.048198000000	-1.959954000000	5.273728000000
C	0.651279000000	-0.731574000000	4.753034000000
C	-0.382636000000	-0.665185000000	3.824021000000
C	-3.119454000000	-5.134457000000	-3.222280000000
C	-4.543962000000	-3.073364000000	-3.476496000000
H	-5.958737000000	-2.407191000000	1.500069000000
H	-5.724196000000	-0.658899000000	1.297189000000
H	-5.391519000000	-0.877387000000	-0.986963000000
H	-5.793661000000	-2.599643000000	-0.868315000000
H	-3.183016000000	-1.487848000000	-1.476420000000
H	-4.267615000000	-4.254853000000	-0.898213000000
H	-2.510315000000	-4.000868000000	-0.826145000000
H	-2.441065000000	-3.103809000000	-3.041055000000
H	-1.119616000000	-3.981549000000	3.600863000000
H	0.719166000000	-4.088939000000	5.286318000000
H	1.861930000000	-2.004856000000	5.989811000000
H	1.153733000000	0.179381000000	5.057988000000
H	-0.706509000000	0.281515000000	3.403604000000
H	-2.980189000000	-5.194726000000	-4.305654000000
H	-2.239129000000	-5.572646000000	-2.742045000000
H	-3.988711000000	-5.750582000000	-2.963348000000
H	-4.442380000000	-3.155233000000	-4.562697000000
H	-4.659257000000	-2.010222000000	-3.239765000000
H	-5.465141000000	-3.596305000000	-3.189192000000

H 0.453622000000 0.067306000000 1.331652000000

INTb10

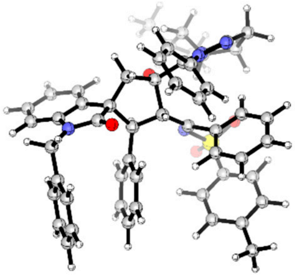

Zero-point correction= 0.950939 (Hartree/Particle)

Thermal correction to Energy= 1.005281

Thermal correction to Enthalpy= 1.006225

Thermal correction to Gibbs Free Energy= 0.859962

Sum of electronic and zero-point Energies= -3059.245481

Sum of electronic and thermal Energies= -3059.191139

Sum of electronic and thermal Enthalpies= -3059.190195

Sum of electronic and thermal Free Energies= -3059.336458

E(RM062X) = -3060.85919190

C -0.160328000000 -0.372155000000 0.334841000000

C -0.787017000000 -1.897809000000 0.458815000000

C 1.035950000000 -0.752667000000 -0.524096000000

C 1.639131000000 -1.987894000000 0.247813000000

C 2.323646000000 -2.907373000000 -0.721306000000

C 3.706063000000 -2.837605000000 -0.517489000000

C 4.594811000000 -3.528923000000 -1.326649000000

C 4.052687000000 -4.311206000000 -2.352809000000

C 1.795097000000 -3.675774000000 -1.740611000000

C 2.677972000000 -4.390578000000 -2.558946000000

N 3.965966000000 -2.007865000000 0.587337000000

C 2.796394000000 -1.536883000000 1.138173000000

O 2.727467000000 -0.868243000000 2.154459000000

C 0.468554000000 -2.551764000000 1.094503000000

C -1.108383000000 0.581103000000 -0.278974000000

N -1.331412000000 0.446219000000 -1.542564000000

O -1.129400000000 -2.429284000000 -0.677891000000

S -2.364408000000 1.481988000000 -2.380386000000

O -2.055674000000 1.280627000000 -3.783490000000

O -3.742998000000 1.293948000000 -1.914958000000

C -1.797992000000 3.101526000000 -1.902275000000

C -0.441218000000 3.389293000000 -2.039538000000

C 0.028880000000 4.614101000000 -1.586403000000

C -0.838348000000 5.551659000000 -1.012816000000

C -2.194230000000 5.235917000000 -0.900618000000

C -2.683370000000 4.008500000000 -1.338295000000

C -0.314754000000 6.881166000000 -0.535507000000

C 5.258743000000 -1.609355000000 1.103012000000

C 5.723438000000 -0.280010000000 0.545569000000

C 5.774923000000 -0.070821000000 -0.835569000000

C 6.232770000000 1.138567000000 -1.343676000000

C 6.637239000000 2.154922000000 -0.479365000000

C 6.566950000000 1.960220000000 0.895574000000

C 6.106987000000 0.746864000000 1.404631000000

C 1.930050000000 0.399559000000 -0.915108000000

C 2.416395000000 1.335446000000 0.009401000000

C 3.119618000000 2.456327000000 -0.421201000000

C 3.350471000000 2.663795000000 -1.780325000000

C 2.893453000000 1.730088000000 -2.706032000000

C 2.189146000000 0.607743000000 -2.272859000000

C -1.710129000000 1.581097000000 0.662931000000

C -3.071674000000 1.594218000000 0.971661000000

C -3.568110000000 2.515537000000 1.892593000000

C -2.717509000000 3.449341000000 2.477713000000

C -1.359435000000 3.448270000000 2.158366000000

C -0.852409000000 2.502879000000 1.274604000000

H	4.724574000000	-4.865689000000	-3.000390000000
H	5.668680000000	-3.467580000000	-1.180040000000
H	0.714844000000	-3.696614000000	-1.872621000000
H	2.290711000000	-5.006135000000	-3.363841000000
H	0.602398000000	-1.168380000000	-1.436864000000
H	0.386781000000	-3.637237000000	1.004131000000
H	0.632498000000	-2.266901000000	2.137078000000
H	0.242717000000	2.654874000000	-2.458847000000
H	1.090040000000	4.835429000000	-1.666070000000
H	-2.873819000000	5.951685000000	-0.446247000000
H	-3.726469000000	3.733793000000	-1.218455000000
H	0.681324000000	6.777630000000	-0.097724000000
H	-0.236061000000	7.588308000000	-1.367671000000
H	-0.976754000000	7.322065000000	0.213295000000
H	5.440555000000	-0.852753000000	-1.513063000000
H	6.261185000000	1.293971000000	-2.417605000000
H	6.995277000000	3.098870000000	-0.878886000000
H	6.866659000000	2.752249000000	1.575006000000
H	6.040440000000	0.595855000000	2.478777000000
H	5.976210000000	-2.403985000000	0.872311000000
H	5.164895000000	-1.543860000000	2.190441000000
H	2.250370000000	1.177060000000	1.071409000000
H	3.501553000000	3.163963000000	0.308638000000
H	3.903215000000	3.538556000000	-2.111239000000
H	3.071772000000	1.878324000000	-3.766792000000
H	1.802523000000	-0.106095000000	-2.995094000000
H	-3.739669000000	0.906073000000	0.467044000000
H	-4.624722000000	2.509324000000	2.140878000000
H	-3.110844000000	4.176077000000	3.181353000000
H	-0.692637000000	4.180557000000	2.602724000000
H	0.205366000000	2.492083000000	1.024305000000
C	-2.052083000000	-1.740466000000	1.337200000000
N	-2.301392000000	-1.407067000000	2.616076000000

N	-3.637887000000	-1.214766000000	2.870025000000
C	-4.191019000000	-1.450203000000	1.716398000000
N	-3.266903000000	-1.772437000000	0.769509000000
C	-5.563416000000	-1.412414000000	1.128836000000
C	-5.241814000000	-1.318758000000	-0.387986000000
C	-3.828751000000	-1.931000000000	-0.595181000000
C	-3.800044000000	-3.386712000000	-1.067737000000
C	-3.537935000000	-3.462475000000	-2.575086000000
C	-1.349603000000	-1.073343000000	3.635812000000
C	-0.618806000000	-2.072359000000	4.265728000000
C	0.322303000000	-1.707960000000	5.223193000000
C	0.504586000000	-0.366225000000	5.547804000000
C	-0.259743000000	0.618801000000	4.926699000000
C	-1.199134000000	0.269191000000	3.963641000000
C	-3.455233000000	-4.919507000000	-3.022954000000
C	-4.586247000000	-2.701303000000	-3.388640000000
H	-6.099884000000	-2.332742000000	1.379760000000
H	-6.144841000000	-0.568020000000	1.501921000000
H	-5.226362000000	-0.274148000000	-0.712464000000
H	-5.982623000000	-1.844441000000	-0.991997000000
H	-3.214993000000	-1.321347000000	-1.258002000000
H	-4.747308000000	-3.888035000000	-0.815015000000
H	-2.979169000000	-3.893784000000	-0.558069000000
H	-2.559120000000	-2.988786000000	-2.724763000000
H	-0.779036000000	-3.110697000000	3.994059000000
H	0.913769000000	-2.473586000000	5.712797000000
H	1.246331000000	-0.087636000000	6.288871000000
H	-0.120308000000	1.663924000000	5.181823000000
H	-1.809130000000	1.014080000000	3.461887000000
H	-3.214354000000	-4.989070000000	-4.087877000000
H	-2.684628000000	-5.459341000000	-2.464575000000
H	-4.412378000000	-5.430897000000	-2.863780000000
H	-4.373143000000	-2.777337000000	-4.458759000000

H -4.609638000000 -1.634313000000 -3.140735000000

H -5.587937000000 -3.117770000000 -3.218916000000

H 0.142767000000 -0.029868000000 1.331682000000

TSb7

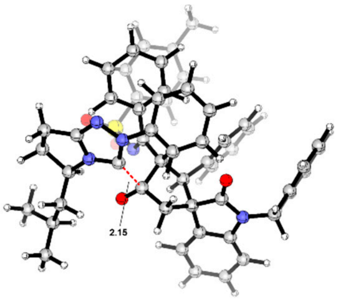

Frequency -86.4326

Zero-point correction= 0.949065 (Hartree/Particle)

Thermal correction to Energy= 1.003523

Thermal correction to Enthalpy= 1.004468

Thermal correction to Gibbs Free Energy= 0.857257

Sum of electronic and zero-point Energies= -3059.231946

Sum of electronic and thermal Energies= -3059.177487

Sum of electronic and thermal Enthalpies= -3059.176543

Sum of electronic and thermal Free Energies= -3059.323754

E(RM062X) = -3060.84438091

C -0.842487000000 -1.922477000000 0.188337000000

C -2.280204000000 -1.436642000000 1.706629000000

C -0.216485000000 -0.487438000000 0.123807000000

C 1.044179000000 -0.880526000000 -0.659535000000

C 1.537555000000 -2.191468000000 0.077474000000

C 2.254134000000 -3.076481000000 -0.902234000000

C 3.619783000000 -3.082809000000 -0.594941000000

C 4.537573000000 -3.764133000000 -1.379714000000

C 4.045022000000 -4.456645000000 -2.491285000000

C 1.778207000000 -3.756668000000 -2.006435000000

C 2.688500000000 -4.460758000000 -2.803059000000

N 3.828609000000 -2.343415000000 0.580783000000

C 2.643376000000 -1.862077000000 1.081467000000

O 2.521797000000 -1.282654000000 2.145352000000

C 0.284237000000 -2.754202000000 0.801536000000

C -1.099626000000 0.510200000000 -0.555838000000

N -1.301596000000 0.312769000000 -1.808376000000

O -1.529780000000 -2.374374000000 -0.721918000000

S -2.208883000000 1.377674000000 -2.766157000000

O -1.847724000000 1.043278000000 -4.130035000000

O -3.611813000000 1.348176000000 -2.355048000000

C -1.510627000000 2.969250000000 -2.374446000000

C -0.132830000000 3.130675000000 -2.491782000000

C 0.436263000000 4.333403000000 -2.092534000000

C -0.356362000000 5.373483000000 -1.597756000000

C -1.739084000000 5.186545000000 -1.511849000000

C -2.325514000000 3.985127000000 -1.893013000000

C 0.262012000000 6.678274000000 -1.168601000000

C 5.089044000000 -2.088902000000 1.248344000000

C 5.684887000000 -0.748750000000 0.873901000000

C 5.979473000000 -0.454518000000 -0.460142000000

C 6.556170000000 0.763477000000 -0.797957000000

C 6.837913000000 1.703968000000 0.191969000000

C 6.525034000000 1.425850000000 1.518204000000

C 5.945511000000 0.204134000000 1.855848000000

C 2.046157000000 0.225001000000 -0.887732000000

C 2.489015000000 1.065264000000 0.143060000000

C 3.331455000000 2.137498000000 -0.134038000000

C 3.748747000000 2.386070000000 -1.440394000000

C 3.334356000000 1.544368000000 -2.467992000000

C 2.490026000000 0.470649000000 -2.189733000000

C -1.643072000000 1.616790000000 0.293949000000

C -3.013117000000 1.772280000000 0.513202000000

C	-3.477041000000	2.830850000000	1.290140000000
C	-2.582553000000	3.759444000000	1.815897000000
C	-1.214287000000	3.607308000000	1.594391000000
C	-0.742155000000	2.524884000000	0.861872000000
H	4.740966000000	-5.000816000000	-3.121583000000
H	5.598097000000	-3.763938000000	-1.148656000000
H	0.715136000000	-3.737586000000	-2.236474000000
H	2.337164000000	-5.007502000000	-3.671251000000
H	0.686580000000	-1.208925000000	-1.640637000000
H	0.122253000000	-3.815166000000	0.607506000000
H	0.386551000000	-2.565457000000	1.870183000000
H	0.490812000000	2.317923000000	-2.854152000000
H	1.514168000000	4.456422000000	-2.154128000000
H	-2.362509000000	5.985503000000	-1.119073000000
H	-3.391970000000	3.812158000000	-1.791159000000
H	1.336833000000	6.572294000000	-1.006186000000
H	0.113683000000	7.448762000000	-1.932005000000
H	-0.191424000000	7.042638000000	-0.242685000000
H	5.748013000000	-1.180359000000	-1.236230000000
H	6.778335000000	0.984015000000	-1.837472000000
H	7.291295000000	2.654060000000	-0.073773000000
H	6.727535000000	2.159623000000	2.292257000000
H	5.689362000000	-0.012463000000	2.889476000000
H	5.773496000000	-2.906934000000	1.001321000000
H	4.895091000000	-2.122503000000	2.323890000000
H	2.176522000000	0.876596000000	1.166864000000
H	3.672631000000	2.774372000000	0.676179000000
H	4.409397000000	3.222104000000	-1.650991000000
H	3.659700000000	1.723756000000	-3.488284000000
H	2.146358000000	-0.174701000000	-2.994308000000
H	-3.709290000000	1.081299000000	0.051472000000
H	-4.542285000000	2.936822000000	1.470088000000
H	-2.949348000000	4.596965000000	2.401272000000
H	-0.510733000000	4.327391000000	2.000660000000
H	0.323253000000	2.403037000000	0.685557000000
N	-2.432369000000	-0.699466000000	2.831184000000
N	-3.709466000000	-0.235845000000	3.064949000000
C	-4.361501000000	-0.712040000000	2.049483000000
N	-3.542581000000	-1.420312000000	1.217662000000
C	-5.758653000000	-0.671426000000	1.530011000000
C	-5.558449000000	-1.120638000000	0.056465000000
C	-4.198877000000	-1.875926000000	-0.023677000000
C	-4.279353000000	-3.402136000000	-0.075035000000
C	-4.316656000000	-3.951766000000	-1.504405000000
C	-1.364379000000	-0.210376000000	3.637303000000
C	-0.322131000000	-1.055763000000	3.998026000000
C	0.766126000000	-0.532346000000	4.688799000000
C	0.788733000000	0.813883000000	5.040754000000
C	-0.279307000000	1.642085000000	4.699113000000
C	-1.360545000000	1.136601000000	3.987760000000
C	-4.365766000000	-5.478320000000	-1.471295000000
C	-5.478903000000	-3.393873000000	-2.326550000000
H	-6.386969000000	-1.370248000000	2.090688000000
H	-6.202378000000	0.321780000000	1.617706000000
H	-5.521163000000	-0.246483000000	-0.600263000000
H	-6.377990000000	-1.754043000000	-0.285526000000
H	-3.597158000000	-1.519256000000	-0.863063000000
H	-5.156233000000	-3.748390000000	0.493835000000
H	-3.385197000000	-3.798170000000	0.417168000000
H	-3.375162000000	-3.639973000000	-1.974126000000
H	-0.373477000000	-2.106781000000	3.738228000000
H	1.599821000000	-1.178759000000	4.937715000000
H	1.639748000000	1.218908000000	5.578295000000
H	-0.263599000000	2.693395000000	4.968907000000
H	-2.187861000000	1.764933000000	3.676437000000
H	-4.327554000000	-5.895539000000	-2.481805000000

H -3.525770000000 -5.888910000000 -0.902895000000

H -5.293733000000 -5.825190000000 -1.001261000000

H -5.486958000000 -3.834631000000 -3.327789000000

H -5.413079000000 -2.308114000000 -2.450277000000

H -6.440592000000 -3.629372000000 -1.852783000000

H 0.042587000000 -0.158384000000 1.136815000000

INTb11

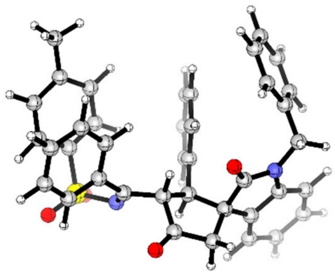

Zero-point correction= 0.626541 (Hartree/Particle)

Thermal correction to Energy= 0.664724

Thermal correction to Enthalpy= 0.665668

Thermal correction to Gibbs Free Energy= 0.554306

Sum of electronic and zero-point Energies= -2312.589927

Sum of electronic and thermal Energies= -2312.551744

Sum of electronic and thermal Enthalpies= -2312.550800

Sum of electronic and thermal Free Energies= -2312.662162

E(RM062X) = -2313.71001422

C 0.018637000000 -2.713533000000 1.797225000000

C -0.318553000000 -1.454280000000 0.987907000000

C 0.777317000000 -1.417083000000 -0.075243000000

C 2.046044000000 -1.899777000000 0.695063000000

C 3.191396000000 -2.176840000000 -0.238123000000

C 4.198254000000 -1.229814000000 -0.010470000000

C 5.354878000000 -1.198464000000 -0.773221000000

C 5.486328000000 -2.152876000000 -1.787024000000

C 3.329316000000 -3.108009000000 -1.249553000000

C 4.493583000000 -3.097431000000 -2.026107000000

N 3.825915000000 -0.409444000000 1.066343000000

C 2.598904000000 -0.765156000000 1.561909000000

O 2.057325000000 -0.248665000000 2.524610000000

C 1.495154000000 -3.045068000000 1.560714000000

C -1.717734000000 -1.407120000000 0.428029000000

N -1.842801000000 -1.820279000000 -0.778134000000

O -0.744275000000 -3.346381000000 2.475165000000

S -3.251632000000 -1.563029000000 -1.704080000000

O -2.790265000000 -1.701319000000 -3.072460000000

O -4.353059000000 -2.379670000000 -1.210218000000

C -3.614401000000 0.151685000000 -1.396049000000

C -2.636185000000 1.103794000000 -1.669177000000

C -2.887959000000 2.429729000000 -1.335746000000

C -4.099661000000 2.807752000000 -0.749652000000

C -5.067498000000 1.828061000000 -0.507007000000

C -4.832736000000 0.495829000000 -0.823833000000

C -4.370161000000 4.244930000000 -0.388315000000

C 4.581273000000 0.693358000000 1.636244000000

C 4.111162000000 2.041510000000 1.136844000000

C 4.497997000000 2.506282000000 -0.120848000000

C 4.074795000000 3.751875000000 -0.572468000000

C 3.262728000000 4.545934000000 0.234865000000

C 2.855397000000 4.078714000000 1.481902000000

C 3.272958000000 2.827131000000 1.928843000000

C 0.869845000000 -0.119620000000 -0.840020000000

C 0.841905000000 1.123469000000 -0.198243000000

C 0.860402000000 2.301210000000 -0.940524000000

C 0.915392000000 2.254405000000 -2.331860000000

C 0.951755000000 1.021707000000 -2.978734000000

C 0.925465000000 -0.155617000000 -2.235462000000

C -2.765285000000 -0.835226000000 1.315718000000

C	-3.888874000000	-1.586400000000	1.667019000000
C	-4.858834000000	-1.021794000000	2.489733000000
C	-4.720811000000	0.287997000000	2.941578000000
C	-3.599314000000	1.036628000000	2.587882000000
C	-2.610973000000	0.470262000000	1.793021000000
H	6.382688000000	-2.150652000000	-2.398404000000
H	6.135063000000	-0.465142000000	-0.598636000000
H	2.546644000000	-3.838421000000	-1.439316000000
H	4.619966000000	-3.825784000000	-2.819198000000
H	0.550503000000	-2.208984000000	-0.799618000000
H	1.541934000000	-3.995341000000	1.016553000000
H	2.012680000000	-3.176181000000	2.513552000000
H	-1.685862000000	0.813536000000	-2.112089000000
H	-2.123296000000	3.178025000000	-1.526062000000
H	-6.009093000000	2.110667000000	-0.043556000000
H	-5.559237000000	-0.280542000000	-0.606877000000
H	-3.453087000000	4.837509000000	-0.401272000000
H	-5.072236000000	4.697511000000	-1.095748000000
H	-4.815092000000	4.321307000000	0.607768000000
H	5.134782000000	1.888962000000	-0.749807000000
H	4.385152000000	4.106263000000	-1.550109000000
H	2.942462000000	5.523833000000	-0.111121000000
H	2.215150000000	4.690259000000	2.110133000000
H	2.946899000000	2.447467000000	2.893276000000
H	5.637265000000	0.533516000000	1.401517000000
H	4.460422000000	0.634893000000	2.721437000000
H	0.818632000000	1.179454000000	0.887654000000
H	0.841839000000	3.255713000000	-0.425048000000
H	0.929007000000	3.174273000000	-2.908406000000
H	0.985922000000	0.972931000000	-4.062258000000
H	0.921067000000	-1.117269000000	-2.741209000000
H	-3.997452000000	-2.596326000000	1.289067000000
H	-5.727264000000	-1.607155000000	2.772942000000
H	-5.486242000000	0.726037000000	3.574439000000
H	-3.493174000000	2.059631000000	2.934322000000
H	-1.742499000000	1.053992000000	1.496343000000
H	-0.188451000000	-0.628111000000	1.699864000000

INTb12'''

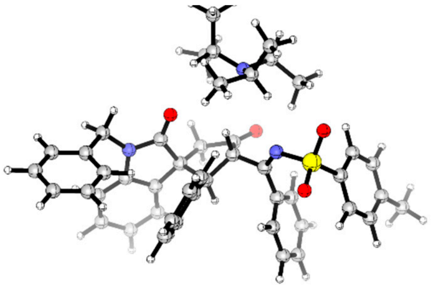

Zero-point correction=	0.893087 (Hartree/Particle)
Thermal correction to Energy=	0.944536
Thermal correction to Enthalpy=	0.945481
Thermal correction to Gibbs Free Energy=	0.805530
Sum of electronic and zero-point Energies=	-2683.209234
Sum of electronic and thermal Energies=	-2683.157785
Sum of electronic and thermal Enthalpies=	-2683.156840
Sum of electronic and thermal Free Energies=	-2683.296791

E(RM062X) = -2684.67870745

C	0.418806000000	0.083796000000	-0.647165000000
C	0.581107000000	0.271152000000	-2.150487000000
C	1.665941000000	-0.278475000000	0.117713000000
C	-0.692109000000	-0.976588000000	-0.559783000000
N	2.036220000000	0.570019000000	0.997090000000
C	2.249479000000	-1.629788000000	-0.137902000000
C	2.982636000000	-1.899626000000	-1.296291000000
C	3.493203000000	-3.178199000000	-1.496630000000
C	3.241124000000	-4.189962000000	-0.571425000000
C	2.487095000000	-3.922473000000	0.568146000000

C	1.995347000000	-2.639808000000	0.794762000000
S	3.374589000000	0.383359000000	2.032048000000
C	4.689164000000	-0.379955000000	1.112040000000
C	5.058972000000	-1.684787000000	1.421429000000
C	6.047570000000	-2.294660000000	0.663071000000
C	6.664419000000	-1.617713000000	-0.395372000000
C	6.288553000000	-0.299388000000	-0.663603000000
C	5.305378000000	0.333327000000	0.090233000000
C	7.706215000000	-2.314541000000	-1.230403000000
O	2.967553000000	-0.520139000000	3.099895000000
O	3.770247000000	1.752388000000	2.311634000000
C	-1.298332000000	-1.197824000000	0.807584000000
C	-1.118931000000	-0.280604000000	1.847407000000
C	-1.652758000000	-0.529301000000	3.110889000000
C	-2.372316000000	-1.695171000000	3.350263000000
C	-2.568008000000	-2.610030000000	2.316245000000
C	-2.030592000000	-2.365940000000	1.057226000000
H	0.069000000000	1.070539000000	-0.301832000000
H	3.169639000000	-1.104505000000	-2.011109000000
H	4.085825000000	-3.386065000000	-2.382142000000
H	3.635252000000	-5.187448000000	-0.738789000000
H	2.288792000000	-4.708659000000	1.289345000000
H	1.428151000000	-2.407888000000	1.692256000000
H	4.560208000000	-2.201409000000	2.234323000000
H	6.339240000000	-3.317704000000	0.885808000000
H	6.773250000000	0.240257000000	-1.471948000000
H	5.017593000000	1.360957000000	-0.109920000000
H	8.504867000000	-2.720582000000	-0.603291000000
H	7.262369000000	-3.152762000000	-1.777371000000
H	8.154439000000	-1.635080000000	-1.957876000000
H	-0.534877000000	0.619829000000	1.690456000000
H	-1.488308000000	0.188267000000	3.907869000000
H	-2.772732000000	-1.895622000000	4.339503000000
H	-3.130996000000	-3.520959000000	2.493263000000
H	-2.169969000000	-3.091927000000	0.260671000000
C	-1.682757000000	-0.543250000000	-1.694750000000
C	-2.565659000000	0.600648000000	-1.178401000000
N	-3.864647000000	0.152620000000	-1.146799000000
C	-3.962290000000	-1.152277000000	-1.650217000000
C	-5.094672000000	-1.940099000000	-1.786055000000
C	-4.925586000000	-3.214537000000	-2.334707000000
C	-3.672331000000	-3.674841000000	-2.730477000000
C	-2.694697000000	-1.601647000000	-2.035587000000
C	-2.542122000000	-2.864277000000	-2.578815000000
O	-2.191733000000	1.708537000000	-0.845283000000
C	-0.744512000000	-0.081341000000	-2.821881000000
O	1.580572000000	0.616781000000	-2.728946000000
C	-4.981364000000	0.971243000000	-0.723128000000
C	-5.716082000000	0.393215000000	0.467652000000
C	-7.109043000000	0.429896000000	0.514384000000
C	-7.791820000000	-0.078929000000	1.616344000000
C	-7.081821000000	-0.634434000000	2.676205000000
C	-5.689932000000	-0.677751000000	2.629388000000
C	-5.007222000000	-0.166805000000	1.530874000000
H	-3.571003000000	-4.667127000000	-3.156081000000
H	-5.792461000000	-3.856870000000	-2.450615000000
H	-6.067997000000	-1.588335000000	-1.461776000000
H	-1.560563000000	-3.224300000000	-2.878194000000
H	-0.555971000000	-0.895137000000	-3.530846000000
H	-7.662760000000	0.859010000000	-0.317677000000
H	-8.876336000000	-0.046359000000	1.642642000000
H	-7.610479000000	-1.036569000000	3.534558000000
H	-5.127667000000	-1.113422000000	3.449110000000
H	-3.920634000000	-0.206235000000	1.495355000000
H	-5.674040000000	1.100799000000	-1.564037000000
H	-4.551376000000	1.949225000000	-0.484017000000

H	-1.124968000000	0.772234000000	-3.388711000000
H	-0.268618000000	-1.934300000000	-0.897805000000
N	0.759100000000	3.672152000000	-0.600176000000
C	1.981374000000	3.972801000000	-1.340261000000
C	0.932383000000	3.756633000000	0.865913000000
C	-0.442270000000	4.309901000000	-1.161691000000
C	3.150554000000	3.068330000000	-0.963555000000
H	2.292195000000	5.027507000000	-1.234377000000
H	1.774852000000	3.815147000000	-2.402617000000
H	4.016542000000	3.318570000000	-1.585270000000
H	3.443622000000	3.169865000000	0.086366000000
H	2.880854000000	2.025782000000	-1.151780000000
C	-0.382652000000	3.505646000000	1.601167000000
C	1.610921000000	5.024950000000	1.408649000000
H	1.591911000000	2.923149000000	1.125612000000
H	-0.936105000000	2.674586000000	1.153075000000
H	-0.170668000000	3.265723000000	2.647488000000
H	-1.036833000000	4.384912000000	1.589362000000
H	2.587170000000	5.195054000000	0.946388000000
H	1.006220000000	5.924907000000	1.267363000000
H	1.782366000000	4.899034000000	2.482185000000
C	-0.662349000000	3.864995000000	-2.610338000000
C	-0.495343000000	5.843653000000	-1.077177000000
H	-1.285693000000	3.907945000000	-0.596988000000
H	-0.406468000000	2.808051000000	-2.715874000000
H	-1.713967000000	3.998486000000	-2.879962000000
H	-0.061365000000	4.442039000000	-3.320710000000
H	-0.594991000000	6.191766000000	-0.046682000000
H	0.403814000000	6.297308000000	-1.507462000000
H	-1.357625000000	6.219495000000	-1.638091000000

INTb12''

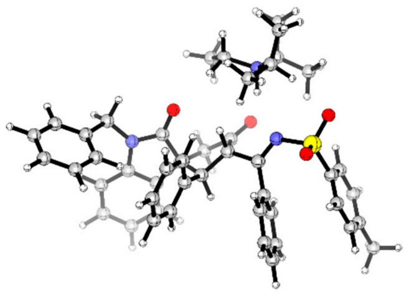

Zero-point correction= 0.908859 (Hartree/Particle)

Thermal correction to Energy= 0.959316

Thermal correction to Enthalpy= 0.960260

Thermal correction to Gibbs Free Energy= 0.822804

Sum of electronic and zero-point Energies= -2683.630319

Sum of electronic and thermal Energies= -2683.579863

Sum of electronic and thermal Enthalpies= -2683.578918

Sum of electronic and thermal Free Energies= -2683.716374

E(RM062X) = -2685.11030910

C	0.274679000000	0.106282000000	-0.394917000000
H	-0.077111000000	0.980040000000	0.167633000000
C	1.574776000000	-0.336452000000	0.232422000000
C	0.433066000000	0.681077000000	-1.810776000000
C	-0.863051000000	-0.909507000000	-0.510063000000
N	2.346737000000	0.619236000000	0.592727000000
S	3.844132000000	0.353692000000	1.397111000000
C	1.808570000000	-1.802183000000	0.362150000000
C	4.702508000000	-0.931423000000	0.538987000000
O	3.520062000000	-0.082342000000	2.745527000000
O	4.552782000000	1.603957000000	1.163696000000
C	2.159115000000	-2.546299000000	-0.768632000000
C	1.616459000000	-2.427623000000	1.594108000000
C	-0.719015000000	0.190590000000	-2.664338000000
O	1.309017000000	1.444947000000	-2.142193000000
C	-1.585852000000	-1.238902000000	0.777224000000

C	-1.755138000000	-0.309607000000	-1.648356000000
H	-0.456065000000	-1.844890000000	-0.916591000000
H	-0.354988000000	-0.653632000000	-3.265804000000
H	-1.073534000000	0.964968000000	-3.347753000000
C	-1.667943000000	-0.341022000000	1.846366000000
C	-2.202960000000	-2.488322000000	0.900465000000
C	-2.559139000000	0.869854000000	-1.084900000000
C	-2.832105000000	-1.234899000000	-2.136004000000
C	2.354885000000	-3.917074000000	-0.653823000000
H	2.317012000000	-2.046327000000	-1.721700000000
C	1.793885000000	-3.806055000000	1.693756000000
H	1.342129000000	-1.837379000000	2.462701000000
C	2.167106000000	-4.547329000000	0.576617000000
H	2.653831000000	-4.494016000000	-1.522857000000
H	2.311666000000	-5.619306000000	0.662019000000
H	1.642894000000	-4.296990000000	2.649124000000
C	4.900328000000	-2.151891000000	1.175288000000
C	5.143358000000	-0.696787000000	-0.760269000000
C	5.535030000000	-3.168845000000	0.474257000000
H	4.544039000000	-2.294112000000	2.189931000000
C	5.767851000000	-1.730848000000	-1.445776000000
H	5.002196000000	0.275826000000	-1.222258000000
C	5.968321000000	-2.977764000000	-0.841635000000
H	5.690212000000	-4.131404000000	0.953067000000
C	6.666767000000	-4.086004000000	-1.582937000000
H	6.115025000000	-1.568469000000	-2.461995000000
H	6.274866000000	-5.063397000000	-1.292066000000
H	6.556412000000	-3.974449000000	-2.663577000000
H	7.737602000000	-4.080263000000	-1.356144000000
C	-2.331936000000	-0.694152000000	3.019075000000
H	-1.226965000000	0.649078000000	1.775020000000
C	-2.873668000000	-2.838830000000	2.067743000000
H	-2.150774000000	-3.190925000000	0.072776000000
C	-2.929829000000	-1.945444000000	3.135411000000
H	-2.382836000000	0.012356000000	3.840689000000
H	-3.438339000000	-2.222587000000	4.053218000000
H	-3.342565000000	-3.814091000000	2.147185000000
N	-3.881215000000	0.560190000000	-1.164135000000
O	-2.077833000000	1.906218000000	-0.642540000000
C	-4.071807000000	-0.687020000000	-1.796080000000
C	-2.765829000000	-2.460102000000	-2.775484000000
C	-4.944021000000	1.436782000000	-0.711290000000
C	-5.265701000000	-1.330733000000	-2.077051000000
C	-5.828471000000	0.811306000000	0.347806000000
H	-5.549392000000	1.736824000000	-1.575073000000
H	-4.443717000000	2.330872000000	-0.326204000000
C	-5.185237000000	-2.565297000000	-2.726828000000
H	-6.220530000000	-0.903240000000	-1.791012000000
C	-3.958435000000	-3.126207000000	-3.074040000000
H	-6.101115000000	-3.096714000000	-2.961767000000
H	-3.928138000000	-4.085041000000	-3.579217000000
H	-1.807630000000	-2.899800000000	-3.042455000000
C	-7.139876000000	1.267322000000	0.485581000000
C	-5.356059000000	-0.187680000000	1.198417000000
C	-7.967355000000	0.744871000000	1.474260000000
H	-7.515817000000	2.037051000000	-0.184129000000
C	-6.187736000000	-0.714426000000	2.182629000000
H	-4.342732000000	-0.565382000000	1.087635000000
C	-7.491839000000	-0.249103000000	2.326023000000
H	-8.984645000000	1.108607000000	1.574133000000
H	-8.138324000000	-0.662892000000	3.092770000000
H	-5.810310000000	-1.495168000000	2.835649000000
N	1.543765000000	3.696190000000	-0.154423000000
C	0.111996000000	4.165181000000	-0.266141000000
C	2.446508000000	4.243226000000	-1.257445000000
C	2.084230000000	3.778684000000	1.274572000000

C	-0.430750000000	4.235003000000	-1.684504000000
H	0.055443000000	5.138633000000	0.225924000000
H	-0.490985000000	3.456833000000	0.303249000000
H	-1.506005000000	4.406447000000	-1.610474000000
H	0.010739000000	5.043023000000	-2.270452000000
H	-0.289629000000	3.290739000000	-2.212504000000
C	3.824949000000	3.610492000000	-1.161202000000
C	2.487609000000	5.767101000000	-1.345285000000
H	1.968996000000	3.851710000000	-2.158439000000
H	3.749098000000	2.520899000000	-1.118432000000
H	4.389899000000	3.885663000000	-2.055061000000
H	4.387277000000	3.947786000000	-0.287114000000
H	1.526352000000	6.238013000000	-1.128187000000
H	3.240511000000	6.202185000000	-0.688800000000
H	2.756245000000	6.033427000000	-2.370571000000
C	1.106447000000	3.115561000000	2.240949000000
C	2.423063000000	5.195879000000	1.717425000000
H	2.993962000000	3.176304000000	1.244917000000
H	0.761773000000	2.145858000000	1.871838000000
H	1.637020000000	2.927604000000	3.177294000000
H	0.244473000000	3.751230000000	2.461275000000
H	3.332070000000	5.571334000000	1.249426000000
H	1.610054000000	5.905230000000	1.538121000000
H	2.601233000000	5.171291000000	2.794847000000
H	1.549241000000	2.681726000000	-0.351177000000

INTb12

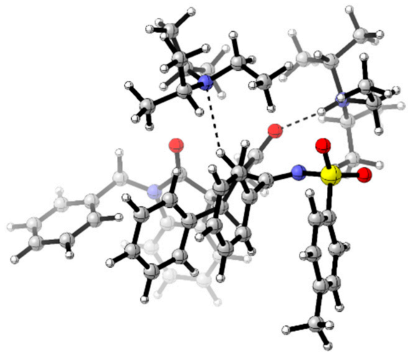

Zero-point correction= 1.175206 (Hartree/Particle)

Thermal correction to Energy= 1.239393

Thermal correction to Enthalpy= 1.240337

Thermal correction to Gibbs Free Energy= 1.074916

Sum of electronic and zero-point Energies= -3054.247250

Sum of electronic and thermal Energies= -3054.183063

Sum of electronic and thermal Enthalpies= -3054.182118

Sum of electronic and thermal Free Energies= -3054.347539

E(RM062X) = -3056.07731292

N	0.289944000000	-2.615416000000	-2.422690000000
H	-0.143673000000	-0.532050000000	-0.760383000000
C	0.147996000000	-0.071056000000	0.201629000000
C	0.560779000000	-1.265472000000	1.051296000000
C	1.243750000000	0.942171000000	-0.073241000000
C	-1.084681000000	0.522132000000	0.929503000000
N	2.396453000000	0.733642000000	0.440617000000
C	0.853174000000	2.082195000000	-0.958865000000
C	0.194740000000	3.195515000000	-0.431644000000
C	-0.231614000000	4.209709000000	-1.278615000000
C	-0.008887000000	4.111490000000	-2.651742000000
C	0.659625000000	3.008132000000	-3.173746000000
C	1.097936000000	1.990495000000	-2.328633000000
S	3.759608000000	1.694504000000	0.062282000000
C	3.336530000000	3.405189000000	0.182869000000
C	3.294879000000	4.172422000000	-0.977316000000

C	2.926170000000	5.505665000000	-0.873364000000
C	2.594778000000	6.070374000000	0.363881000000
C	2.666750000000	5.274467000000	1.512011000000
C	3.045547000000	3.939601000000	1.433540000000
C	2.161238000000	7.509357000000	0.444644000000
O	4.109309000000	1.381559000000	-1.317553000000
O	4.679239000000	1.376226000000	1.147879000000
C	-2.089896000000	1.291297000000	0.096269000000
C	-2.195895000000	1.133235000000	-1.287239000000
C	-3.083217000000	1.912679000000	-2.024089000000
C	-3.884319000000	2.855323000000	-1.385260000000
C	-3.809596000000	2.998081000000	-0.001553000000
C	-2.917309000000	2.222085000000	0.732479000000
H	0.021096000000	3.273175000000	0.637800000000
H	-0.746065000000	5.071632000000	-0.866387000000
H	-0.353121000000	4.899004000000	-3.314053000000
H	0.845283000000	2.936006000000	-4.240220000000
H	1.642516000000	1.137407000000	-2.722200000000
H	3.530522000000	3.715937000000	-1.932893000000
H	2.886251000000	6.119942000000	-1.768449000000
H	2.435705000000	5.710852000000	2.479183000000
H	3.123722000000	3.325017000000	2.325280000000
H	2.848504000000	8.156281000000	-0.106371000000
H	1.168224000000	7.635754000000	0.001266000000
H	2.116143000000	7.856881000000	1.478113000000
H	-1.556791000000	0.431516000000	-1.812036000000
H	-3.136876000000	1.787420000000	-3.100792000000
H	-4.562986000000	3.474919000000	-1.962999000000
H	-4.438854000000	3.720521000000	0.508046000000
H	-2.850191000000	2.350908000000	1.809961000000
C	-1.693975000000	-0.698428000000	1.702226000000
C	-2.536704000000	-1.529803000000	0.728783000000
N	-3.831704000000	-1.515010000000	1.162013000000
C	-3.950213000000	-0.799076000000	2.367498000000
C	-5.091215000000	-0.567154000000	3.118683000000
C	-4.940174000000	0.163662000000	4.300202000000
C	-3.696088000000	0.639959000000	4.707814000000
C	-2.694539000000	-0.321043000000	2.757144000000
C	-2.558779000000	0.399464000000	3.930282000000
O	-2.101315000000	-2.100378000000	-0.256302000000
C	-0.463912000000	-1.497559000000	2.140518000000
O	1.551750000000	-1.941569000000	0.883228000000
C	-4.914115000000	-2.211701000000	0.492346000000
C	-6.030276000000	-1.288108000000	0.052983000000
C	-7.351671000000	-1.731793000000	0.098073000000
C	-8.386530000000	-0.906579000000	-0.333984000000
C	-8.105061000000	0.372375000000	-0.805050000000
C	-6.786730000000	0.820704000000	-0.842363000000
C	-5.751366000000	-0.004804000000	-0.416250000000
H	-3.610443000000	1.200324000000	5.632064000000
H	-5.814649000000	0.365018000000	4.909750000000
H	-6.062259000000	-0.924281000000	2.793845000000
H	-1.587837000000	0.775034000000	4.245349000000
H	-0.051277000000	-1.070253000000	3.065788000000
H	-7.572904000000	-2.728528000000	0.472311000000
H	-9.411353000000	-1.260983000000	-0.295996000000
H	-8.910640000000	1.019139000000	-1.136726000000
H	-6.559203000000	1.819033000000	-1.202582000000
H	-4.724441000000	0.351246000000	-0.441850000000
H	-5.308454000000	-2.985670000000	1.161804000000
H	-4.453693000000	-2.711701000000	-0.365721000000
H	-0.642026000000	-2.562113000000	2.306804000000
H	-0.712297000000	1.197458000000	1.715077000000
C	1.723256000000	-2.884958000000	-2.472308000000
C	-0.165109000000	-1.693671000000	-3.486777000000
C	-0.522696000000	-3.832234000000	-2.219212000000

C	2.583735000000	-1.631108000000	-2.365211000000
H	2.020021000000	-3.445042000000	-3.375689000000
H	1.964256000000	-3.527350000000	-1.617777000000
H	3.644132000000	-1.903826000000	-2.381872000000
H	2.432664000000	-0.940874000000	-3.199531000000
H	2.377251000000	-1.099784000000	-1.430587000000
C	-1.689406000000	-1.587139000000	-3.546066000000
C	0.386525000000	-1.957345000000	-4.897814000000
H	0.206400000000	-0.698427000000	-3.188554000000
H	-2.127388000000	-1.491937000000	-2.549628000000
H	-1.964414000000	-0.709349000000	-4.139092000000
H	-2.134174000000	-2.460585000000	-4.033091000000
H	1.478660000000	-1.937929000000	-4.933936000000
H	0.055891000000	-2.922267000000	-5.289230000000
H	0.022084000000	-1.180946000000	-5.577140000000
C	-0.173494000000	-4.507458000000	-0.891065000000
C	-0.475307000000	-4.866332000000	-3.353469000000
H	-1.550900000000	-3.486515000000	-2.108555000000
H	-0.031769000000	-3.754384000000	-0.112087000000
H	-0.994385000000	-5.164798000000	-0.592213000000
H	0.727014000000	-5.127105000000	-0.962847000000
H	-0.926467000000	-4.491175000000	-4.274901000000
H	0.552489000000	-5.172271000000	-3.575823000000
H	-1.028275000000	-5.764047000000	-3.060444000000
H	3.560625000000	-1.904177000000	0.774768000000
N	4.338377000000	-2.439881000000	1.195938000000
C	5.578292000000	-1.601214000000	1.020232000000
C	4.385051000000	-3.738545000000	0.384354000000
C	3.903204000000	-2.566050000000	2.653316000000
C	5.843988000000	-1.253778000000	-0.433672000000
H	6.406858000000	-2.146280000000	1.474285000000
H	5.408548000000	-0.677532000000	1.570382000000
H	6.641621000000	-0.508691000000	-0.463661000000
H	6.155569000000	-2.110739000000	-1.035147000000
H	4.969348000000	-0.789051000000	-0.896040000000
C	3.208905000000	-4.651068000000	0.711991000000
C	5.714480000000	-4.479891000000	0.486225000000
H	4.264223000000	-3.383040000000	-0.644537000000
H	2.255953000000	-4.119036000000	0.690915000000
H	3.173846000000	-5.436743000000	-0.047198000000
H	3.331955000000	-5.139397000000	1.682876000000
H	6.566062000000	-3.888634000000	0.148256000000
H	5.917615000000	-4.841486000000	1.494987000000
H	5.648493000000	-5.354001000000	-0.165998000000
C	3.659880000000	-1.174317000000	3.232798000000
C	4.867665000000	-3.375725000000	3.507306000000
H	2.940582000000	-3.075171000000	2.588691000000
H	3.139462000000	-0.523998000000	2.523100000000
H	3.041454000000	-1.285746000000	4.127150000000
H	4.588622000000	-0.683244000000	3.533271000000
H	4.883014000000	-4.433572000000	3.243303000000
H	5.885951000000	-2.980330000000	3.465187000000
H	4.535512000000	-3.303712000000	4.545860000000

TSb8'

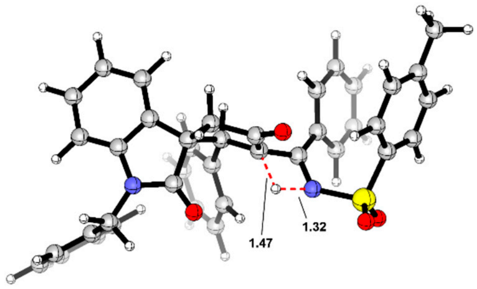

Frequency -1787.4137

Zero-point correction= 0.620833 (Hartree/Particle)

Thermal correction to Energy= 0.658959

Thermal correction to Enthalpy= 0.659903

Thermal correction to Gibbs Free Energy= 0.547585

Sum of electronic and zero-point Energies= -2312.517085

Sum of electronic and thermal Energies= -2312.478960

Sum of electronic and thermal Enthalpies= -2312.478015

Sum of electronic and thermal Free Energies= -2312.590334

E(RM062X) = -2313.63057160

C	0.683357000000	-0.463575000000	-0.526791000000
H	0.935131000000	0.398667000000	-1.690168000000
C	1.815962000000	0.368671000000	-0.217408000000
C	0.871523000000	-1.649432000000	-1.394742000000
C	-0.511671000000	-0.690936000000	0.367768000000
N	2.044230000000	1.023791000000	-1.343321000000
S	3.593473000000	1.635285000000	-1.839191000000
C	2.537611000000	0.489138000000	1.052858000000
C	4.747189000000	0.510597000000	-1.094071000000
O	3.765270000000	2.945622000000	-1.231576000000
O	3.584822000000	1.465510000000	-3.276378000000
C	2.886119000000	-0.673214000000	1.748346000000
C	2.869710000000	1.749730000000	1.561395000000
C	-0.402095000000	-2.489246000000	-1.273344000000
O	1.832371000000	-1.957602000000	-2.068956000000
C	-1.193990000000	0.552118000000	0.896253000000
C	-1.414705000000	-1.613405000000	-0.523064000000
H	-0.223078000000	-1.303621000000	1.237557000000
H	-0.155785000000	-3.380467000000	-0.683045000000
H	-0.738991000000	-2.819317000000	-2.258203000000
C	-1.166123000000	1.760588000000	0.191005000000
C	-1.873231000000	0.498927000000	2.116845000000
C	-2.212889000000	-0.751248000000	-1.515302000000
C	-2.500666000000	-2.307673000000	0.249953000000
C	3.602978000000	-0.575274000000	2.934133000000
H	2.625969000000	-1.641014000000	1.328192000000
C	3.561146000000	1.838743000000	2.763999000000
H	2.588997000000	2.640055000000	1.007393000000
C	3.935802000000	0.680137000000	3.441717000000
H	3.898426000000	-1.474024000000	3.465317000000
H	4.485908000000	0.755089000000	4.374526000000
H	3.813626000000	2.812807000000	3.169501000000
C	5.598527000000	0.979488000000	-0.097891000000
C	4.711063000000	-0.829303000000	-1.464455000000
C	6.425097000000	0.072208000000	0.550061000000
H	5.584821000000	2.031088000000	0.167713000000
C	5.544842000000	-1.720342000000	-0.797272000000
H	4.013204000000	-1.175542000000	-2.220795000000
C	6.403259000000	-1.286782000000	0.216365000000
H	7.087979000000	0.418694000000	1.338428000000
C	7.295977000000	-2.255296000000	0.946444000000
H	5.519492000000	-2.772923000000	-1.063902000000
H	7.175533000000	-2.153869000000	2.029141000000
H	7.072631000000	-3.288020000000	0.671870000000
H	8.348352000000	-2.063700000000	0.715594000000
C	-1.783641000000	2.894671000000	0.711748000000
H	-0.668766000000	1.811353000000	-0.772964000000
C	-2.494020000000	1.631992000000	2.635822000000
H	-1.908191000000	-0.439501000000	2.665094000000
C	-2.440563000000	2.836806000000	1.938386000000
H	-1.749699000000	3.825463000000	0.155050000000
H	-2.911227000000	3.725884000000	2.346779000000
H	-3.012041000000	1.574101000000	3.588080000000
N	-3.552186000000	-0.911871000000	-1.227854000000
O	-1.762368000000	-0.056937000000	-2.402661000000
C	-3.741943000000	-1.833175000000	-0.189344000000
C	-2.438110000000	-3.237476000000	1.271474000000
C	-4.607956000000	-0.246891000000	-1.959028000000
C	-4.934653000000	-2.268446000000	0.368220000000

C	-5.483655000000	0.621659000000	-1.078519000000
H	-5.224034000000	-0.995930000000	-2.472784000000
H	-4.100987000000	0.351771000000	-2.722921000000
C	-4.855582000000	-3.211849000000	1.396320000000
H	-5.888720000000	-1.879702000000	0.029106000000
C	-3.629448000000	-3.694141000000	1.846155000000
H	-5.772318000000	-3.570715000000	1.852803000000
H	-3.596960000000	-4.425700000000	2.646174000000
H	-1.478821000000	-3.610614000000	1.622459000000
C	-6.833616000000	0.784265000000	-1.389252000000
C	-4.954637000000	1.271563000000	0.036293000000
C	-7.646288000000	1.596066000000	-0.602373000000
H	-7.252245000000	0.271292000000	-2.252075000000
C	-5.768674000000	2.077299000000	0.826064000000
H	-3.906763000000	1.139229000000	0.291923000000
C	-7.114671000000	2.243263000000	0.509608000000
H	-8.695475000000	1.715586000000	-0.853451000000
H	-7.747912000000	2.870067000000	1.129293000000
H	-5.342712000000	2.571419000000	1.693900000000

TSb8'''

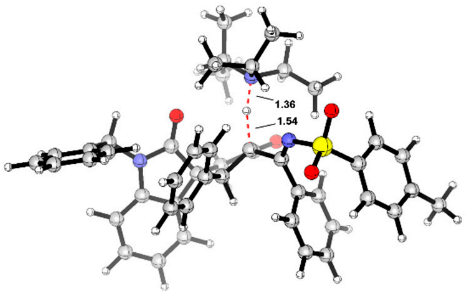

Frequency -1358.3394

Zero-point correction= 0.890532 (Hartree/Particle)

Thermal correction to Energy= 0.940914

Thermal correction to Enthalpy= 0.941858

Thermal correction to Gibbs Free Energy= 0.805067

Sum of electronic and zero-point Energies= -2683.173688

Sum of electronic and thermal Energies= -2683.123306

Sum of electronic and thermal Enthalpies= -2683.122362

Sum of electronic and thermal Free Energies= -2683.259153

E(RM062X) = -2684.64074025

N	0.519837000000	3.143960000000	-0.811047000000
H	0.328428000000	1.802056000000	-0.756644000000
C	0.462046000000	0.271010000000	-0.743821000000
C	0.585721000000	0.212672000000	-2.202028000000
C	1.684781000000	-0.094095000000	0.059488000000
C	-0.641819000000	-0.769864000000	-0.423302000000
N	1.965712000000	0.633782000000	1.075386000000
C	2.354833000000	-1.393650000000	-0.267297000000
C	3.177987000000	-1.553496000000	-1.385521000000
C	3.751591000000	-2.795471000000	-1.639671000000
C	3.478021000000	-3.883606000000	-0.812158000000
C	2.635721000000	-3.728371000000	0.284919000000
C	2.083004000000	-2.482554000000	0.568647000000
S	3.234475000000	0.393063000000	2.172182000000
C	4.650892000000	-0.266009000000	1.318156000000
C	5.003434000000	-1.597635000000	1.511013000000
C	6.078430000000	-2.114027000000	0.802230000000
C	6.802371000000	-1.317985000000	-0.091968000000
C	6.448673000000	0.025281000000	-0.235761000000
C	5.378361000000	0.562736000000	0.471796000000
C	7.932323000000	-1.913981000000	-0.889293000000
O	2.795347000000	-0.584930000000	3.157952000000
O	3.573217000000	1.754422000000	2.562613000000
C	-1.272822000000	-0.780576000000	0.952688000000
C	-1.089241000000	0.264710000000	1.856807000000
C	-1.642984000000	0.212259000000	3.135284000000

C	-2.382191000000	-0.896925000000	3.531339000000
C	-2.567028000000	-1.953061000000	2.640165000000
C	-2.010964000000	-1.899595000000	1.367563000000
H	3.364672000000	-0.710120000000	-2.041828000000
H	4.410107000000	-2.914275000000	-2.494640000000
H	3.920373000000	-4.852062000000	-1.024395000000
H	2.414804000000	-4.573085000000	0.929692000000
H	1.448502000000	-2.340652000000	1.440394000000
H	4.427638000000	-2.207292000000	2.198297000000
H	6.355293000000	-3.156629000000	0.934502000000
H	7.021736000000	0.659806000000	-0.905728000000
H	5.116995000000	1.611736000000	0.381835000000
H	8.608777000000	-2.485459000000	-0.248000000000
H	7.543853000000	-2.600868000000	-1.648572000000
H	8.512094000000	-1.142131000000	-1.399310000000
H	-0.465804000000	1.100793000000	1.570512000000
H	-1.470707000000	1.030698000000	3.827452000000
H	-2.796057000000	-0.949892000000	4.533962000000
H	-3.138131000000	-2.826110000000	2.939933000000
H	-2.137369000000	-2.741713000000	0.692731000000
C	-1.634828000000	-0.674307000000	-1.632736000000
C	-2.667760000000	0.420648000000	-1.366454000000
N	-3.906887000000	-0.166892000000	-1.279850000000
C	-3.829805000000	-1.546398000000	-1.514681000000
C	-4.852881000000	-2.480925000000	-1.501913000000
C	-4.509956000000	-3.806942000000	-1.782050000000
C	-3.196065000000	-4.171816000000	-2.064123000000
C	-2.502429000000	-1.896357000000	-1.781252000000
C	-2.178511000000	-3.210816000000	-2.061993000000
O	-2.455998000000	1.611840000000	-1.234124000000
C	-0.700928000000	-0.357410000000	-2.808617000000
O	1.522260000000	0.596751000000	-2.891732000000
C	-5.129239000000	0.574018000000	-1.054149000000
C	-5.791534000000	0.243075000000	0.265521000000
C	-7.168052000000	0.030790000000	0.327073000000
C	-7.788136000000	-0.235444000000	1.545388000000
C	-7.029647000000	-0.294017000000	2.710374000000
C	-5.652550000000	-0.089167000000	2.651059000000
C	-5.032596000000	0.177110000000	1.435008000000
H	-2.959159000000	-5.207704000000	-2.281209000000
H	-5.286782000000	-4.564751000000	-1.776237000000
H	-5.872705000000	-2.198239000000	-1.262952000000
H	-1.148592000000	-3.495688000000	-2.264088000000
H	-0.419274000000	-1.282470000000	-3.325812000000
H	-7.758699000000	0.073735000000	-0.585377000000
H	-8.860200000000	-0.400913000000	1.581949000000
H	-7.508617000000	-0.501620000000	3.661936000000
H	-5.051679000000	-0.136958000000	3.553182000000
H	-3.955572000000	0.331960000000	1.392936000000
H	-5.822403000000	0.381657000000	-1.882346000000
H	-4.841090000000	1.629237000000	-1.088548000000
H	-1.135567000000	0.311980000000	-3.555363000000
H	-0.193419000000	-1.769828000000	-0.547654000000
C	1.827088000000	3.427227000000	-1.475511000000
C	0.528128000000	3.509052000000	0.659708000000
C	-0.587659000000	3.811757000000	-1.591600000000
C	3.042558000000	2.699814000000	-0.921883000000
H	1.998762000000	4.507698000000	-1.433961000000
H	1.716383000000	3.141820000000	-2.521905000000
H	3.934632000000	3.168929000000	-1.349446000000
H	3.116340000000	2.746279000000	0.167435000000
H	3.035007000000	1.655795000000	-1.237044000000
C	-0.893208000000	3.576099000000	1.224089000000
C	1.317552000000	4.766049000000	1.039136000000
H	1.039670000000	2.665957000000	1.133438000000
H	-1.527179000000	2.768536000000	0.849567000000

H -0.836410000000 3.487498000000 2.312947000000

H -1.380956000000 4.528723000000 0.997160000000

H 2.377918000000 4.688650000000 0.792898000000

H 0.922323000000 5.679480000000 0.590706000000

H 1.253198000000 4.873819000000 2.125361000000

C -0.645678000000 3.304700000000 -3.032503000000

C -0.550781000000 5.343097000000 -1.606179000000

H -1.503822000000 3.495070000000 -1.105373000000

H -0.386506000000 2.251180000000 -3.107869000000

H -1.666005000000 3.431388000000 -3.402887000000

H 0.024016000000 3.866940000000 -3.690761000000

H -0.715304000000 5.787350000000 -0.624335000000

H 0.381196000000 5.739109000000 -2.019235000000

H -1.360913000000 5.685345000000 -2.257229000000

TSb8''

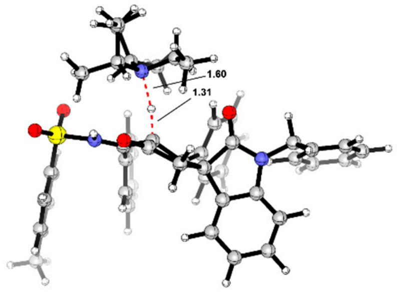

Frequency -1141.1714

Zero-point correction= 0.903368 (Hartree/Particle)

Thermal correction to Energy= 0.953557

Thermal correction to Enthalpy= 0.954501

Thermal correction to Gibbs Free Energy= 0.819333

Sum of electronic and zero-point Energies= -2683.566878

Sum of electronic and thermal Energies= -2683.516689

Sum of electronic and thermal Enthalpies= -2683.515745

Sum of electronic and thermal Free Energies= -2683.650914

E(RM062X) = -2685.03910999

C 0.485707000000 0.719181000000 -0.697482000000

H 0.573832000000 1.916846000000 -0.177560000000

C 1.758891000000 0.178423000000 -0.244580000000

C 0.468869000000 1.219487000000 -2.129511000000

C -0.708009000000 -0.267569000000 -0.606551000000

N 2.876957000000 0.682964000000 -0.709638000000

S 4.523045000000 0.496613000000 -0.087700000000

C 1.817204000000 -0.977202000000 0.692037000000

C 4.847021000000 -1.224113000000 -0.202248000000

O 4.448285000000 0.901483000000 1.299088000000

O 5.261760000000 1.231994000000 -1.086484000000

C 1.797020000000 -2.254904000000 0.121471000000

C 1.869946000000 -0.815677000000 2.074515000000

C -0.853276000000 0.881150000000 -2.783968000000

O 1.391277000000 1.794148000000 -2.670280000000

C -1.342785000000 -0.598696000000 0.729175000000

C -1.711815000000 0.147361000000 -1.739820000000

H -0.305752000000 -1.218588000000 -0.975694000000

H -0.636851000000 0.244075000000 -3.649086000000

H -1.318676000000 1.794697000000 -3.166285000000

C -1.837171000000 0.372087000000 1.606042000000

C -1.467277000000 -1.944736000000 1.088277000000

C -2.820626000000 1.050174000000 -1.182251000000

C -2.495921000000 -1.040554000000 -2.238234000000

C 1.814186000000 -3.373470000000 0.945057000000

H 1.793103000000 -2.371811000000 -0.959459000000

C 1.873881000000 -1.944199000000 2.890949000000

H 1.925354000000 0.177118000000 2.505105000000

C 1.838711000000 -3.217624000000 2.330010000000

H 1.809893000000 -4.365273000000 0.504863000000

H 1.840260000000 -4.091769000000 2.972538000000

H 1.907085000000 -1.822942000000 3.968150000000

C	5.000391000000	-1.961067000000	0.967433000000
C	4.951464000000	-1.797298000000	-1.468869000000
C	5.247610000000	-3.323390000000	0.854321000000
H	4.911250000000	-1.476637000000	1.934063000000
C	5.183957000000	-3.161561000000	-1.552866000000
H	4.861082000000	-1.186599000000	-2.362314000000
C	5.335111000000	-3.941044000000	-0.397280000000
H	5.368161000000	-3.918678000000	1.754482000000
C	5.624208000000	-5.412934000000	-0.507444000000
H	5.266743000000	-3.631555000000	-2.528220000000
H	5.311553000000	-5.948815000000	0.390766000000
H	5.120891000000	-5.853305000000	-1.370836000000
H	6.699109000000	-5.576631000000	-0.634384000000
C	-2.386541000000	0.009023000000	2.832344000000
H	-1.814402000000	1.416847000000	1.320350000000
C	-2.026105000000	-2.311746000000	2.309404000000
H	-1.111226000000	-2.712554000000	0.406150000000
C	-2.473421000000	-1.332805000000	3.192843000000
H	-2.761153000000	0.777915000000	3.500137000000
H	-2.901893000000	-1.613773000000	4.149527000000
H	-2.106974000000	-3.362300000000	2.569510000000
N	-4.008313000000	0.381859000000	-1.275138000000
O	-2.660005000000	2.168087000000	-0.726623000000
C	-3.843147000000	-0.857703000000	-1.918786000000
C	-2.086020000000	-2.192790000000	-2.885057000000
C	-5.269019000000	0.920306000000	-0.804574000000
C	-4.809220000000	-1.800043000000	-2.233438000000
C	-5.946728000000	0.049084000000	0.234523000000
H	-5.938077000000	1.067546000000	-1.661196000000
H	-5.031770000000	1.907014000000	-0.394435000000
C	-4.382797000000	-2.956343000000	-2.891648000000
H	-5.850401000000	-1.652021000000	-1.968888000000
C	-3.043146000000	-3.156663000000	-3.215823000000
H	-5.114827000000	-3.713271000000	-3.152426000000
H	-2.742633000000	-4.062706000000	-3.729866000000
H	-1.039829000000	-2.347239000000	-3.141793000000
C	-7.325959000000	0.166558000000	0.413279000000
C	-5.228880000000	-0.847228000000	1.025739000000
C	-7.979478000000	-0.590438000000	1.379913000000
H	-7.892749000000	0.856014000000	-0.207833000000
C	-5.885308000000	-1.609466000000	1.988303000000
H	-4.156983000000	-0.961532000000	0.887197000000
C	-7.259053000000	-1.482744000000	2.170491000000
H	-9.051808000000	-0.488649000000	1.510792000000
H	-7.768370000000	-2.078715000000	2.920506000000
H	-5.313957000000	-2.306709000000	2.593624000000
N	0.977562000000	3.399883000000	0.278632000000
C	-0.389883000000	3.985730000000	0.399322000000
C	1.818063000000	4.254493000000	-0.648791000000
C	1.606958000000	3.128950000000	1.615403000000
C	-0.993108000000	4.440551000000	-0.924195000000
H	-0.384015000000	4.815946000000	1.117441000000
H	-1.040678000000	3.211285000000	0.806847000000
H	-2.059376000000	4.611533000000	-0.773410000000
H	-0.544240000000	5.355235000000	-1.313352000000
H	-0.910270000000	3.656896000000	-1.680756000000
C	3.268341000000	3.784339000000	-0.816481000000
C	1.818367000000	5.767784000000	-0.334607000000
H	1.331964000000	4.130738000000	-1.617775000000
H	3.386880000000	3.090671000000	-1.648820000000
H	3.892626000000	4.644727000000	-1.068451000000
H	3.697735000000	3.344491000000	0.087289000000
H	0.879971000000	6.140450000000	0.076014000000
H	2.618143000000	6.050786000000	0.348904000000
H	1.996304000000	6.298686000000	-1.274331000000
C	0.631173000000	2.439467000000	2.565971000000

C 2.217185000000 4.342947000000 2.322440000000

H 2.421193000000 2.418268000000 1.419230000000

H 0.178376000000 1.549196000000 2.128206000000

H 1.177661000000 2.131818000000 3.461815000000

H -0.170047000000 3.108959000000 2.891813000000

H 3.110172000000 4.715026000000 1.820572000000

H 1.497986000000 5.162067000000 2.415258000000

H 2.511728000000 4.044624000000 3.331568000000

H 2.797786000000 1.379634000000 -1.463493000000

TSb8

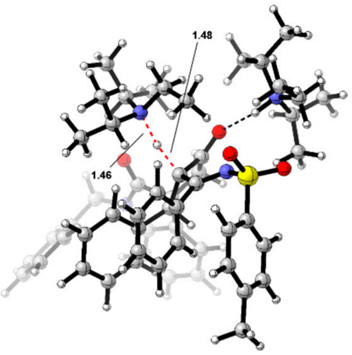

Frequency -1504.3359

Zero-point correction= 1.173118 (Hartree/Particle)

Thermal correction to Energy= 1.236098

Thermal correction to Enthalpy= 1.237042

Thermal correction to Gibbs Free Energy= 1.077155

Sum of electronic and zero-point Energies= -3054.216791

Sum of electronic and thermal Energies= -3054.153811

Sum of electronic and thermal Enthalpies= -3054.152866

Sum of electronic and thermal Free Energies= -3054.312754

E(RM062X) = -3056.04461739

N 0.354337000000 -2.137138000000 -2.328007000000

H 0.130770000000 -1.243974000000 -1.199117000000

C 0.187603000000 -0.328244000000 -0.035154000000

C 0.642398000000 -1.434089000000 0.832066000000

C 1.182176000000 0.771534000000 -0.244951000000

C -1.095123000000 0.215043000000 0.679912000000

N 2.375120000000 0.634392000000 0.231510000000

C 0.718125000000 2.017035000000 -0.948153000000

C 0.034335000000 3.013509000000 -0.245382000000

C -0.421942000000 4.146770000000 -0.904630000000

C -0.196192000000 4.297727000000 -2.271850000000

C 0.515115000000 3.326336000000 -2.967954000000

C 0.982524000000 2.192073000000 -2.306609000000

S 3.641190000000 1.723498000000 -0.033534000000

C 3.118950000000 3.380924000000 0.303143000000

C 3.065134000000 4.303902000000 -0.735334000000

C 2.639382000000 5.594172000000 -0.451134000000

C 2.265974000000 5.964040000000 0.845757000000

C 2.352983000000 5.015945000000 1.870339000000

C 2.788486000000 3.722330000000 1.610456000000

C 1.773784000000 7.360094000000 1.119650000000

O 4.026316000000 1.612669000000 -1.438126000000

O 4.596738000000 1.358544000000 1.009307000000

C -2.142325000000 1.008384000000 -0.076482000000

C -2.182464000000 1.034099000000 -1.466858000000

C -3.085390000000 1.844537000000 -2.148950000000

C -3.969991000000 2.649098000000 -1.436962000000

C -3.952756000000 2.621540000000 -0.043846000000

C -3.045534000000 1.811507000000 0.631821000000

H -0.141615000000 2.906289000000 0.820557000000

H -0.960194000000 4.908801000000 -0.350427000000

H -0.563000000000 5.178185000000 -2.789362000000

H 0.717896000000 3.451313000000 -4.026564000000

H 1.574067000000 1.457445000000 -2.842910000000

H 3.340110000000 4.000219000000 -1.739680000000

H 2.585200000000 6.327724000000 -1.250904000000

H	2.087530000000	5.299923000000	2.884333000000
H	2.879171000000	2.988829000000	2.405998000000
H	2.466052000000	8.104346000000	0.717740000000
H	0.802660000000	7.525566000000	0.642170000000
H	1.659066000000	7.541530000000	2.189720000000
H	-1.446991000000	0.464472000000	-2.015201000000
H	-3.077390000000	1.864872000000	-3.234471000000
H	-4.662177000000	3.299821000000	-1.962325000000
H	-4.641871000000	3.240380000000	0.521775000000
H	-3.023072000000	1.827002000000	1.718099000000
C	-1.676445000000	-1.004319000000	1.470606000000
C	-2.638767000000	-1.774193000000	0.564494000000
N	-3.882850000000	-1.744589000000	1.127205000000
C	-3.868558000000	-1.047791000000	2.347233000000
C	-4.927786000000	-0.784109000000	3.200774000000
C	-4.644128000000	-0.070162000000	4.368329000000
C	-3.351224000000	0.355277000000	4.664244000000
C	-2.567382000000	-0.617105000000	2.621022000000
C	-2.298983000000	0.081425000000	3.783954000000
O	-2.347906000000	-2.305601000000	-0.493307000000
C	-0.431145000000	-1.817267000000	1.835533000000
O	1.712718000000	-2.029629000000	0.767614000000
C	-5.043672000000	-2.391265000000	0.544218000000
C	-6.125591000000	-1.414784000000	0.139921000000
C	-7.455061000000	-1.668804000000	0.473487000000
C	-8.461858000000	-0.792050000000	0.077303000000
C	-8.140015000000	0.348284000000	-0.651997000000
C	-6.811075000000	0.607387000000	-0.982435000000
C	-5.805107000000	-0.268661000000	-0.589554000000
H	-3.160240000000	0.902748000000	5.580441000000
H	-5.451879000000	0.155754000000	5.056302000000
H	-5.937466000000	-1.100451000000	2.962573000000
H	-1.291029000000	0.423413000000	4.008337000000
H	-0.057883000000	-1.503049000000	2.819738000000
H	-7.705565000000	-2.559161000000	1.045505000000
H	-9.493883000000	-0.998837000000	0.341046000000
H	-8.921507000000	1.034368000000	-0.961769000000
H	-6.553195000000	1.495137000000	-1.550721000000
H	-4.767970000000	-0.061759000000	-0.849456000000
H	-5.440031000000	-3.119661000000	1.261240000000
H	-4.666323000000	-2.939554000000	-0.324245000000
H	-0.588540000000	-2.898128000000	1.879132000000
H	-0.731520000000	0.882203000000	1.478870000000
C	1.806368000000	-2.458019000000	-2.417835000000
C	-0.090216000000	-1.228335000000	-3.454640000000
C	-0.420071000000	-3.436938000000	-2.267556000000
C	2.765829000000	-1.290590000000	-2.273625000000
H	1.988008000000	-2.967340000000	-3.368383000000
H	2.018717000000	-3.172145000000	-1.618255000000
H	3.757988000000	-1.603492000000	-2.614650000000
H	2.496948000000	-0.414969000000	-2.868184000000
H	2.838400000000	-0.976147000000	-1.235285000000
C	-1.601521000000	-1.297286000000	-3.692527000000
C	0.646132000000	-1.384863000000	-4.790726000000
H	0.142868000000	-0.221363000000	-3.083444000000
H	-2.175972000000	-1.314199000000	-2.764742000000
H	-1.900791000000	-0.413130000000	-4.262629000000
H	-1.873813000000	-2.174597000000	-4.284750000000
H	1.716619000000	-1.184518000000	-4.721787000000
H	0.511079000000	-2.373779000000	-5.232830000000
H	0.224198000000	-0.655602000000	-5.487491000000
C	-0.113170000000	-4.229761000000	-0.999225000000
C	-0.243033000000	-4.355043000000	-3.481868000000
H	-1.462280000000	-3.147962000000	-2.205374000000
H	-0.061174000000	-3.587530000000	-0.123389000000
H	-0.924483000000	-4.944179000000	-0.839743000000

H	0.817134000000	-4.801149000000	-1.078537000000
H	-0.580002000000	-3.905302000000	-4.416035000000
H	0.787483000000	-4.697727000000	-3.609221000000
H	-0.856346000000	-5.245051000000	-3.314838000000
H	3.701694000000	-1.789280000000	0.706262000000
N	4.497057000000	-2.313297000000	1.112859000000
C	5.720881000000	-1.447268000000	0.958090000000
C	4.583211000000	-3.597222000000	0.282317000000
C	4.057995000000	-2.473047000000	2.566202000000
C	5.983659000000	-1.049431000000	-0.483624000000
H	6.559599000000	-1.992026000000	1.393139000000
H	5.535057000000	-0.543312000000	1.533693000000
H	6.801316000000	-0.325772000000	-0.487860000000
H	6.270862000000	-1.888636000000	-1.121339000000
H	5.120534000000	-0.542944000000	-0.921168000000
C	3.456876000000	-4.572927000000	0.608564000000
C	5.943786000000	-4.284405000000	0.362444000000
H	4.437020000000	-3.232177000000	-0.740559000000
H	2.480706000000	-4.085631000000	0.622348000000
H	3.450901000000	-5.344384000000	-0.166019000000
H	3.623200000000	-5.075147000000	1.565979000000
H	6.761732000000	-3.671174000000	-0.016334000000
H	6.187934000000	-4.603606000000	1.376641000000
H	5.894956000000	-5.181388000000	-0.259329000000
C	3.722621000000	-1.099551000000	3.142225000000
C	5.061673000000	-3.226262000000	3.426286000000
H	3.127175000000	-3.036074000000	2.490231000000
H	3.157233000000	-0.493622000000	2.427651000000
H	3.112500000000	-1.250673000000	4.036467000000
H	4.615061000000	-0.543979000000	3.439630000000
H	5.154292000000	-4.277843000000	3.151326000000
H	6.051192000000	-2.762031000000	3.406992000000
H	4.708786000000	-3.190387000000	4.459867000000

INTb13

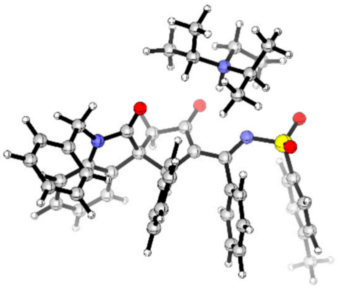

Zero-point correction= 0.895959 (Hartree/Particle)

Thermal correction to Energy= 0.946630

Thermal correction to Enthalpy= 0.947574

Thermal correction to Gibbs Free Energy= 0.809623

Sum of electronic and zero-point Energies= -2683.226425

Sum of electronic and thermal Energies= -2683.175754

Sum of electronic and thermal Enthalpies= -2683.174809

Sum of electronic and thermal Free Energies= -2683.312761

E(RM062X) = -2684.69980578

N	1.550424000000	3.805344000000	-0.087316000000
H	1.520590000000	2.891230000000	-0.600685000000
C	0.480307000000	0.114564000000	-0.927255000000
C	0.378655000000	1.131109000000	-1.923673000000
C	1.644664000000	-0.159630000000	-0.195373000000
C	-0.743926000000	-0.781411000000	-0.964260000000
N	2.630233000000	0.736374000000	-0.151517000000
C	1.702186000000	-1.510569000000	0.461402000000
C	1.799513000000	-2.645946000000	-0.346291000000
C	1.797096000000	-3.914293000000	0.223861000000
C	1.666858000000	-4.054628000000	1.603602000000
C	1.561059000000	-2.924322000000	2.410633000000
C	1.592612000000	-1.653114000000	1.845141000000
S	4.024199000000	0.481211000000	0.671791000000

C	4.688325000000	-1.079982000000	0.123535000000
C	4.857413000000	-2.114735000000	1.032132000000
C	5.298774000000	-3.350947000000	0.569356000000
C	5.568728000000	-3.556988000000	-0.784293000000
C	5.400302000000	-2.490480000000	-1.675773000000
C	4.965437000000	-1.249763000000	-1.230777000000
C	6.046160000000	-4.895510000000	-1.285667000000
O	3.794414000000	0.379707000000	2.119506000000
O	4.931594000000	1.534779000000	0.212572000000
C	-1.300884000000	-1.226363000000	0.375684000000
C	-1.351571000000	-0.359348000000	1.474256000000
C	-1.811020000000	-0.804943000000	2.708373000000
C	-2.229263000000	-2.125526000000	2.867019000000
C	-2.205200000000	-2.989080000000	1.775924000000
C	-1.747088000000	-2.538415000000	0.538585000000
H	1.896645000000	-2.523667000000	-1.422191000000
H	1.892718000000	-4.791800000000	-0.408364000000
H	1.647796000000	-5.044246000000	2.049701000000
H	1.453625000000	-3.030695000000	3.485484000000
H	1.531948000000	-0.768215000000	2.469552000000
H	4.609042000000	-1.948979000000	2.075303000000
H	5.418051000000	-4.172911000000	1.269830000000
H	5.611465000000	-2.640665000000	-2.731473000000
H	4.825246000000	-0.421586000000	-1.919134000000
H	5.905904000000	-5.673568000000	-0.532233000000
H	5.506791000000	-5.192385000000	-2.189449000000
H	7.111051000000	-4.860387000000	-1.537364000000
H	-1.013706000000	0.665653000000	1.354119000000
H	-1.836383000000	-0.121846000000	3.551726000000
H	-2.573873000000	-2.476760000000	3.834886000000
H	-2.530645000000	-4.018915000000	1.887035000000
H	-1.707534000000	-3.219300000000	-0.308217000000
C	-1.766516000000	0.042336000000	-1.832868000000
C	-2.574767000000	1.014588000000	-0.963864000000
N	-3.886097000000	0.602885000000	-0.970265000000
C	-4.070449000000	-0.481233000000	-1.843350000000
C	-5.247978000000	-1.144153000000	-2.151607000000
C	-5.169757000000	-2.187292000000	-3.078767000000
C	-3.958259000000	-2.547721000000	-3.661716000000
C	-2.843467000000	-0.828004000000	-2.418535000000
C	-2.781219000000	-1.869010000000	-3.326615000000
O	-2.157633000000	2.006080000000	-0.389353000000
C	-0.866529000000	0.853818000000	-2.769003000000
O	1.141224000000	2.077349000000	-2.175343000000
C	-4.941358000000	1.286061000000	-0.259091000000
C	-5.665218000000	0.404936000000	0.739247000000
C	-6.977139000000	0.717063000000	1.098306000000
C	-7.652374000000	-0.049071000000	2.043111000000
C	-7.021741000000	-1.142961000000	2.631121000000
C	-5.717025000000	-1.463731000000	2.266668000000
C	-5.039344000000	-0.695540000000	1.324136000000
H	-3.925354000000	-3.362273000000	-4.377167000000
H	-6.073117000000	-2.727583000000	-3.342354000000
H	-6.187084000000	-0.873577000000	-1.680491000000
H	-1.833124000000	-2.152984000000	-3.776481000000
H	-0.573652000000	0.251373000000	-3.638205000000
H	-7.472685000000	1.566958000000	0.634568000000
H	-8.672395000000	0.204036000000	2.314727000000
H	-7.548164000000	-1.744656000000	3.365145000000
H	-5.213509000000	-2.316050000000	2.712720000000
H	-4.023965000000	-0.958014000000	1.040034000000
H	-5.660442000000	1.696659000000	-0.979893000000
H	-4.456398000000	2.127933000000	0.245564000000
H	-1.324676000000	1.777220000000	-3.132164000000
H	-0.548538000000	-1.698424000000	-1.541287000000
C	2.360670000000	4.657915000000	-1.017597000000

C	2.274132000000	3.494049000000	1.215571000000
C	0.093546000000	4.232652000000	0.042432000000
C	3.718171000000	4.033556000000	-1.303186000000
H	2.429126000000	5.663588000000	-0.594013000000
H	1.790767000000	4.706310000000	-1.944561000000
H	4.184916000000	4.579341000000	-2.126466000000
H	4.396186000000	4.051237000000	-0.448230000000
H	3.589667000000	2.987442000000	-1.597398000000
C	1.412024000000	2.614097000000	2.110843000000
C	2.848775000000	4.707647000000	1.939150000000
H	3.120817000000	2.897191000000	0.878740000000
H	0.954279000000	1.806690000000	1.532201000000
H	2.064840000000	2.147561000000	2.852241000000
H	0.628717000000	3.177599000000	2.626352000000
H	3.504051000000	5.301467000000	1.297797000000
H	2.096017000000	5.363672000000	2.375860000000
H	3.466648000000	4.319983000000	2.753276000000
C	-0.537343000000	4.525346000000	-1.317579000000
C	-0.104538000000	5.424544000000	0.971694000000
H	-0.406962000000	3.350366000000	0.448446000000
H	-0.242854000000	3.791747000000	-2.068878000000
H	-1.620184000000	4.465126000000	-1.188933000000
H	-0.283831000000	5.531240000000	-1.667773000000
H	0.083140000000	5.189510000000	2.018980000000
H	0.521470000000	6.274218000000	0.680656000000
H	-1.146925000000	5.741806000000	0.886609000000

TSb9

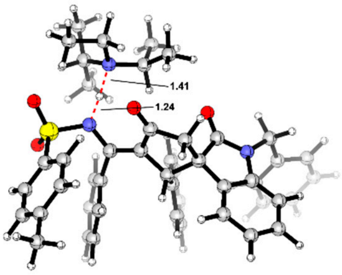

Frequency -912.2379

Zero-point correction= 0.891442 (Hartree/Particle)

Thermal correction to Energy= 0.941383

Thermal correction to Enthalpy= 0.942327

Thermal correction to Gibbs Free Energy= 0.807400

Sum of electronic and zero-point Energies= -2683.212146

Sum of electronic and thermal Energies= -2683.162205

Sum of electronic and thermal Enthalpies= -2683.161261

Sum of electronic and thermal Free Energies= -2683.296188

E(RM062X) = -2684.67820328

N	2.638289000000	0.641707000000	0.188451000000
H	2.533059000000	1.774720000000	-0.302964000000
N	2.398310000000	3.171640000000	-0.454184000000
C	1.557165000000	-0.199313000000	0.285638000000
C	0.543912000000	-0.149449000000	-0.634943000000
C	0.703089000000	0.373982000000	-1.985732000000
C	-0.779482000000	-0.878914000000	-0.512877000000
C	1.445302000000	-1.208497000000	1.386072000000
C	1.399116000000	-2.564589000000	1.056201000000
C	1.164533000000	-3.517995000000	2.041250000000
C	0.950537000000	-3.115706000000	3.356772000000
C	1.006811000000	-1.763948000000	3.690911000000
C	1.272742000000	-0.813248000000	2.712823000000
S	4.113411000000	0.125654000000	0.761464000000
C	4.283965000000	-1.454129000000	-0.042796000000
C	4.734971000000	-2.541729000000	0.692959000000

C	4.833344000000	-3.779203000000	0.065395000000	C	-3.949856000000	-0.682535000000	-1.900118000000
C	4.485074000000	-3.933962000000	-1.278696000000	C	-5.150710000000	-1.331916000000	-2.140400000000
C	4.028989000000	-2.818571000000	-1.990724000000	C	-5.084434000000	-2.635247000000	-2.640510000000
C	3.925751000000	-1.572704000000	-1.383693000000	C	-3.862967000000	-3.257772000000	-2.882062000000
C	4.614239000000	-5.273708000000	-1.955529000000	C	-2.712749000000	-1.291693000000	-2.136546000000
O	4.115490000000	-0.101061000000	2.202447000000	C	-2.663638000000	-2.584439000000	-2.624567000000
O	5.095810000000	1.056714000000	0.216756000000	O	-1.938222000000	1.988814000000	-1.016017000000
C	-1.484412000000	-0.783060000000	0.826406000000	C	-0.551618000000	0.009481000000	-2.784086000000
C	-1.531192000000	0.412676000000	1.554974000000	O	1.681399000000	0.913270000000	-2.482508000000
C	-2.153600000000	0.458983000000	2.797056000000	C	-4.787872000000	1.559210000000	-1.075176000000
C	-2.730892000000	-0.688669000000	3.338508000000	C	-5.687734000000	1.070742000000	0.042560000000
C	-2.693901000000	-1.880150000000	2.620596000000	C	-7.008780000000	1.515504000000	0.106215000000
C	-2.077758000000	-1.922269000000	1.371177000000	C	-7.843237000000	1.104290000000	1.141545000000
H	1.570805000000	-2.864369000000	0.024185000000	C	-7.364186000000	0.234660000000	2.117952000000
H	1.144266000000	-4.571460000000	1.780457000000	C	-6.048970000000	-0.216998000000	2.052688000000
H	0.749096000000	-3.855638000000	4.125082000000	C	-5.212225000000	0.198726000000	1.021323000000
H	0.847546000000	-1.449762000000	4.717146000000	H	-3.840576000000	-4.270706000000	-3.268855000000
H	1.329341000000	0.240689000000	2.967082000000	H	-6.007205000000	-3.170500000000	-2.839142000000
H	4.964386000000	-2.415277000000	1.745977000000	H	-6.101976000000	-0.852773000000	-1.935152000000
H	5.172026000000	-4.642324000000	0.631805000000	H	-1.707821000000	-3.069314000000	-2.807910000000
H	3.744038000000	-2.931323000000	-3.033278000000	H	-0.320366000000	-0.864243000000	-3.405828000000
H	3.537557000000	-0.709370000000	-1.922240000000	H	-7.386909000000	2.188686000000	-0.659751000000
H	4.500852000000	-6.091357000000	-1.239980000000	H	-8.868777000000	1.457638000000	1.180984000000
H	3.864053000000	-5.395752000000	-2.740452000000	H	-8.015234000000	-0.092805000000	2.922163000000
H	5.599441000000	-5.376279000000	-2.422028000000	H	-5.662269000000	-0.899589000000	2.803148000000
H	-1.071017000000	1.306264000000	1.143461000000	H	-4.189467000000	-0.164370000000	0.974020000000
H	-2.181720000000	1.393464000000	3.348561000000	H	-5.388812000000	1.780845000000	-1.966340000000
H	-3.205405000000	-0.651733000000	4.314374000000	H	-4.267964000000	2.478299000000	-0.785489000000
H	-3.134773000000	-2.782190000000	3.033718000000	H	-0.839999000000	0.828037000000	-3.448120000000
H	-2.035389000000	-2.857167000000	0.817811000000	H	-0.637155000000	-1.949444000000	-0.718781000000
C	-1.615836000000	-0.343574000000	-1.734918000000	C	3.128733000000	3.664813000000	-1.651840000000
C	-2.402678000000	0.911583000000	-1.339853000000	C	3.113154000000	3.527539000000	0.823497000000
N	-3.744166000000	0.619428000000	-1.419320000000	C	0.918809000000	3.512963000000	-0.516385000000

C	4.590437000000	3.254733000000	-1.740231000000
H	3.032315000000	4.757958000000	-1.712463000000
H	2.624670000000	3.238633000000	-2.517010000000
H	4.991401000000	3.649749000000	-2.677919000000
H	5.208457000000	3.635279000000	-0.923957000000
H	4.688815000000	2.169222000000	-1.748860000000
C	2.373726000000	3.002918000000	2.075642000000
C	3.535241000000	4.991466000000	0.985842000000
H	4.042805000000	2.960010000000	0.744170000000
H	1.459158000000	2.461365000000	1.820569000000
H	3.011114000000	2.310607000000	2.632163000000
H	2.099773000000	3.823734000000	2.744777000000
H	4.100009000000	5.356210000000	0.124522000000
H	2.698804000000	5.667516000000	1.165019000000
H	4.198017000000	5.047619000000	1.854817000000
C	0.383354000000	3.707061000000	-1.940970000000
C	0.490628000000	4.752216000000	0.275811000000
H	0.400098000000	2.642488000000	-0.098379000000
H	0.621232000000	2.881170000000	-2.608045000000
H	-0.702231000000	3.777495000000	-1.869395000000
H	0.760685000000	4.636772000000	-2.377952000000
H	0.729875000000	4.704036000000	1.337058000000
H	0.930570000000	5.661878000000	-0.145570000000
H	-0.595977000000	4.838229000000	0.186344000000

----

b

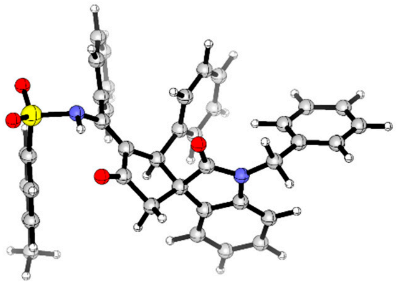

Zero-point correction= 0.627366 (Hartree/Particle)

Thermal correction to Energy= 0.665187

Thermal correction to Enthalpy= 0.666131

Thermal correction to Gibbs Free Energy= 0.553124

Sum of electronic and zero-point Energies= -2312.603048

Sum of electronic and thermal Energies= -2312.565227

Sum of electronic and thermal Enthalpies= -2312.564283

Sum of electronic and thermal Free Energies= -2312.677290

E(RM062X) = -2313.72282199

N	2.924871000000	0.871462000000	-1.819380000000
H	2.761209000000	0.158496000000	-2.536954000000
C	1.993688000000	0.793681000000	-0.768676000000
C	1.087117000000	-0.220286000000	-0.747400000000
C	1.174269000000	-1.386380000000	-1.654932000000
C	-0.088727000000	-0.424922000000	0.192229000000
C	2.041045000000	1.894080000000	0.218096000000
C	1.844518000000	1.647291000000	1.579651000000
C	1.815622000000	2.700795000000	2.484830000000
C	1.983567000000	4.008310000000	2.034735000000
C	2.203425000000	4.257970000000	0.681878000000
C	2.241664000000	3.206044000000	-0.223988000000
S	4.582664000000	0.826065000000	-1.445589000000
C	4.676831000000	-0.489610000000	-0.252732000000
C	4.777562000000	-0.175933000000	1.098912000000
C	4.756813000000	-1.211525000000	2.026283000000
C	4.629191000000	-2.542736000000	1.617446000000

C	4.529880000000	-2.822975000000	0.251152000000
C	4.550773000000	-1.803149000000	-0.693266000000
C	4.583992000000	-3.650171000000	2.637839000000
O	4.956988000000	2.059350000000	-0.783293000000
O	5.204966000000	0.402104000000	-2.685617000000
C	-0.914261000000	0.785264000000	0.578049000000
C	-1.077365000000	1.869692000000	-0.289153000000
C	-1.809079000000	2.983557000000	0.109607000000
C	-2.398669000000	3.023339000000	1.371686000000
C	-2.271578000000	1.932216000000	2.226947000000
C	-1.531355000000	0.820571000000	1.830360000000
H	1.729900000000	0.624739000000	1.927273000000
H	1.658706000000	2.502190000000	3.539936000000
H	1.952267000000	4.832191000000	2.740588000000
H	2.349192000000	5.274524000000	0.332340000000
H	2.425110000000	3.385012000000	-1.278022000000
H	4.872831000000	0.861991000000	1.401929000000
H	4.844487000000	-0.984535000000	3.085411000000
H	4.440073000000	-3.854259000000	-0.077987000000
H	4.462538000000	-2.015327000000	-1.754590000000
H	3.619568000000	-3.661199000000	3.156245000000
H	4.724019000000	-4.626934000000	2.170559000000
H	5.360319000000	-3.518220000000	3.395810000000
H	-0.617410000000	1.838676000000	-1.272547000000
H	-1.914425000000	3.824958000000	-0.567400000000
H	-2.958453000000	3.899787000000	1.683494000000
H	-2.738186000000	1.947845000000	3.207011000000
H	-1.419746000000	-0.026536000000	2.503583000000
C	-0.929770000000	-1.551448000000	-0.510635000000
C	-1.861843000000	-0.928184000000	-1.558441000000
N	-3.159217000000	-1.202678000000	-1.187217000000
C	-3.196411000000	-1.986959000000	-0.025298000000
C	-4.305601000000	-2.476564000000	0.647390000000
C	-4.073688000000	-3.253312000000	1.785838000000
C	-2.782500000000	-3.524083000000	2.230241000000
C	-1.891221000000	-2.248814000000	0.407995000000
C	-1.677678000000	-3.016018000000	1.538229000000
O	-1.516498000000	-0.314714000000	-2.547255000000
C	0.126611000000	-2.399386000000	-1.224909000000
O	1.965025000000	-1.567658000000	-2.565503000000
C	-4.313861000000	-0.774496000000	-1.946252000000
C	-5.265083000000	0.087021000000	-1.141457000000
C	-6.634471000000	0.036813000000	-1.401112000000
C	-7.518161000000	0.840100000000	-0.685663000000
C	-7.037431000000	1.693282000000	0.304002000000
C	-5.671083000000	1.739955000000	0.570382000000
C	-4.787249000000	0.941722000000	-0.148184000000
H	-2.632265000000	-4.130436000000	3.116818000000
H	-4.922409000000	-3.649322000000	2.333973000000
H	-5.312180000000	-2.250727000000	0.312294000000
H	-0.667203000000	-3.223912000000	1.882735000000
H	0.597304000000	-3.092656000000	-0.514808000000
H	-7.012143000000	-0.636813000000	-2.166740000000
H	-8.581934000000	0.792983000000	-0.895931000000
H	-7.724921000000	2.314748000000	0.868820000000
H	-5.283208000000	2.397114000000	1.342741000000
H	-3.723111000000	0.974396000000	0.068921000000
H	-4.843948000000	-1.654717000000	-2.331853000000
H	-3.911430000000	-0.223182000000	-2.802551000000
H	-0.246816000000	-2.971289000000	-2.075792000000
H	0.269315000000	-0.889266000000	1.123568000000

[sr]INTb5'

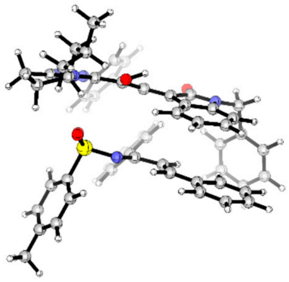

Zero-point correction= 0.948227 (Hartree/Particle)

Thermal correction to Energy= 1.004310

Thermal correction to Enthalpy= 1.005254

Thermal correction to Gibbs Free Energy= 0.855749

Sum of electronic and zero-point Energies= -3059.238033

Sum of electronic and thermal Energies= -3059.181950

Sum of electronic and thermal Enthalpies= -3059.181006

Sum of electronic and thermal Free Energies= -3059.330511

E(RM062X) = -3060.85071828

C	1.230325000000	-1.616186000000	1.278055000000
C	0.432718000000	1.283049000000	-0.610660000000
C	-0.061948000000	-1.244614000000	1.651580000000
C	-1.262057000000	-1.718536000000	1.173427000000
O	-1.314727000000	-2.784602000000	0.282431000000
H	-0.539422000000	-3.333918000000	0.461770000000
C	1.831792000000	-2.322982000000	0.159181000000
C	3.231590000000	-2.356035000000	0.403890000000
C	4.134007000000	-2.916605000000	-0.484590000000
C	3.636068000000	-3.469703000000	-1.666772000000
C	1.370606000000	-2.826830000000	-1.062072000000
C	2.273324000000	-3.418421000000	-1.949345000000
N	3.493053000000	-1.732289000000	1.619665000000
C	2.317789000000	-1.229757000000	2.170949000000
O	2.284725000000	-0.568623000000	3.215176000000
C	1.790244000000	0.879850000000	-0.992294000000
C	2.020552000000	0.158056000000	-2.106515000000
N	-0.543522000000	0.854553000000	-1.352336000000
S	-2.159628000000	1.087395000000	-1.078007000000
O	-2.806578000000	-0.125084000000	-1.575310000000
O	-2.511403000000	1.545070000000	0.266517000000
C	-2.546633000000	2.392645000000	-2.222722000000
C	-2.305794000000	2.195874000000	-3.581714000000
C	-2.624697000000	3.210627000000	-4.471221000000
C	-3.189866000000	4.412343000000	-4.024282000000
C	-3.430856000000	4.574865000000	-2.659668000000
C	-3.112008000000	3.569195000000	-1.750113000000
C	-3.532058000000	5.497749000000	-5.010948000000
C	4.793456000000	-1.413359000000	2.165135000000
C	5.375423000000	-0.150818000000	1.561893000000
C	6.581382000000	-0.155334000000	0.866533000000
C	7.079354000000	1.021981000000	0.305253000000
C	6.362058000000	2.208098000000	0.419666000000
C	5.155135000000	2.220801000000	1.121030000000
C	4.671181000000	1.052007000000	1.697521000000
C	3.312612000000	-0.236517000000	-2.660602000000
C	4.534600000000	0.009357000000	-2.017881000000
C	5.725573000000	-0.398515000000	-2.604399000000
C	5.721707000000	-1.053421000000	-3.835183000000
C	4.514794000000	-1.302951000000	-4.483334000000
C	3.323190000000	-0.897979000000	-3.896460000000
C	0.366417000000	2.254661000000	0.522788000000
C	-0.224248000000	3.507254000000	0.341392000000
C	-0.132752000000	4.471601000000	1.339689000000
C	0.541880000000	4.190725000000	2.525705000000
C	1.119600000000	2.937642000000	2.714878000000
C	1.042381000000	1.977343000000	1.713401000000
H	4.325128000000	-3.900470000000	-2.385967000000
H	5.201173000000	-2.896326000000	-0.284776000000
H	0.324519000000	-2.735811000000	-1.335081000000

H	1.906444000000	-3.813782000000	-2.891816000000
H	1.146743000000	-0.143715000000	-2.681707000000
H	-1.875539000000	1.259824000000	-3.923195000000
H	-2.436680000000	3.073511000000	-5.532699000000
H	-3.873123000000	5.500318000000	-2.302015000000
H	-3.291446000000	3.683129000000	-0.685446000000
H	-4.291998000000	5.153764000000	-5.719078000000
H	-3.916287000000	6.386918000000	-4.507405000000
H	-2.652067000000	5.788320000000	-5.592010000000
H	7.135661000000	-1.085134000000	0.758771000000
H	8.024054000000	1.007578000000	-0.230538000000
H	6.742440000000	3.120289000000	-0.028649000000
H	4.591253000000	3.143428000000	1.224193000000
H	3.746638000000	1.054662000000	2.269008000000
H	5.467336000000	-2.262990000000	2.014690000000
H	4.642066000000	-1.275369000000	3.239916000000
H	4.558234000000	0.503916000000	-1.050117000000
H	6.661868000000	-0.206068000000	-2.091407000000
H	6.657632000000	-1.369664000000	-4.285536000000
H	4.502542000000	-1.815369000000	-5.440054000000
H	2.376390000000	-1.101303000000	-4.390012000000
H	-0.722864000000	3.738580000000	-0.594836000000
H	-0.578889000000	5.448960000000	1.184162000000
H	0.614060000000	4.947117000000	3.301762000000
H	1.626992000000	2.689527000000	3.642089000000
H	1.498629000000	1.008287000000	1.874247000000
C	-2.548616000000	-1.220327000000	1.493092000000
N	-2.986153000000	-0.284604000000	2.384291000000
N	-4.329323000000	-0.004959000000	2.232341000000
C	-4.702349000000	-0.808152000000	1.292109000000
N	-3.678287000000	-1.580978000000	0.832156000000
C	-5.959863000000	-1.115928000000	0.549153000000
C	-5.389383000000	-1.810239000000	-0.713006000000

C	-4.045424000000	-2.460713000000	-0.289171000000
C	-4.140533000000	-3.922579000000	0.163608000000
C	-3.814907000000	-4.915061000000	-0.957004000000
C	-2.264770000000	0.487220000000	3.338750000000
C	-1.498491000000	-0.143630000000	4.314623000000
C	-0.846000000000	0.630692000000	5.267840000000
C	-1.006184000000	2.013915000000	5.271071000000
C	-1.793566000000	2.628636000000	4.300026000000
C	-2.413181000000	1.871335000000	3.313849000000
C	-3.882397000000	-6.346595000000	-0.427910000000
C	-4.723850000000	-4.749987000000	-2.175517000000
H	-6.589897000000	-1.787813000000	1.141070000000
H	-6.534886000000	-0.218054000000	0.322716000000
H	-5.175067000000	-1.053879000000	-1.471257000000
H	-6.080062000000	-2.546903000000	-1.125738000000
H	-3.290734000000	-2.343380000000	-1.066203000000
H	-5.144148000000	-4.125813000000	0.567515000000
H	-3.424371000000	-4.077848000000	0.975954000000
H	-2.781548000000	-4.704953000000	-1.266986000000
H	-1.405104000000	-1.224950000000	4.312884000000
H	-0.224011000000	0.148033000000	6.013437000000
H	-0.508970000000	2.613553000000	6.027013000000
H	-1.905274000000	3.707857000000	4.291673000000
H	-3.001385000000	2.321368000000	2.523139000000
H	-3.592092000000	-7.066385000000	-1.198839000000
H	-3.219970000000	-6.484167000000	0.431844000000
H	-4.902545000000	-6.590706000000	-0.109670000000
H	-4.475616000000	-5.487153000000	-2.944893000000
H	-4.630921000000	-3.757587000000	-2.627484000000
H	-5.774207000000	-4.901631000000	-1.897113000000
H	2.597850000000	1.231490000000	-0.355916000000
H	-0.084964000000	-0.528256000000	2.467170000000

----

^{sr}TSb3'

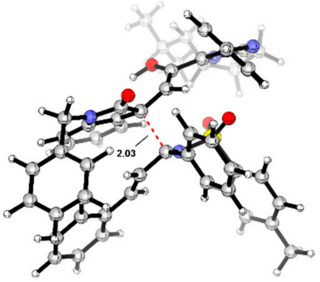

Frequency -283.7768

Zero-point correction= 0.948452 (Hartree/Particle)

Thermal correction to Energy= 1.003439

Thermal correction to Enthalpy= 1.004384

Thermal correction to Gibbs Free Energy= 0.856567

Sum of electronic and zero-point Energies= -3059.208057

Sum of electronic and thermal Energies= -3059.153069

Sum of electronic and thermal Enthalpies= -3059.152125

Sum of electronic and thermal Free Energies= -3059.299942

E(RM062X) = -3060.82104413

C	0.616715000000	-1.122898000000	0.925804000000
C	0.853681000000	0.696502000000	0.053209000000
C	-0.765653000000	-0.988945000000	1.374150000000
C	-1.836672000000	-1.666474000000	0.926765000000
O	-1.879494000000	-2.779986000000	0.140243000000
H	-0.976102000000	-3.005672000000	-0.134560000000
C	1.036477000000	-1.988340000000	-0.201551000000
C	2.312937000000	-2.504837000000	0.104343000000
C	3.081606000000	-3.186561000000	-0.822134000000
C	2.542797000000	-3.366441000000	-2.101350000000
C	0.533031000000	-2.147782000000	-1.489296000000
C	1.290774000000	-2.859775000000	-2.429488000000
N	2.632810000000	-2.165359000000	1.417291000000
C	1.630127000000	-1.405150000000	1.997034000000
O	1.632241000000	-1.090008000000	3.179916000000
C	2.248337000000	0.528974000000	-0.459047000000
C	2.530246000000	0.423004000000	-1.762804000000
N	-0.077472000000	0.700208000000	-0.950603000000
S	-1.574311000000	1.231322000000	-0.837654000000
O	-2.436472000000	0.323202000000	-1.625072000000
O	-2.079879000000	1.556908000000	0.512933000000
C	-1.573778000000	2.772853000000	-1.739500000000
C	-1.304675000000	2.757452000000	-3.107253000000
C	-1.267522000000	3.956781000000	-3.802908000000
C	-1.494943000000	5.176180000000	-3.151205000000
C	-1.763278000000	5.162897000000	-1.782968000000
C	-1.804863000000	3.965993000000	-1.069668000000
C	-1.445024000000	6.468429000000	-3.925017000000
C	3.824880000000	-2.552733000000	2.141761000000
C	4.972164000000	-1.595280000000	1.904517000000
C	6.172501000000	-2.022953000000	1.343235000000
C	7.206743000000	-1.115841000000	1.112842000000
C	7.036880000000	0.228357000000	1.427883000000
C	5.837367000000	0.661748000000	1.993803000000
C	4.813150000000	-0.245606000000	2.239276000000
C	3.849881000000	0.185214000000	-2.360318000000
C	4.979259000000	-0.169835000000	-1.607525000000
C	6.198978000000	-0.397772000000	-2.231484000000
C	6.319260000000	-0.285479000000	-3.616125000000
C	5.203719000000	0.054243000000	-4.375844000000
C	3.982850000000	0.283693000000	-3.751030000000
C	0.816141000000	1.823802000000	1.086767000000
C	0.945039000000	3.101156000000	0.519005000000
C	0.964269000000	4.240666000000	1.310898000000
C	0.871304000000	4.127085000000	2.697261000000
C	0.764119000000	2.865299000000	3.266700000000
C	0.733230000000	1.716360000000	2.472686000000

H	3.124943000000	-3.891756000000	-2.851380000000
H	4.080117000000	-3.535677000000	-0.579398000000
H	-0.404303000000	-1.673723000000	-1.769031000000
H	0.902842000000	-2.993115000000	-3.433899000000
H	1.693101000000	0.506953000000	-2.453126000000
H	-1.130956000000	1.809521000000	-3.606992000000
H	-1.058133000000	3.955108000000	-4.869690000000
H	-1.941727000000	6.101960000000	-1.265883000000
H	-2.001572000000	3.943498000000	-0.002970000000
H	-2.177564000000	6.467802000000	-4.737994000000
H	-1.655603000000	7.324910000000	-3.281065000000
H	-0.458909000000	6.617352000000	-4.375304000000
H	6.301278000000	-3.071032000000	1.083042000000
H	8.140421000000	-1.460673000000	0.678430000000
H	7.834793000000	0.938289000000	1.235253000000
H	5.699843000000	1.708981000000	2.243097000000
H	3.877717000000	0.083928000000	2.687989000000
H	4.100194000000	-3.571978000000	1.851629000000
H	3.546098000000	-2.554622000000	3.199758000000
H	4.900982000000	-0.284234000000	-0.529720000000
H	7.059161000000	-0.670919000000	-1.628192000000
H	7.274864000000	-0.466999000000	-4.098102000000
H	5.283658000000	0.139438000000	-5.455316000000
H	3.110155000000	0.546780000000	-4.342927000000
H	1.033195000000	3.187494000000	-0.560755000000
H	1.056948000000	5.216389000000	0.843079000000
H	0.892203000000	5.013902000000	3.323304000000
H	0.702339000000	2.756497000000	4.345742000000
H	0.664442000000	0.752546000000	2.961658000000
C	-3.174907000000	-1.116390000000	1.100914000000
N	-3.675683000000	-0.305599000000	2.045733000000
N	-4.901278000000	0.193804000000	1.701165000000
C	-5.144684000000	-0.365228000000	0.552829000000
N	-4.134201000000	-1.193639000000	0.172299000000
C	-6.203860000000	-0.325684000000	-0.498544000000
C	-5.416134000000	-0.869191000000	-1.720670000000
C	-4.317964000000	-1.808098000000	-1.156123000000
C	-4.706022000000	-3.284584000000	-1.023450000000
C	-4.241076000000	-4.124738000000	-2.218014000000
C	-3.034012000000	0.153553000000	3.238893000000
C	-2.575976000000	-0.775289000000	4.166729000000
C	-1.912157000000	-0.314086000000	5.297143000000
C	-1.739841000000	1.054805000000	5.494440000000
C	-2.217630000000	1.967307000000	4.556685000000
C	-2.861667000000	1.521510000000	3.407657000000
C	-4.615934000000	-5.589945000000	-2.003755000000
C	-4.796004000000	-3.615575000000	-3.548853000000
H	-7.038731000000	-0.976036000000	-0.218218000000
H	-6.589609000000	0.682052000000	-0.651879000000
H	-4.916479000000	-0.037967000000	-2.223564000000
H	-6.062354000000	-1.388772000000	-2.429029000000
H	-3.379344000000	-1.688060000000	-1.697840000000
H	-5.795495000000	-3.376921000000	-0.899614000000
H	-4.238017000000	-3.689010000000	-0.120783000000
H	-3.145094000000	-4.045694000000	-2.248219000000
H	-2.720357000000	-1.836097000000	3.990095000000
H	-1.529721000000	-1.023608000000	6.022200000000
H	-1.220904000000	1.409658000000	6.379076000000
H	-2.060295000000	3.030849000000	4.699906000000
H	-3.189517000000	2.195886000000	2.625684000000
H	-4.241338000000	-6.214851000000	-2.819394000000
H	-4.204357000000	-5.972937000000	-1.065401000000
H	-5.705265000000	-5.706156000000	-1.967058000000
H	-4.462541000000	-4.251587000000	-4.373795000000
H	-4.470025000000	-2.594140000000	-3.768736000000
H	-5.892927000000	-3.630741000000	-3.540244000000

H 3.025873000000 0.486129000000 0.302875000000

H -0.974470000000 -0.145020000000 2.020519000000

[sr]INTb6'

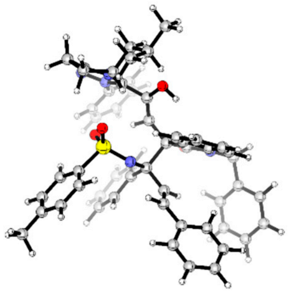

Zero-point correction= 0.948327 (Hartree/Particle)

Thermal correction to Energy= 1.003875

Thermal correction to Enthalpy= 1.004819

Thermal correction to Gibbs Free Energy= 0.853839

Sum of electronic and zero-point Energies= -3059.213094

Sum of electronic and thermal Energies= -3059.157546

Sum of electronic and thermal Enthalpies= -3059.156601

Sum of electronic and thermal Free Energies= -3059.307582

E(RM062X) = -3060.82596765

O -1.813835000000 2.909144000000 0.135528000000

H -0.909845000000 3.018154000000 0.475023000000

C -1.846485000000 1.832843000000 -0.694237000000

C -0.854430000000 1.079050000000 -1.181983000000

C 0.568655000000 1.015513000000 -0.733714000000

C 0.956487000000 1.845025000000 0.476125000000

C 2.121672000000 2.563187000000 0.175334000000

C 2.810477000000 3.294871000000 1.129439000000

C 2.284649000000 3.317850000000 2.423980000000

C 0.455994000000 1.860484000000 1.772530000000

C 1.121481000000 2.623652000000 2.740718000000

N 2.438623000000 2.390960000000 -1.173398000000

C 1.547981000000 1.547873000000 -1.802936000000

O 1.569752000000 1.328448000000 -2.998921000000

C 2.580215000000 -0.907920000000 1.476357000000

C 2.248057000000 -0.603611000000 0.221470000000

C 0.814240000000 -0.563103000000 -0.288988000000

N -0.073887000000 -0.819036000000 0.804781000000

S -1.546005000000 -1.342904000000 0.725479000000

O -2.360836000000 -0.617177000000 1.734079000000

O -2.189853000000 -1.425224000000 -0.611366000000

C -1.496515000000 -3.032367000000 1.317000000000

C -1.052528000000 -3.279628000000 2.615490000000

C -0.942407000000 -4.588959000000 3.059540000000

C -1.267166000000 -5.664346000000 2.221617000000

C -1.711351000000 -5.391531000000 0.929289000000

C -1.827758000000 -4.079750000000 0.469921000000

C -1.122230000000 -7.081044000000 2.715566000000

C 3.555214000000 2.999676000000 -1.870351000000

C 4.830806000000 2.210505000000 -1.678897000000

C 5.916511000000 2.741973000000 -0.987710000000

C 7.068349000000 1.981032000000 -0.792837000000

C 7.132883000000 0.679907000000 -1.280870000000

C 6.048743000000 0.142723000000 -1.974530000000

C 4.905509000000 0.905895000000 -2.178976000000

C 3.947513000000 -0.909066000000 2.022782000000

C 4.965769000000 -0.098797000000 1.501923000000

C 6.241046000000 -0.126910000000 2.055184000000

C 6.524686000000 -0.956309000000 3.138138000000

C 5.517762000000 -1.755610000000 3.672615000000

C 4.241341000000 -1.724558000000 3.122938000000

C 0.722947000000 -1.617989000000 -1.417371000000

C 0.933177000000 -2.937414000000 -0.988757000000

C 0.872895000000 -4.004123000000 -1.873225000000

C 0.613031000000 -3.776963000000 -3.224274000000

C	0.413421000000	-2.475040000000	-3.661348000000
C	0.460233000000	-1.400881000000	-2.769277000000
H	2.801719000000	3.880646000000	3.194372000000
H	3.733073000000	3.811350000000	0.887434000000
H	-0.410507000000	1.253818000000	2.020582000000
H	0.739595000000	2.647281000000	3.755663000000
H	1.771424000000	-1.184664000000	2.150228000000
H	-0.797730000000	-2.443143000000	3.259385000000
H	-0.597533000000	-4.788751000000	4.071267000000
H	-1.966346000000	-6.216111000000	0.268502000000
H	-2.155783000000	-3.856758000000	-0.539786000000
H	-1.663433000000	-7.229019000000	3.654845000000
H	-1.507311000000	-7.796825000000	1.986006000000
H	-0.071702000000	-7.324400000000	2.903902000000
H	5.863524000000	3.757349000000	-0.601320000000
H	7.910294000000	2.403770000000	-0.252952000000
H	8.023068000000	0.081269000000	-1.115688000000
H	6.095112000000	-0.872743000000	-2.353887000000
H	4.056055000000	0.497424000000	-2.723082000000
H	3.672692000000	4.028563000000	-1.514409000000
H	3.272987000000	3.025389000000	-2.926552000000
H	4.749681000000	0.576073000000	0.676089000000
H	7.013371000000	0.513830000000	1.641549000000
H	7.521174000000	-0.971451000000	3.568615000000
H	5.726415000000	-2.400639000000	4.520790000000
H	3.453001000000	-2.345344000000	3.540670000000
H	1.135857000000	-3.116651000000	0.063542000000
H	1.027236000000	-5.013609000000	-1.504010000000
H	0.572879000000	-4.606079000000	-3.924190000000
H	0.217383000000	-2.274425000000	-4.710820000000
H	0.313126000000	-0.402646000000	-3.161295000000
C	-3.216773000000	1.336567000000	-0.839870000000
N	-3.801205000000	0.652397000000	-1.830613000000
N	-4.993598000000	0.106382000000	-1.445110000000
C	-5.127012000000	0.505429000000	-0.214179000000
N	-4.081569000000	1.284085000000	0.176397000000
C	-6.079653000000	0.316104000000	0.919290000000
C	-5.172429000000	0.684202000000	2.125064000000
C	-4.110938000000	1.685973000000	1.595286000000
C	-4.456612000000	3.170904000000	1.749405000000
C	-3.798995000000	3.789218000000	2.988441000000
C	-3.255395000000	0.334710000000	-3.114748000000
C	-2.849327000000	1.362904000000	-3.957853000000
C	-2.287287000000	1.032160000000	-5.185216000000
C	-2.160053000000	-0.305225000000	-5.556335000000
C	-2.579808000000	-1.317583000000	-4.696758000000
C	-3.122842000000	-1.005500000000	-3.455043000000
C	-4.142617000000	5.274434000000	3.080890000000
C	-4.182579000000	3.064921000000	4.279458000000
H	-6.930939000000	0.996433000000	0.813739000000
H	-6.457049000000	-0.705149000000	0.966733000000
H	-4.641324000000	-0.209203000000	2.460076000000
H	-5.741933000000	1.108452000000	2.953032000000
H	-3.126529000000	1.456376000000	2.006943000000
H	-5.547981000000	3.299572000000	1.799453000000
H	-4.102424000000	3.713001000000	0.867412000000
H	-2.712792000000	3.689676000000	2.847562000000
H	-2.964109000000	2.396900000000	-3.649321000000
H	-1.952736000000	1.818888000000	-5.852211000000
H	-1.721764000000	-0.557679000000	-6.516480000000
H	-2.456245000000	-2.358228000000	-4.975932000000
H	-3.399973000000	-1.764225000000	-2.732456000000
H	-3.633391000000	5.743590000000	3.927441000000
H	-3.852264000000	5.807972000000	2.171127000000
H	-5.220897000000	5.410594000000	3.222949000000
H	-3.707616000000	3.540100000000	5.142501000000

H -3.875573000000 2.014279000000 4.272011000000

H -5.267805000000 3.102857000000 4.435114000000

H 3.015440000000 -0.363945000000 -0.516952000000

H -1.167181000000 0.257343000000 -1.812847000000

----

*no*INTb5

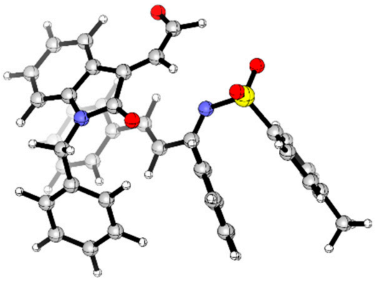

Zero-point correction= 0.622177 (Hartree/Particle)

Thermal correction to Energy= 0.662237

Thermal correction to Enthalpy= 0.663181

Thermal correction to Gibbs Free Energy= 0.547103

Sum of electronic and zero-point Energies= -2312.550746

Sum of electronic and thermal Energies= -2312.510686

Sum of electronic and thermal Enthalpies= -2312.509742

Sum of electronic and thermal Free Energies= -2312.625820

E(RM062X) = -2313.66619165

C 0.535807000000 2.709331000000 -1.495114000000

C 0.363430000000 3.984820000000 -0.765501000000

O 1.240889000000 4.542726000000 -0.139131000000

C 1.606217000000 1.895437000000 -1.458780000000

C 2.942999000000 1.907030000000 -0.858121000000

C 3.503339000000 0.630660000000 -1.089840000000

C 4.757527000000 0.279163000000 -0.622639000000

C 5.488766000000 1.255213000000 0.058344000000

C 3.693746000000 2.867099000000 -0.185257000000

C 4.973595000000 2.532721000000 0.261294000000

N 2.615143000000 -0.159056000000 -1.830987000000

C 1.458288000000 0.535006000000 -2.099386000000

O 0.503694000000 0.096557000000 -2.716209000000

C 1.072291000000 0.507983000000 1.411213000000

C 0.132067000000 -0.292297000000 0.874178000000

C -1.118907000000 0.200723000000 0.291108000000

N -1.355901000000 1.469757000000 0.324755000000

S -2.773661000000 2.170547000000 -0.262414000000

O -2.815781000000 3.472665000000 0.382798000000

O -2.787677000000 2.086635000000 -1.721080000000

C -4.109753000000 1.188886000000 0.380034000000

C -4.288669000000 1.119945000000 1.757233000000

C -5.289436000000 0.295779000000 2.259672000000

C -6.110285000000 -0.442108000000 1.402326000000

C -5.924140000000 -0.324953000000 0.021090000000

C -4.925086000000 0.486596000000 -0.499688000000

C -7.169619000000 -1.364705000000 1.945866000000

C 2.874222000000 -1.483202000000 -2.367659000000

C 2.449894000000 -2.596179000000 -1.435940000000

C 3.382873000000 -3.407700000000 -0.793380000000

C 2.962884000000 -4.409983000000 0.080246000000

C 1.604339000000 -4.602154000000 0.318372000000

C 0.664794000000 -3.801032000000 -0.331909000000

C 1.086416000000 -2.805906000000 -1.208415000000

C 2.342865000000 0.046235000000 1.971486000000

C 2.779038000000 -1.282504000000 1.841345000000

C 3.993161000000 -1.676743000000 2.382015000000

C 4.791494000000 -0.757701000000 3.065341000000

C 4.370891000000 0.561969000000 3.196937000000

C 3.157749000000 0.962093000000 2.646832000000

C -1.996772000000 -0.867268000000 -0.282030000000

C -2.538776000000 -1.827040000000 0.578408000000

C -3.344787000000 -2.839663000000 0.069504000000

C -3.575614000000 -2.920331000000 -1.303281000000

C -3.010430000000 -1.980414000000 -2.161182000000

C -2.229405000000 -0.942477000000 -1.657476000000

H 6.473953000000 1.004534000000 0.439270000000

H 5.153639000000 -0.720754000000 -0.763514000000

H 3.275844000000 3.850318000000 -0.017080000000

H 5.565324000000 3.274303000000 0.787196000000

H 0.881657000000 1.581343000000 1.435602000000

H -3.653700000000 1.701966000000 2.418469000000

H -5.438664000000 0.225477000000 3.333306000000

H -6.562614000000 -0.889362000000 -0.653328000000

H -4.752630000000 0.568682000000 -1.567462000000

H -7.316669000000 -1.216627000000 3.017532000000

H -8.127055000000 -1.204096000000 1.443157000000

H -6.887212000000 -2.410394000000 1.784998000000

H 4.444536000000 -3.255496000000 -0.973990000000

H 3.696890000000 -5.038479000000 0.575273000000

H 1.277242000000 -5.379243000000 1.001792000000

H -0.398684000000 -3.944969000000 -0.159465000000

H 0.361310000000 -2.172564000000 -1.716030000000

H 3.940189000000 -1.555300000000 -2.605208000000

H 2.308714000000 -1.547203000000 -3.302150000000

H 2.176641000000 -2.008204000000 1.303087000000

H 4.318923000000 -2.706009000000 2.264842000000

H 5.740833000000 -1.071565000000 3.488354000000

H 4.992105000000 1.284556000000 3.716427000000

H 2.835062000000 1.996488000000 2.728326000000

H -2.346835000000 -1.760604000000 1.646098000000

H -3.785978000000 -3.568064000000 0.742772000000

H -4.192720000000 -3.718859000000 -1.703089000000

H -3.179456000000 -2.049119000000 -3.230876000000

H -1.787481000000 -0.202898000000 -2.315839000000

H 0.269318000000 -1.367153000000 0.836235000000

H -0.331458000000 2.350413000000 -2.048154000000

H -0.658221000000 4.402563000000 -0.798789000000

----

^{no}TSb3

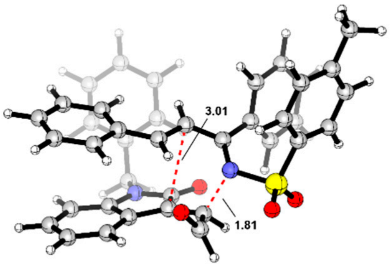

Frequency -407.1167

Zero-point correction= 0.623100 (Hartree/Particle)

Thermal correction to Energy= 0.661163

Thermal correction to Enthalpy= 0.662107

Thermal correction to Gibbs Free Energy= 0.553607

Sum of electronic and zero-point Energies= -2312.514834

Sum of electronic and thermal Energies= -2312.476772

Sum of electronic and thermal Enthalpies= -2312.475828

Sum of electronic and thermal Free Energies= -2312.584328

E(RM062X) = -2313.63034975

C -1.227518000000 -1.526967000000 -1.225236000000

C -0.810143000000 -0.582827000000 1.199543000000

C 0.050142000000 -2.140476000000 -1.293502000000

C 0.131049000000 -3.531573000000 -0.709113000000

H 0.541513000000 -2.007338000000 -2.259752000000

O -0.172351000000 -3.754555000000 0.438822000000

H 0.430609000000 -4.337636000000 -1.399500000000

C -2.564017000000 -1.937513000000 -0.826602000000

C -1.350204000000 -0.206652000000 -1.826216000000

C -3.414008000000 -0.830978000000 -1.096195000000

C -3.132535000000 -3.100514000000 -0.305898000000

C	-4.770461000000	-0.853930000000	-0.813352000000	H	6.422232000000	1.786969000000	3.005365000000
N	-2.664366000000	0.196031000000	-1.668911000000	C	-2.933502000000	2.586869000000	-1.134605000000
C	-5.310753000000	-2.028533000000	-0.287952000000	H	-4.186606000000	1.387819000000	-2.425328000000
H	-5.394381000000	0.015690000000	-0.994525000000	H	-2.558078000000	1.703652000000	-3.054512000000
C	-4.503390000000	-3.139993000000	-0.052470000000	C	-4.004124000000	3.093867000000	-0.401805000000
H	-6.371332000000	-2.071743000000	-0.062695000000	C	-1.644911000000	3.078256000000	-0.895281000000
H	-2.517355000000	-3.964523000000	-0.082944000000	C	-3.796593000000	4.072603000000	0.569733000000
H	-4.941365000000	-4.046917000000	0.351524000000	H	-5.006677000000	2.713255000000	-0.583295000000
C	-3.132181000000	1.482410000000	-2.149694000000	C	-2.511099000000	4.544657000000	0.818732000000
O	-0.451451000000	0.479951000000	-2.315393000000	H	-4.638931000000	4.462296000000	1.133670000000
C	0.017500000000	0.403458000000	0.718964000000	C	-1.435708000000	4.048149000000	0.080983000000
C	-2.096608000000	-0.369237000000	1.835402000000	H	-2.348021000000	5.302008000000	1.578827000000
H	-0.436293000000	-1.604780000000	1.204738000000	H	-0.430247000000	4.416054000000	0.264462000000
C	1.136171000000	0.086717000000	-0.073574000000	H	-0.811488000000	2.671696000000	-1.464218000000
H	-0.235760000000	1.454320000000	0.814552000000	C	-2.736014000000	0.882145000000	1.862229000000
N	1.316265000000	-1.210589000000	-0.399388000000	C	-2.752142000000	-1.482464000000	2.384073000000
C	2.041653000000	1.200812000000	-0.463499000000	C	-3.995512000000	1.006406000000	2.428688000000
S	2.803669000000	-1.786441000000	-1.011803000000	H	-2.258608000000	1.755380000000	1.427542000000
O	2.695150000000	-3.227936000000	-0.820887000000	C	-4.640256000000	-0.110165000000	2.962938000000
O	3.104535000000	-1.284745000000	-2.345510000000	H	-4.481836000000	1.976479000000	2.438346000000
C	3.958373000000	-1.101754000000	0.140794000000	C	-4.016870000000	-1.354718000000	2.942376000000
C	3.836020000000	-1.416494000000	1.489846000000	H	-5.631642000000	-0.007420000000	3.393489000000
C	4.942488000000	-0.241280000000	-0.332114000000	H	-4.520820000000	-2.226409000000	3.346612000000
C	4.717208000000	-0.827036000000	2.388496000000	H	-2.261462000000	-2.451046000000	2.341937000000
H	3.059410000000	-2.098860000000	1.822628000000	C	2.561487000000	1.997725000000	0.562267000000
C	5.710553000000	0.054164000000	1.949712000000	C	2.363394000000	1.482611000000	-1.795581000000
H	4.634041000000	-1.053740000000	3.447283000000	C	3.425176000000	3.046760000000	0.266362000000
C	5.817046000000	0.329472000000	0.582813000000	H	2.308945000000	1.766297000000	1.593561000000
C	6.643542000000	0.717576000000	2.928398000000	C	3.761530000000	3.313978000000	-1.058517000000
H	6.584448000000	1.014347000000	0.232904000000	H	3.838032000000	3.650002000000	1.068828000000
H	4.988577000000	-0.009259000000	-1.391064000000	C	3.222179000000	2.538250000000	-2.083508000000
H	6.551797000000	0.282000000000	3.925169000000	H	4.434112000000	4.132915000000	-1.293965000000
H	7.683593000000	0.621394000000	2.605569000000	H	3.463830000000	2.761320000000	-3.117737000000

H 1.918946000000 0.886329000000 -2.582609000000

----

*no*INTb6

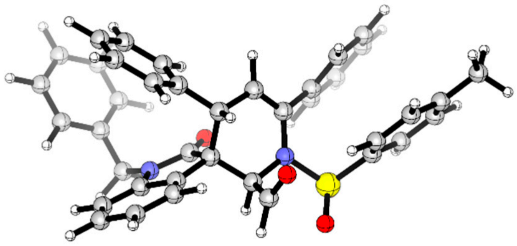

Zero-point correction= 0.626835 (Hartree/Particle)

Thermal correction to Energy= 0.664802

Thermal correction to Enthalpy= 0.665747

Thermal correction to Gibbs Free Energy= 0.555192

Sum of electronic and zero-point Energies= -2312.571173

Sum of electronic and thermal Energies= -2312.533205

Sum of electronic and thermal Enthalpies= -2312.532261

Sum of electronic and thermal Free Energies= -2312.642816

E(RM062X) = -2313.69058468

C	-0.905213000000	-1.140893000000	-0.164010000000
C	-0.623023000000	-0.640719000000	1.288207000000
C	0.404788000000	-1.598885000000	-0.844761000000
C	0.857179000000	-2.929849000000	-0.240973000000
H	0.194259000000	-1.770788000000	-1.905936000000
O	1.399030000000	-3.004835000000	0.835187000000
H	0.605364000000	-3.830917000000	-0.829291000000
C	-1.983980000000	-2.203518000000	-0.168196000000
C	-1.539426000000	-0.038570000000	-1.042149000000
C	-3.093382000000	-1.719044000000	-0.873733000000
C	-2.057372000000	-3.427005000000	0.476505000000
C	-4.268417000000	-2.448275000000	-0.976691000000
N	-2.800299000000	-0.457469000000	-1.398490000000
C	-4.317171000000	-3.692658000000	-0.344281000000
H	-5.126769000000	-2.068546000000	-1.520109000000
C	-3.231909000000	-4.179994000000	0.376321000000
H	-5.225850000000	-4.282194000000	-0.410074000000
H	-1.220532000000	-3.796849000000	1.063447000000
H	-3.297782000000	-5.143553000000	0.869129000000
C	-3.664668000000	0.372187000000	-2.222435000000
O	-1.038340000000	1.005567000000	-1.406690000000
C	0.479789000000	0.374590000000	1.243134000000
C	-1.889981000000	-0.105216000000	1.929572000000
H	-0.301334000000	-1.528113000000	1.850719000000
C	1.410274000000	0.407085000000	0.281731000000
H	0.453608000000	1.199082000000	1.946518000000
N	1.446238000000	-0.575477000000	-0.734567000000
C	2.348457000000	1.556040000000	0.184004000000
S	2.811290000000	-0.909434000000	-1.688130000000
O	2.667797000000	-2.333489000000	-1.986214000000
O	2.921466000000	0.025319000000	-2.793960000000
C	4.179231000000	-0.647835000000	-0.592397000000
C	4.223127000000	-1.342341000000	0.611924000000
C	5.155884000000	0.272799000000	-0.954200000000
C	5.278391000000	-1.088550000000	1.479278000000
H	3.430573000000	-2.036588000000	0.878282000000
C	6.275520000000	-0.163984000000	1.152865000000
H	5.324355000000	-1.609870000000	2.431147000000
C	6.202404000000	0.506243000000	-0.071959000000
C	7.418022000000	0.102880000000	2.097028000000
H	6.964951000000	1.236805000000	-0.327540000000
H	5.062876000000	0.814799000000	-1.889201000000
H	7.166719000000	-0.191495000000	3.118165000000
H	8.307318000000	-0.460419000000	1.796329000000
H	7.685457000000	1.162733000000	2.099208000000
C	-4.373197000000	1.455472000000	-1.438827000000

H	-4.383482000000	-0.285431000000	-2.719047000000
H	-3.028701000000	0.824215000000	-2.988713000000
C	-5.597657000000	1.205860000000	-0.819177000000
C	-3.795803000000	2.722316000000	-1.328873000000
C	-6.249885000000	2.213143000000	-0.113836000000
H	-6.047029000000	0.218533000000	-0.892829000000
C	-5.682192000000	3.482054000000	-0.028471000000
H	-7.204873000000	2.010492000000	0.360513000000
C	-4.450936000000	3.733170000000	-0.630754000000
H	-6.196047000000	4.272571000000	0.509626000000
H	-4.001000000000	4.718458000000	-0.558876000000
H	-2.828429000000	2.901402000000	-1.789425000000
C	-2.353441000000	1.178736000000	1.628938000000
C	-2.639716000000	-0.908501000000	2.791002000000
C	-3.545965000000	1.643896000000	2.174462000000
H	-1.783184000000	1.809782000000	0.951146000000
C	-4.290141000000	0.832819000000	3.026315000000
H	-3.898628000000	2.637274000000	1.918901000000
C	-3.832728000000	-0.444468000000	3.337283000000
H	-5.223728000000	1.196328000000	3.444537000000
H	-4.405117000000	-1.082710000000	4.003101000000
H	-2.287289000000	-1.909321000000	3.027490000000
C	3.171634000000	1.881459000000	1.264373000000
C	2.376995000000	2.339234000000	-0.973711000000
C	4.038440000000	2.965443000000	1.179767000000
H	3.151704000000	1.255016000000	2.151317000000
C	4.082002000000	3.733445000000	0.018223000000
H	4.688464000000	3.202946000000	2.016367000000
C	3.245420000000	3.423128000000	-1.052261000000
H	4.763774000000	4.575360000000	-0.051359000000
H	3.266317000000	4.028845000000	-1.952580000000
H	1.724080000000	2.083064000000	-1.801499000000
